# Rhodium(i)-catalyzed stereoselective [4+2] cycloaddition of oxetanols with alkynes through C(sp^3^)–C(sp^3^) bond cleavage†
†Electronic supplementary information (ESI) available. CCDC 1512439 and 1512440. For ESI and crystallographic data in CIF or other electronic format see DOI: 10.1039/c6sc05246k



**DOI:** 10.1039/c6sc05246k

**Published:** 2017-01-23

**Authors:** Rui Guo, Xinxin Zheng, Dayong Zhang, Guozhu Zhang

**Affiliations:** a State Key Laboratory of Organometallic Chemistry , Shanghai Institute of Organic Chemistry , Chinese Academy of Sciences , 345 Lingling Road , Shanghai 200032 , P. R. China . Email: guozhuzhang@sioc.ac.cn; b University of Chinese Academy of Sciences , Beijing , 100049 , China; c Institute of Pharmaceutical Science , China Pharmaceutical University , Nanjing , P. R. China . Email: cpuzdy@163.com

## Abstract

An efficient and convenient synthesis of highly functionalized dihydropyrans has been achieved through rhodium(i)-catalysed tandem C(sp^3^)–C(sp^3^) bond cleavage and annulation of oxetanols with alkynes. An enantioselective version was enabled using a Binaphine ligand. Excellent site-selectivity and remarkable enantioretention are obtained for 2-substituted oxetanols.

## Abstract 2

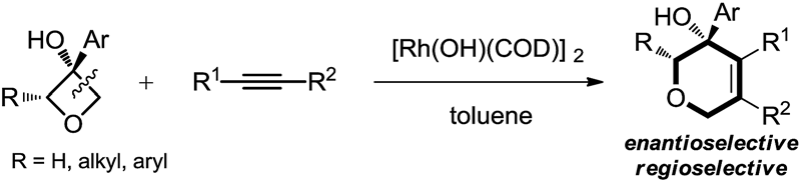
An efficient and convenient synthesis of highly functionalized dihydropyrans has been achieved through rhodium(i)-catalysed tandem C(sp^3^)–C(sp^3^) bond cleavage and annulation of oxetanols with alkynes.

## Introduction

**Fig. 1:**
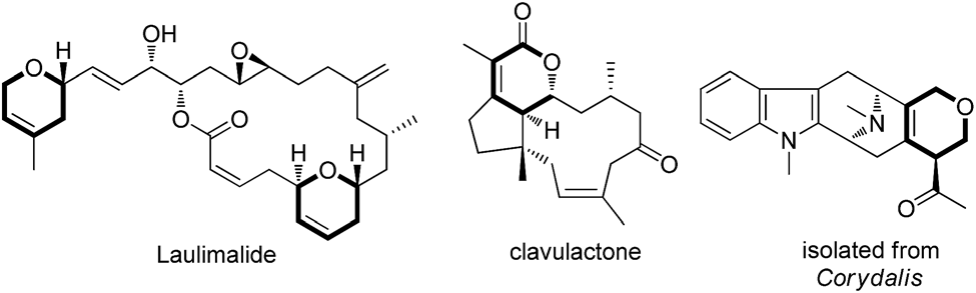
Representative natural products.

Dihydropyran and its derivatives are ubiquitous molecular skeletons, which are widely observed in natural products^1^ (Fig. 1), and are advanced intermediates that can lead to substances of biological or medicinal importance.^2^ Thus, new methods which enable convenient access to this type of motif in a step-economic, flexible and stereoselective fashion are highly demanded.

The selective cleavage and functionalization of carbon–carbon single bonds by transition-metal catalysts has undergone significant progress in recent years, allowing ready access to a variety of synthetically useful molecular scaffolds.^3^ Among those, cyclobutanols are frequently employed as privileged building blocks for the construction of complex cyclic molecules.^4^ In 2012, Murakami and co-workers reported seminal work on rhodium-catalyzed tandem C–C single bond cleavage/formal cycloaddition of benzocyclobutanols with alkynes [eqn (1)].^4*b*^ Since then, vinyl ketones,^5^ carbene precursors,^6^ and allenes^7^ have been proven to be suitable cycloaddition counterparts, demonstrating the broad applicability of this protocol [eqn (2)–(4)]. However, to the best of our knowledge, under rhodium catalyzed cycloaddition with unsaturated 2-π systems, ring opening of cyclobutanols exclusively occurred on the C(sp^2^)–C(sp^3^) bond adjacent to the hydroxyl group, with subsequent addition of the ipso carbon [*i.e.* (sp^2^)C] to the 2-π units.

Previous work: Rh catalysed C–C bond cleavage of 4-membered *tert*-alcohols1
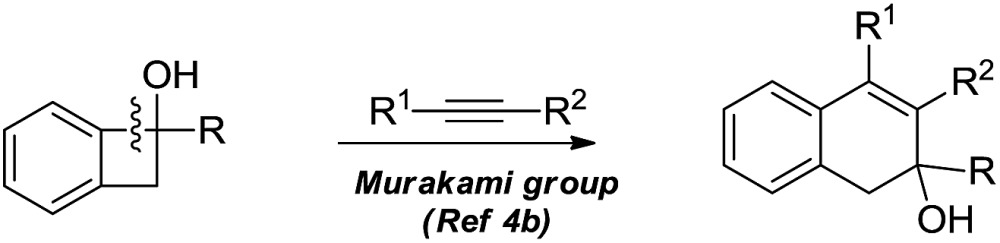

2
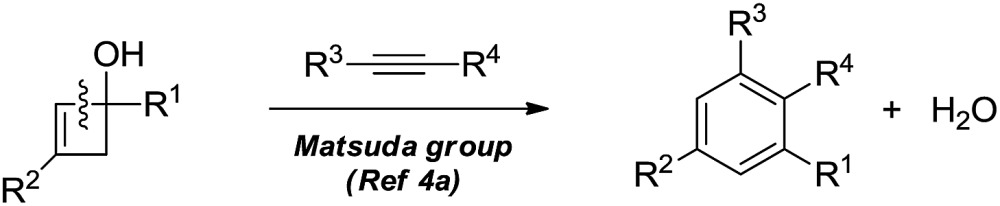

3
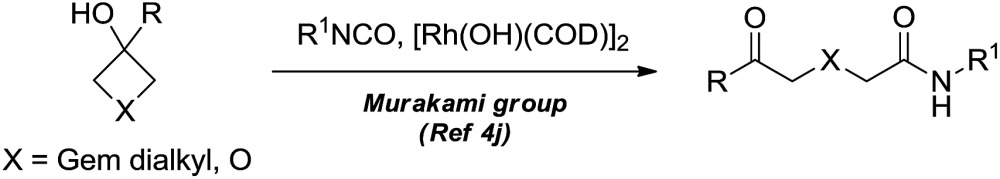

4
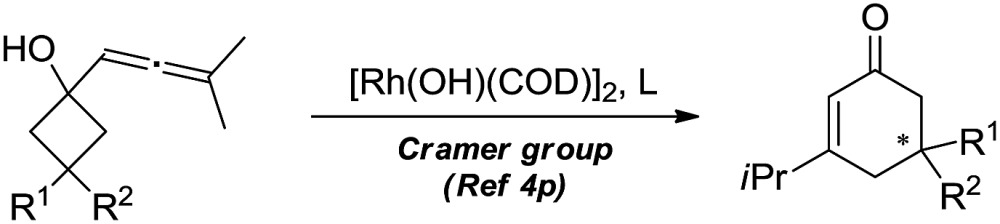



This work: C(sp^3^)–C(sp^3^) cleavage and cycloaddition5
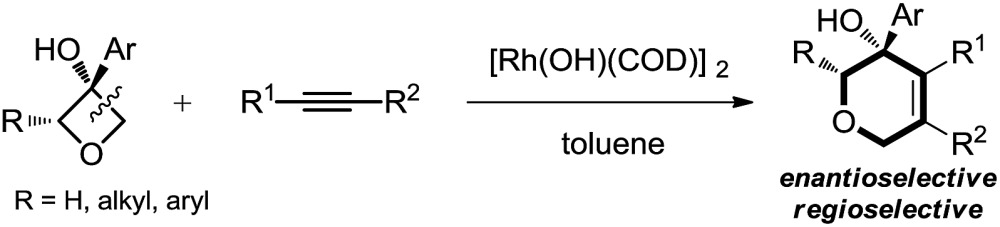



Therefore, the means to alter the hybridized nature of the additional carbon would offer a unique opportunity for chemists and would add to the fast expanding synthetic tool box, allowing the facile assembly of important molecular skeletons which are not easy to construct by conventional methods. Encouraged by the considerable medicinal utility and unique molecular skeleton of oxetane and its derivatives,^8^ we envisioned that they set a good stage for exploring new reactions. Herein, we report the highly efficient rhodium(i)-catalyzed cycloaddition of oxetanols with alkynes, *via* a tandem C(sp^3^)–C(sp^3^) bond cleavage and subsequent cross addition over a 2-π unit for the first time, leading to complex dihydropyran ring systems. Binaphine proves to be a viable chiral phosphine ligand to promote the enantioselective version of this transformation. Furthermore, for 2-substituted oxetanols, it was found that the ring opening selectively took place on the nonsubstituted side, with the subsequent ring closing proceeding in a highly diastereoselective manner [eqn (5)].

## Results and discussion

We initiated our studies by exploring the reaction of 3-phenyloxetan-3-ol, which was readily prepared from oxetan-3-one and 1,2-diphenylethyne (Table 1). After numerous trials, [Rh(OH)(COD)]_2_ proved to be the most effective catalyst, and the [4+2] cyclized product **2a** was obtained in excellent yield (Table 1, entry 1). Emphasis was then put on the use of commercial chiral ligands for asymmetric carbon–carbon bond formation. BINAP and Segphos were not effective (Table 1, entry 2 and 3), with moderate yields and negligible enantiomer ratios being obtained. The reaction using Josiphos proceeded in good yield, albeit with only moderate enantioselectivity (Table 1, entry 4). To our delight, a much improved enantioselectivity of 82 : 18 er was obtained for **2a** by using ^i^Pr-Duphos as the ligand (Table 1, entry 5). Further ligand examination identified Binaphine as the most effective ligand among those tested, which gave **2a** in 91 : 9 er. Interestingly, analogous to Cramer's observations,^4*i*^ the addition of 1.1 equiv. of K_2_CO_3_ significantly improved the reaction kinetics. Shortened reaction times and comparable product yields were achieved, suggesting base-facilitated ring opening and alkyne insertion. The er was further improved while lowering the temperature. Eventually, a 70% yield with 96.5 : 3.5 er of **2a** was obtained when the reaction was conducted at 30 °C, however, a prolonged reaction time (72 hours) was required for full conversion of the starting material.

**Table 1:** Optimization of the reaction conditions

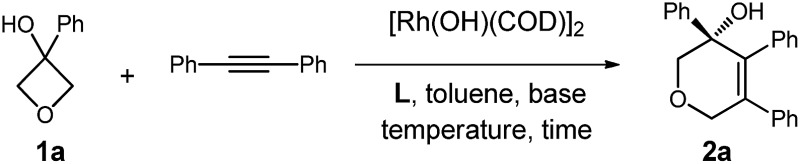						
Entry	Ligand	Base	Temperature	Time	Yield^*a*^ [%]	er^*b*^
1	—	—	110 °C	6 h	96	—
2	**L1**	—	110 °C	6 h	75	55 : 45
3	**L2**	—	110 °C	6 h	50	54 : 46
4	**L3**	—	110 °C	6 h	45	37 : 63
5	**L4**	—	110 °C	6 h	95	91 : 9
6^*c*^	**L5**	K_2_CO_3_	110 °C	6 h	90	90.5 : 8.5
7^*c*^	**L5**	K_2_CO_3_	70 °C	12 h	86	94 : 6
8^*c*^	**L5**	K_2_CO_3_	50 °C	24 h	85	94 : 6
9^*c*^	**L5**	K_2_CO_3_	30 °C	72 h	70	96.5 : 3.5
10	**L5**	—	30 °C	72 h	—	—
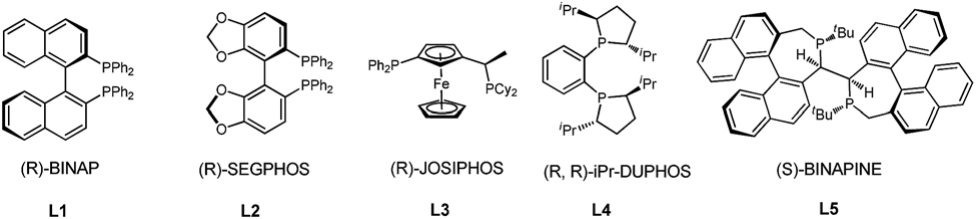						

^*a*^Isolated yields.

^*b*^The er was determined by chiral HPLC analysis. The absolute configuration of the products was assigned by single crystal X-ray analysis of **2i**.

^*c*^1.1 equiv. of K_2_CO_3_ was used.

With the optimized conditions in hand, we demonstrated the general applicability of our method with a range of oxetanols and alkynes (Table 2). We first evaluated the reactions of different oxetanols with diphenylethyne. Various arylated oxetanols bearing *ortho*-, *para*- and *meta*-substituted phenyl groups reacted smoothly to give the desired products in moderate to good yields with high enantioselectivities (er > 95 : 5). The substituted groups could be alkyl, methoxy, or fluoro groups. It needs to be pointed out that alkyl and alkenyl oxetanols did not undergo cycloaddition with alkynes under the current reaction conditions. The variations of diaryl alkynes were then briefly investigated and, to our delight, it was observed that alkynes with electron-donating methyl and methoxy groups at the *para* position can serve as suitable substrates and the corresponding dihydropyrans were isolated in moderate yields with high enantiomeric ratios.

**Table 2:** Scope studies: enantioselective cycloadditions^*a*^ ^,^ ^*b*^

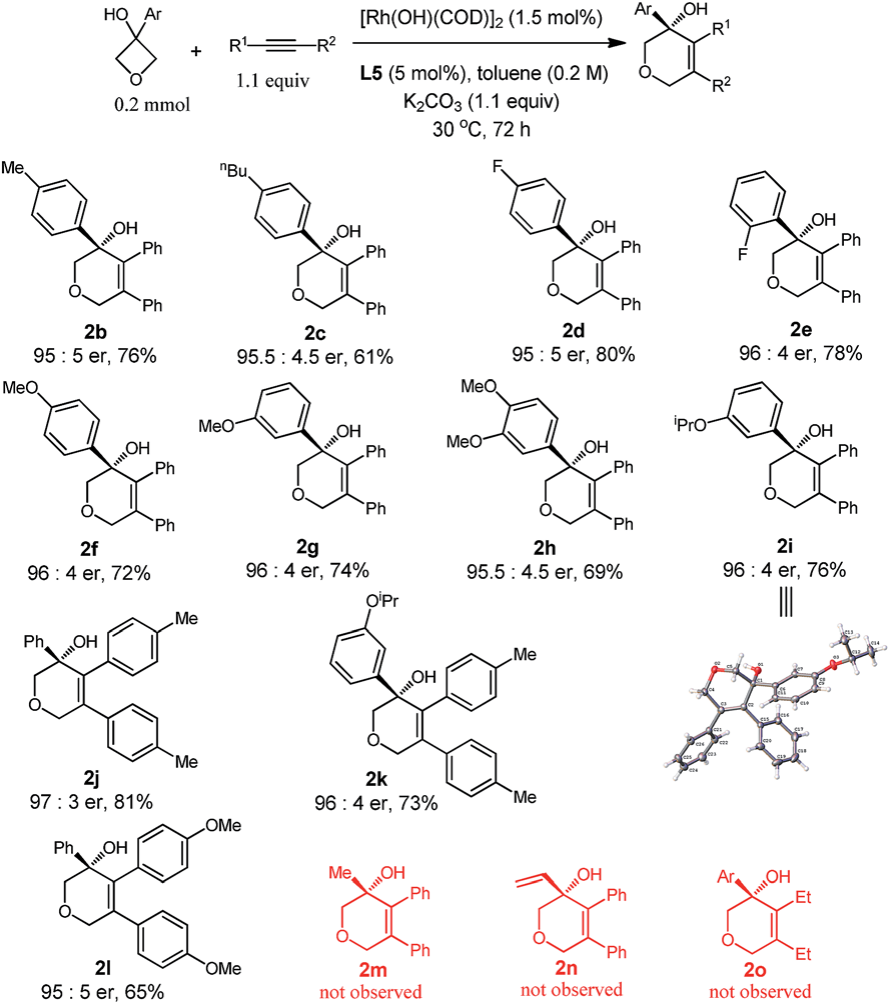

^*a*^Reaction conditions: oxetanol (0.2 mmol), alkyne (1.1 equiv.), [Rh(OH)(COD)]_2_ (1.5 mol%), **L5** (5.0 mol%), K_2_CO_3_ (1.1 equiv.), toluene (0.2 M), 30 °C, 72 h.

^*b*^Isolated yield.

The attempts to further expand the substrate scope by changing the aryl substitution of cyclobutanols to alkyl or alkenyl groups were not successful (**2m** and **2n**), suggesting that the aryl group might provide extra coordination to facilitate the β-carbon elimination.^4b,p^ The reaction of **1a** with 3-hexyne did proceed to give the racemic product **2o**, however, no cyclization took place in the presence of the chiral ligand, probably due to the incompatibility of the ligand and the substrate.^9^

To address the question of regioselectivity in the ring opening of 2-substituted oxetanols, we synthesized a series of 2-substituted oxetanones from propargyl alcohols using Zhang's gold catalyzed procedure^10^ and treated them with the corresponding Grignard reagents or aryl lithium reagents. Gratifyingly, single diastereomers were obtained in high yields in all of the reactions of 2-substituted oxetanols with alkynes (Table 3). The above experimental result suggests that preferential cleavage of the C–C single bond between the hydroxyl carbon atom and the unsubstituted carbon atom of oxetanol takes place to afford the heterocyclic product. The scope of the site-selective insertion reaction is shown in Table 3. Various 2-alkylated phenyloxetan-3-ols bearing different linear alkyl, phenyl ethyl, and cyclic hexyl groups are cyclized readily with 1,2-diphenylethyne, giving the corresponding tetrahydropyrans in good yields as a single diastereomer (**4a**, **4b** and **4f**).

**Table 3:** Scope studies: cycloaddition of 2-substituted oxetanols^*a*^ ^,^ ^*b*^

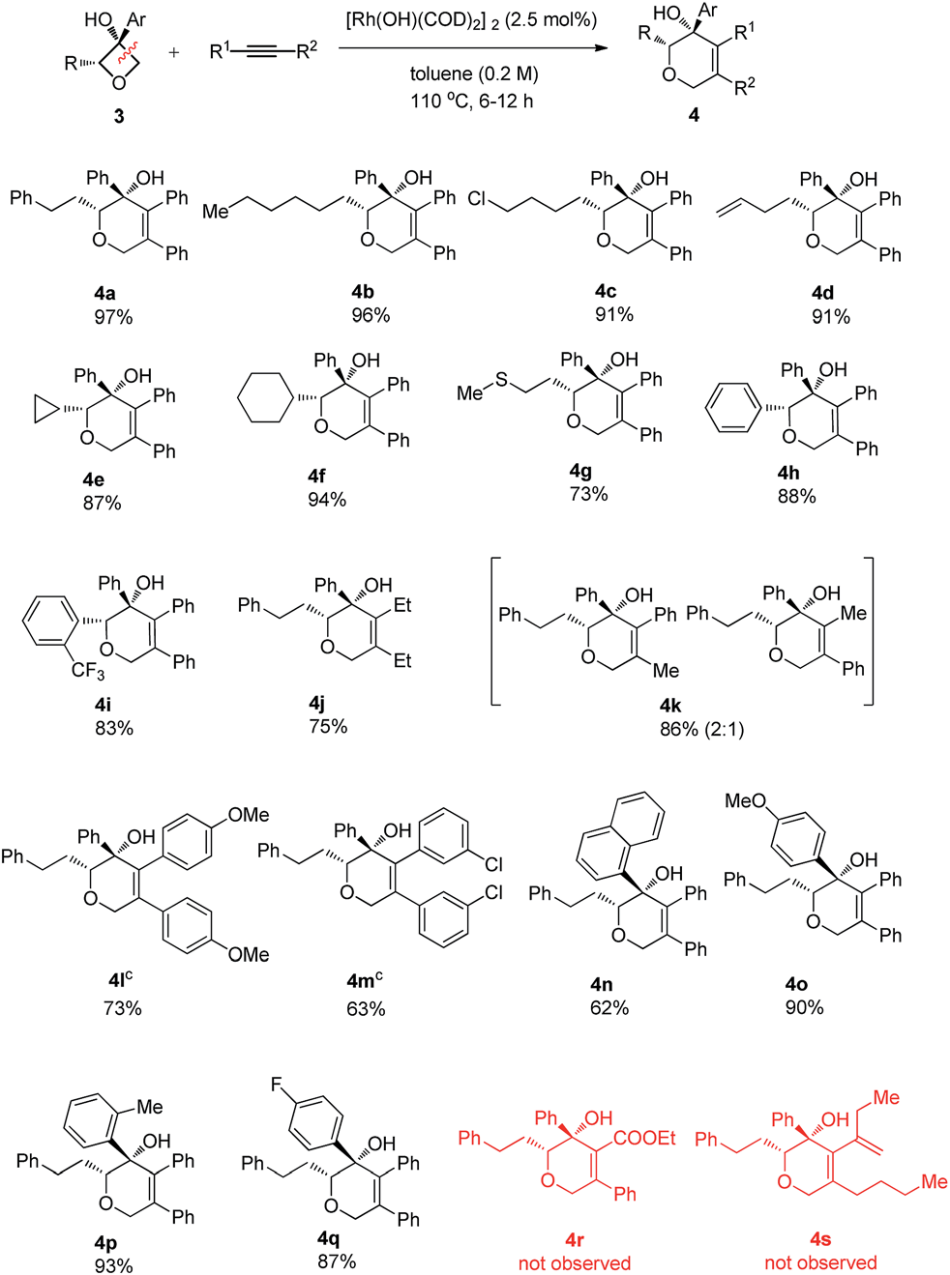

^*a*^Reaction conditions: 2-substituted oxetanol (0.2 mmol), alkyne (1.1 equiv.), [Rh(OH)(COD)]_2_ (2.5 mol%), toluene (0.2 M), 110 °C, 6 h.

^*b*^Isolated yield.

^*c*^Reaction took 12 hours at 110 °C.

Notably, a cyclopropyl group which is sensitive to rhodium(i)-catalyzed reactions could also survive under the current conditions (**4e**). The relative configuration of the complex product was established by single crystal X-ray analysis of the product **4a**-*ent*. Furthermore, terminal chloro, C–C double bond and methylthio groups remained intact under the optimized conditions (**4c**, **4d** and **4g**). Phenyl phenyloxetan-3-ol was also successfully engaged in the insertion reaction (**4h**). The site selective ring cyclization was observed even when a sterically demanding *ortho*-trifluoromethyl group was present (**4i**). Variations of the alkynes were also briefly investigated. The nature of the aryl substitution (electron-donating or -withdrawing) does not seem to affect the reaction (**4l** and **4m**). Hex-3-yne was a good substrate for this reaction as well (**4j**). The reaction of an unsymmetric alkyne bearing methyl and phenyl substitution groups (**3k**) gave two inseparable regioisomers at a ratio of 2 : 1; this result indicates that the electronic and steric properties of the alkyne substitution groups have less effect on the site-selectivity compared to that in the Rh(i)-catalyzed cycloaddition of benzocyclobutanol with the same alkyne.^4b,11^ The aryl group at the 3 position of oxetanol could be functionalized, and naphthalene, *para*-methoxy phenyl, *para*-fluoro phenyl and *ortho*-methyl phenyl groups were all well tolerated (**4n**, **4o**, **4p** and **4q**). A further substrate scope study showed that no desired cyclized product was observed from the reaction of ethyl 3-phenylpropiolate and 1,3-enyne (**4r** and **4s**), and these results further suggest that the alkyl-rhodium species is more sensitive to electronic properties and steric hindrance than (sp^2^)C–rhodium species.^9^
6
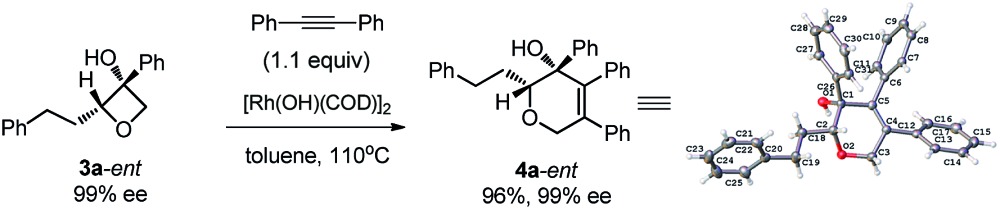

7
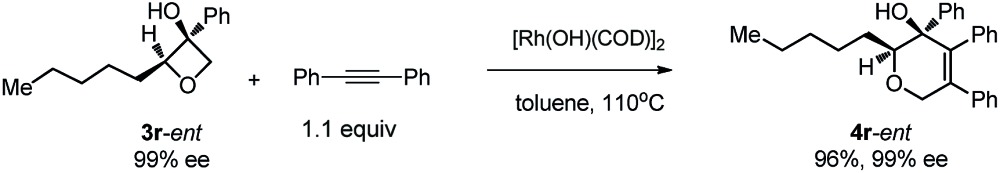

8
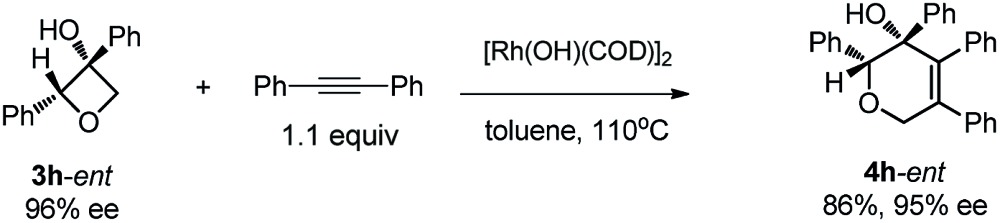



Excellent retention of enantiomeric purity was observed when enantiomerically pure 2-substituted oxetanols were used. Despite the potential epimerization challenges,^7,12^ the cycloaddition worked effectively to afford enantio-enriched cycloadducts in high yields [eqn (6)–(8)]. Both 2-alkyl substrates (**3a**-*ent* and **3r**-*ent*) and a 2-aryl substrate (**3h**-*ent*) afforded regioselective cycloadducts in high yields with excellent enantioretention.

**Scheme 1:**
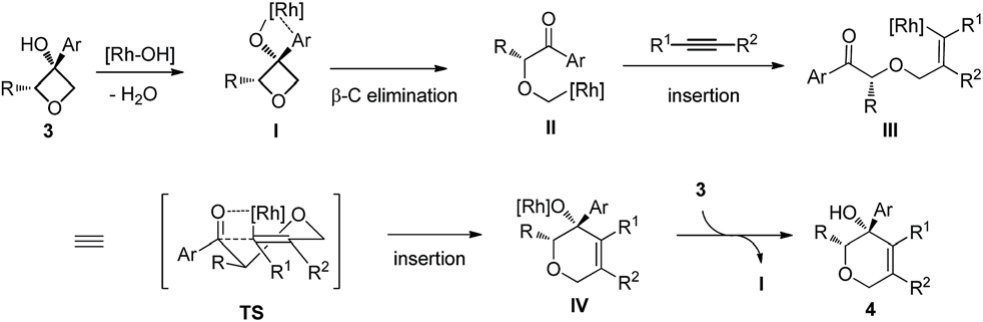
Proposed reaction mechanism.

Based on previous studies and our own observations, a tentative mechanism is proposed (Scheme 1). First, simultaneous coordination of the Rh(i) center to both the hydroxy group and the arene moiety should be favored.^4b,p^ Site-selective ring opening through β-carbon elimination should lead to **II**. Next, a *cis*-migratory insertion of C(sp^3^)–Rh(i) across the alkyne occurs to give **III**.^4a,b^ The last ring closing takes place in a highly stereoselective manner. A four-center interaction of the carbonyl moiety with the carbon–rhodium bond might be involved in the transition state.^13^

**Scheme 2:**
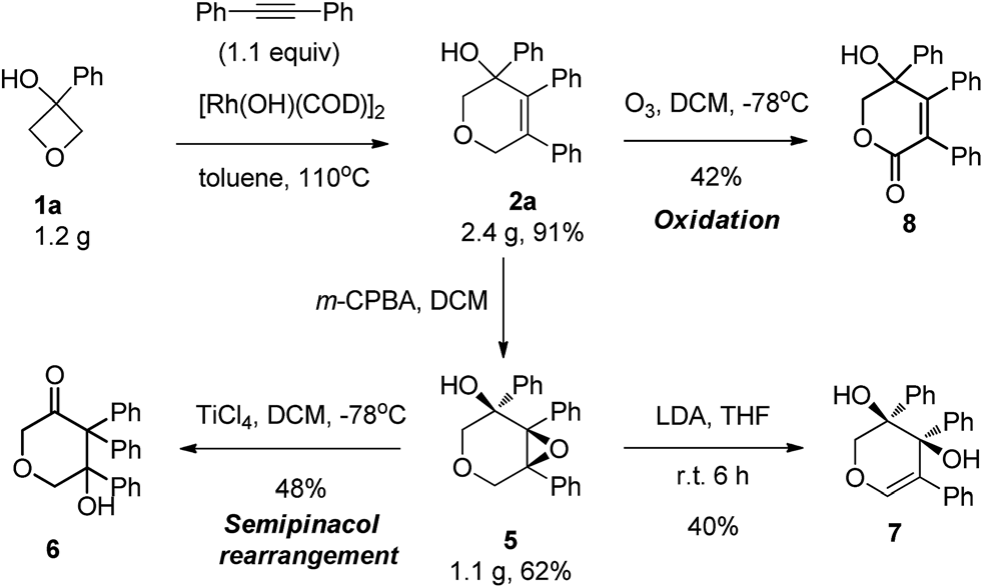
Synthetic utilities of dihydropyrans.

This reaction could be carried out at gram scale with the same high efficiency (Scheme 2). This series of highly functionalized dihydropyran products are synthetically versatile building blocks. For example, epoxidation of the alkene using *m*-CPBA produced the corresponding epoxide **5** in 62% yield with perfect diastereoselectivity (>20 : 1 dr) at gram scale. Treatment of the epoxide with a Lewis acid produced the ketone **6** in 78% yield through a semipinacol rearrangement. In contrast, treatment with strong base induced epoxide ring opening, giving rise to allylic alcohol **7**. Our attempt to cleave the double bond by ozonolysis failed to provide the ring opening product, with lactone **8** being isolated instead, and so represented another type of important molecular scaffold which would be difficult to access using other methods.^14^

## Conclusions

In summary, an efficient method has been developed to prepare highly functionalized dihydropyrans through ring opening of arylated oxetanols and cyclization with alkynes by Rh(i) catalysis. A high degree of enantioselective control was realized when using the chiral ligand Binaphine.^15^ Excellent site-selectivity and diastereoselectivity were observed in the cases of 2-substituted oxetanols. A high degree of retention of enantiomeric purity in the products was achieved in the reactions using optically pure oxetanols. The synthetic potential of the products was demonstrated in a gram scale operation and 4 facile derivatization reactions.
