# Supplementary material for: Rhodium(i)-catalyzed stereoselective [4+2] cycloaddition of oxetanols with alkynes through C(sp3)–C(sp3) bond cleavage
Source: Chem Sci. 2017 Jan 23;8(4):3002–6. doi: 10.1039/c6sc05246k (PMC5380880; doi:10.1039/c6sc05246k)
Supplement: Supplementary file 1 [file SC-008-C6SC05246K-s001.pdf]

Electronic Supplementary Material (ESI) for Chemical Science.
This journal is © The Royal Society of Chemistry 2017

SUPPORTING INFORMATION

Rhodium(I)-Catalyzed Stereoselective [4+2] Cycloaddition of Oxetanols with alkynes through $C(sp^3)$-$C(sp^3)$ bond cleavage

Rui Guo[a,b] , Xinxin Zheng[c], Dayong Zhang[c], Guozhu Zhang[a,b]

[a] State Key Laboratory of Organometallic Chemistry, Shanghai Institute of Organic Chemistry, Chinese Academy of Sciences, 345 Lingling Road, Shanghai 200032, P. R. China;

[b] University of Chinese Academy of Sciences, Beijing, 100049, China

[c] Institute of Pharmaceutical Science, China Pharmaceutical University, Nanjing, China

CONTENTS

1. General Experiment Information **2**
2. General Procedures **3**
3. Unsuccesful cases **5**
4. Synthesis and Characterization of Materials **7**
5. Synthesis and Characterization of Products **18**
6. Reference **36**

1. General Experiment Information

NMR spectra were recorded at room temperature on the following spectrometers: Agilent (400 MHz) and VARIAN (400 MHz). Chemical shifts are given in ppm and coupling constants in Hz. ^{1}H spectra were calibrated in relation to the reference measurement of TMS (0.00 ppm). ^{13}C spectra were calibrated in relation to deuterated solvents, namely $CDCl_3$ (77.16 ppm). The following abbreviations were used for ^{1}H NMR spectra to indicate the signal multiplicity: s (singlet), d (doublet), t (triplet), q (quartet) and m (multiplet) as well as combinations of them. When combinations of multiplicities are given the first character noted refers to the largest coupling constant. High performance liquid chromatography (HPLC) was carried out with Agilent 1260 Infinity on a UV spectrophotometric detector (210 nm, Agilent). For DART-HR and $EI^{-}HR$ (GC-TOF) spectrometer was applied. Infrared Spectroscopy (IR) was processed on an FT-IR spectrometer named Nicolet 380. The method is denoted in brackets. For the most significant bands the wave number ṽ (cm^{-1}) is given.

Chemicals were purchased from commercial suppliers, all solvents of flash silica gel column chromatography were purchased from "Adamas-beta". Unless stated otherwise, all the substrates and solvents were purified and dried according to standard methods prior to use. Reactions requiring inert conditions were carried out in glove box.

2. General Procedures

(1) General Procedure A： Synthesis of starting materials

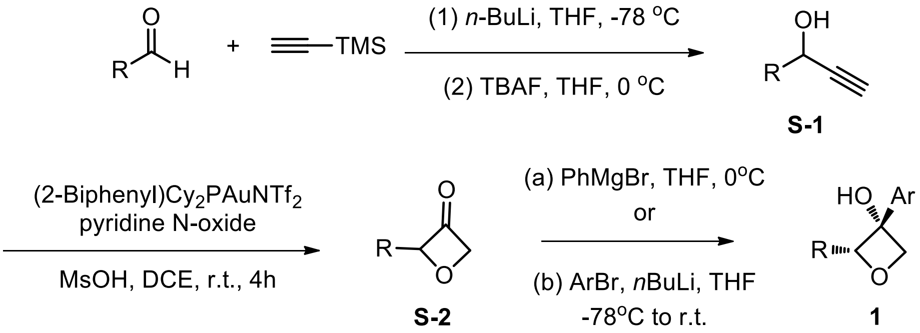

Synthesis of **S-1**

(1) A solution of *n*-butyllithium in hexanes (2.5 M, 12.8 mL, 32.0 mmol) was added dropwise to a solution of (trimethylsilyl)acetylene (30.0 mmol) in anhydrous THF (100 mL) at -78 °C. After 15 min, a solution of an aldehyde (20.0 mmol) in anhydrous THF (10 mL) was added dropwise to the reaction mixture, and the resulting mixture was further stirred at the same temperature for 3 h. The reaction was quenched with saturated NH_4Cl solution, and the mixture was extracted three times with Et_2O. The combined organic layers were washed with brine, dried with anhydrous $MgSO_4$, and the solvents were evaporated under vacuum to obtain the oily residue.

(2) A solution of tetrabutylammonium fluoride (1.0 M, 24.0 mL, 24.0 mmol) was added dropwise to a solution of oily residue in THF (50 mL) at 0 °C and the mixture was stirred at 0 °C for 15 min. After addition of an aqueous NH_4Cl solution, the mixture was extracted three times with Et_2O. The combined organic layers were washed with brine, dried with anhydrous $MgSO_4$, and the solvents was evaporated under vacuum. The oily residue was purified by flash silica gel column chromatography (hexanes/EtOAc=10:1) to yield propargylic alcohols **S-1**.

Synthesis of **S-2**

According to a procedure reported by Limin Zhang et al.[1] Pyridine *N*-oxide (6.0 mmol), MsOH (18.0 mL, 0.20 M in DCE), and (2-biphenyl)$Cy_2PAuNTf_2$ (125 mg, 0.15 mmol) were added sequentially to a solution of secondary propargyl alcohol **S-1** (3.0 mmol) in DCE (42 mL) at room temperature. The reaction mixture was stirred at r.t. and the progress of the reaction was monitored by TLC. The reaction typically took 3 – 4 h. Upon completion, the reaction was treated with saturated aqueous $NaHCO_3$ (15 mL), and the resulting solution was extracted with DCM (2 × 30 mL). The combined organic layers were dried with $MgSO_4$. The mixture was concentrated and the residue was purified by silica gel flash chromatography (hexanes/EtOAc=5:1) to afford desired products **S-2**.

Synthesis of **1**

(a) To a solution of oxetan-3-one **S-2** (1.0 mmol) in anhydrous THF (5 ml) at 0 °C was

added a solution of phenylmagnesium bromide in diethyl ether (3.0 M, 0.37 mL, 1.1 mmol). The resulting mixture was further stirred at the same temperature for 2 h. The reaction was quenched with saturated NH_4Cl solution, and the mixture was extracted three times with Et_2O. The combined organic phases were dried over $MgSO_4$, concentrated in vacuo and the residue was purified by silica gel flash chromatography (hexane/EtOAc = 5:1) to give pure product **1** as white solid.

(b) To a solution of aryl bromide (3.0 mmol) in 10 mL dry THF was added *n*-butyllithium in hexanes (2.5 M, 1.4 mL, 3.4 mmol) at -78 ^{o}C. After the addition was finished, the mixture was stirred for 2.5 h, before a solution of oxetan-3-one **S-2** (2.0 mmol) in 3 mL anhydrous THF was added at -78 ^{o}C. The mixture was allowed to warm to room temperature over night and quenched with saturated NH_4Cl solution. The aqueous phase was extracted twice with Et_2O, the combined organic phases were dried over $MgSO_4$, concentrated in vacuo and the residue was purified by silica gel flash chromatography (hexane/EtOAc = 5:1) to give pure products **1** .

(2) General Procedure B： Synthesis of dihydropyran products 2a – 2l

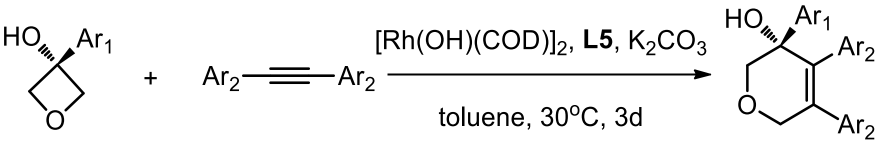

1 **2**

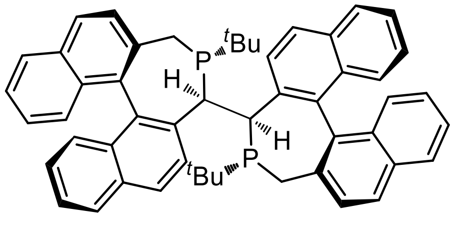

(S)-BINAPINE

L5

To an oven-dried sealed tube equipped with a stirrer bar was added $[Rh(OH)(COD)]_2$ (1.5 mg, 3 umol, 1.5 mol%) , oxetanols **1** (0.20 mmol, 1.0 equiv), alkyne (0.22 mmol, 1.1 equiv) , **L5** [2] (7.3 mg, 10 umol, 5.0 mol%) and Potassium Carbonate (30.4 mg, 0.22 mmol, 1.1 equiv) in glove box. Then dry toluene (1.0ml) was added. After the mixture was stirred at room temperature for 3d , the resulting mixture was removed from the glove box. The resulting mixture was passed through a pad of silica gel and eluted with ethyl acetate. The filtrate was concentrated under reduced pressure. The residue was purified by silica gel flash chromatography (hexane/EtOAc = 5:1) to give pure products **2** .

(3) General Procedure C： Synthesis of dihydropyran products 4a - 4r

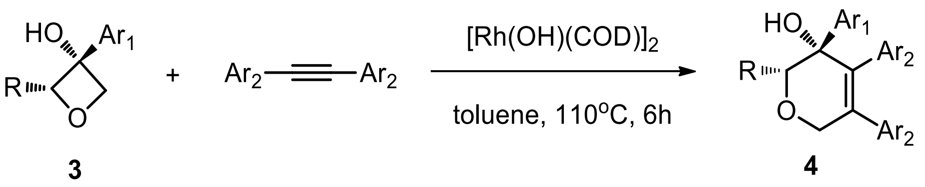

To an oven-dried sealed tube equipped with a stirrer bar was added $[Rh(OH)(COD)]_2$ (2.3 mg, 5 μmol, 2.5 mol%) in glove box. Then, a solution of oxetanols **3** (0.20 mmol, 1.0 equiv) and alkyne (0.22 mmol, 1.1 equiv) in dry toluene (1 mL) was added. The tube was sealed and removed from the glove box. After stirred at 110 °C for 6 h, the reaction mixture was cooled to room temperature. The resulting mixture was passed through a pad of silica gel and eluted with ethyl acetate. The filtrate was concentrated under reduced pressure. The residue was purified by silica gel flash chromatography (hexane/EtOAc = 5:1) to give pure products **4** .

3. Unsuccessful cases under current reaction conditions

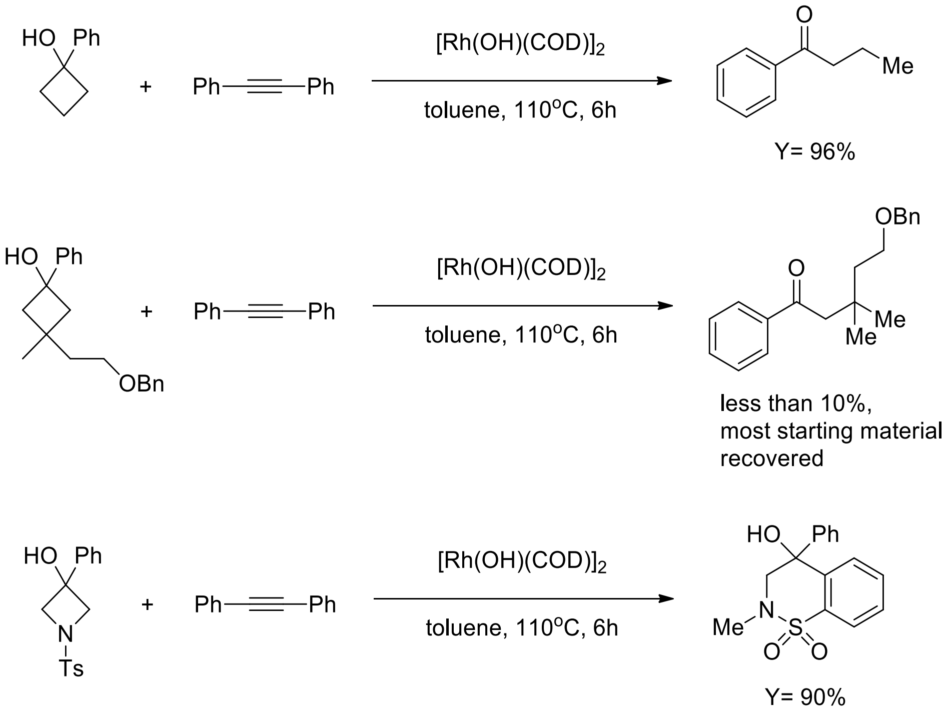

The above experiments suggest the presence of oxygen in the oxetanol facilitate the addition of sp3C-Rh species to the alkyne, the exact reason is not clear currently. We tentatively believe that the oxygen might provide extra stabilization by attracting the electron density from the anionic carbon.

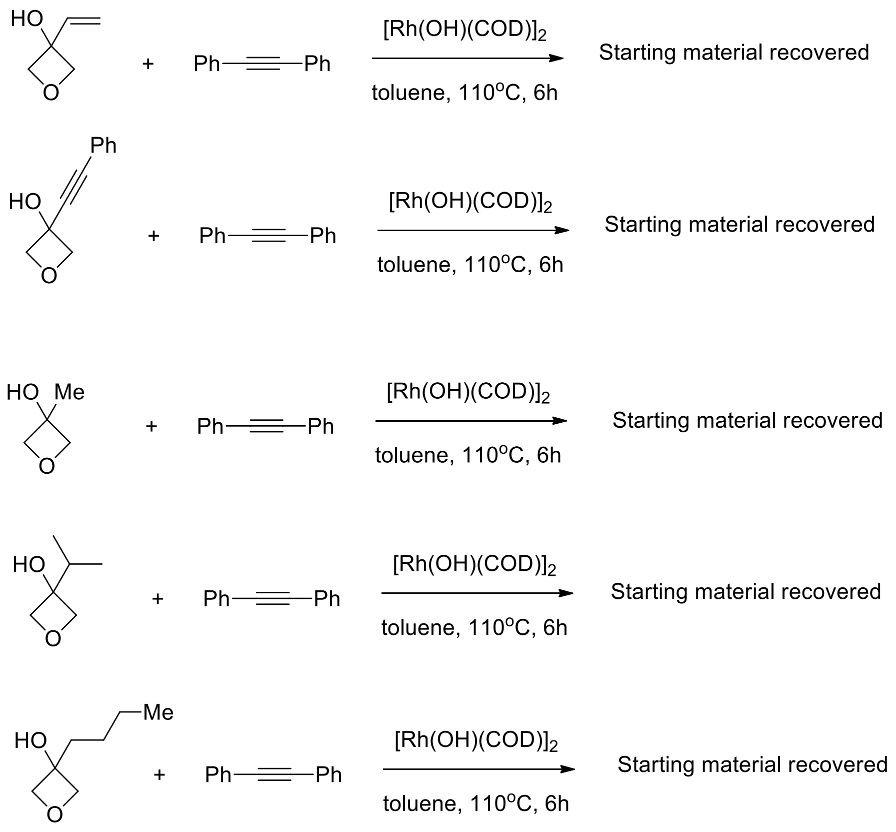

The above experiments suggest the aryl group might provide extra coordination to facilitate the hydroxy metalation followed by β -carbon elimination.

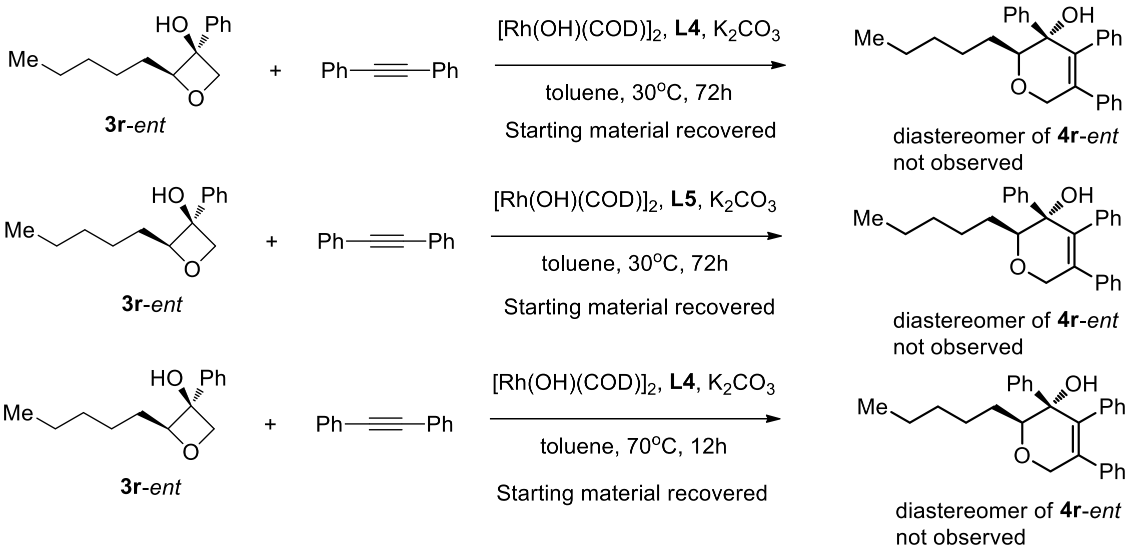

A mismatch of the chiral ligand and the substrate is likely through above experiments. Or it might because the substrate control is override the effect of chiral ligand.

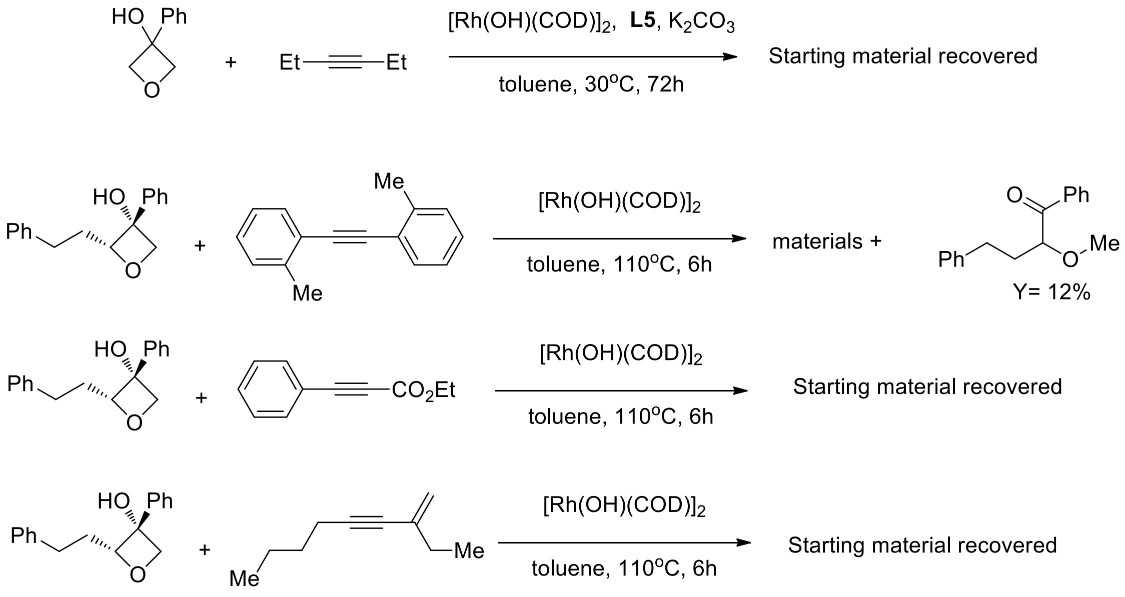

These results suggest the alkyl-rhodium species are quite sensitive to the electron inductivity and steric hindrance.

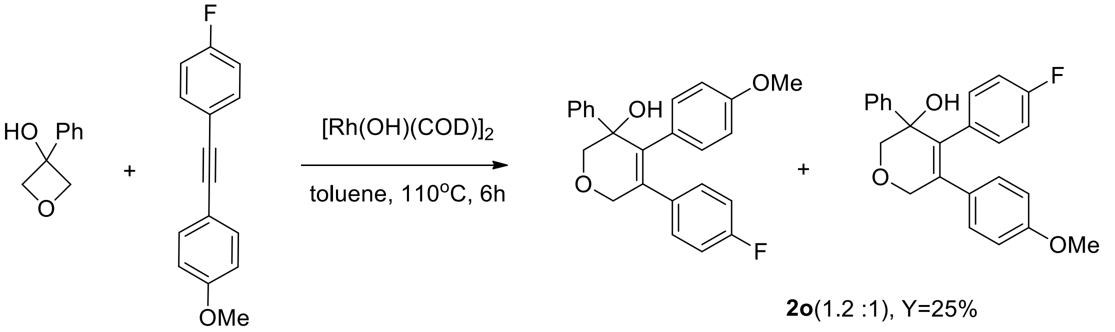

2o(1.2 :1), Y=25%

The reaction of alkyne bearing different substitutions by opposite electronic properties proceeded smoothly, however, giving two inseparable region isomers in roughly 1.2/1 ratio. This result together with **4k** indicates the electronic and steric properties of the alkyne substitution have less effect on the site-selectivity of sp3C-Rh compared with that in Rh(I)-catalyzed cycloaddition of benzocyclobutanol with similar alkynes. (N. Ishida, S. Sawano, Y. Masuda and M. Murakami, J. Am. Chem. Soc., 2012, 134, 17502.)

4. Synthesis and Characterization of Materials

2-phenethyloxetan-3-one (S-2a)

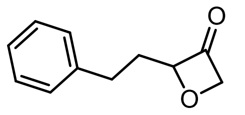

(S-2a)

According to General Procedure A, product **S-2a** (1.58 g, 9.0 mmol, 45% over three steps) was obtained from phenylpropyl aldehyde (2.68 g, 20.0 mmol) as colorless oil with spectral properties identical to the reported in the literature. [1]

^{1}H NMR (400 MHz, $CDCl_3$): δ 7.32 – 7.27 (m, 2H), 7.24 – 7.17 (m, 3H), 5.49 – 5.43 (m, 1H), 5.30 – 5.26 (m, 2H), 2.86 – 2.73 (m, 2H), 2.24 – 2.07 (m, 2H).^{13}C NMR (101 MHz, $CDCl_3$): δ 203.2, 140.3, 128.5, 128.4, 126.3, 102.8, 88.8, 32.8, 30.2.

2-hexyloxetan-3-one (S-2b)

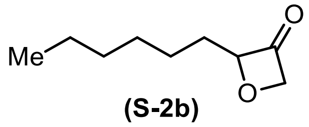

(S-2b)

According to General Procedure A, product **S-2b** (1.53 g, 9.8 mmol, 49% over three steps) was obtained from heptaldehyde (2.28 g, 20.0 mmol) as colorless oil.

^{1}H NMR (400 MHz, $CDCl_3$): δ 5.39 (dd, *J* = 10.8, 6.3 Hz, 1H), 5.23 – 5.11 (m, 2H), 1.81 – 1.72 (m, 2H), 1.45 – 1.33 (m, 2H), 1.27 – 1.19 (m, 6H), 0.81 (t, *J* = 6.5 Hz, 3H).^{13}C NMR (101 MHz, $CDCl_3$): δ 203.2, 103.7, 88.4, 31.4, 31.1, 28.8, 23.9, 22.3, 13.8. IR (neat) cm^{-1} ṽ: 2962, 2860, 1730, 1596, 1413, 1260, 1089, 1018, 865, 797, 701, 663; HRMS (EI(+), 70 eV) : $C_9H_{16}O_2$ $[M]^+$: calcd. 156.1150, found: 156.1156;

2-(4-chlorobutyl)oxetan-3-one (S-2c)

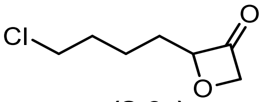

(S-2c)

According to General Procedure A, product **S-2c** (2.00 g, 12.4 mmol, 62% over three steps) was obtained from 5-chloropentanal (2.41 g, 20.0 mmol) as colorless oil.

^{1}H NMR (400 MHz, $CDCl_3$): δ 5.45 (dd, J = 10.4, 6.1 Hz, 1H), 5.28 (dd, J = 15.0, 1.0 Hz, 1H), 5.21 (dd, J = 15.0, 4.3 Hz, 1H), 3.52 (t, J = 6.5 Hz, 2H), 1.89 – 1.76 (m, 4H), 1.68 – 1.52 (m, 2H).^{13}C NMR (101 MHz, $CDCl_3$): δ 202.9, 103.3, 88.8, 44.4, 32.0, 30.3, 21.5. IR (neat) cm^{-1} ṽ: 3463, 2962, 1821, 1721, 1445, 1413, 1259, 1088, 1017, 865, 796, 701, 661; HRMS (EI(+), 70 eV) : $C_7H_{11}ClO_2$ $[M]^+$: calcd. 162.0448, found: 162.6140.

2-(but-3-en-1-yl)oxetan-3-one (S-2d)

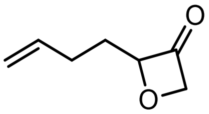

(S-2d)

According to General Procedure A, product **S-2d** (1.22 g, 9.8 mmol, 49% over three steps) was obtained from 4-pentenal (1.68 g, 20.0mmol) as colorless oil.

^{1}H NMR (400 MHz, $CDCl_3$): δ 5.75 (ddt, *J* = 16.9, 10.1, 6.6 Hz, 1H), 5.44 (dd, *J* = 10.8, 6.5 Hz, 1H), 5.28 – 5.15 (m, 2H), 5.00 (dd, *J* = 22.9, 13.6 Hz, 2H), 2.23– 2.14 (m, 2H), 1.94 – 1.83 (m, 2H).^{13}C NMR (101 MHz, $CDCl_3$): δ 203.1, 136.6, 115.7, 102.9, 88.7, 30.2, 28.1. IR (neat) cm^{-1} ṽ: 3431, 3080, 2926, 2856, 2544, 1727, 1642, 1419, 1364, 1197, 1167, 1090, 1069, 996, 915, 783, 655; HRMS (EI(+), 70 eV) : $C_7H_{10}O_2$ $[M]^+$: calcd. 126.0681, found: 126.1531.

2-cyclopropyloxetan-3-one (S-2e)

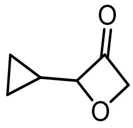

(S-2e)

According to General Procedure A, product **S-2e** (1.00 g, 9.0 mmol, 45% over three steps) was obtained from cyclopropanecarboxaldehyde (1.42 g, 20.0 mmol) as colorless oil.
^{1}H NMR (400 MHz, $CDCl_3$): δ 5.23 – 5.10 (m, 2H), 5.06 – 4.99 (m, 1H), 1.22 – 1.15 (m, 1H), 0.65 – 0.57 (m, 2H), 0.49 – 0.35 (m, 2H).^{13}C NMR (101 MHz, $CDCl_3$): δ 202.0, 106.4, 88.4, 11.1, 1.8, 1.5. IR (neat) cm^{-1} ṽ: 3436, 2962, 2922, 1720, 1640, 1410, 1260, 1088, 1019, 865, 797, 701, 663; HRMS (EI(+), 70 eV) : $C_6H_8O_2$ $[M]^+$: calcd. 112.0524, found: 112.1265.

2-cyclohexyloxetan-3-one (S-2f)

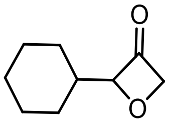

(S-2f)

According to General Procedure A, product **S-2f** (1.72 g, 11.2 mmol, 56% over three steps) was obtained from cyclohexanecarboxaldehyde (2.24 g, 20.0 mmol) as colorless oil with spectral properties identical to the reported in the literature. [1]
^{1}H NMR (300 MHz, $CDCl_3$): δ 5.27 – 5.20 (m, 2H), 5.18 – 5.14 (m, 1H), 1.88 – 1.65 (m, 6H), 1.30 – 1.06 (m, 5H).^{13}C NMR (75 MHz, $CDCl_3$): δ 203.4, 107.6, 88.6, 40.0, 27.4, 27.3, 26.1, 25.4, 25.3.

2-(2-(methylthio)ethyl)oxetan-3-one (S-2g)

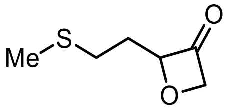

(S-2g)

According to General Procedure A, product **S-2g** (1.26 g, 8.6 mmol, 43% over three steps) was obtained from 3-(Methylthio)propionaldehyde (2.08 g, 20.0 mmol) as colorless oil.
^{1}H NMR (400 MHz, $CDCl_3$): δ 5.47 – 5.42 (m, 1H), 5.15 (dd, *J* = 4.5, 2.7 Hz, 2H), 2.52 – 2.48 (m, 2H), 2.04 – 1.95 (m, 2H), 1.94 (s, 3H).^{13}C NMR (101 MHz, $CDCl_3$): δ 202.3, 101.5, 88.7, 30.1, 28.2, 14.8. IR (neat) cm^{-1} ṽ: 2963, 2905, 1722, 1641, 1412, 1260, 1090, 1018,

865, 798, 701, 662; HRMS (EI(+), 70 eV) : $C_6H_{10}O_2S$[M]$^+$: calcd. 146.0402, found: 146.2074.

2-phenyloxetan-3-one (S-2h)

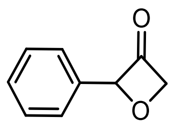

(S-2h)

According to General Procedure A, product **S-2h** (950 mg, 6.4 mmol, 32% over three steps) was obtained from benzaldehyde (2.12 g, 20.0 mmol) as colorless oil with spectral properties identical to the reported in the literature. [1]

^{1}H NMR (400 MHz, $CDCl_3$): δ 7.42 – 7.33 (m, 6H), 6.36 (d, J = 4.0 Hz, 1H), 5.52 – 5.40 (m, 2H).^{13}C NMR (101 MHz, $CDCl_3$): δ 199.2, 130.4, 128.8, 128.7 125.2, 104.2, 90.0.

2-(2-(trifluoromethyl)phenyl)oxetan-3-one (S-2i)

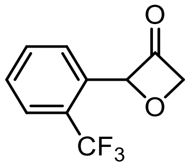

(S-2i)

According to General Procedure A, product **S-2i** (1.56 g, 7.2 mmol, 36% over three steps) was obtained from 2-(Trifluoromethyl)benzaldehyde (3.48 g, 20.0 mmol) as colorless oil.

^{1}H NMR (400 MHz, $CDCl_3$): δ 7.86 (d, J = 7.8 Hz, 1H), 7.70 (d, J = 7.8 Hz, 1H), 7.64 (t, J = 7.7 Hz, 1H), 7.46 (t, J = 7.6 Hz, 1H), 6.81 (s, 1H), 5.59 – 5.46 (m, 2H).^{13}C NMR (101 MHz, $CDCl_3$): δ 196.2, 132.3, 132.0 (q, $J_{C\text{-}F}$ = 1.4 Hz), 128.8, 126.9 (q, $J_{C\text{-}F}$ = 32.3Hz), 126.6, 126.4 (q, $J_{C\text{-}F}$ = 5.0Hz), 123.7 (q, $J_{C\text{-}F}$ = 274.7Hz) , 101.25 (q, $J_{C\text{-}F}$ = 1.9 Hz), 90.2; IR (neat) cm^{-1} ṽ: 2958, 1829, 1733, 1605, 1585, 1493, 1454, 1422, 1312, 1276, 1161, 1109, 1059, 1035, 1001, 960, 906, 826, 768, 654, 615; HRMS (EI(+), 70 eV) : $C_{10}H_7F_3O_2$ [M]$^+$: calcd. 216.0398 , found: 216.0401.

2-pentyloxetan-3-one (S-2r)

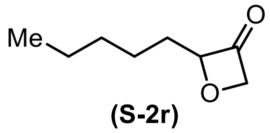

(S-2r)

According to General Procedure A, product **S-2r** (1.50 g, 10.6 mmol, 53% over three steps) was obtained from hexanal (2.00 g, 20.0 mmol) as colorless oil.

^{1}H NMR (400 MHz, $CDCl_3$): δ 5.42 (dd, J = 10.7, 5.8 Hz, 1H), 5.26 – 5.13 (m, 2H), 1.83 – 1.74 (m, 2H), 1.49 – 1.35 (m, 2H), 1.31 – 1.24 (m, 4H), 0.85 (t, J = 6.2 Hz, 3H).^{13}C NMR (101 MHz, $CDCl_3$): δ 203.4, 103.8, 88.5, 31.4, 31.1, 23.7, 22.3, 13.8. IR (neat) cm^{-1} ṽ: 2959, 2930, 2860, 1821, 1727, 1688, 1647, 1463, 1413, 1259, 1088, 1016, 865, 796, 700; HRMS (EI(+), 70 eV) : $C_8H_{14}O_2$ [M]$^+$: calcd. 142.0994, found: 142.1956.

3-phenyloxetan-3-ol (1a)

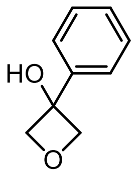

(1a)

According to General Procedure A, product **1a** (3.6 g, 23.9 mmol, 86%) was obtained from **1b** (2.0 g, 27.8 mmol) as colorless oil with spectral properties identical to the reported in the literature. [3]

^{1}H NMR (400 MHz, $CDCl_3$): δ 7.57 (d, *J* = 7.5 Hz, 2H), 7.42 (t, *J* = 7.6 Hz, 2H), 7.33 (t, *J* = 7.3 Hz, 1H), 4.92 – 4.82 (m, 4H), 3.27 (br, 1H).^{13}C NMR (101 MHz, $CDCl_3$): δ 142.2, 128.6, 127.8, 124.4, 85.6, 75.6.

3-(p-tolyl)oxetan-3-ol (1b)

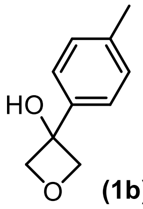

(1b)

According to General Procedure A, product **1b** (412.0 mg, 2.51mmol, 69%) was obtained from 3-Oxetanone (265.0 mg, 3.67 mmol) as colorless oil with spectral properties identical to the reported in the literature. [3]

^{1}H NMR (400 MHz, $CDCl_3$) δ 7.42 (d, *J* = 7.8 Hz, 2H), 7.20 (d, *J* = 7.7 Hz, 2H), 4.85 (q, *J* = 6.7 Hz, 4H), 3.57 (s, 1H), 2.36 (s, 3H).

3-(4-butylphenyl)oxetan-3-ol (1c)

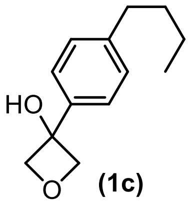

(1c)

According to General Procedure A, product **1c** (426.0 mg, 2.1mmol, 60%) was obtained from 3-Oxetanone (250.0 mg, 3.47 mmol) as colorless oil.

^{1}H NMR (400 MHz, $CDCl_3$) δ 7.47 – 7.43 (m, 2H), 7.21 (d, *J* = 8.0 Hz, 2H), 4.86 (t, *J* = 7.0 Hz, 4H), 3.40 (s, 1H), 2.64 – 2.59 (m, 2H), 1.63 – 1.56 (m, 2H), 1.36 (dd, *J* = 15.0, 7.4 Hz, 2H), 0.93 (t, *J* = 7.3 Hz, 3H). ^{13}C NMR (101 MHz, cdcl$_3$) δ 142.7, 139.6, 128.7, 124.5, 85.7, 77.4, 77.1, 76.7, 75.6, 35.2, 33.6, 22.4, 14.0. IR (neat) cm^{-1} ṽ: 3385, 2928, 2867, 1913, 1654, 1622, 1513, 1456, 1413, 1326, 1283, 1236, 1175, 1128, 1061, 970, 877, 828, 730. HRMS (EI(+), 70 eV) : $C_{13}H_{18}O_2$ [M]+: calcd. 206.1307, found: 206.1310.

3-(4-fluorophenyl)oxetan-3-ol (1d)

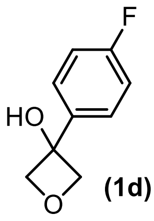

(1d)

According to General Procedure A, product **1d** (346.0 mg, 2.06mmol, 74%) was obtained from 3-Oxetanone (200.0 mg, 2.78 mmol) as colorless oil with spectral properties identical to the reported in the literature. [4]

^{1}H NMR (400 MHz, $CDCl_3$) δ 7.51 (ddd, *J* = 8.3, 5.2, 2.6 Hz, 2H), 7.07 (ddd, *J* = 10.6, 6.0, 2.6 Hz, 2H), 4.90 – 4.81 (m, 4H), 4.06 (s, 1H).

3-(2-fluorophenyl)oxetan-3-ol (1e)

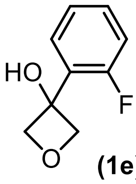

(1e)

According to General Procedure A, product **1e** (314.0 mg, 1.87mmol, 52%) was obtained from 3-Oxetanone (250.0 mg, 3.57 mmol) as colorless oil with spectral properties identical to the reported in the literature. [4]

^{1}H NMR (400 MHz, $CDCl_3$) δ 7.37 – 7.23 (m, 2H), 7.19 – 7.03 (m, 2H), 5.10 (d, *J* = 7.7 Hz, 2H), 4.83 (d, *J* = 7.2 Hz, 2H).

3-(4-butylphenyl)oxetan-3-ol (1f)

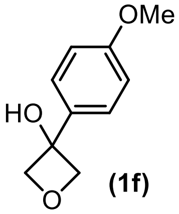

(1f)

According to General Procedure A, product **1f** (150.0 mg, 0.83mmol, 24%) was obtained from 3-Oxetanone (150.0 mg, 3.47 mmol)as colorless oil with spectral properties identical to the reported in the literature. [3]

^{1}H NMR (400 MHz, $CDCl_3$) δ 7.51 – 7.45 (m, 2H), 6.97 – 6.91 (m, 2H), 4.89 (q, *J* = 7.0 Hz, 4H), 3.83 (s, 3H).

3-(3-methoxyphenyl)oxetan-3-ol (1g)

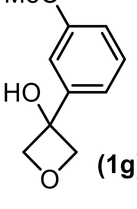

(1g)

According to General Procedure A, product **1g** (400.0 mg, 2.22mmol, 63%) was obtained from 3-Oxetanone (250.0 mg, 3.57 mmol) as colorless oil with spectral properties identical to the reported in the literature. [3]

^{1}H NMR (400 MHz, $CDCl_3$) δ 7.36 – 7.25 (m, 1H), 7.17 – 7.08 (m, 2H), 6.89 – 6.83 (m, 1H), 4.90 – 4.83 (m, 4H), 3.82 (s, 3H).

3-(3,4-dimethoxyphenyl)oxetan-3-ol (1h)

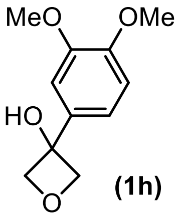

(1h)

According to General Procedure A, product **1h** (412.0 mg, 1.96mmol, 45%) was obtained from 3-Oxetanone (250.0 mg, 3.47 mmol)as colorless oil with spectral properties identical to the reported in the literature. [3]

^{1}H NMR (400 MHz, $CDCl_3$) δ 7.13 – 7.08 (m, 2H), 6.88 (d, *J* = 8.0 Hz, 1H), 4.91 – 4.84 (m, 4H), 3.91 – 3.85 (m, 6H), 3.68 (s, 1H).

3-(3-isopropoxyphenyl)oxetan-3-ol (1i)

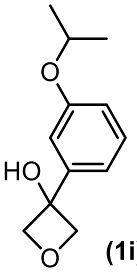

(1i)

According to General Procedure A, product **1i** (316.0 mg, 1.52mmol, 55%) was obtained from 3-Oxetanone (200.0 mg, 2.78 mmol)as colorless oil with spectral properties identical to the reported in the literature. [3]

^{1}H NMR (400 MHz, $CDCl_3$) δ 7.33 – 7.26 (m, 1H), 7.15 – 7.10 (m, 2H), 6.85 (ddd, *J* = 8.2, 2.5, 0.9 Hz, 1H), 4.89 (q, *J* = 7.0 Hz, 4H), 4.59 (dt, *J* = 12.1, 6.1 Hz, 1H), 1.35 (d, *J* = 6.1 Hz, 6H)

2-phenethyl-3-phenyloxetan-3-ol (3a)

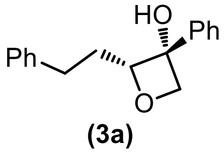

(3a)

According to General Procedure A, product **3a** (682 mg, 2.7 mmol, 82%) was obtained from **S-2a** (580 mg, 3.3 mmol) as white solid, mp 102-105 °C.

^{1}H NMR (400 MHz, $CDCl_3$): δ 7.49 (d, *J* = 7.7 Hz, 2H), 7.38 (t, *J* = 7.6 Hz, 2H), 7.31 (t, *J* = 7.1 Hz, 1H), 7.23 (t, *J* = 7.3 Hz, 2H), 7.15 (dd, *J* = 16.1, 7.2 Hz, 3H), 4.90 (t, *J* = 6.9 Hz, 1H), 4.85 (d, *J* = 7.1 Hz, 1H), 4.65 (d, *J* = 7.1 Hz, 1H), 2.78 – 2.69 (m, 1H), 2.64 – 2.55 (m, 2H),

2.31 – 2.13 (m, 2H).^{13}C NMR (101 MHz, $CDCl_3$): δ 142.8, 141.4, 128.6, 128.41, 128.35, 127.7, 125.9, 124.6, 92.0, 82.5, 76.6, 32.7, 30.6. IR (neat) cm^{-1} ṽ: 3376, 3084, 3027, 2963, 1741, 1601, 1494, 1451, 1406, 1260, 1214, 1156, 1093, 1020, 970, 893, 867, 799, 752, 698; HRMS (EI(+), 70 eV) : $C_{17}H_{18}O_2$ [M-H_2O]$^+$: calcd. 236.1307, found: 236.1199.

2-hexyl-3-phenyloxetan-3-ol (3b)

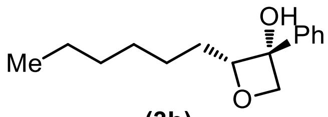

(3b)

According to General Procedure A, product **3b** (140.0 mg, 0.60 mmol, 63%) was obtained from **S-2b** (150.0 mg, 0.96 mmol) as colorless oil.
^{1}H NMR (400 MHz, $CDCl_3$): δ 7.52 (d, *J* = 7.6 Hz, 2H), 7.39 (t, *J* = 7.6 Hz, 2H), 7.31 (t, *J* = 7.3 Hz, 1H), 4.86 (dd, *J* = 7.6, 6.3 Hz, 1H), 4.83 (d, *J* = 7.1 Hz, 1H), 4.59 (d, *J* = 7.0 Hz, 1H), 3.49 (br, 1H), 1.94 – 1.80 (m, 2H), 1.36 – 1.24 (m, 8H), 0.89 (t, *J* = 6.6 Hz, 3H).
^{13}C NMR (101 MHz, $CDCl_3$): δ 143.0, 128.4, 127.6, 124.6, 93.0, 82.3, 76.5, 31.6, 30.8, 29.3, 24.3, 22.5 14.0. IR (neat) cm^{-1} ṽ: 3392, 2959, 2926, 2857, 1450, 1412, 1260, 1172, 1089, 1019, 863, 798, 700; HRMS (EI(+), 70 eV) : $C_{15}H_{22}O_2$ [M]$^+$: calcd. 234.1620, found: 234.3340.

2-(4-chlorobutyl)-3-phenyloxetan-3-ol (3c)

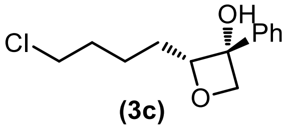

(3c)

According to General Procedure A, product **3c** (250.0 mg, 1.05 mmol, 68%) was obtained from **S-2c** (250.0 mg, 1.54 mmol) as colorless oil.
^{1}H NMR (400 MHz, $CDCl_3$): δ 7.52 (d, *J* = 7.3 Hz, 2H), 7.41 (t, *J* = 7.5 Hz, 2H), 7.33 (t, *J* = 7.3 Hz, 1H), 4.90 – 4.84 (m, 2H), 4.62 (d, *J* = 7.1 Hz, 1H), 3.53 (t, *J* = 6.6 Hz, 2H), 2.83 (br, 1H), 1.96 – 1.78 (m, 4H), 1.58 – 1.41 (m, 2H).^{13}C NMR (101 MHz, $CDCl_3$): δ 142.7, 128.6, 127.8, 124.6, 92.4, 82.4, 76.6, 44.7, 32.4, 30.0, 21.8. IR (neat) cm^{-1} ṽ: 3368, 2949, 2881, 1603, 1495, 1448, 1399, 1309, 1277, 1172, 1129, 1074, 964, 863, 760, 734, 699, 648; HRMS (EI(+), 70 eV) : $C_{13}H_{17}ClO_2$ [M]$^+$: calcd. 240.0917, found: 240.0912.

2-(but-3-en-1-yl)-3-phenyloxetan-3-ol (3d)

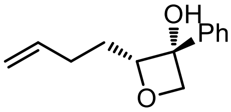

(3d)

According to General Procedure A, product **3d** (160.0 mg, 0.78 mmol, 90%) was obtained from **S-2d** (110.0 mg, 0.87 mmol) as colorless oil.
^{1}H NMR (400 MHz, $CDCl_3$): δ 7.52 (d, *J* = 7.4 Hz, 2H), 7.40 (t, *J* = 7.5 Hz, 2H), 7.31 (t, *J* = 7.3 Hz, 1H), 5.80 (ddt, *J* = 16.8, 10.2, 6.3 Hz, 1H), 5.04 – 4.95 (m, 2H), 4.89 (t, *J* = 6.6 Hz, 1H), 4.83 (d, *J* = 7.0 Hz, 1H), 4.60 (d, *J* = 7.0 Hz, 1H), 3.36 (br, 1H), 2.20 – 1.90 (m, 4H).

^{13}C NMR (101 MHz, $CDCl_3$): δ 142.8, 137.7, 128.5, 127.6, 124.6, 115.1, 92.2, 82.3, 76.4, 30.0, 28.4. IR (neat) cm^{-1} ṽ: 3378, 2921, 2854, 1677, 1640, 1494, 1448, 1377, 1279, 1238, 1173, 1129, 1075, 965, 911, 874, 759, 698; HRMS (EI(+), 70 eV) : $C_{13}H_{16}O_2$ $[M]^+$: calcd. 204.1150, found: 204.1157.

2-cyclopropyl-3-phenyloxetan-3-ol (3e)

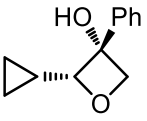

(3e)

According to General Procedure A, product **3e** (160.0 mg, 0.85 mmol, 48%) was obtained from **S-2e** (200.0 mg, 1.78 mmol) as colorless oil.

^1H NMR (400 MHz, $CDCl_3$): δ 7.53 (d, J = 7.3 Hz, 2H), 7.39 (t, J = 7.5 Hz, 2H), 7.31 (t, J = 7.3 Hz, 1H), 4.86 – 4.80 (m, 1H), 4.64 (dd, J = 6.9, 2.6 Hz, 1H), 4.25 (t, J = 7.5 Hz, 1H), 3.34 (br, 1H), 1.48 – 1.38 (m, 1H), 0.77 – 0.68 (m, 1H), 0.67 – 0.59 (m, 1H), 0.53 – 0.44 (m, 1H), 0.26 – 0.19 (m, 1H).^{13}C NMR (101 MHz, $CDCl_3$): δ 142.8, 128.5, 127.6, 124.6, 96.6, 82.4, 76.9, 10.3, 2.3, 1.0; IR (neat) cm^{-1} ṽ: 3407, 2962, 2926, 1676, 1448, 1412, 1260, 1090, 1018, 865, 798, 700; HRMS (EI(+), 70 eV) : $C_{12}H_{14}O_2$ $[M]^+$: calcd. 190.0994, found: 190.0996.

2-cyclohexyl-3-phenyloxetan-3-ol (3f)

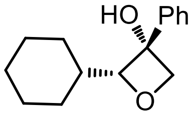

(3f)

According to General Procedure A, product **3f** (680.0 mg, 2.95 mmol, 84%) was obtained from **S-2f** (540.0 mg, 3.51 mmol) as colorless oil.

^1H NMR (400 MHz, $CDCl_3$): δ 7.53 (d, J = 7.2 Hz, 2H), 7.39 (t, J = 7.5 Hz, 2H), 7.30 (t, J = 7.3 Hz, 1H), 4.85 (d, J = 7.3 Hz, 1H), 4.56 – 4.51 (m, 2H), 3.10 (br, 1H), 2.19 – 2.07 (m, 1H), 1.95 (d, J = 12.9 Hz, 1H), 1.81 – 1.73 (m, 1H), 1.72 – 1.61 (m, 3H), 1.36 – 1.24 (m, 2H), 1.22 – 1.12 (m, 1H), 0.89 (ddd, J = 24.2, 12.5, 3.5 Hz, 1H), 0.82 – 0.71 (m, 1H).^{13}C NMR (101 MHz, $CDCl_3$): δ 142.9, 128.5, 127.5, 124.8, 95.9, 82.2, 77.2, 38.7, 27.8, 27.5, 26.5, 25.4, 25.2. IR (neat) cm^{-1} ṽ: 3373, 2923, 2850, 1952, 1878, 1813, 1711, 1600, 1496, 1448, 1398, 1353, 1261, 1166, 1074, 1025, 969, 914, 881, 856, 801, 756, 698; HRMS (EI(+), 70 eV) : $C_{15}H_{20}O_2$ $[M]^+$: calcd. 232.1463, found: 232.1459.

2-(2-(methylthio)ethyl)-3-phenyloxetan-3-ol (3g)

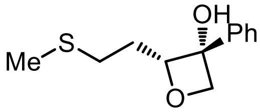

(3g)

According to General Procedure A, product **3g** (210.0 mg, 0.94 mmol, 81%) was obtained from **S-2g** (170.0 mg, 1.16 mmol) as colorless oil.

^1H NMR (400 MHz, $CDCl_3$): δ 7.51 (d, *J* = 7.5 Hz, 2H), 7.38 (t, *J* = 7.6 Hz, 2H), 7.29 (t, *J* = 7.3 Hz, 1H), 4.93 (t, *J* = 6.8 Hz, 1H), 4.77 (d, *J* = 6.9 Hz, 1H), 4.61 (d, *J* = 6.9 Hz, 1H), 3.80 (br, 1H), 2.59 – 2.51 (m, 1H), 2.46 – 2.38 (m, 1H), 2.21 – 2.14 (m, 2H), 2.04 (s, 3H).
^{13}C NMR (101 MHz, $CDCl_3$): δ 142.8, 128.4, 127.6, 124.5, 91.5, 82.3, 75.9, 30.4, 28.9, 15.4. IR (neat) cm^{-1} ṽ: 3375, 3060, 2953, 2914, 2881, 1956, 1884, 1813, 1603, 1495, 1446, 1312, 1279, 1171, 1127, 1025, 959, 866, 808, 763, 698; HRMS (EI(+), 70 eV) : $C_{12}H_{16}O_2S$ $[M]^+$: calcd. 224.0871, found: 224.0875.

2,3-diphenyloxetan-3-ol (3h)

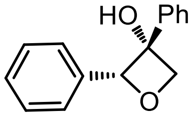

(3h)

According to General Procedure A, product **3h** (263.0 mg,1.16 mmol, 64%) was obtained from **S-2h** (270.0 mg, 1.82 mmol) as white solid, mp 114-117 °C.
^1H NMR (400 MHz, $CDCl_3$) δ 7.65 (d, *J* = 7.3 Hz, 2H), 7.46 – 7.33 (m, 8H), 5.89 (s, 1H), 5.04 (d, *J* = 7.1 Hz, 1H), 4.76 (d, *J* = 7.1 Hz, 1H), 2.31 (s, 1H). ^{13}C NMR (101 MHz, $CDCl_3$): δ 142.5, 136.2, 128.7, 128.58, 128.56, 127.8, 126.2, 124.6, 94.0, 84.0, 76.9. IR (neat) cm^{-1} ṽ: 3400, 2962, 2924, 1494, 1449, 1412, 1260, 1089, 1018, 867, 797, 698; HRMS (EI(+), 70 eV) : $C_{15}H_{14}O_2$ $[M]^+$: calcd. 226.0994, found: 226.1002.

3-phenyl-2-(2-(trifluoromethyl)phenyl)oxetan-3-ol (3i)

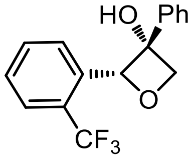

(3i)

According to General Procedure A, product **3i** (140.0 mg, 0.48 mmol, 74%) was obtained from **S-2i** (140.0 mg, 0.65 mmol) as white solid, mp 116-118 °C.
^1H NMR (400 MHz, $CDCl_3$) δ 8.08 (d, *J* = 7.7 Hz, 1H), 7.68 (d, *J* = 7.3 Hz, 3H), 7.61 (d, *J* = 7.7 Hz, 1H), 7.41 (t, *J* = 7.5 Hz, 3H), 7.36 – 7.30 (m, 1H), 6.20 (s, 1H), 4.86 (d, *J* = 6.9 Hz, 1H), 4.82 (d, *J* = 7.0 Hz, 1H), 2.37 (s, 1H). ^{13}C NMR (101 MHz, cdcl$_3$) δ 142.4, 135.2, 132.3, 128.4, 128.2, 127.9, 127.7, 127.2 (q, $J_{C\text{-}F}$ = 31.2 Hz), 125.8 (q, $J_{C\text{-}F}$ = 2.7Hz), 124.0, 123.6 (q, $J_{C\text{-}F}$ = 275.1Hz), 91.5, 85.1, 77.05. IR (neat) cm^{-1} ṽ: 3394, 3064, 2959, 2926, 2855, 1709, 1659, 1595, 1498, 1473, 1452, 1386, 1314, 1260, 1164, 1112, 1036, 886, 798, 756, 696, 664; HRMS (EI(+), 70 eV) : $C_{16}H_{13}F_3O_2$ $[M]^+$: calcd. 294.0868, found: 294.0876.

3-(naphthalen-1-yl)-2-phenethyloxetan-3-ol (3n)

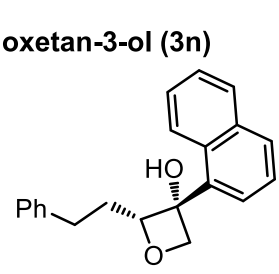

(3n)

According to General Procedure A, product **3n** (80.0 mg, 0.26 mmol, 46%) was obtained from **S-2a** (100.0 mg, 0.57 mmol) as white solid, mp 122-125 °C.
^{1}H NMR (400 MHz, $CDCl_3$): δ 7.83 – 7.79 (m, 1H), 7.73 (d, *J* = 8.2 Hz, 1H), 7.68 – 7.63 (m, 1H), 7.47 – 7.40 (m, 2H), 7.35 – 7.14 (m, 7H), 5.28 (dd, *J* = 8.7, 4.8 Hz, 1H), 4.87 (s, 2H), 2.89 (br, 1H), 2.86 – 2.77 (m, 1H), 2.67 – 2.58 (m, 1H), 2.45 – 2.34 (m, 1H), 2.24 – 2.14 (m, 1H).^{13}C NMR (101 MHz, $CDCl_3$): δ 141.5, 138.0, 134.3, 130.3, 129.2, 129.0, 128.4, 126.4, 125.93, 125.91, 124.8, 124.6, 123.7, 88.9, 81.5, 77.7, 32.6, 30.6. IR (neat) cm^{-1} ṽ: 3397, 2961, 2918, 2850, 1728, 1598, 1538, 1496, 1462, 1380, 1260, 1094, 1020, 975, 898, 871, 799, 744, 696, 663, 626, 612; HRMS (EI(+), 70 eV) : $C_{21}H_{20}O_2$ $[M]^+$: calcd. 304.1463, found: 304.1459.

3-(4-methoxyphenyl)-2-phenethyloxetan-3-ol (3o)

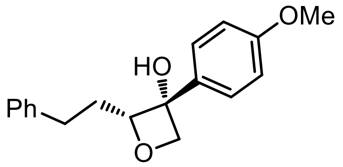

(3o)

According to General Procedure A, product **3o** (150.0 mg, 0.53 mmol, 62%) was obtained from **S-2a** (150.0 mg, 0.85 mmol) as white solid, mp 109-111 °C.
^{1}H NMR (400 MHz, $CDCl_3$): δ 7.36 (d, *J* = 8.5 Hz, 2H), 7.22 (t, *J* = 7.2 Hz, 2H), 7.15 (d, *J* = 7.0 Hz, 1H), 7.11 (d, *J* = 7.4 Hz, 2H), 6.87 (d, *J* = 8.5 Hz, 2H), 4.85 (t, *J* = 6.8 Hz, 1H), 4.78 (d, *J* = 7.0 Hz, 1H), 4.58 (d, *J* = 7.0 Hz, 1H), 3.76 (s, 3H), 3.10 (br, 1H), 2.74 – 2.63 (m, 1H), 2.62 – 2.52 (m, 1H), 2.26 – 2.08 (m, 2H).^{13}C NMR (101 MHz, $CDCl_3$): δ 158.9, 141.4, 135.1, 128.30, 128.27, 125.9, 125.8, 113.8, 92.1, 82.4, 76.2, 55.2, 32.5, 30.5. IR (neat) cm^{-1} ṽ: 3364, 3060, 3026, 3008, 2921, 2852, 1884, 1727, 1611, 1580, 1515, 1491, 1456, 1384, 1299, 1248, 1177, 1118, 1027, 981, 952, 901, 879, 857, 828, 796, 751, 730, 700, 642, 616; HRMS (EI(+), 70 eV) : $C_{18}H_{20}O_3$ $[M]^+$: calcd. 284.1412, found: 284.1408.

2-phenethyl-3-(o-tolyl)oxetan-3-ol (3p)

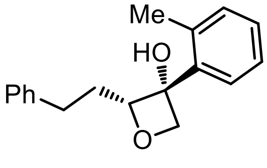

(3p)

According to General Procedure A, product **3p** (120.0 mg, 0.45 mmol, 79%) was obtained from **S-2a** (100.0 mg, 0.57 mmol) as white solid, mp 104-107 °C.
^{1}H NMR (400 MHz, $CDCl_3$): δ 7.26 (t, *J* = 7.4 Hz, 2H), 7.22 – 7.12 (m, 4H), 7.11 – 7.04 (m, 2H), 6.91 (d, *J* = 7.4 Hz, 1H), 5.15 (dd, *J* = 9.0, 4.3 Hz, 1H), 4.74 (d, *J* = 7.1 Hz, 1H), 4.54 (d, *J* = 7.0 Hz, 1H), 2.97 (br, 1H), 2.84 – 2.72 (m, 1H), 2.64 – 2.53 (m, 1H), 2.37 – 2.24 (m, 1H), 2.13 (s, 3H), 2.10 – 2.00 (m, 1H).^{13}C NMR (101 MHz, $CDCl_3$): δ 141.5, 140.0, 136.5, 131.5, 128.32, 128.30, 128.2, 125.8, 125.7, 88.5, 80.7, 77.9, 32.5, 30.5, 19.2. IR (neat) cm^{-1} ṽ:

3309, 3083, 3064, 3023, 3001, 2953, 2925, 2895, 2855, 1812, 1602, 1491, 1454, 1398, 1315, 1283, 1254, 1235, 1178, 1122, 1062, 1031, 972, 957, 901, 824, 758, 730, 700, 667, 645; HRMS (EI(+), 70 eV) : $C_{18}H_{20}O_2$ $[M]^+$: calcd. 268.1463, found: 268.1458.

3-(4-fluorophenyl)-2-phenethyloxetan-3-ol (3q)

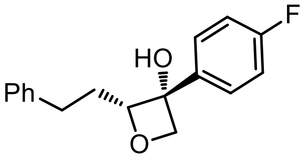

(3q)

According to General Procedure A, product **3q** (120.0 mg, 0.44 mmol, 52%) was obtained from **S-2a** (150.0 mg, 0.85 mmol) as white solid, mp 102-105 °C.
^{1}H NMR (400 MHz, $CDCl_3$) δ 7.46 (d, J = 5.3 Hz, 1H), 7.44 (d, J = 5.3 Hz, 1H), 7.25 – 7.20 (m, 2H), 7.19 – 7.14 (m, 1H), 7.11 (d, J = 7.2 Hz, 2H), 7.05 (t, J = 8.6 Hz, 2H), 4.85 (t, J = 6.9 Hz, 1H), 4.79 (d, J = 7.1 Hz, 1H), 4.61 (d, J = 7.1 Hz, 1H), 2.86 (br, 1H), 2.75 – 2.66 (m, 1H), 2.62 – 2.53 (m, 1H), 2.26 – 2.12 (m, 2H).^{13}C NMR (101 MHz, cdcl$_3$) δ 162.1 (d, $J_{C\text{-}F}$ = 247.8 Hz), 141.2, 138.59 (d, $J_{C\text{-}F}$ = 3.2 Hz), 128.4, 128.3, 126.40 (d, $J_{C\text{-}F}$ = 8.1 Hz), 126.0, 115.3 (d, $J_{C\text{-}F}$ = 21.5 Hz), 92.3, 82.6, 76.2, 32.6, 30.5. IR (neat) cm^{-1} ṽ: 3360, 3027, 2961, 2921, 1603, 1482, 1454, 1408, 1260, 1226, 1176, 1097, 1020, 970, 876, 799, 750, 699, 623; HRMS (EI(+), 70 eV) : $C_{17}H_{17}FO_2$ $[M]^+$: calcd. 272.1213, found: 272.1220.

2-pentyl-3-phenyloxetan-3-ol (3r)

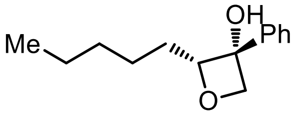

(3r)

According to General Procedure A, product **3r** (98.0 mg, 044 mmol, 79%) was obtained from **S-2r** (80.0 mg, 0.56 mmol) as colorless oil.
^{1}H NMR (400 MHz, $CDCl_3$): δ 7.52 (d, J = 7.4 Hz, 2H), 7.39 (t, J = 7.5 Hz, 2H), 7.31 (t, J = 7.3 Hz, 1H), 4.90 – 4.79 (m, 2H), 4.59 (d, J = 7.0 Hz, 1H), 3.38 (br, 1H), 1.95 – 1.79 (m, 2H), 1.38 – 1.24 (m, 6H), 0.89 (t, J = 6.2 Hz, 3H).^{13}C NMR (101 MHz, $CDCl_3$): δ 143.0, 128.5, 127.6, 124.6, 93.0, 82.3, 76.5, 31.8, 30.7, 24.0, 22.4, 13.9. IR (neat) cm^{-1} ṽ: 3382, 3062, 3031, 2957, 2929, 2858, 1603, 1495, 1450, 1409, 1379, 1260, 1173, 1086, 1020, 969, 884, 798, 700; HRMS (EI(+), 70 eV) : $C_{14}H_{20}O_2$ $[M]^+$: calcd. 220.1463, found: 220.1457.

5. Synthesis and Characterization of Products

(R)-3,4,5-triphenyl-3,6-dihydro-2H-pyran-3-ol (2a)

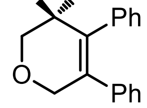

(2a)

According to General Procedure B, product **2a** (85.0 mg, 0.26 mmol, 70%) was obtained from **1a** (55.5 mg, 0.37 mmol) and diphenylacetylene (7.5mg, 0.41mmol) as yellow oil.

^{1}H NMR (400 MHz, $CDCl_3$) δ 7.58 – 7.52 (m, 2H), 7.31 (t, *J* = 7.6 Hz, 2H), 7.26 – 7.21 (m, 1H), 7.20 – 7.13 (m, 3H), 7.07 (d, *J* = 2.1 Hz, 1H), 7.05 (d, *J* = 1.6 Hz, 1H), 6.98 – 6.93 (m, 3H), 6.93 – 6.88 (m, 2H), 4.68 (d, *J* = 16.5 Hz, 1H), 4.61 (d, *J* = 16.5 Hz, 1H), 4.08 – 3.99 (m, 2H), 2.87 (br, 1H).^{13}C NMR (101 MHz, $CDCl_3$): δ 142.3, 137.9, 137.5, 137.0, 136.4, 130.5, 128.4,128.0, 127.9, 127.3, 127.04, 126.98, 126.5, 126.3, 77.2, 72.6, 69.9. IR (neat) cm^{-1} ṽ: 2963, 2905, 1412, 1260, 1089, 1018, 865, 797, 701, 663; HRMS (EI(+), 70 eV) : $C_{23}H_{20}O_2$ $[M]^+$: calcd. 328.1463, found: 328.1464. $[\alpha]_D^{20}$ = -10.5 (c =0.33, CH_2Cl_2); HPLC (Chiralcel OD-H column, hexanes:i-PrOH = 90:10, 1.0 mL/min, 210 nm), t_{minor} = 5.4min, t_{major} =6.1 min, 93% ee.

(R)-4,5-diphenyl-3-(p-tolyl)-3,6-dihydro-2H-pyran-3-ol (2b)

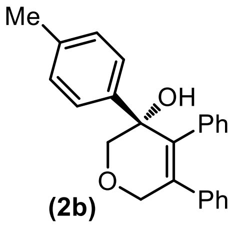

(2b)

According to General Procedure B, product **2b** (53.7 mg, 0.15 mmol, 72%) was obtained from **1b** (37.8 mg, 0.21 mmol) and diphenylacetylene (41.0 mg, 0.23mmol) as yellow oil.

^{1}H NMR (400 MHz, $CDCl_3$) δ 7.40 (d, *J* = 8.2 Hz, 2H), 7.16 – 7.07 (m, 5H), 7.02 (dd, *J* = 7.7, 1.8 Hz, 2H), 6.97 – 6.83 (m, 5H), 4.65 – 4.55 (m, 2H), 4.01 – 3.93 (m, 2H), 2.29 (s, 3H). ^{13}C NMR (101 MHz, $cdcl_3$) δ 139.4, 138.0, 137.5, 137.0, 136.54, 136.5, 130.5, 128.6, 128.5, 128.0, 127.3, 127.0, 126.5, 126.2, 77.2, 72.5, 69.9, 21.0. IR (neat) cm^{-1} ṽ: 2963.64, 2909.80, 1408.78, 1260.39, 1087.61, 1021.43, 799.70, 695.00. HRMS (EI(+), 70 eV) : $C_{24}H_{22}O_2$ [M]+: calcd. 342.1620, found: 342.1310. $[\alpha]_D^{20}$ = -19.4 (c = 0.49, CH_2Cl_2); HPLC (Chiralcel IE-H column, hexanes:i-PrOH = 98:2, 1.0 mL/min, 210 nm), t_{minor} = 8.6 min, t_{major} = 9.9 min, 93% ee.

(R)-3-(4-butylphenyl)-4,5-diphenyl-3,6-dihydro-2H-pyran-3-ol (2c)

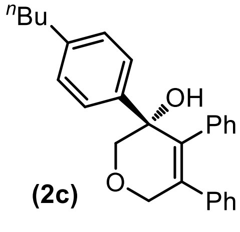

(2c)

According to General Procedure B, product **2c** (42.3 mg, 0.11 mmol, 61%) was obtained from **1c** (37.1 mg, 0.18 mmol) and diphenylacetylene (35.3 mg, 0.20mmol) as white solid, mp 131-134 ^oC.

^{1}H NMR (400 MHz, $CDCl_3$) δ 7.41 (d, *J* = 8.1 Hz, 2H), 7.11 (dd, *J* = 13.6, 7.5 Hz, 5H), 7.03 (d, *J* = 7.6 Hz, 2H), 6.97 – 6.89 (m, 3H), 6.85 (dd, *J* = 7.1, 1.1 Hz, 2H), 4.60 (s, 2H), 4.03 – 3.95 (m, 2H), 2.67 (s, 1H), 2.59 – 2.53 (m, 2H), 1.60 – 1.53 (m, 2H), 1.32 (dd, *J* = 14.8, 7.4 Hz, 2H), 0.90 (t, *J* = 7.3 Hz, 3H). ^{13}C NMR (101 MHz, $cdcl_3$) δ 141.6 , 139.7 , 138.0 , 137.7,

136.9 , 136.5, 130.6 , 128.5, 128.0, 127.97 127.3, 127.0, 126.5, 126.2, 77.1, 72.5, 70.0, 35.2 , 33.5, 22.4, 14.0. IR (neat) cm^{-1} ṽ: 3384, 3058, 2927, 2862, 1709, 1605, 1500, 1451, 1260, 1172, 1042, 1001, 828, 761, 698, 641. HRMS (EI(+), 70 eV) : $C_{27}H_{29}O_2$ [M]+: calcd. 384.2089, found: 384.2080. $[\alpha]_D^{20}$ = -31.2 (c = 0.23, CH_2Cl_2); HPLC (Chiralcel OD-H column, hexanes:i-PrOH = 98:2, 1.0 mL/min, 210 nm), t_{minor} = 7.1 min, t_{major} =8.3 min, 91% ee.

(R)-3-(4-fluorophenyl)-4,5-diphenyl-3,6-dihydro-2H-pyran-3-ol (2d)

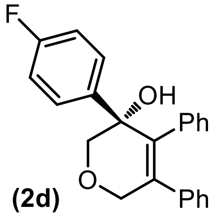

(2d)

According to General Procedure B, product **2d** (55.4 mg, 0.16 mmol, 80%) was obtained from **1d** (33.6 mg, 0.20 mmol) and diphenylacetylene (39.2 mg, 0.22mmol) as yellow oil. ^{1}H NMR (400 MHz, $CDCl_3$) δ 7.50 – 7.42 (m, 2H), 7.17 – 7.08 (m, 3H), 7.04 – 6.98 (m, 2H), 6.98 – 6.89 (m, 5H), 6.88 – 6.81 (m, 2H), 4.68 – 4.51 (m, 2H), 4.01 – 3.91 (m, 2H), 2.90 (s, 1H). ^{13}C NMR (101 MHz, $cdcl_3$) δ 161.8 (d, $J_{C\text{-}F}$ = 246.4 Hz), 138.2(d, $J_{C\text{-}F}$ = 3.0 Hz), 137.8, 137.3, 137.2, 136.3, 130.4, 128.4, 128.02 (d, $J_{C\text{-}F}$ = 3.5 Hz), 128.0, 127.98, 127.4 127.1, 126.6, 114.7(d, $J_{C\text{-}F}$ = 21.2 Hz), , 77.2, 72.3, 69.9. IR (neat) cm^{-1} ṽ: 3434, 3059, 2962, 2852, 1731, 1601, 1502, 1446, 1373, 1228, 1118, 1082, 1022, 961, 915, 829, 805, 757, 697. HRMS (EI(+), 70 eV) :$C_{23}H_{19}FO_2$ [M]+: calcd. 346.1369, found: 346.1371. $[\alpha]_D^{20}$ = 3.8050 (c = 0.50, CH_2Cl_2); HPLC (Chiralcel OD-H column, hexanes:i-PrOH = 95:%, 1.0 mL/min, 210 nm), t_{minor} = 12.2 min, t_{major} = 13.0 min, 90% ee.

(S)-3-(2-fluorophenyl)-4,5-diphenyl-3,6-dihydro-2H-pyran-3-ol (2e)

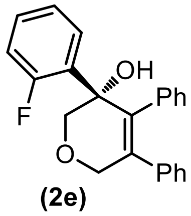

(2e)

According to General Procedure B, product **2e** (63.3 mg, 0.19 mmol, 78%) was obtained from **1e** (40.3 mg, 0.24 mmol) and diphenylacetylene (47.0 mg, 0.26 mmol) as yellow oil. ^{1}H NMR (400 MHz, $CDCl_3$) δ 7.69 (t, J = 7.9 Hz, 1H), 7.16 – 7.07 (m, 4H), 7.00 (dd, J = 5.4, 2.4 Hz, 5H), 6.92 – 6.77 (m, 4H), 4.73 (d, J = 16.2 Hz, 1H), 4.46 (d, J = 16.3 Hz, 1H), 4.27 (d, J = 11.4 Hz, 1H), 3.94 (d, J = 11.4 Hz, 1H), 3.33 (s, 1H). ^{13}C NMR (101 MHz, $cdcl_3$) δ 159.7(d, $J_{C\text{-}F}$ = 246.4 Hz), 138.1, 136.9, 136.1, 130.0, 129.1(d, $J_{C\text{-}F}$ = 8.1Hz), 128.8, 128.4, 128.2 ,128.1, 128.0, 127.02, 127.0, 126.3, 123.9(d, $J_{C\text{-}F}$ = 4.0 Hz), 115.2 (d, $J_{C\text{-}F}$ = 22.2Hz), 74.8, 71.4, 69.8. IR (neat) cm^{-1} ṽ: 2964, 2910, 1409, 1261, 1091, 1023, 801, 694.92. HRMS (EI(+), 70 eV) : $C_{23}H_{19}FO_2$ [M]+: calcd. 346.1369, found: 346.1374. $[\alpha]_D^{20}$ = 48.9070 (c = 0.16, CH_2Cl_2); HPLC (Chiralcel OD-H column, hexanes:i-PrOH = 98:2, 1.0 mL/min, 210 nm), t_{minor} = 7.8 min, t_{major} = 9.3 min, 92% ee.

(R)-3-(4-methoxyphenyl)-4,5-diphenyl-3,6-dihydro-2H-pyran-3-ol (2f)

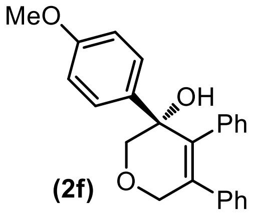

(2f)

According to General Procedure B, product **2f** (53.7 mg, 0.15 mmol, 72%) was obtained from **1f** (37.8 mg, 0.21 mmol) and diphenylacetylene (41.2 mg, 0.23mmol) as yellow oil. ^{1}H NMR (400 MHz, $CDCl_3$) δ 7.46 – 7.39 (m, 2H), 7.18 – 7.09 (m, 3H), 7.02 (dd, *J* = 7.6, 1.8 Hz, 2H), 6.97 – 6.90 (m, 3H), 6.90 – 6.84 (m, 2H), 6.84 – 6.79 (m, 2H), 4.64 – 4.54 (m, 2H), 4.01 – 3.90 (m, 2H), 3.75 (s, 3H). ^{13}C NMR (101 MHz, cdcl$_3$) δ 158.5, 137.9, 137.6, 136.9, 136.5, 134.6, 130.5 , 128.5, 128.0, 127.4, 127.36, 127.0, 126.5, 113.3, 77.2, 72.3, 69.9, 55.1. IR (neat) cm^{-1} ṽ: 2964, 2913, 1409, 1261, 1090, 1023, 801, 695. HRMS (EI(+), 70 eV) : $C_{25}H_{24}O_4$ [M]+: calcd. 358.1569, found: 358.1582. $[\alpha]_D^{20}$ = 3.2131 (c = 0.44, CH_2Cl_2); HPLC (Chiralcel OD-H column, hexanes:i-PrOH = 98:2, 1.0 mL/min, 210 nm), t_{minor} = 14.7 min, t_{major} = 15.2 min, 92% ee.

(R)-3-(3-methoxyphenyl)-4,5-diphenyl-3,6-dihydro-2H-pyran-3-ol (2g)

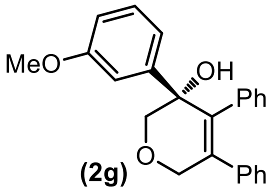

(2g)

According to General Procedure B, product **2g** (50.1 mg, 0.14 mmol, 74%) was obtained from **1g** (34.2 mg, 0.19 mmol) and diphenylacetylene (37.2 mg, 0.21 mmol) as yellow oil. ^{1}H NMR (400 MHz, $CDCl_3$) δ 7.20 (t, *J* = 8.0 Hz, 1H), 7.16 – 7.07 (m, 5H), 7.04 – 6.99 (m, 2H), 6.95 – 6.87 (m, 5H), 6.76 – 6.71 (m, 1H), 4.65 – 4.54 (m, 2H), 4.02 – 3.96 (m, 2H), 3.74 (s, 3H), 2.82 (s, 1H). ^{13}C NMR (101 MHz, cdcl$_3$) δ 159.3, 144.2, 137.9, 137.3, 137.0, 136.4, 130.5, 128.8, 128.4, 128.0, 127.4, 127.0 , 126.6, 118.8, 112.3, 112.0, 77.1, 72.6, 69.9, 55.1. IR (neat) cm^{-1} ṽ: 2964, 2908, 1409, 1261, 1091, 1023, 801, 695.52. HRMS (EI(+), 70 eV) : C24H22O3 [M]+: calcd. 358.1569, found: 358.1563. $[\alpha]_D^{20}$ = -11.6 (c = 0.50, CH_2Cl_2); HPLC (Chiralcel OD-H column, hexanes:i-PrOH = 98:2, 0.8 mL/min, 210 nm), t_{minor} = 14.9min, t_{major} =16.0 min, 92% ee.

(R)-3-(3,4-dimethoxyphenyl)-4,5-diphenyl-3,6-dihydro-2H-pyran-3-ol (2h)

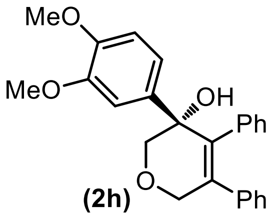

(2h)

According to General Procedure B, product **2h** (46.6 mg, 0.12 mmol, 69%) was obtained

from **1h** (36.7 mg, 0.17 mmol) and diphenylacetylene (33.3 mg, 0.19 mmol) as white solid, mp 129-131 °C.

^{1}H NMR (400 MHz, $CDCl_3$) δ 7.18 – 7.10 (m, 3H), 7.09 – 6.99 (m, 4H), 6.98 – 6.91 (m, 3H), 6.88 (ddd, *J* = 4.2, 3.7, 2.4 Hz, 2H), 6.80 (d, *J* = 8.6 Hz, 1H), 4.65 – 4.55 (m, 2H), 3.98 (d, *J* = 1.8 Hz, 2H), 3.84 (s, 3H), 3.81 (s, 3H), 2.77 (s, 1H). ^{13}C NMR (101 MHz, $cdcl_3$) δ 148.4, 147.8, 137.9, 137.6, 136.9, 136.4, 135.1, 130.5, 128.4, 128.0, 127.4, 127.0, 126.6, 118.8, 110.4, 109.7, 77.05, 72.3, 69.8, 55.74. 55.7. IR (neat) cm^{-1} ṽ:2964, 2910, 1410, 1261, 1091, 1023, 866, 802, 695. HRMS (EI(+), 70 eV) : $C_{25}H_{22}O_3$ [M]+: calcd. 388.1675, found: 388.1667. $[\alpha]_D^{20}$ = -27.9 (c = 0.44, CH_2Cl_2); HPLC (Chiralcel IE-H column, hexanes:i-PrOH = 99:1, 0.6 mL/min, 210 nm), t_{minor} = 14.8 min, t_{major} = 15.8 min, 91% ee.

(R)-3-(3-isopropoxyphenyl)-4,5-diphenyl-3,6-dihydro-2H-pyran-3-ol (2i)

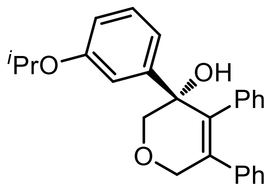

(2i)

According to General Procedure B, product **2i** (50.2 mg, 0.13 mmol, 76%) was obtained from **1i** (35.4 mg, 0.17 mmol) and diphenylacetylene (41.0 mg, 0.23mmol) as white solid, mp 134-136 °C.

^{1}H NMR (400 MHz, $CDCl_3$) δ 7.16 (ddd, *J* = 8.6, 7.8, 3.9 Hz, 4H), 7.08 (d, *J* = 7.3 Hz, 2H), 7.02 (dd, *J* = 7.4, 1.9 Hz, 2H), 6.95 – 6.87 (m, 5H), 6.75 – 6.71 (m, 1H), 4.65 – 4.55 (m, 2H), 4.50 (dq, *J* = 6.1, 3.9 Hz, 1H), 4.00 (d, *J* = 2.2 Hz, 2H), 2.77 (s, 1H), 1.29 – 1.24 (m, 6H). ^{13}C NMR (101 MHz, $cdcl_3$) δ 157.6, 144.0, 137.9, 137.4, 136.9, 136.4, 130.5, 128.8, 128.4, 128.0, 127.3, 127.0, 126.5, 118.7, 114.7, 114.3, 77.1, 72.5, 69.9, 69.8, 22.01, 22.0. IR (neat) cm^{-1} ṽ: 3438, 3071, 2965, 2917, 2853, 1582, 1446, 1261, 1097, 1025, 805, 701. HRMS (EI(+), 70 eV) : $C_{26}H_{26}O_3$.[M]+: calcd. 386.1882, found: 386.1890. $[\alpha]_D^{20}$ = 143.1 (c = 0.30, CH_2Cl_2); HPLC (Chiralcel OD-H column, hexanes:i-PrOH = 90:10, 1.0 mL/min, 210 nm), t_{minor} = 8.2min, t_{major} =5.8 min, 94% ee.

(R)-3-phenyl-4,5-di-p-tolyl-3,6-dihydro-2H-pyran-3-ol (2j)

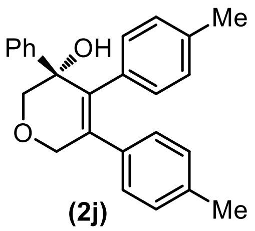

(2j)

According to General Procedure B, product **2j** (78.4 mg, 0.22 mmol, 81%) was obtained from **1a** (40.5 mg, 0.27 mmol) and 1,2-di-p-tolylethyne (61.8 mg, 0.30mmol) as white solid, mp 127-130 °C.

^{1}H NMR (400 MHz, $CDCl_3$) δ 7.54 – 7.50 (m, 2H), 7.32 – 7.27 (m, 2H), 7.22 – 7.17 (m, 1H), 6.97 – 6.91 (m, 4H), 6.73 (s, 4H), 4.58 (s, 2H), 4.00 – 3.93 (m, 2H), 2.71 (s, 1H), 2.23 (s, 3H), 2.10 (s, 3H). ^{13}C NMR (101 MHz, $cdcl_3$) δ 142.8, 136.9, 136.6 , 136.0, 135.0, 133.4,

130.3, 128.7,128.3, 128.2, 127.9, 126.9, 126.3, 77.2, 72.6, 70.1, 21.0. IR (neat) cm^{-1} ṽ: 3563, 3437, 3027, 2921, 2859, 1900, 1606, 1505, 1447, 1380, 1259, 1182, 1113, 1018, 961, 914, 808, 733, 700, 651. HRMS (EI(+), 70 eV) : $C_{25}H_{24}O_2$.[M]+: calcd. 356.1776, found: 356.1768. $[\alpha]_D^{20}$ = -24.8 (c = 0.66, CH_2Cl_2); HPLC (Chiralcel OD-H column, hexanes:i-PrOH = 90:10, 1.0 mL/min, 210 nm), t_{minor} = 4.8min, t_{major} =5.2 min, 94% ee.

(R)-3-(3-isopropoxyphenyl)-4,5-di-p-tolyl-3,6-dihydro-2H-pyran-3-ol (2k)

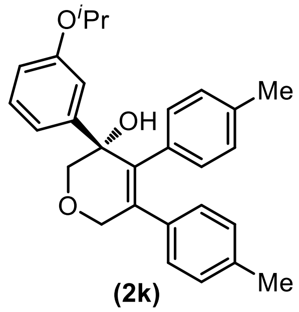

(2k)

According to General Procedure B, product **2k** (45.6 mg, 0.11 mmol, 73%) was obtained from **1i** (31.2 mg, 0.15 mmol) and 1,2-di-p-tolylethyne (34.0 mg, 0.16 mmol) as white solid, mp 136-139 °C.

^{1}H NMR (400 MHz, $CDCl_3$) δ 7.19 (t, *J* = 7.8 Hz, 1H), 7.10 – 7.05 (m, 2H), 6.94 (q, *J* = 8.2 Hz, 4H), 6.77 – 6.70 (m, 5H), 4.57 (s, 2H), 4.50 (dt, *J* = 12.1, 6.0 Hz, 1H), 4.00 – 3.93 (m, 2H), 2.62 (s, 1H), 2.24 (s, 3H), 2.12 (s, 3H), 1.27 (dd, *J* = 6.0, 2.5 Hz, 6H). ^{13}C NMR (101 MHz, $cdcl_3$) δ 157.6, 144.6, 136.9, 136.6, 136.5, 136.0, 135.1, 133.3, 130.3, 128.8, 128.7 128.3, 128.2, 118.8, 114.8, 114.2, 77.1, 72.6, 70.0, 69.9, 22.02, 22.0, 21.1, 21.0. IR (neat) cm^{-1} ṽ: 3460, 2965, 2918, 1595, 1260, 1094, 1023, 802, 698. HRMS (EI(+), 70 eV) : $C_{28}H_{30}O_3$.[M]+: calcd. 414.2195, found: 414.2197. $[\alpha]_D^{20}$ = -5.6 (c = 0.37, CH_2Cl_2); HPLC (Chiralcel OD-H column, hexanes:i-PrOH = 90:10, 1.0 mL/min, 210 nm), t_{minor} = 4.8min, t_{major} =5.2 min, 92% ee.

(R)-4,5-bis(4-methoxyphenyl)-3-phenyl-3,6-dihydro-2H-pyran-3-ol (2l)

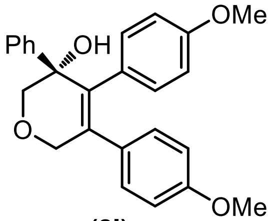

(2l)

According to General Procedure B, product **2l** (58.2 mg, 0.15 mmol, 65%) was obtained from **1a** (34.5 mg, 0.23 mmol) and 1,2-bis(4-methoxyphenyl)ethyne (60.2 mg, 0.25 mmol) as white solid, mp 130-132 °C.

^{1}H NMR (400 MHz, $CDCl_3$) δ 7.51 (d, *J* = 7.9 Hz, 2H), 7.30 (s, 2H), 7.21 (d, *J* = 7.1 Hz, 1H), 6.96 (d, *J* = 8.7 Hz, 2H), 6.76 (d, *J* = 8.7 Hz, 2H), 6.70 (d, *J* = 8.8 Hz, 2H), 6.49 (d, *J* = 8.8 Hz, 2H), 4.58 (s, 2H), 3.96 (t, *J* = 8.3 Hz, 2H), 3.74 (s, 3H), 3.64 (s, 3H), 2.61 (s, 1H). ^{13}C NMR (101 MHz, $cdcl_3$) δ 158.3, 157.9, 142.7, 136.4, 136.2, 131.6, 130.3, 129.6, 128.8, 127.9, 126.9, 126.3, 113.4, 112.9, 77.3, 72.8, 70.0, 55.0, 54.8. IR (neat) cm^{-1} ṽ: 3457, 2925,

2851,1669, 1607, 1508, 1454, 1288, 1247, 1179, 1114, 1030, 917, 822, 701, 657. HRMS (EI(+), 70 eV) : $C_{25}H_{24}O_4$.[M]+: calcd. 388.1700, found: 388.1684. $[\alpha]_D^{20}$ = 22.9 (c = 0.28, CH_2Cl_2); HPLC (Chiralcel OD-H column, hexanes:i-PrOH = 90:10, 1.0 mL/min, 210 nm), t_{minor} = 7.7min, t_{major} =8.5 min, 90% ee.

5-(4-fluorophenyl)-4-(4-methoxyphenyl)-3-phenyl-3,6-dihydro-2H-pyran-3-ol
4-(4-fluorophenyl)-5-(4-methoxyphenyl)-3-phenyl-3,6-dihydro-2H-pyran-3-ol (2o)

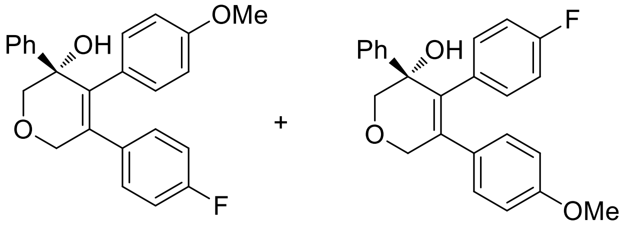

(2o) (1.2 :1)

According to General Procedure C, product **2o** (9.0 mg, 0.024 mmol, 25%) was obtained from **1a** (14.3 mg, 0.095 mmol) and 1-fluoro-4-((4-methoxyphenyl)ethynyl)benzene (23.7 mg, 0.105 mmol) as colorless oil.

^{1}H NMR (400 MHz, $CDCl_3$): δ 7.51 (d, J = 8.2 Hz, 1H), 7.46 (d, J = 8.1 Hz, 1H), 7.33 – 7.23 (m, 2H), 7.23 – 7.16 (m, 1H), 7.04 – 6.97 (m, 1H), 6.92 (d, J = 8.6 Hz, 1H), 6.89 – 6.82 (m, 2H), 6.72 (dd, J = 17.8, 8.5 Hz, 2H), 6.63 (t, J = 8.7 Hz, 1H), 6.49 (d, J = 8.5 Hz, 1H), 4.67 – 4.49 (m, 2H), 4.02 – 3.93 (m, 2H), 3.74 (s, 1.59 H), 3.63 (s, 1.33 H), 2.76 (s, 0.53 H), 2.63 (s, 0.45 H).

4,5-diethyl-3-phenyl-3,6-dihydro-2H-pyran-3-ol (2p)

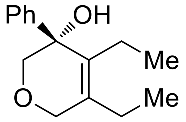

(2p)

According to General Procedure C, product **2p** (7.8 mg, 0.034 mmol, 17%) was obtained from **1a** (30.0 mg, 0.20 mmol) and hex-3-yne (18.0 mg, 0.22 mmol) as colorless oil.

^{1}H NMR (400 MHz, $CDCl_3$): δ 7.47 – 7.40 (m, 2H), 7.34 (t, *J* = 7.5 Hz, 2H), 7.29 – 7.22 (m, 1H), 4.18 (s, 2H), 3.71 (dd, *J* = 32.4, 11.5 Hz, 2H), 2.43 (s, 1H), 2.17 – 2.02 (m, 3H), 1.77 (td, *J* = 14.8, 7.3 Hz, 1H), 1.08 (t, *J* = 7.6 Hz, 3H), 0.88 (t, *J* = 7.6 Hz, 3H).

2-phenethyl-3,4,5-triphenyl-3,6-dihydro-2H-pyran-3-ol (4a)

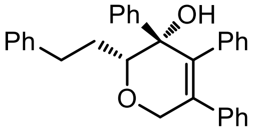

(4a)

According to General Procedure C, product **4a** (33.0 mg, 0.076 mmol, 97%) was obtained from **3a** (20.0 mg, 0.079 mmol) and diphenylacetylene (16.0 mg, 0.087mmol) as yellow

solid, mp 141-143 °C.

^{1}H NMR (400 MHz, $CDCl_3$): δ 7.26 (d, *J* = 7.5 Hz, 2H), 7.23 – 7.18 (m, 2H), 7.16 – 7.07 (m, 6H), 7.04 (t, *J* = 8.3 Hz, 3H), 6.97 (d, *J* = 7.5 Hz, 2H), 6.83 (s, 5H), 4.71 (d, *J* = 16.4 Hz, 1H), 4.55 (d, *J* = 16.4 Hz, 1H), 3.85 (d, *J* = 9.5 Hz, 1H), 2.89 – 2.77 (m, 2H), 2.59 – 2.50 (m, 1H), 2.08 – 1.96 (m, 1H), 1.63 – 1.53 (m, 1H). ^{13}C NMR (101 MHz, $CDCl_3$): δ 141.9, 140.9, 139.1, 138.1, 137.5, 137.2, 130.6, 128.5, 128.4, 128.2, 127.9, 127.6, 127.0, 126.9, 126.45, 126.37, 126.0, 125.6, 83.3, 74.8, 70.3, 31.9, 29.7. IR (neat) cm^{-1} ṽ: 3435, 3057, 3025, 2925, 2854, 1948, 1883, 1805, 1670, 1600, 1492, 1445, 1377, 1331, 1258, 1175, 1143, 1095, 1046, 1009, 903, 880, 800, 754, 724, 698; HRMS (DART) : $C_{31}H_{28}O_2$ $[M+NH_4]^+$: calcd. 450.2089, found: 450.2421.

2-hexyl-3,4,5-triphenyl-3,6-dihydro-2H-pyran-3-ol (4b)

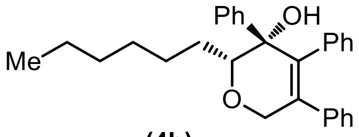

(4b)

According to General Procedure C, product **4b** (135.0 mg, 0.328 mmol, 96%) was obtained from **3b** (80.0 mg, 0.342 mmol) and diphenylacetylene (67.0 mg, 0.376mmol) as yellow solid, mp 139-142 °C.

^{1}H NMR (400 MHz, $CDCl_3$): δ 7.34 (d, *J* = 7.4 Hz, 2H), 7.20 – 7.06 (m, 6H), 7.02 – 6.98 (m, 2H), 6.85 (s, 5H), 4.76 (d, *J* = 16.4 Hz, 1H), 4.53 (d, *J* = 16.4 Hz, 1H), 3.87 (dd, *J* = 9.8, 1.8 Hz, 1H), 2.67 (s, 1H), 1.69 – 1.61 (m, 1H), 1.52 – 1.44 (m, 1H), 1.25 – 1.13 (m, 8H), 0.83 (t, *J* = 7.0 Hz, 3H). ^{13}C NMR (101 MHz, $CDCl_3$): δ 141.2, 139.2, 138.2, 137.6, 137.2, 130.6, 128.5, 127.9, 127.6, 126.89, 126.86, 126.41,126.36, 126.0, 84.6, 74.9, 70.3, 31.7, 29.1, 28.3, 26.2, 22.6, 14.0. IR (neat) cm^{-1} ṽ: 3059, 3028, 2955, 2924, 2855, 1945, 1744, 1658, 1633, 1601, 1492, 1447, 1377, 1259, 1173, 1095, 1014, 862, 797, 755, 730, 699, 662, 613; HRMS (DART) : $C_{29}H_{32}O_2$ $[M+NH_4]^+$: calcd. 430.2402, found: 430.2736.

2-(4-chlorobutyl)-3,4,5-triphenyl-3,6-dihydro-2H-pyran-3-ol (4c)

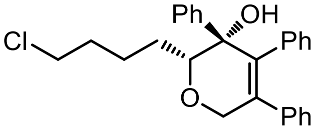

(4c)

According to General Procedure C, product **4c** (79.0 mg, 0.189 mmol, 91%) was obtained from **3c** (50.0 mg, 0.208 mmol) and diphenylacetylene (40.8 mg, 0.229 mmol) as yellow solid, mp 140-142 °C.

^{1}H NMR (400 MHz, $CDCl_3$): δ 7.38 (d, *J* = 7.6 Hz, 2H), 7.21 (t, *J* = 7.6 Hz, 2H), 7.17 – 7.08 (m, 4H), 7.05 – 6.99 (m, 2H), 6.93 – 6.83 (m, 5H), 4.80 (d, *J* = 16.4 Hz, 1H), 4.57 (d, *J* = 16.4 Hz, 1H), 3.92 (d, *J* = 9.7 Hz, 1H), 3.46 (t, *J* = 6.3 Hz, 2H), 2.93 (s, 1H), 1.79 – 1.64 (m, 4H), 1.44 – 1.35 (m, 1H), 1.32 – 1.27 (m, 1H). ^{13}C NMR (101 MHz, $CDCl_3$): δ 141.0, 139.0, 138.0, 137.4, 137.2, 130.5, 128.4, 127.9, 127.6, 126.94, 126.89, 126.5, 126.3, 126.0, 84.3, 74.8, 70.3, 44.9, 32.4, 27.6, 23.6. IR (neat) cm^{-1} ṽ: 3081, 3056, 3026, 2926, 2857, 1948, 1884, 1602, 1582, 1492, 1451, 1375, 1310, 1164, 1130, 1090, 1065, 1036, 996, 945, 927,

850, 753, 699, 678, 617; HRMS (DART) : $C_{27}H_{27}ClO_2$ $[M+NH_4]^+$: calcd. 436.1700, found: 436.2032.

2-(but-3-en-1-yl)-3,4,5-triphenyl-3,6-dihydro-2H-pyran-3-ol (4d)

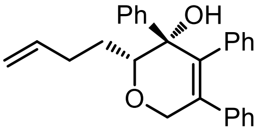

(4d)

According to General Procedure C, product **4d** (85.0 mg, 0.223 mmol, 91%) was obtained from **3d** (50.0 mg, 0.245 mmol) and diphenylacetylene (48.0 mg, 0.269 mmol) as yellow oil. ^{1}H NMR (400 MHz, $CDCl_3$): δ 7.34 (d, *J* = 8.0 Hz, 2H), 7.21 – 7.05 (m, 6H), 7.03 – 6.97 (m, 2H), 6.85 (s, 5H), 5.67 (dt, *J* = 17.1, 6.6 Hz, 1H), 4.99 – 4.86 (m, 2H), 4.74 (d, *J* = 16.4 Hz, 1H), 4.52 (d, *J* = 16.4 Hz, 1H), 3.90 (d, *J* = 9.8 Hz, 1H), 2.67 (s, 1H), 2.27 – 2.17 (m, 1H), 2.07 – 1.97 (m, 1H), 1.83 – 1.72 (m, 1H), 1.37 – 1.29 (m, 1H). ^{13}C NMR (101 MHz, $CDCl_3$): δ 141.0, 139.2, 138.4, 138.2, 137.5, 137.3, 130.6, 128.5, 127.9, 127.6, 126.94, 126.90, 126.45, 126.41, 126.0, 114.7, 83.5, 74.9, 70.3, 30.0, 27.5. IR (neat) cm^{-1} ṽ: 3404, 3059, 3027, 2975, 2926, 2852, 1951, 1883, 1809, 1641, 1601, 1491, 1446, 1380, 1320, 1259, 1189, 1176, 1136, 1089, 1044, 948, 913, 878, 754, 727, 698, 612; HRMS (DART) : $C_{27}H_{26}O_2$ $[M+NH_4]^+$: calcd. 400.1933, found: 400.2271.

2-cyclopropyl-3,4,5-triphenyl-3,6-dihydro-2H-pyran-3-ol (4e)

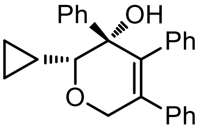

(4e)

According to General Procedure C, product **4e** (101.0 mg, 0.274 mmol, 87%) was obtained from **3e** (60.0 mg, 0.315 mmol) and diphenylacetylene (61.7 mg, 0.346 mmol) as yellow oil. ^{1}H NMR (400 MHz, $CDCl_3$): δ 7.38 (d, *J* = 7.5 Hz, 2H), 7.21 – 7.12 (m, 5H), 7.09 (t, *J* = 7.3 Hz, 1H), 7.02 (d, *J* = 2.1 Hz, 1H), 7.00 (d, *J* = 1.5 Hz, 1H), 6.96 – 6.92 (m, 2H), 6.89 – 6.85 (m, 3H), 4.76 (d, *J* = 16.4 Hz, 1H), 4.57 (d, *J* = 16.4 Hz, 1H), 3.27 (d, *J* = 8.1 Hz, 1H), 2.93 (s, 1H), 1.26 – 1.16 (m, 1H), 0.57 – 0.48 (m, 1H), 0.41 – 0.34 (m, 1H), 0.19 – 0.10 (m, 1H), -0.39 – -0.47 (m, 1H). ^{13}C NMR (101 MHz, $CDCl_3$): δ 141.4, 138.8, 138.2, 137.4, 137.2, 130.6, 128.4, 128.0, 127.4, 126.95, 126.92, 126.6, 126.3, 126.0, 88.4, 75.2, 70.4, 10.0, 3.5, 1.7. IR (neat) cm^{-1} ṽ: 3457, 3054, 3026, 2926, 1741, 1599, 1490, 1442, 1366, 1347, 1316, 1270, 1185, 1130, 1092, 1031, 995, 956, 896, 857, 828, 808, 753, 727, 696, 660, 625; HRMS (EI(+), 70 eV) : $C_{26}H_{24}O_2$ $[M-H_2O]^+$: calcd. 350.1776, found: 350.1667.

2-cyclohexyl-3,4,5-triphenyl-3,6-dihydro-2H-pyran-3-ol (4f)

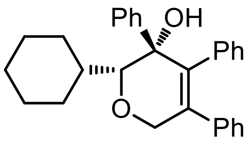

(4f)

According to General Procedure C, product **4f** (132.0 mg, 0.323 mmol, 94%) was obtained from **3f** (80.0 mg, 0.345 mmol) and diphenylacetylene (67.6 mg, 0.380 mmol) as yellow solid, mp 136-138 °C.

^{1}H NMR (400 MHz, $CDCl_3$): δ 7.34 (d, *J* = 7.5 Hz, 2H), 7.21 – 7.05 (m, 6H), 6.99 (d, *J* = 7.7 Hz, 2H), 6.87 – 6.78 (m, 5H), 4.73 (d, *J* = 16.3 Hz, 1H), 4.56 (d, *J* = 16.3 Hz, 1H), 3.75 (d, *J* = 3.9 Hz, 1H), 2.71 (s, 1H), 2.19 (d, *J* = 12.3 Hz, 1H), 1.72 (d, *J* = 11.6 Hz, 1H), 1.60 – 1.49 (m, 3H), 1.26 – 1.01 (m, 6H).^{13}C NMR (101 MHz, $CDCl_3$): δ 141.5, 139.9, 138.2, 137.5, 136.8, 130.7, 128.5, 127.9, 127.5, 126.85, 126.79, 126.5, 126.3, 125.9, 88.0, 75.9, 71.0, 37.9, 32.1, 28.1, 26.4, 26.3. IR (neat) cm^{-1} ṽ: 3488, 3056, 3026, 2925, 2852, 1942, 1731, 1680, 1599, 1557, 1541, 1492, 1446, 1378, 1324, 1259, 1173, 1094, 1050, 915, 883, 801, 756, 722, 698, 658, 618; HRMS (DART) : $C_{29}H_{30}O_2$ $[M+NH_4]^+$: calcd. 428.2246, found: 428.2578.

2-(2-(methylthio)ethyl)-3,4,5-triphenyl-3,6-dihydro-2H-pyran-3-ol (4g)

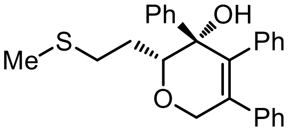

(4g)

According to General Procedure C, product **4g** (78.0 mg, 0.194 mmol, 73%) was obtained from **3g** (60.0 mg, 0.267 mmol) and diphenylacetylene (52.3 mg, 0.294 mmol) as yellow oil.

^{1}H NMR (400 MHz, $CDCl_3$): δ 7.36 (d, *J* = 7.5 Hz, 2H), 7.21 – 7.05 (m, 6H), 7.02 – 6.98 (m, 2H), 6.86 (s, 5H), 4.79 (d, *J* = 16.4 Hz, 1H), 4.52 (d, *J* = 16.4 Hz, 1H), 4.16 (dd, *J* = 9.6, 1.7 Hz, 1H), 2.72 (s, 1H), 2.66 – 2.57 (m, 1H), 2.55 – 2.46 (m, 1H), 2.02 – 1.91 (m, 1H), 1.82 (s, 3H), 1.56 – 1.47 (m, 1H).^{13}C NMR (101 MHz, $CDCl_3$): δ 140.7, 139.0, 138.1, 137.44, 137.39, 130.6, 128.5, 128.0, 127.7, 127.0, 126.95, 126.6, 126.4, 126.1, 82.2, 74.8, 70.4, 30.6, 27.3, 14.6. IR (neat) cm^{-1} ṽ: 2963, 2921, 2849, 1945, 1647, 1491, 1469, 1445, 1418, 1335, 1261, 1094, 1019, 865, 799, 756, 731, 699, 662, 613; HRMS (EI(+), 70 eV) : $C_{26}H_{26}O_2S$ $[M]^+$: calcd. 402.1654, found: 402.1657.

2,3,4,5-tetraphenyl-3,6-dihydro-2H-pyran-3-ol (4h)

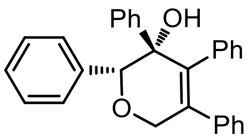

(4h)

According to General Procedure C, product **4h** (78.0 mg, 0.193 mmol, 88%) was obtained from **3h** (50.0 mg, 0.220 mmol) and diphenylacetylene (43.1 mg, 0.242 mmol) as yellow

solid, mp 145-147 °C.
^{1}H NMR (400 MHz, $CDCl_3$): δ 7.23 – 7.04 (m, 12H), 7.00 – 6.93 (m, 2H), 6.93 – 6.80 (m, 6H), 5.10 (s, 1H), 4.98 (d, J = 16.5 Hz, 1H), 4.72 (d, J = 16.5 Hz, 1H), 2.69 (s, 1H).
^{13}C NMR (101 MHz, $CDCl_3$): δ 140.9, 138.6, 138.1, 137.5, 137.4, 136.4, 130.7, 128.5, 128.0, 127.56, 127.53, 127.4, 127.3, 127.1, 126.9, 126.8, 126.6, 126.1, 86.1, 75.3, 70.6.
IR (neat) cm^{-1} ṽ: 3359, 3053, 2961, 2924, 2873, 2852, 1945, 1727, 1651, 1600, 1491, 1446, 1411, 1341, 1259, 1172, 1095, 1018, 862, 797, 754, 720, 699, 663, 615; HRMS (DART) : $C_{29}H_{24}O_2$ $[M+NH_4]^+$: calcd. 422.1776, found: 422.2110.

3,4,5-triphenyl-2-(2-(trifluoromethyl)phenyl)-3,6-dihydro-2H-pyran-3-ol (4i)

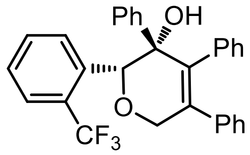

(4i)

According to General Procedure C, product **4i** (152.0 mg, 0.322 mmol, 83%) was obtained from **3i** (114.0 mg, 0.387 mmol) and diphenylacetylene (75.9 mg, 0.426 mmol) as yellow solid, mp 148-150 °C.
^{1}H NMR (400 MHz, $CDCl_3$): δ 8.50 (d, J = 8.3 Hz, 1H), 7.51 – 7.43 (m, 2H), 7.27 (t, J = 7.6 Hz, 1H), 7.20 (d, J = 7.6 Hz, 2H), 7.17 – 7.08 (m, 3H), 7.08 – 7.02 (m, 2H), 7.02 – 6.92 (m, 4H), 6.90 (d, J = 7.2 Hz, 1H), 6.88 – 6.82 (m, 3H), 5.45 (s, 1H), 5.01 (d, J = 16.6 Hz, 1H), 4.58 (d, J = 16.6 Hz, 1H), 3.19 (s, 1H).^{13}C NMR (101 MHz, $CDCl_3$): δ 140.0, 139.4, 137.9, 137.1, 137.0, 135.4, 131.4, 131.2, 131.1, 130.6, 129.2 (q, $J_{C\text{-}F}$ = 29.5 Hz), 128.5, 128.1, 127.2, 127.14, 127.07, 126.9, 126.5, 126.2, 125.72 (q, $J_{C\text{-}F}$ = 6.1 Hz), 124.1 (q, $J_{C\text{-}F}$ = 275.7 Hz), 79.7, 75.7, 70.7. IR (neat) cm^{-1} ṽ: 3441, 3378, 3088, 3056, 3029, 2957, 2926, 2855, 1746, 1669, 1652, 1604, 1492, 1449, 1378, 1309, 1259, 1163, 1126, 1091, 1036, 926, 880, 801, 754, 731, 699, 662, 619; HRMS (DART) : $C_{30}H_{23}F_3O_2$ $[M+NH_4]^+$: calcd. 490.1650, found: 490.1986.

4,5-diethyl-2-phenethyl-3-phenyl-3,6-dihydro-2H-pyran-3-ol (4j)

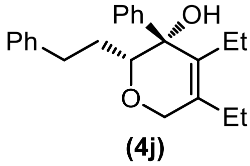

(4j)

According to General Procedure C, product **4j** (35.0 mg, 0.104 mmol, 75%) was obtained from **3a** (35.0 mg, 0.138 mmol) and 3-hexyne (12.5 mg, 0.152 mmol) as yellow oil.
^{1}H NMR (400 MHz, $CDCl_3$): δ 7.33 – 7.20 (m, 5H), 7.17 (t, J = 7.3 Hz, 2H), 7.13 – 7.08 (m, 1H), 6.97 (d, J = 7.3 Hz, 2H), 4.25 – 4.14 (m, 2H), 3.47 (d, J = 9.7 Hz, 1H), 2.79 – 2.70 (m, 1H), 2.47 – 2.37 (m, 1H), 2.16 (s, 1H), 2.13 – 1.93 (m, 3H), 1.90 – 1.80 (m, 1H), 1.71 – 1.62 (m, 1H), 1.49 – 1.39 (m, 1H), 1.05 (t, J = 7.6 Hz, 3H), 0.79 (t, J = 7.5 Hz, 3H).^{13}C NMR (101 MHz, $CDCl_3$): δ 142.1, 141.7, 136.0, 135.6, 128.4, 128.1, 127.8, 126.5, 126.3, 125.5, 83.3, 75.4, 69.0, 32.0, 29.6, 22.3, 21.6, 15.4, 13.2. IR (neat) cm^{-1} ṽ: 3449, 3062, 3025, 2967, 2932, 2872, 1943, 1877, 1741, 1692, 1601, 1493, 1448, 1375, 1345, 1261, 1189, 1174,

1157, 1106, 1050, 995, 949, 927, 899, 842, 753, 730, 696, 660, 634; HRMS (EI(+), 70 eV) : $C_{23}H_{28}O_2$ $[M-H_2O]^+$: calcd. 318.2089, found: 318.1978.

5-methyl-2-phenethyl-3,4-diphenyl-3,6-dihydro-2H-pyran-3-ol (4k)
4-methyl-2-phenethyl-3,5-diphenyl-3,6-dihydro-2H-pyran-3-ol (4k')

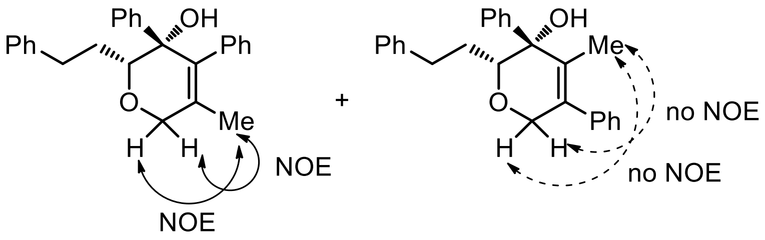

(4k) **(4k')**

40k : **40k'** = 2:1
According to General Procedure C, product **4k** and **4k'** (44.0 mg, 0.119 mmol, 86%) was obtained from **3a** (35.0 mg, 0.138 mmol) and 1-phenyl-1-propyne (17.6 mg, 0.152 mmol) as yellow oil.
^{1}H NMR (400 MHz, $CDCl_3$): δ 7.47 – 7.22 (m, 5H), 7.22 – 7.13 (m, 6H), 7.13 – 7.07 (m, 3H), 7.07 – 6.97 (m, 6H), 6.95 (d, *J* = 6.8 Hz, 2H), 4.52 (d, *J* = 16.0 Hz, 0.5H), 4.35 (d, *J* = 16.2 Hz, 1.5H), 4.26 (d, *J* = 16.2 Hz, 1H), 3.73 (dd, *J* = 9.8, 1.5 Hz, 1H), 3.61 (d, *J* = 8.4 Hz, 0.5H), 3.38 (s, 0.5H), 2.84 – 2.74 (m, 1.5H), 2.62 (s, 1H), 2.55 – 2.44 (m, 1.5H), 2.00 – 1.86 (m, 1.5H), 1.63 – 1.46 (m, 4.5H), 1.32 (s, 1.5H).^{13}C NMR (101 MHz, $CDCl_3$): δ 142.0, 141.3, 141.0, 137.8, 137.5, 133.4, 133.3, 132.0, 129.9, 128.6, 128.4, 128.3, 128.1, 127.5, 127.4, 127.2, , 126.7, 126.25, 126.18, 126.12, 125.5, 83.3, 82.8, 74.7, 74.5, 70.7, 70.4, 32.0, 31.9, 29.8, 29.6, 16.0, 15.2;IR (neat) cm^{-1} ṽ: 3550, 3443, 3059, 3025, 2929, 2863, 2818, 1948, 1885, 1808, 1769, 1693, 1600, 1492, 1447, 1380, 1330, 1173, 1136, 1103, 1032, 999, 935, 883, 849, 755, 699, 654, 625; HRMS (DART) : $C_{26}H_{26}O_2$ $[M+NH_4]^+$: calcd. 388.1933, found: 388.2270. In compound **4k**, an NOE between two methylene protons (δ = 4.35 and δ =4.26) and the methyl protons (δ = 1.52) was observed. On the other hand, in compound **4k'**, no NOE between two methylene protons (δ = 4.52 and δ = 4.35) and the methyl protons (δ = 1.32) was observed.

4,5-bis(4-methoxyphenyl)-2-phenethyl-3-phenyl-3,6-dihydro-2H-pyran-3-ol (4l)

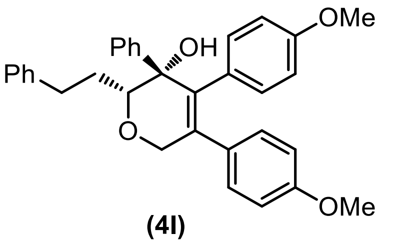

(4l)

According to General Procedure C, product **4l** (72.0 mg, 0.146 mmol, 73%) was obtained from **3a** (51.0 mg, 0.201 mmol) and 1,2-bis(4-methoxyphenyl)ethyne (52.7 mg, 0.221 mmol) as yellow solid, mp 149-151 °C.

^{1}H NMR (400 MHz, $CDCl_3$): δ 7.27 (d, *J* = 7.4 Hz, 2H), 7.22 – 7.11 (m, 5H), 7.10 – 7.04 (m, 1H), 7.01 (d, *J* = 7.0 Hz, 2H), 6.87 (d, *J* = 8.7 Hz, 2H), 6.73 (d, *J* = 8.8 Hz, 2H), 6.62 (d, *J* = 8.7 Hz, 2H), 6.38 (d, *J* = 8.8 Hz, 2H), 4.65 (d, *J* = 16.3 Hz, 1H), 4.53 (d, *J* = 16.3 Hz, 1H), 3.81 (dd, *J* = 9.8, 2.0 Hz, 1H), 3.67 (s, 3H), 3.54 (s, 3H), 2.90 (s, 1H), 2.88 – 2.78 (m, 1H), 2.57 – 2.47 (m, 1H), 2.07 – 1.97 (m, 1H), 1.62 – 1.53 (m, 1H).^{13}C NMR (101 MHz, $CDCl_3$): δ 158.2, 157.5, 141.9, 141.3, 138.0, 136.3, 131.7, 130.5, 130.1, 129.6, 128.4, 128.1, 127.6, 126.42, 126.35, 125.5, 113.3, 112.4, 83.5, 77.3, 77.0, 76.7, 75.0, 70.3, 55.0, 54.7, 31.9, 29.6; IR (neat) cm^{-1} ṽ: 3436, 3394, 3059, 3027, 2931, 2861, 1949, 1892, 1801, 1671, 1606, 1573, 1510, 1451, 1376, 1287, 1246, 1178, 1142, 1100, 1033, 971, 912, 878, 829, 752, 734, 700, 644, 619; HRMS (DART) : $C_{33}H_{32}O_4$ $[M]^+$: calcd. 492.2301, found: 492.2285.

4,5-bis(3-chlorophenyl)-2-phenethyl-3-phenyl-3,6-dihydro-2H-pyran-3-ol (4m)

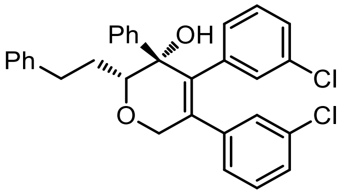

(4m)

According to General Procedure C, product **4m** (62.0 mg, 0.124 mmol, 63%) was obtained from **3a** (50.0 mg, 0.197 mmol) and 1,2-bis(3-chlorophenyl)ethyne (53.3 mg, 0.217 mmol) as yellow solid, mp 149-152 °C.

^{1}H NMR (400 MHz, $CDCl_3$): δ 7.25 – 7.18 (m, 4H), 7.17 – 7.12 (m, 3H), 7.11 – 7.03 (m, 3H), 7.01 (d, *J* = 8.8 Hz, 3H), 6.92 (s, 1H), 6.85 (d, *J* = 7.8 Hz, 1H), 6.81 – 6.73 (m, 2H), 6.68 (d, *J* = 7.2 Hz, 1H), 4.68 (d, *J* = 16.3 Hz, 1H), 4.47 (d, *J* = 16.5 Hz, 1H), 3.81 (d, *J* = 9.6 Hz, 1H), 2.81 (s, 2H), 2.59 – 2.49 (m, 1H), 2.06 – 1.92 (m, 1H), 1.60 – 1.49 (m, 1H).^{13}C NMR (101 MHz, $CDCl_3$): δ 141.7, 140.0, 139.4, 139.1, 138.9, 136.6, 134.0, 132.9, 130.1, 129.4, 128.7, 128.4, 128.23, 128.15, 127.8, 127.4, 126.8, 126.7, 126.5, 126.2, 125.7, 83.1, 74.6, 70.0, 31.8, 29.6. IR (neat) cm^{-1} ṽ: 3461, 3430, 3388, 3181, 3061, 3027, 2922, 2851, 2285, 1940, 1736, 1663, 1592, 1562, 1494, 1451, 1410, 1376, 1333, 1287, 1189, 1175, 1146, 1098, 1049, 1011, 960, 933, 881, 846, 784, 756, 727, 700, 626; HRMS (DART) : $C_{31}H_{26}Cl_2O_2$ $[M+NH_4]^+$: calcd. 518.1310, found: 518.1640

3-(naphthalen-1-yl)-2-phenethyl-4,5-diphenyl-3,6-dihydro-2H-pyran-3-ol (4n)

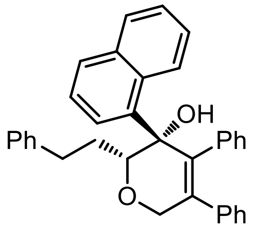

(4n)

According to General Procedure C, product **4n** (28.0 mg, 0.058 mmol, 62%) was obtained from **3n** (28.0 mg, 0.092 mmol) and diphenylacetylene (18.0 mg, 0.101 mmol) as yellow solid, mp 160-162 °C.

^1H NMR (400 MHz, $CDCl_3$): δ 8.36 (d, *J* = 5.5 Hz, 1H), 7.97 (d, *J* = 7.0 Hz, 1H), 7.79 – 7.73 (m, 1H), 7.65 (d, *J* = 8.1 Hz, 1H), 7.43 – 7.33 (m, 3H), 7.20 – 7.12 (m, 3H), 7.11 – 7.00 (m, 5H), 6.83 (d, *J* = 6.8 Hz, 4H), 6.74 (d, *J* = 6.9 Hz, 1H), 6.68 (t, *J* = 7.0 Hz, 2H), 4.92 (d, *J* = 16.8 Hz, 1H), 4.70 (d, *J* = 16.5 Hz, 1H), 4.49 (d, *J* = 8.8 Hz, 1H), 2.99 (s, 1H), 2.75 – 2.65 (m, 1H), 2.39 – 2.28 (m, 1H), 2.07 – 1.97 (m, 1H), 1.47 – 1.37 (m, 1H). ^{13}C NMR (101 MHz, $CDCl_3$): δ 141.7, 140.3, 138.3, 136.9, 136.0, 133.9, 131.4, 130.4, 129.6, 129.1, 128.4, 128.2, 128.14, 128.08, 128.0, 127.1, 126.6, 126.2, 125.4, 125.18, 125.12, 125.0, 124.8, 80.6, 75.0, 70.4, 31.8, 30.6. IR (neat) cm^{-1} ṽ: 3443, 3394, 3266, 3051, 2956, 2926, 2855, 1918, 1829, 1733, 1653, 1599, 1492, 1456, 1378, 1316, 1259, 1217, 1168, 1090, 1050, 881, 843, 802, 781, 757, 698, 658, 642, 619; HRMS (DART) : $C_{35}H_{30}O_2$ $[M+NH_4]^+$: calcd. 500.2246, found: 500.2575.

3-(4-methoxyphenyl)-2-phenethyl-4,5-diphenyl-3,6-dihydro-2H-pyran-3-ol (4o)

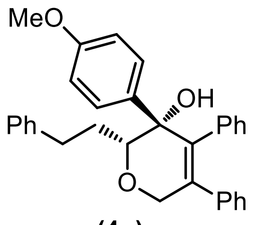

(4o)

According to General Procedure C, product **4o** (66.0 mg, 0.143 mmol, 90%) was obtained from **3o** (45.0 mg, 0.158 mmol) and diphenylacetylene (31.0 mg, 0.174 mmol) as yellow solid, mp 142-145 °C.

^1H NMR (400 MHz, $CDCl_3$): δ 7.23 – 7.11 (m, 5H), 7.11 – 7.01 (m, 5H), 6.98 – 6.93 (m, 2H), 6.83 (s, 5H), 6.67 (d, *J* = 8.8 Hz, 2H), 4.68 (d, *J* = 16.4 Hz, 1H), 4.54 (d, *J* = 16.4 Hz, 1H), 3.81 (dd, *J* = 10.0 Hz, 2.0 Hz, 1H), 3.69 (s, 3H), 2.89 – 2.79 (m, 2H), 2.60 – 2.50 (m, 1H), 2.07 – 1.95 (m, 1H), 1.65 – 1.56 (m, 1H). ^{13}C NMR (101 MHz, $CDCl_3$): δ 157.9, 142.0, 139.3, 138.2, 137.6, 137.1, 133.0, 130.6, 128.5, 128.4, 128.2, 127.9, 127.4, 126.91, 126.89, 126.0, 125.6, 113.0, 83.3, 74.5, 70.2, 55.0, 32.0, 29.6. IR (neat) cm^{-1} ṽ: 3427, 3345, 2953, 2924, 2854, 1734, 1653, 1601, 1510, 1493, 1459, 1377, 1305, 1286, 1256, 1169, 1092, 1029, 965, 927, 880, 803, 759, 731, 699, 630; HRMS (DART) : $C_{32}H_{30}O_3$ $[M+H]^+$: calcd. 463.2195, found: 463.2262.

2-phenethyl-4,5-diphenyl-3-(o-tolyl)-3,6-dihydro-2H-pyran-3-ol (4p)

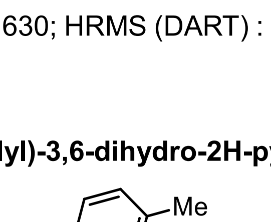

(4p)

According to General Procedure C, product **4p** (50.0 mg, 0.112 mmol, 93%) was obtained from **3p** (32.0 mg, 0.120 mmol) and diphenylacetylene (23.5 mg, 0.132 mmol) as yellow solid, mp 140-143 °C.

^{1}H NMR (400 MHz, DMSO): δ 7.62 (s, 1H), 7.23 – 7.05 (m, 6H), 7.04 – 6.87 (m, 8H), 6.84 (d, *J* = 7.0 Hz, 1H), 6.81 – 6.73 (m, 3H), 5.74 (s, 1H), 4.67 (d, *J* = 16.2 Hz, 1H), 4.39 (d, *J* = 16.2 Hz, 1H), 3.97 (d, *J* = 8.5 Hz, 1H), 2.73 – 2.64 (m, 1H), 2.48 – 2.39 (m, 1H), 2.23 (s, 3H), 1.98 – 1.86 (m, 1H), 1.35 – 1.24 (m, 1H).^{13}C NMR (101 MHz, DMSO): δ 141.9, 139.9 , 139.0, 138.0, 137.2, 135.9, 131.2, 130.0, 128.24, 128.16, 128.0, 127.9, 126.7, 126.45, 126.38, 125.7, 125.6, 125.0, 78.3, 73.3, 69.3, 31.3, 30.5, 20.6;IR (neat) cm^{-1} ṽ: 3330, 2973, 2927, 2883, 1924, 1651, 1452, 1419, 1379, 1327, 1274, 1087, 1045, 879, 804, 735, 697, 660; HRMS (DART) : $C_{32}H_{30}O_2$ $[M+NH_4]^+$: calcd. 464.2246, found: 464.2581.

3-(4-fluorophenyl)-2-phenethyl-4,5-diphenyl-3,6-dihydro-2H-pyran-3-ol (4q)

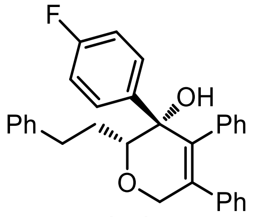

(4q)

According to General Procedure C, product **4q** (72.0 mg, 0.160 mmol, 87%) was obtained from **3q** (50.0 mg, 0.184 mmol) and diphenylacetylene (36.1 mg, 0.202 mmol) as yellow solid, mp 139-141 °C.

^{1}H NMR (400 MHz, $CDCl_3$): δ 7.21 (t, *J* = 7.1 Hz, 4H), 7.17 – 7.12 (m, 1H), 7.12 – 7.07 (m, 3H), 7.03 (d, *J* = 7.2 Hz, 2H), 6.97 – 6.92 (m, 2H), 6.88 – 6.76 (m, 7H), 4.71 (d, *J* = 16.4 Hz, 1H), 4.52 (d, *J* = 16.4 Hz, 1H), 3.79 (d, *J* = 8.6 Hz, 1H), 2.91 (s, 1H), 2.88 – 2.79 (m, 1H), 2.60 – 2.51 (m, 1H), 2.06 – 1.95 (m, 1H), 1.57 – 1.49 (m, 1H).^{13}C NMR (101 MHz, $CDCl_3$): δ 161.3 (d, $J_{C\text{-}F}$ = 245.8 Hz), 141.8, 139.0, 138.0, 137.4, 137.2, 136.7 (d, $J_{C\text{-}F}$ = 3.0 Hz), 130.4, 128.41, 128.40, 128.2, 128.04, 127.96, 127.0, 126.1, 125.7, 114.5 (d, $J_{C\text{-}F}$ = 21.3 Hz), 83.1, 74.6, 70.2, 31.9, 29.7. IR (neat) cm^{-1} ṽ: 3433, 3058, 3025, 2954, 2920, 2851, 1954, 1890, 1667, 1603, 1508, 1442, 1411, 1375, 1331, 1224, 1182, 1157, 1088, 1045, 1031, 1011, 951, 926, 879, 839, 812, 759, 698, 629; HRMS (DART) : $C_{31}H_{27}FO_2$ $[M+NH_4]^+$: calcd. 468.1995, found: 468.2327

2-pentyl-3,4,5-triphenyl-3,6-dihydro-2H-pyran-3-ol (4r)

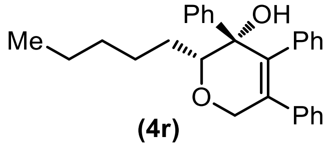

(4r)

According to General Procedure C, product **4r** (84.0 mg, 0.211 mmol, 97%) was obtained from **3r** (48.0 mg, 0.218 mmol) and diphenylacetylene (42.7 mg, 0.240 mmol) as yellow oil. ^{1}H NMR (400 MHz, $CDCl_3$): δ 7.40 (d, *J* = 7.5 Hz, 2H), 7.21 (t, *J* = 7.7 Hz, 2H), 7.17 – 7.08 (m, 4H), 7.06 – 7.00 (m, 2H), 6.94 – 6.86 (m, 5H), 4.82 (d, *J* = 16.4 Hz, 1H), 4.59 (d, *J* = 16.4 Hz, 1H), 3.94 (d, *J* = 8.8 Hz, 1H), 3.01 (s, 1H), 1.74 (dt, *J* = 16.3, 11.6 Hz, 1H), 1.61 – 1.50 (m, 1H), 1.31 – 1.15 (m, 6H), 0.87 (t, *J* = 6.6 Hz, 3H).^{13}C NMR (101 MHz, $CDCl_3$): δ 141.2, 139.2, 138.2, 137.6, 137.1, 130.6, 128.4, 127.9, 127.6, 126.87, 126.85, 126.4, 126.3, 125.9, 84.6, 74.9, 70.3, 31.6, 28.3, 25.9, 22.5, 14.0. IR (neat) cm^{-1} ṽ: 3447, 3057, 3025,

2953, 2925, 2857, 1946, 1881, 1807, 1679, 1599, 1576, 1492, 1444, 1377, 1331, 1259, 1176, 1096, 1073, 1028, 916, 879, 797, 755, 729, 698, 615; HRMS (DART) : $C_{28}H_{30}O_2$ $[M+NH_4]^+$: calcd. 416.2246, found: 416.2582.

(2R,3R)-2-phenethyl-3-phenyloxetan-3-ol (3a-*ent*)

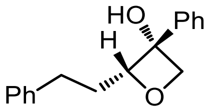

(3a-*ent*)

$[\alpha]_D^{20}$ = + 27.2 (c = 1.40, CH_2Cl_2); HPLC (Chiralcel OD-H column, hexanes:i-PrOH = 90:10, 1.0 mL/min, 210 nm), t_{minor} = 9.1 min, t_{major} = 11.6 min, 99% ee.

(2R,3R)-2-phenethyl-3,4,5-triphenyl-3,6-dihydro-2H-pyran-3-ol (4a-*ent*)

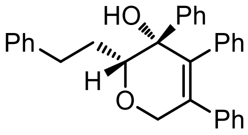

(4a-*ent*)

$[\alpha]_D^{20}$ = + 19.9 (c = 2.20, CH_2Cl_2); HPLC (Chiralcel IE-H column, hexanes:i-PrOH = 90:10, 1.0 mL/min, 210 nm), t_{minor} = 4.6 min, t_{major} = 5.6 min, 99% ee.

(2R,3R)-2,3-diphenyloxetan-3-ol (3h-*ent*)

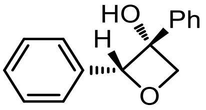

(3h-*ent*)

$[\alpha]_D^{20}$ = + 21.7 (c = 0.90, CH_2Cl_2); HPLC (Chiralcel OD-H column, hexanes:i-PrOH = 90:10, 1.0 mL/min, 210 nm), t_{minor} = 6.9 min, t_{major} = 7.7 min, 96% ee.

(2R,3R)-2,3,4,5-tetraphenyl-3,6-dihydro-2H-pyran-3-ol (4h-*ent*)

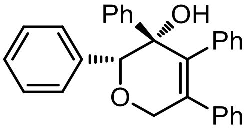

(4h-*ent*)

$[\alpha]_D^{20}$ = + 20.3 (c = 0.20, CH_2Cl_2); HPLC (Chiralcel OD-H column, hexanes:i-PrOH = 90:10, 1.0 mL/min, 210 nm), t_{major} = 6.4 min, t_{minor} = 11.3 min, 95% ee.

(2S,3S)-2-pentyl-3-phenyloxetan-3-ol (3r-*ent*)

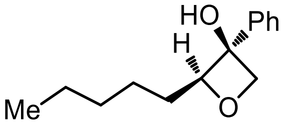

(3r-*ent*)

[α]$_D^{20}$ = -19.0 (c = 1.00, CH_2Cl_2); HPLC (Chiralcel OD-H column, hexanes:i-PrOH = 95:5, 1.0 mL/min, 210 nm), t_{minor} = 6.7 min, t_{major} = 7.5 min, 99% ee.

(2S,3S)-2-pentyl-3,4,5-triphenyl-3,6-dihydro-2H-pyran-3-ol (4r-*ent*)

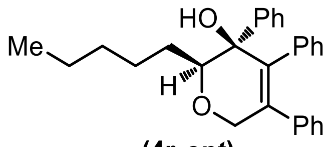

(4r-*ent*)

[α]$_D^{20}$ = -33.5 (c = 1.40, CH_2Cl_2); HPLC (Chiralcel OD-H column, hexanes:i-PrOH = 95:5, 1.0 mL/min, 210 nm), t_{minor} = 4.1 min, t_{major} = 4.5 min, 99% ee.

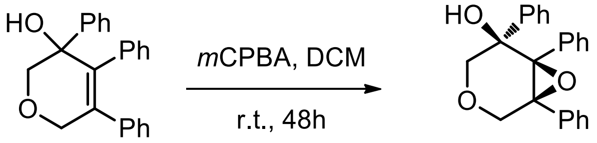

2a **5**

According to a procedure reported by Y.-P. Chang et al.[5] To the stirred solution of dihydropyran product **2a** (5.18 mmol, 1.70 g) in dry CH_2Cl_2 (26 mL), *m*-CPBA (7.77 mmol, 1.34 g) was added in one portion at 0 °C. The reaction mixture was slowly brought to 25 °C and stirred further for 48 h at the same temperature. The reaction was quenched with saturated sodium thiosulfate solution, and the mixture was extracted three times with CH_2Cl_2. The combined organic phases were dried over $MgSO_4$, concentrated in vacuo and the residue was purified by silica gel flash chromatography (hexane/EtOAc = 5:1) to give epoxide product **5** (1.10 g, 62% yield) as yellow oil.

^{1}H NMR (400 MHz, $CDCl_3$): δ 7.60 – 7.52 (m, 2H), 7.27 – 7.08 (m, 8H), 6.90 – 6.73 (m, 5H), 4.65 (d, *J* = 13.5 Hz, 1H), 4.28 (d, *J* = 13.5 Hz, 1H), 4.00 (d, *J* = 11.3 Hz, 1H), 3.91 (d, *J* = 11.3 Hz, 1H), 2.96 (s, 1H). ^{13}C NMR (101 MHz, $CDCl_3$): δ 140.9, 135.0 133.0, 129.2, 127.9, 127.78, 127.75, 127.4, 127.0, 126.6, 126.5 126.2, 73.6, 73.0, 72.9, 70.3, 68.7. IR (neat) cm^{-1} ṽ: 2963, 2916, 2854, 1494, 1448, 1404, 1260, 1092, 1023, 922, 866, 801, 697. HRMS (EI(+), 70 eV) : $C_{23}H_{20}O_3$ [M-H_2O]+: calcd. 326.1412, found: 326.1299.

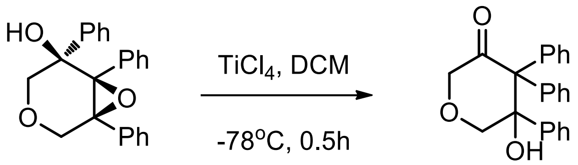

5 **6**

According to a procedure reported by K. Maruoka et al.[6] To a solution of epoxide **5** (200 mg, 0.58 mmol) in CH_2Cl_2 was added a 1 M CH_2Cl_2 solution of $TiCl_4$ (0.64 mL, 0.64 mmol) at -78 °C. The mixture was stirred at -78 °C for 30 min, and quenched with saturated NH_4Cl solution. The mixture was extracted three times with CH_2Cl_2. The combined organic phases were dried over $MgSO_4$, concentrated in vacuo and the residue was purified by

silica gel flash chromatography (hexane/EtOAc = 5:1) to give product **6** (137 mg, 48% yield) as yellow oil.

^{1}H NMR (400 MHz, $CDCl_3$): δ 7.26 – 7.19 (m, 5H), 7.08 – 7.03 (m, 2H), 7.00 – 6.93 (m, 6H), 6.93 – 6.88 (m, 2H), 4.82 – 4.73 (m, 2H), 4.65 (s, 1H), 4.16 (d, *J* = 12.7 Hz, 1H), 4.05 (d, *J* = 11.9 Hz, 1H); ^{13}C NMR (101 MHz, $CDCl_3$): δ 209.2, 141.0, 139.9, 136.8, 129.2, 128.9, 128.05, 127.99, 127.6, 127.36, 127.31, 127.26, 126.0, 76.9, 76.58, 76.56, 64.1. IR (neat) cm^{-1} ṽ: 2964, 1410, 1261, 1091, 1023, 865, 801, 694. HRMS (EI(+), 70 eV) : $C_{23}H_{20}O_3$ [M]+: calcd. 344.1412, found: 344.1406.

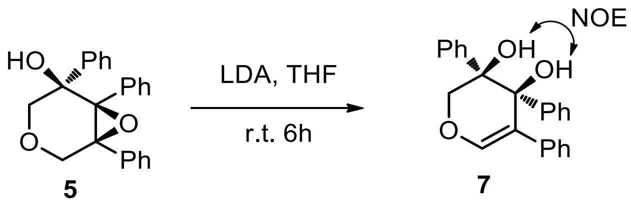

According to a procedure reported by B. Schmidt et al.[7] To a solution of LDA in THF (2.0 M, 0.6 mL, 1.17 mmol) under an atmosphere of dry argon was added a solution of epoxide **5** (130 mg, 0.38 mmol) in dry THF (2.0 mL). The mixture was stirred at room temperature for 6 h, and quenched with saturated NH_4Cl solution. The mixture was extracted three times with Et_2O. The combined organic phases were dried over $MgSO_4$, concentrated in vacuo and the residue was purified by silica gel flash chromatography (hexane/EtOAc = 5:1) to give product **7** (62 mg, 48% yield) as colorless oil.

^{1}H NMR (400 MHz, $CDCl_3$): δ 7.28 – 7.12 (m, 9H), 7.11 – 7.05 (m, 4H), 7.04 (s, 1H), 6.79 (d, *J* = 7.6 Hz, 2H), 4.77 (d, *J* = 12.1 Hz, 1H), 4.06 (d, *J* = 12.1 Hz, 1H), 3.57 (s, 1H), 3.13 (s, 1H); ^{13}C NMR (101 MHz, $CDCl_3$): δ 142.2, 141.7, 138.0, 135.3, 128.3, 128.1, 128.0, 127.5, 127.3, 127.0, 126.8, 126.2, 117.9, 77.6, 73.7, 69.0. IR (neat) cm^{-1} ṽ: 3514, 3358, 3186, 2921, 2853, 1636, 1456, 1328, 1260, 1188, 1018, 913, 800, 752, 698, 624. HRMS (EI(+), 70 eV) : $C_{23}H_{20}O_3$ [M]+: calcd. 344.1412, found: 344.1415. An NOE between two adjacent hydroxyl protons (δ = 3.57 and δ = 3.13) was observed.

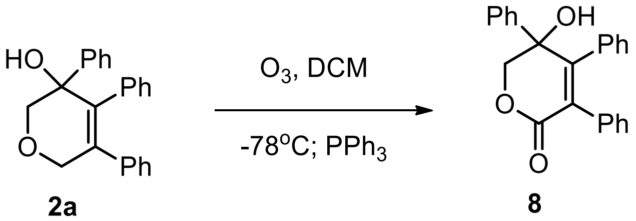

According to a procedure reported by E. V. Boltukhina et al.[8] To a stirred solution of dihydropyran product **2a** (180 mg, 0.55 mmol) in CH_2Cl_2 (6 mL) at -78°C was bubbled in ozone until light blue color appeared. Excessive ozone was removed by a nitrogen flow, then PPh_3 (288 mg, 1.1 mmol) was added. The mixture was left overnight, dried over $MgSO_4$, concentrated in vacuo and the residue was purified by silica gel flash chromatography (hexane/EtOAc = 4:1) to give product **8** (79 mg, 42% yield) as white solid, mp 115-117 °C.

^{1}H NMR (400 MHz, DMSO): δ 7.48 (d, *J* = 7.4 Hz, 2H), 7.27 (t, *J* = 7.6 Hz, 2H), 7.22 – 7.13 (m, 4H), 7.07 (dd, *J* = 7.4, 1.8 Hz, 2H), 6.97 – 6.91 (m, 3H), 6.91 – 6.85 (m, 2H), 6.73 (s, 1H), 4.71 (d, *J* = 11.3 Hz, 1H), 4.43 (d, *J* = 11.3 Hz, 1H); ^{13}C NMR (101 MHz, DMSO): δ 164.0, 155.2, 140.8, 135.63, 135.56, 130.6, 130.5, 129.2, 127.9, 127.41, 127.35, 127.26, 127.0, 126.4, 76.1, 71.7. IR (neat) cm^{-1} ṽ: 3363, 2962, 1702, 1491, 1448, 1395, 1321, 1261, 1093, 1023, 866, 802, 699. HRMS (EI(+), 70 eV) : $C_{23}H_{18}O_3$ [M]+: calcd. 342.1258, found: 342.1263.

6. Reference

1. a) L. Ye, W. He, L. Zhang, *J. Am. Chem. Soc.* **2010**, *132*, 8550; b) A. S. K. Hashmi, A. Loos, A. Littmann, I. Braun, J. Knight, S. Doherty, F. Rominger, *Adv. Synth. Catal.* **2009**, *351*, 576.
2. All Ligands were commercial reagents from Strem company.
3. Z. Wang, Z. Chen, J. Sun, *Angew. Chem. Int. Ed.* **2013**, *52*, 6685.
4. W. Yang, Z. Wang, J. Sun, *Angew.Chem.Int. Ed.* **2016**, *55*, 6954.
5. Y.-P. Chang, R. Gurubrahamam, K. Chen, *Org. Lett.* **2015***, 17,* 2908.
6. K.Maruoka, M. Hasegawa, H. Yamamoto, *J. Am. Chem. Soc.* **1986**, *108*, 3827.
7. B. Schmidt, H. Wildemann, *Synlett ,* **1999,** *10,* 1591.
8. E. V. Boltukhina, A. E. Sheshenev, I. M. Lyapkalo, *Tetrahedron*, **2011**, *67*, 5382.

Copies of NMR spectra and HPLC chromatographs

^{1}H NMR (400 MHz, $CDCl_3$) 2-hexyloxetan-3-one (S-2b)

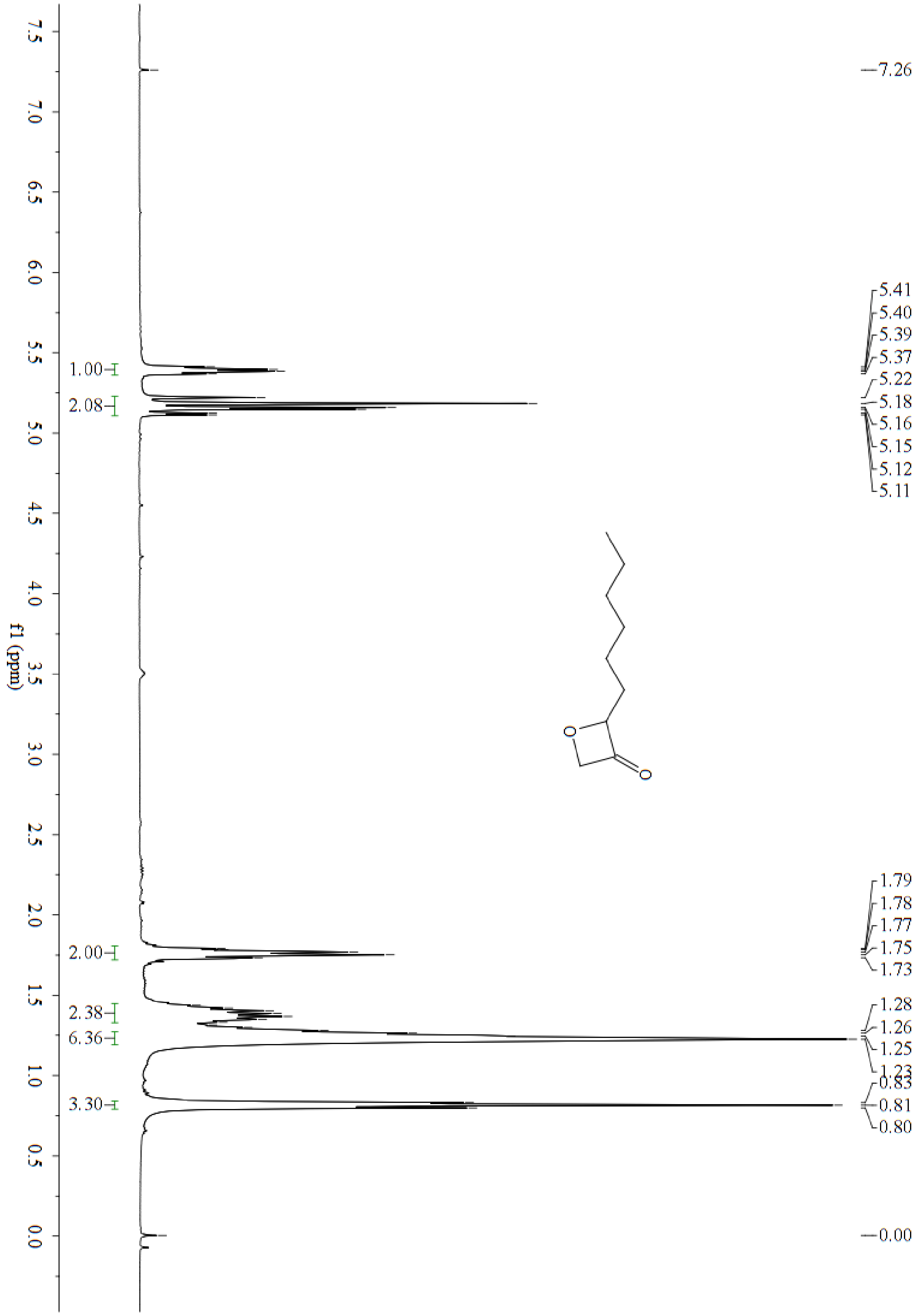

^{13}C NMR (101 MHz, CDCl$_3$) 2-hexyloxetan-3-one (S-2b)

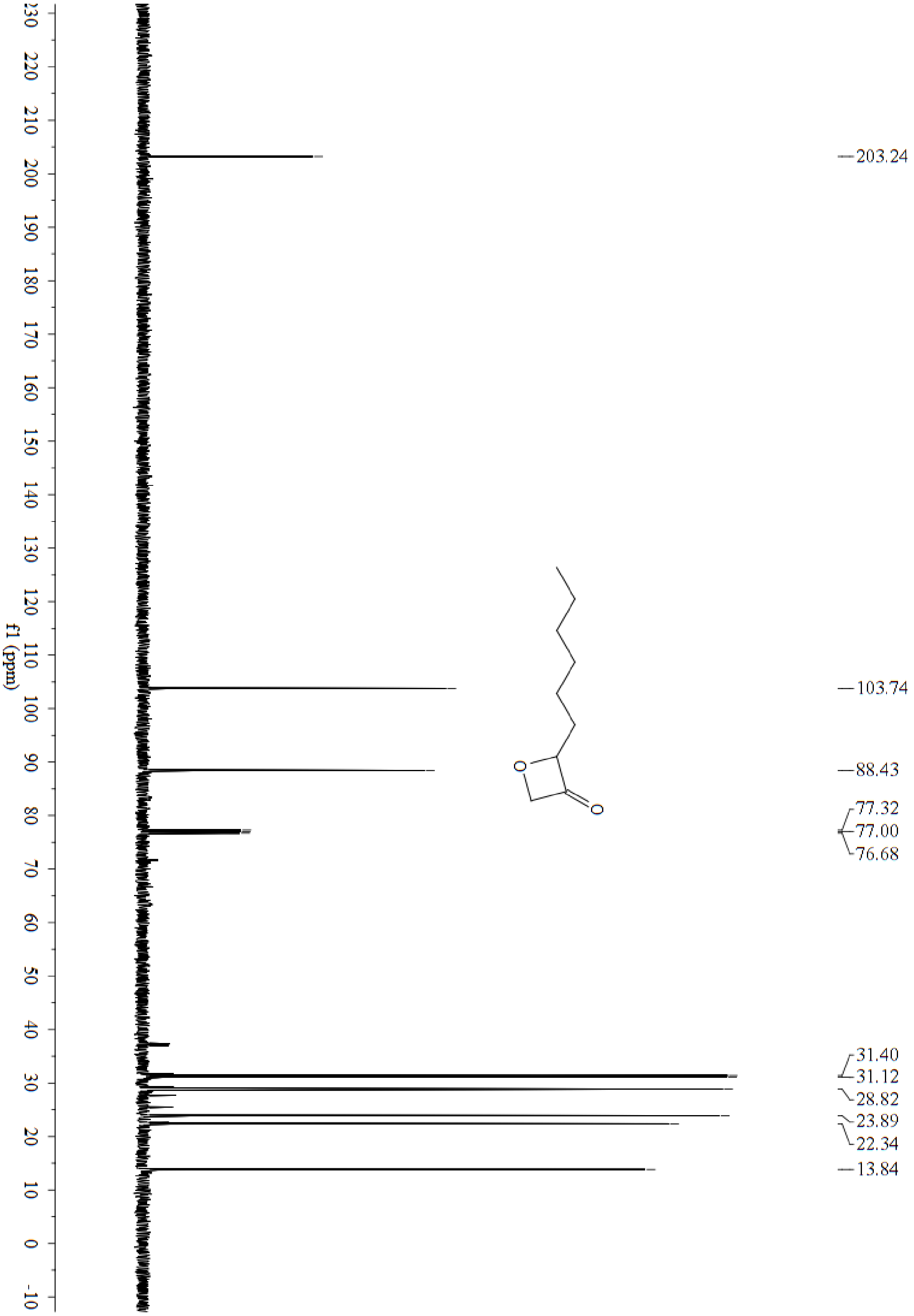

^{1}H NMR (400 MHz, $CDCl_3$) 2-(4-chlorobutyl)oxetan-3-one (S-2c)

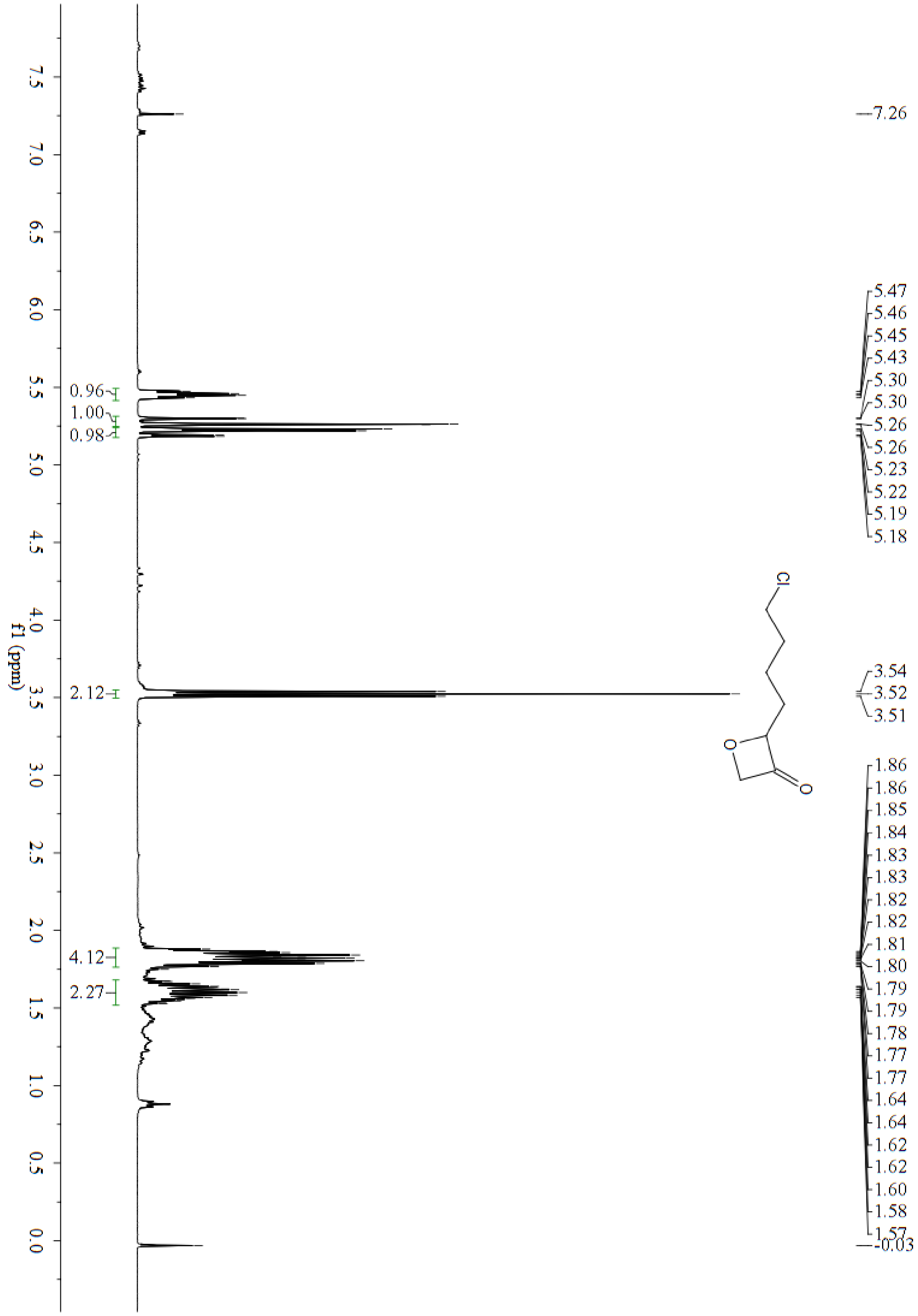

^{13}C NMR (101 MHz, $CDCl_3$) 2-(4-chlorobutyl)oxetan-3-one (S-2c)

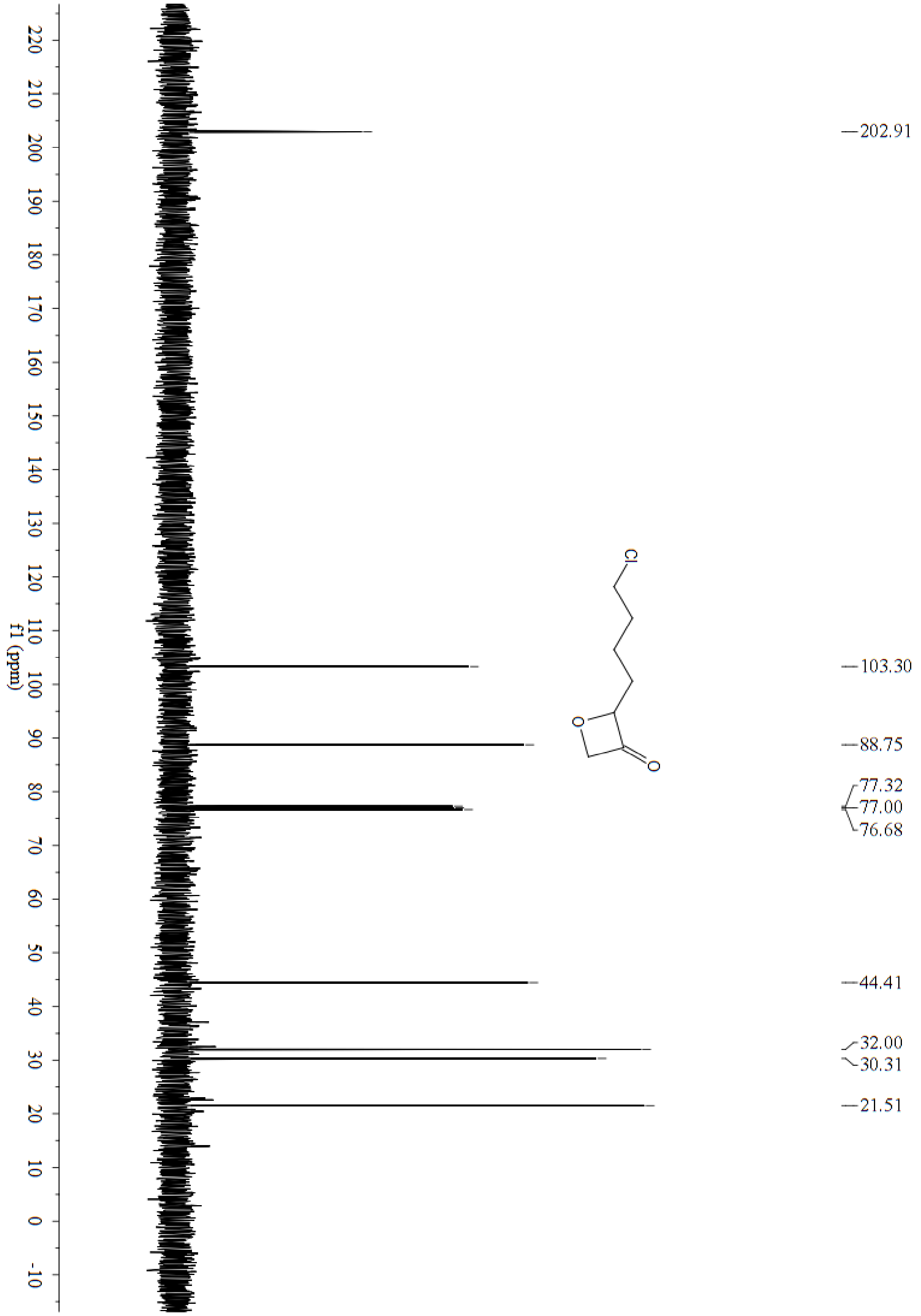

^{1}H NMR (400 MHz, $CDCl_3$)   2-(but-3-en-1-yl)oxetan-3-one (S-2d)

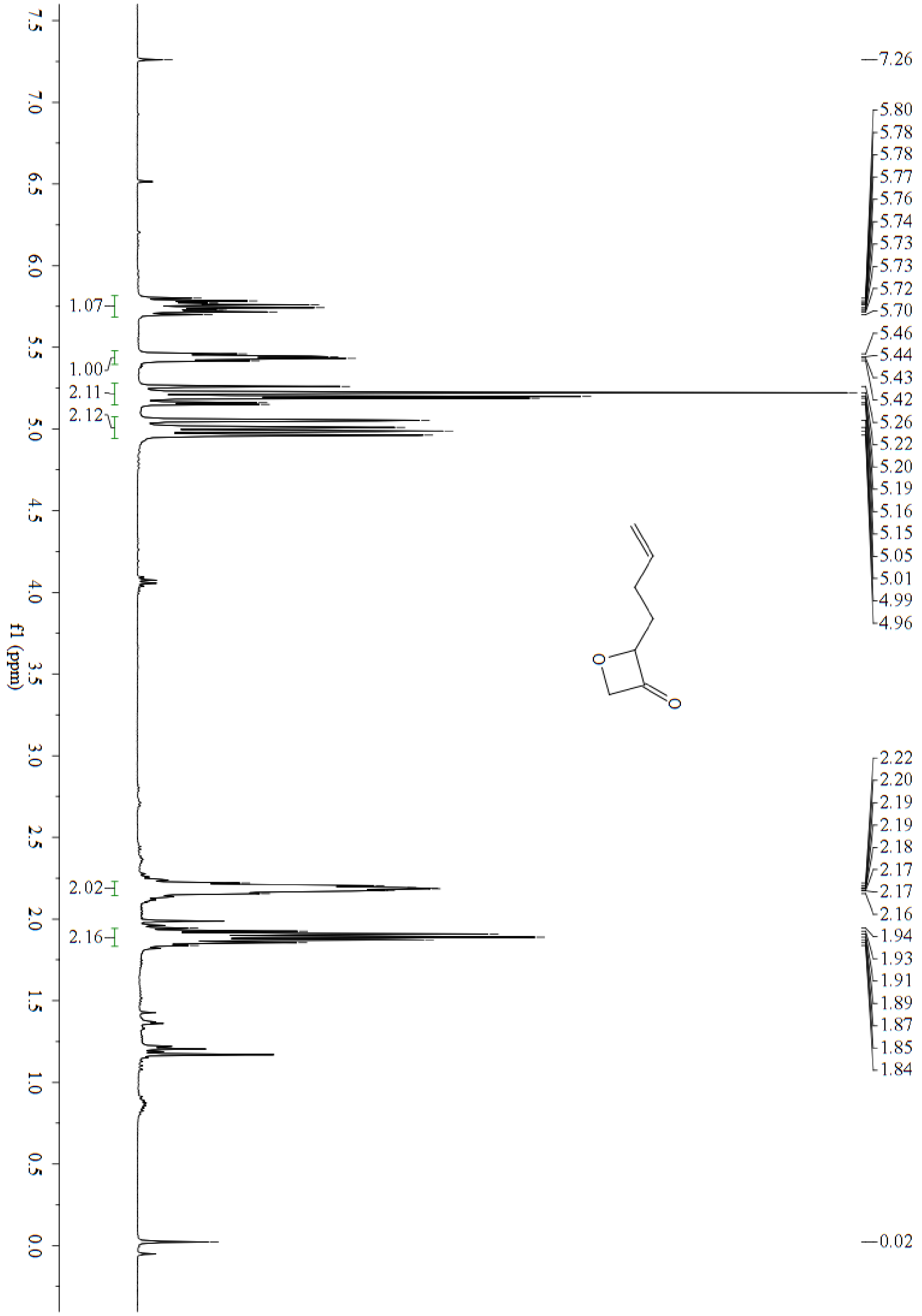

^{13}C NMR (101 MHz, $CDCl_3$) 2-(but-3-en-1-yl)oxetan-3-one (S-2d)

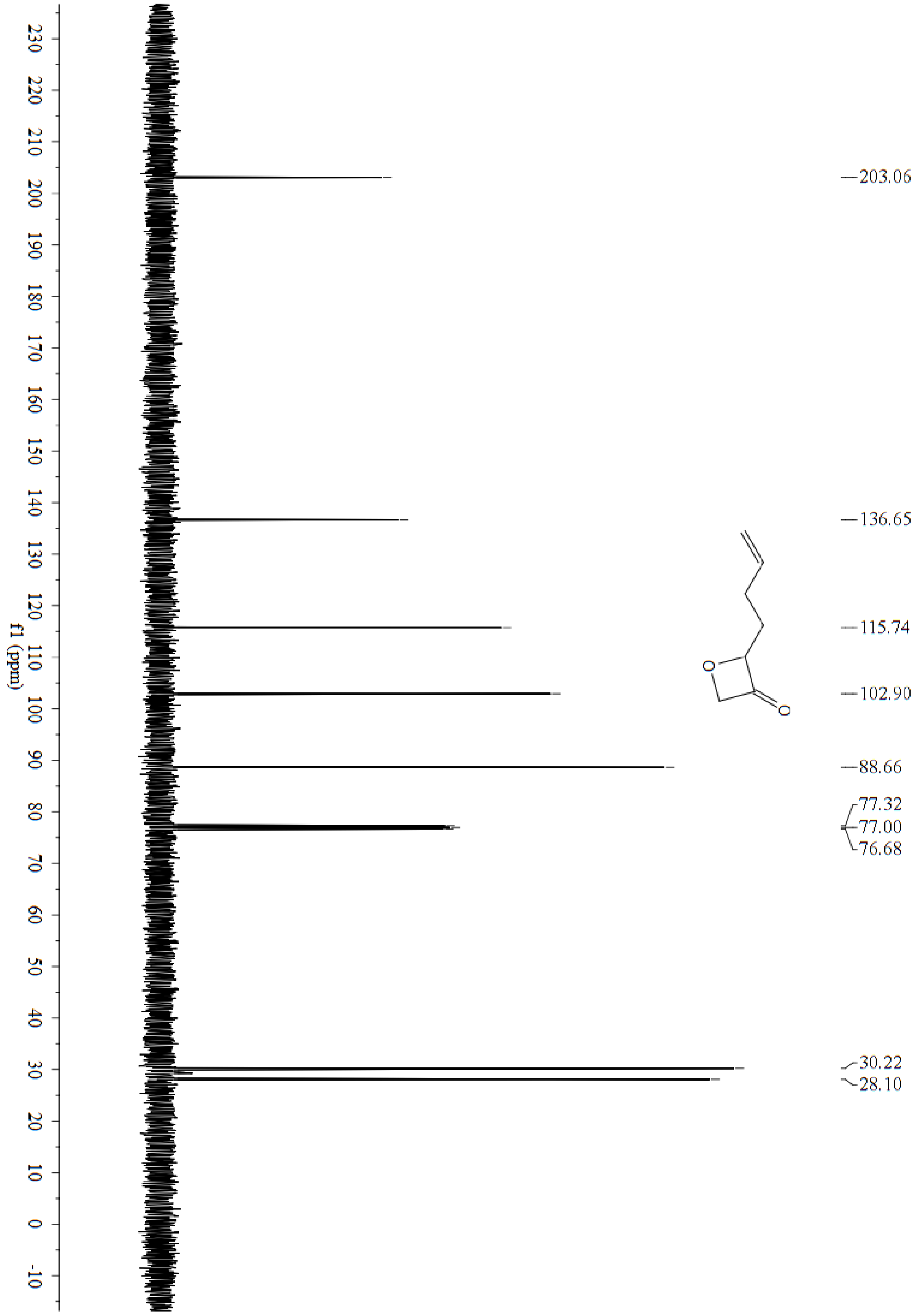

^{1}H NMR (400 MHz, $CDCl_3$) 2-cyclopropyloxetan-3-one (S-2e)

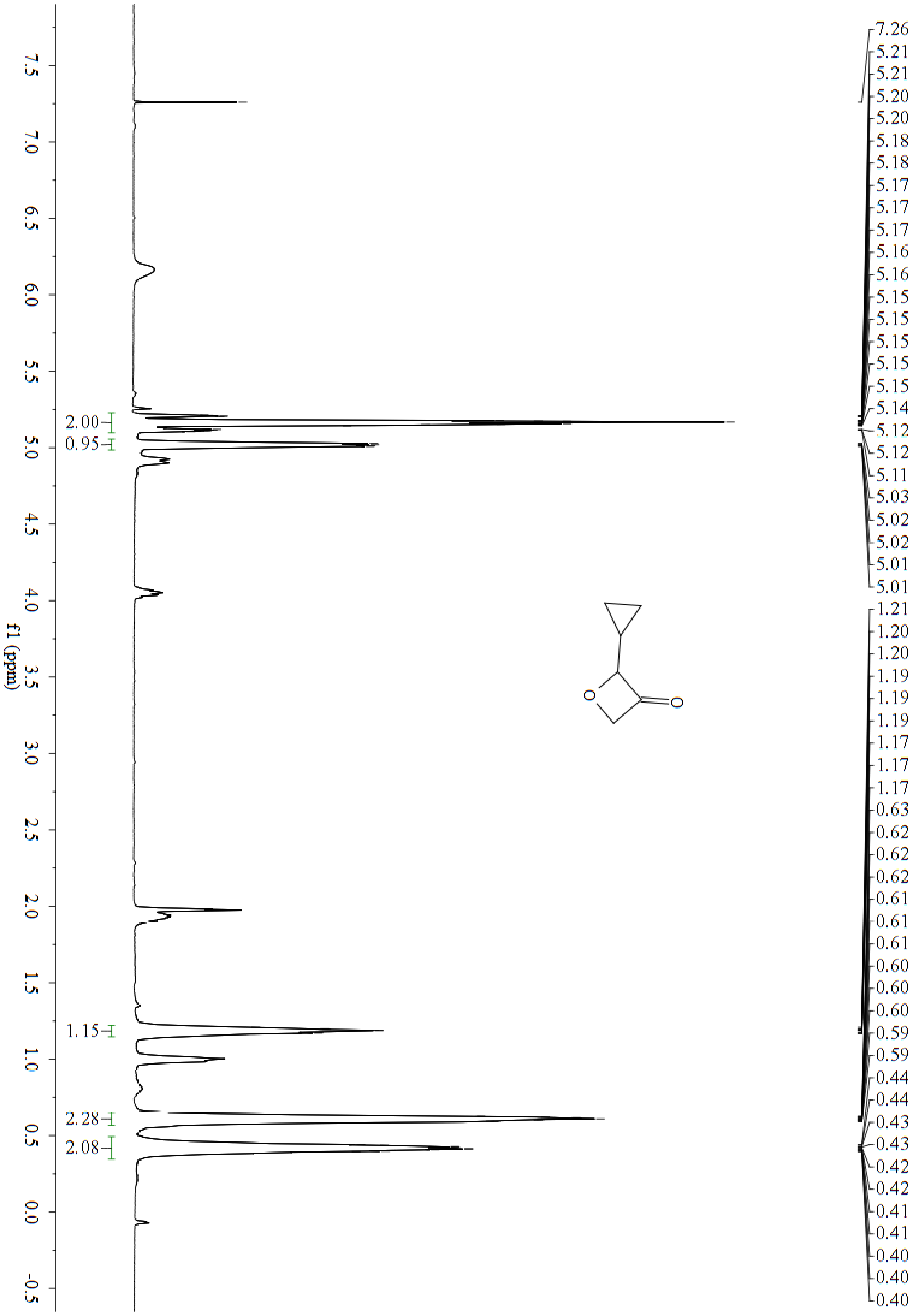

^{13}C NMR (101 MHz, $CDCl_3$) 2-cyclopropyloxetan-3-one (S-2e)

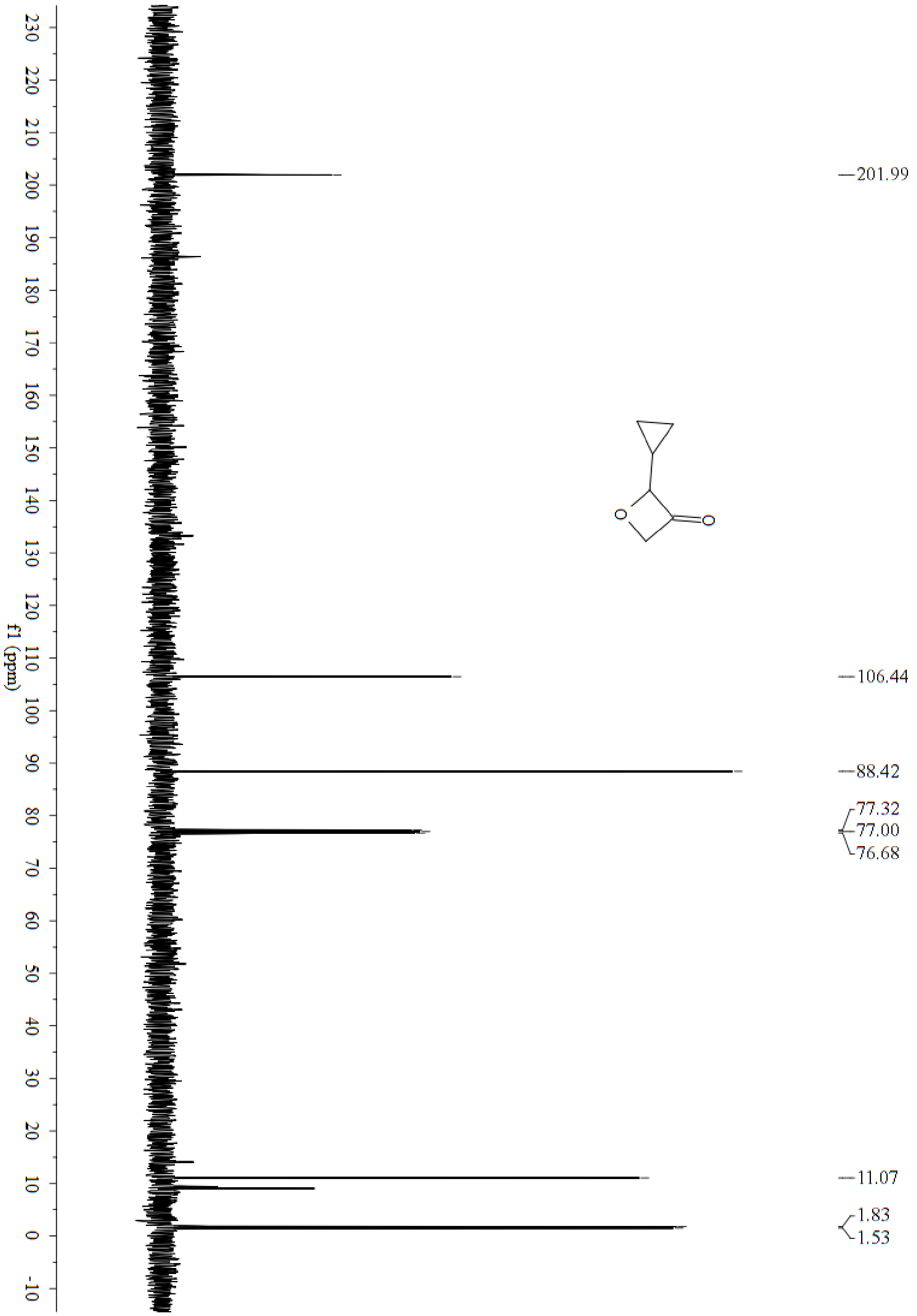

^{1}H NMR (400 MHz, $CDCl_3$)   2-(2-(methylthio)ethyl)oxetan-3-one (S-2g)

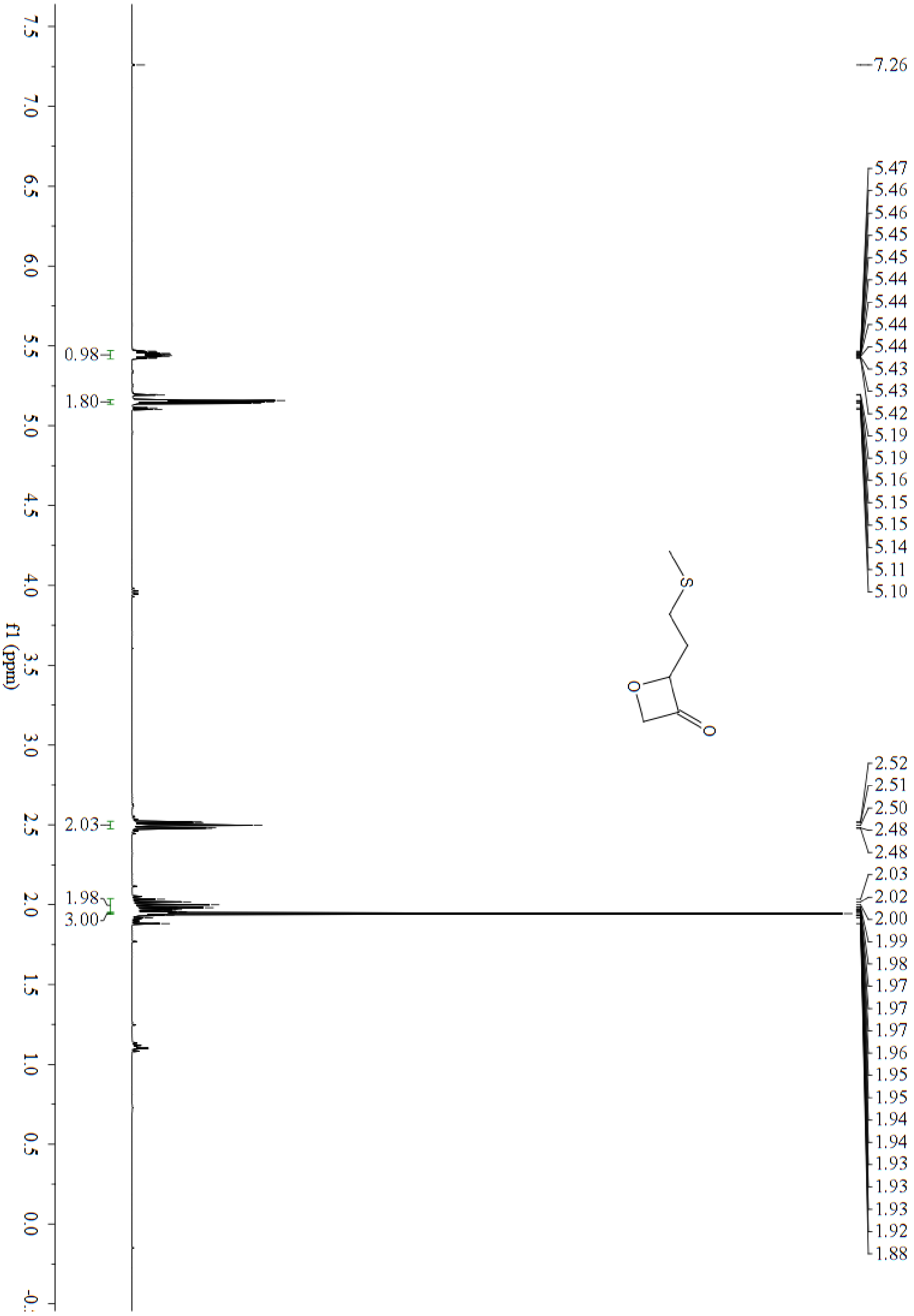

^{13}C NMR (101 MHz, $CDCl_3$) 2-(2-(methylthio)ethyl)oxetan-3-one (S-2g)

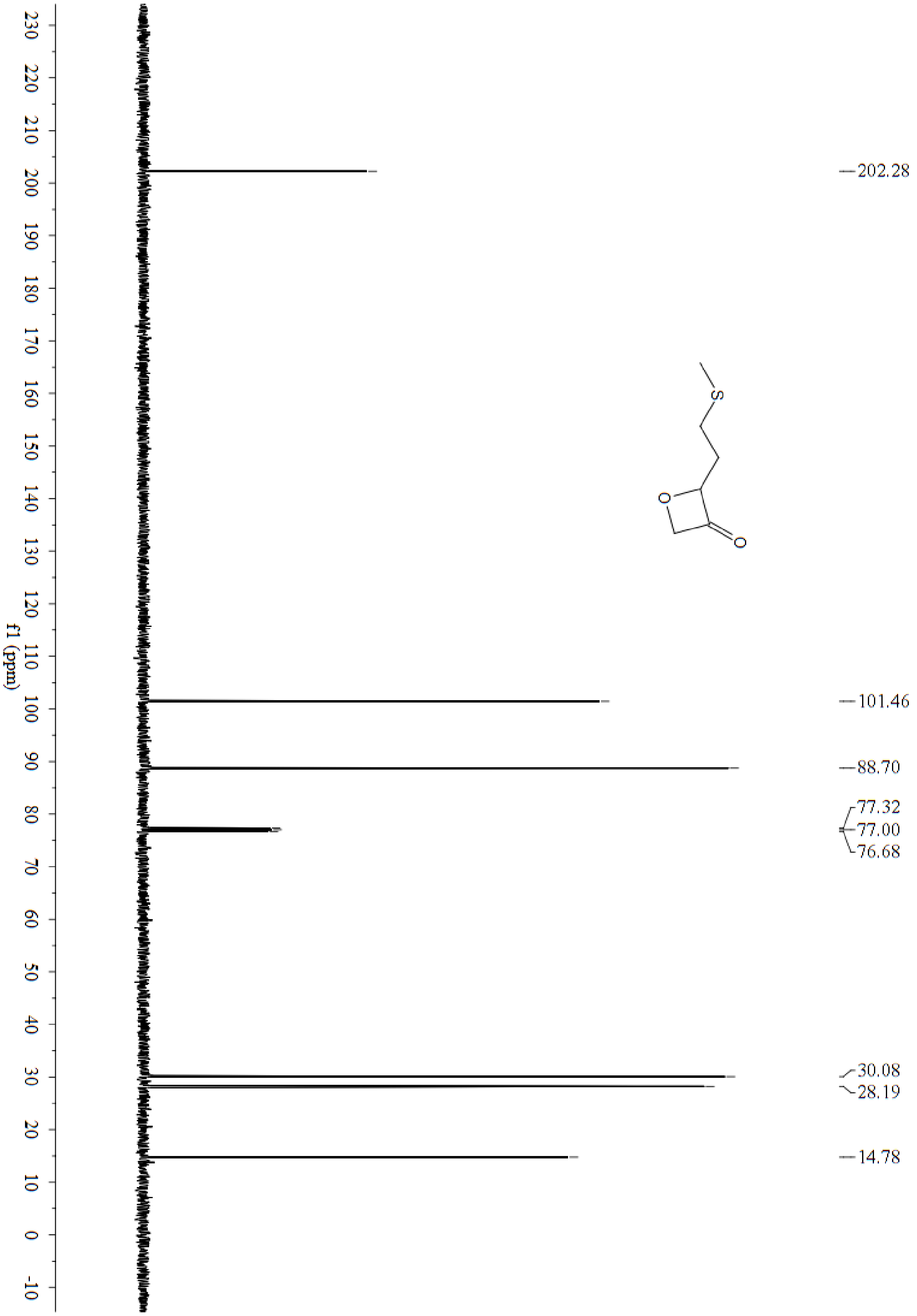

^{1}HNMR (400MHz,$CDCl_3$) 2-(2-(trifluoromethyl)phenyl)oxetan-3-one (S-2i)

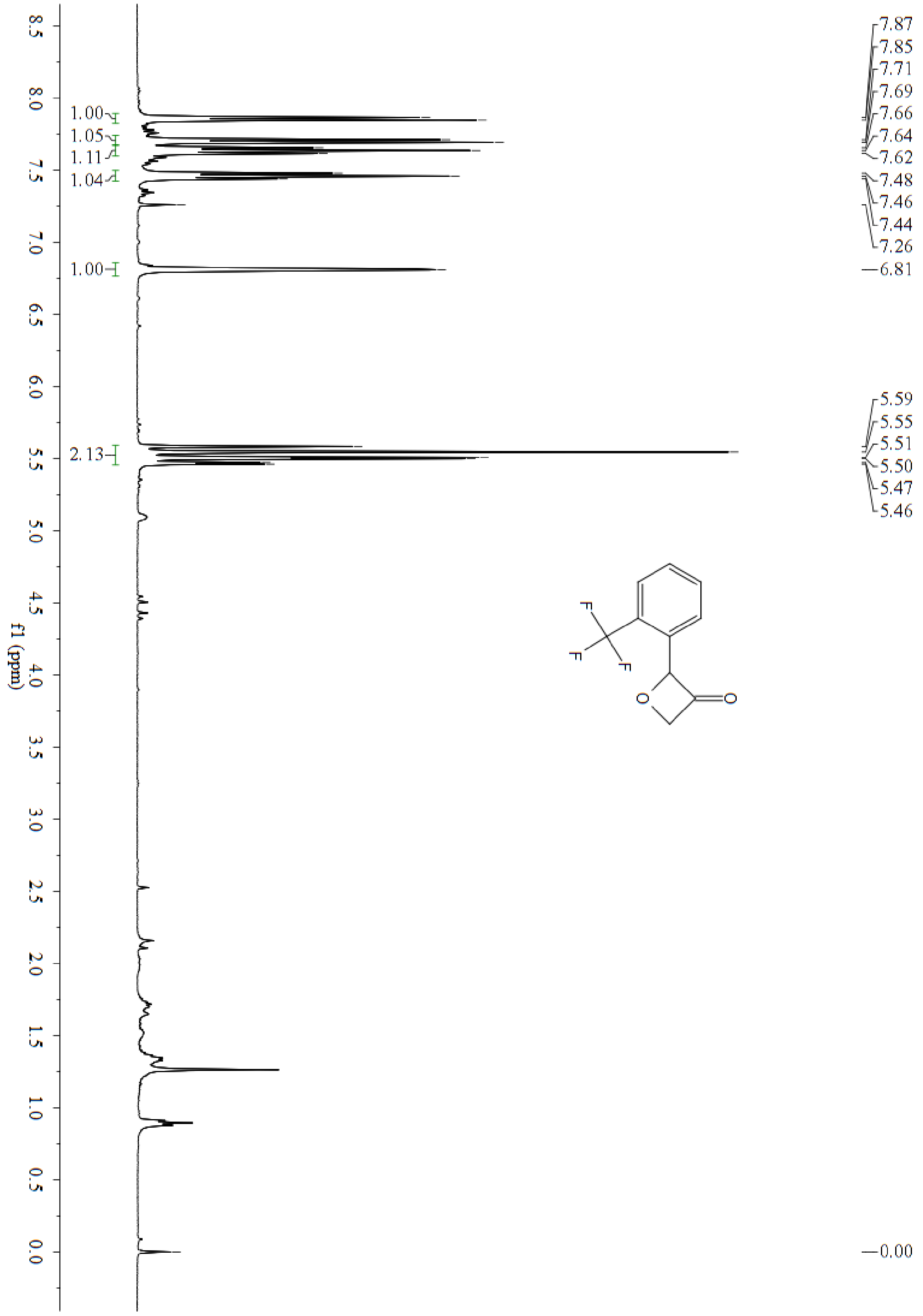

^{13}C NMR (101 MHz, $CDCl_3$) 2-(2-(trifluoromethyl)phenyl)oxetan-3-one (S-2i)

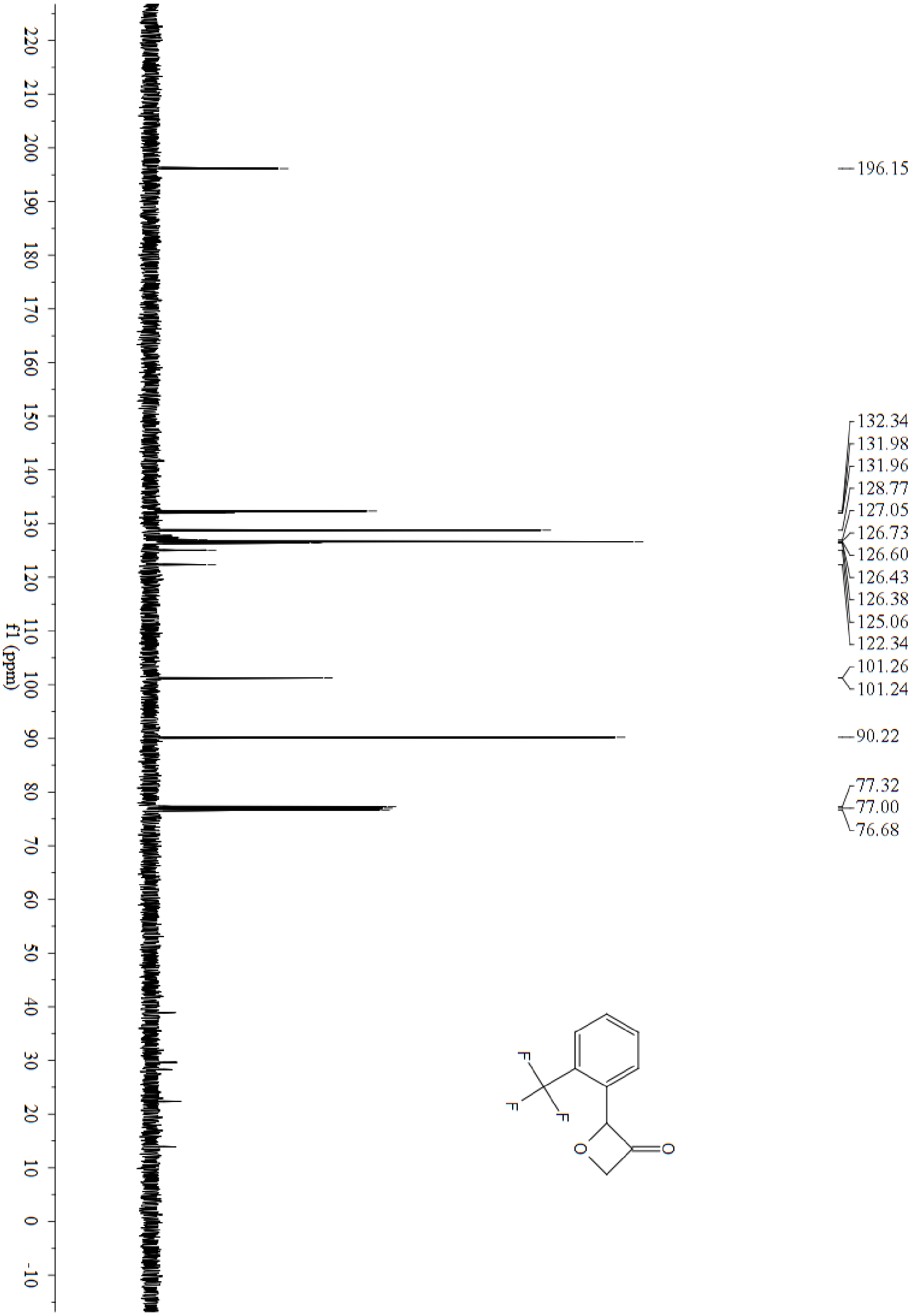

^{1}H NMR (400MHz,$CDCl_3$)   2-pentyloxetan-3-one (S-2r)

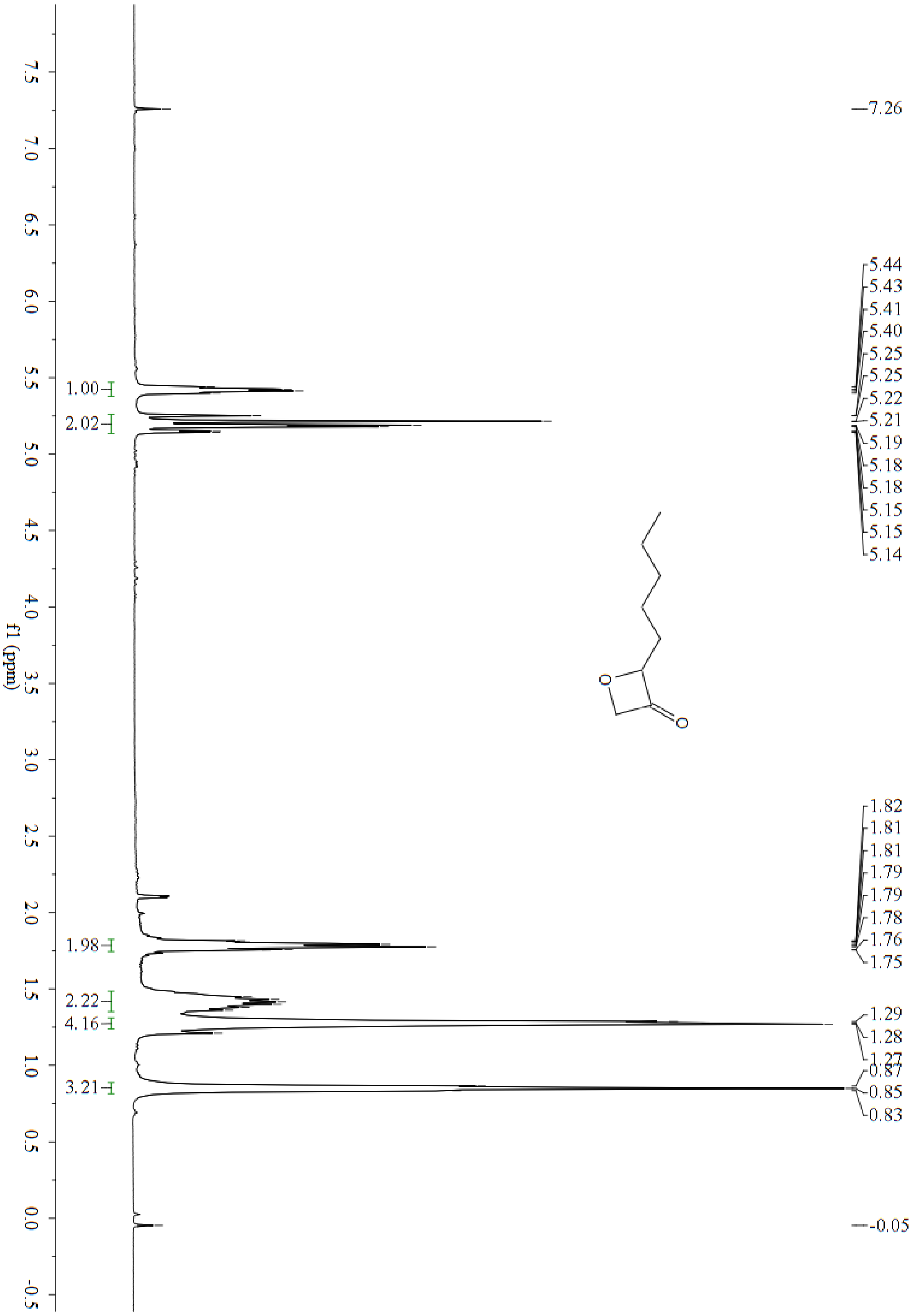

^{13}C NMR (101MHz,$CDCl_3$)   2-pentyloxetan-3-one (S-2r)

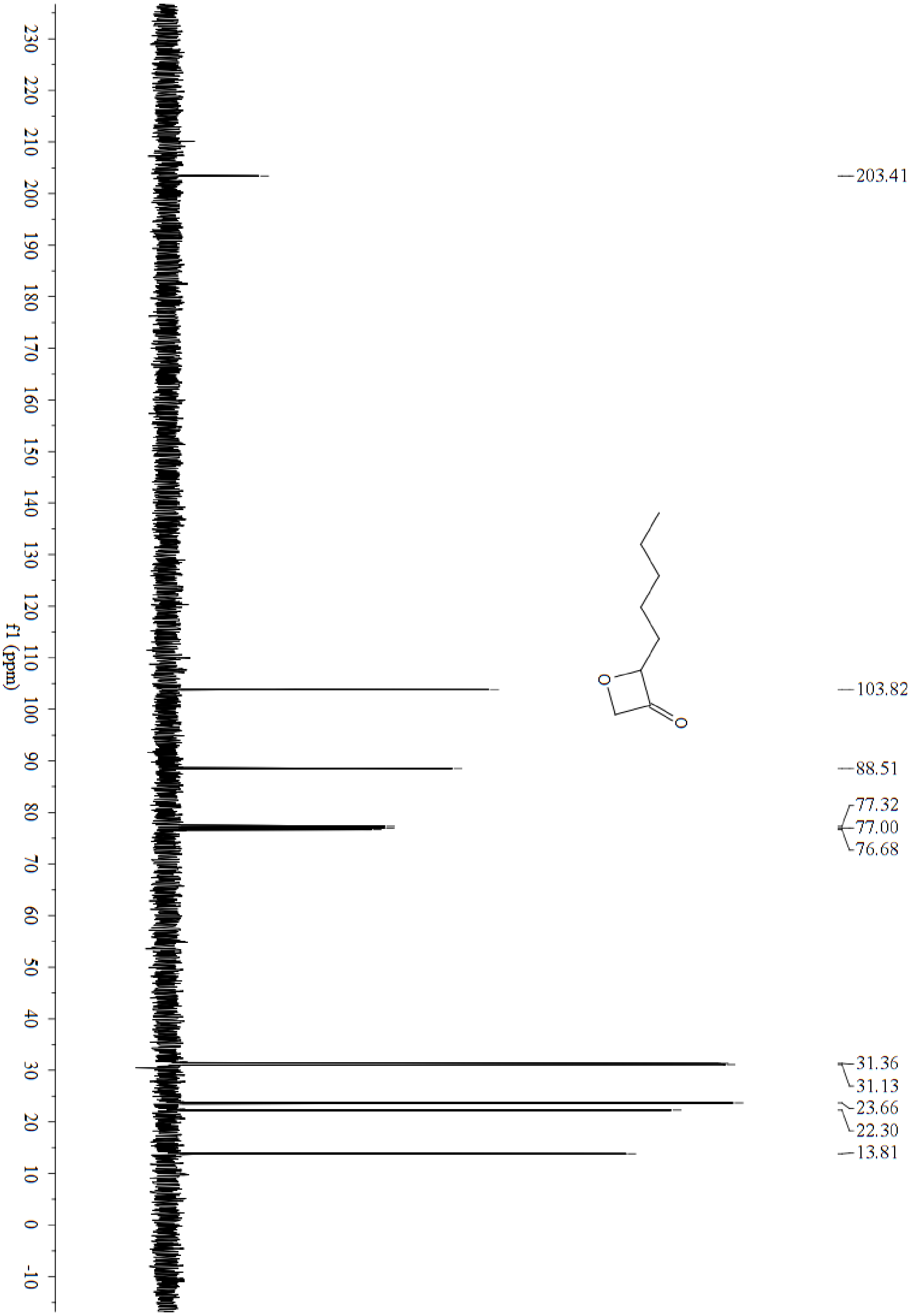

^{1}H NMR (400 MHz, $CDCl_3$) 3-(4-butylphenyl)oxetan-3-ol (1c)

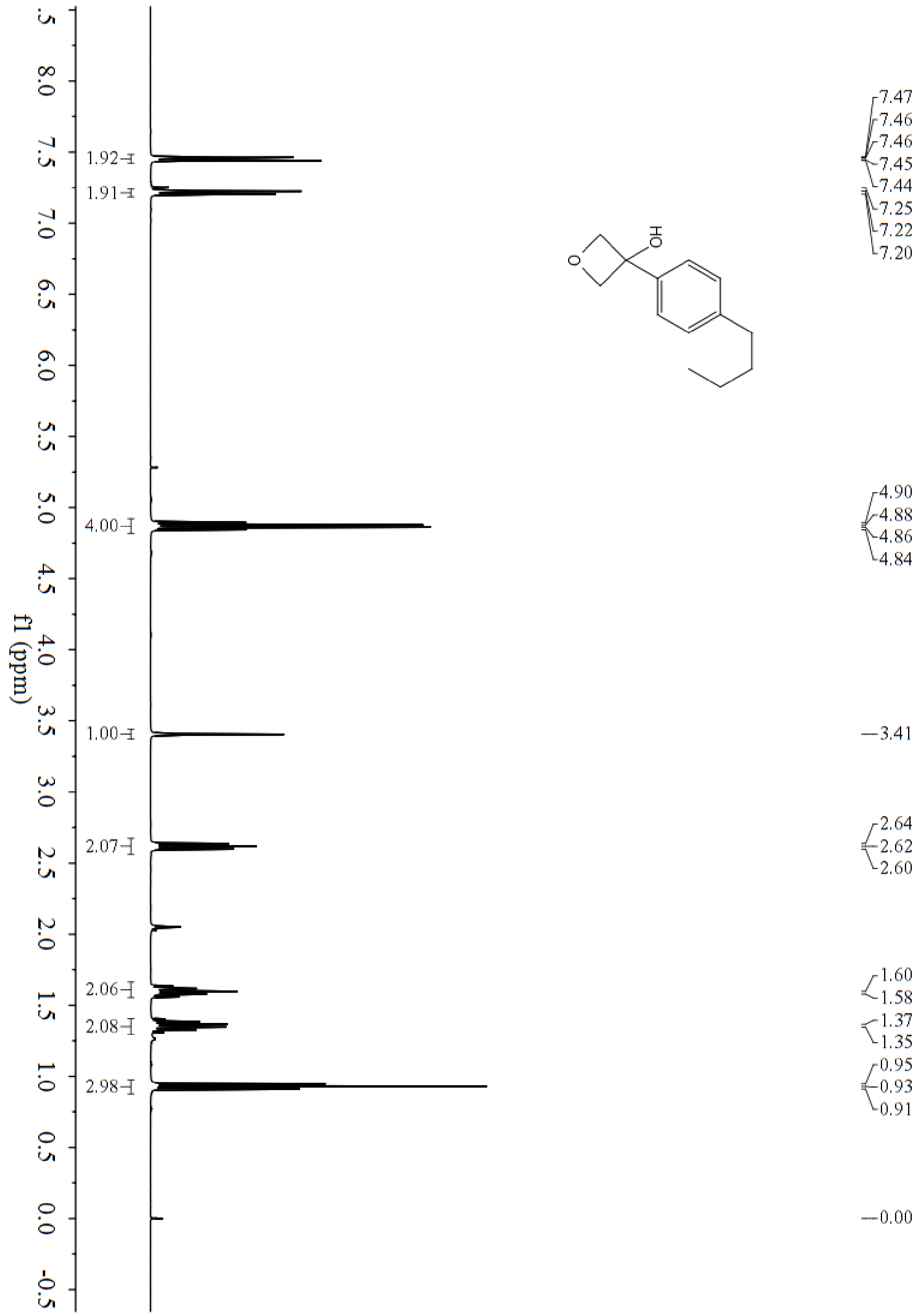

^{13}C NMR (100 MHz, $CDCl_3$) 3-(4-butylphenyl)oxetan-3-ol (1c)

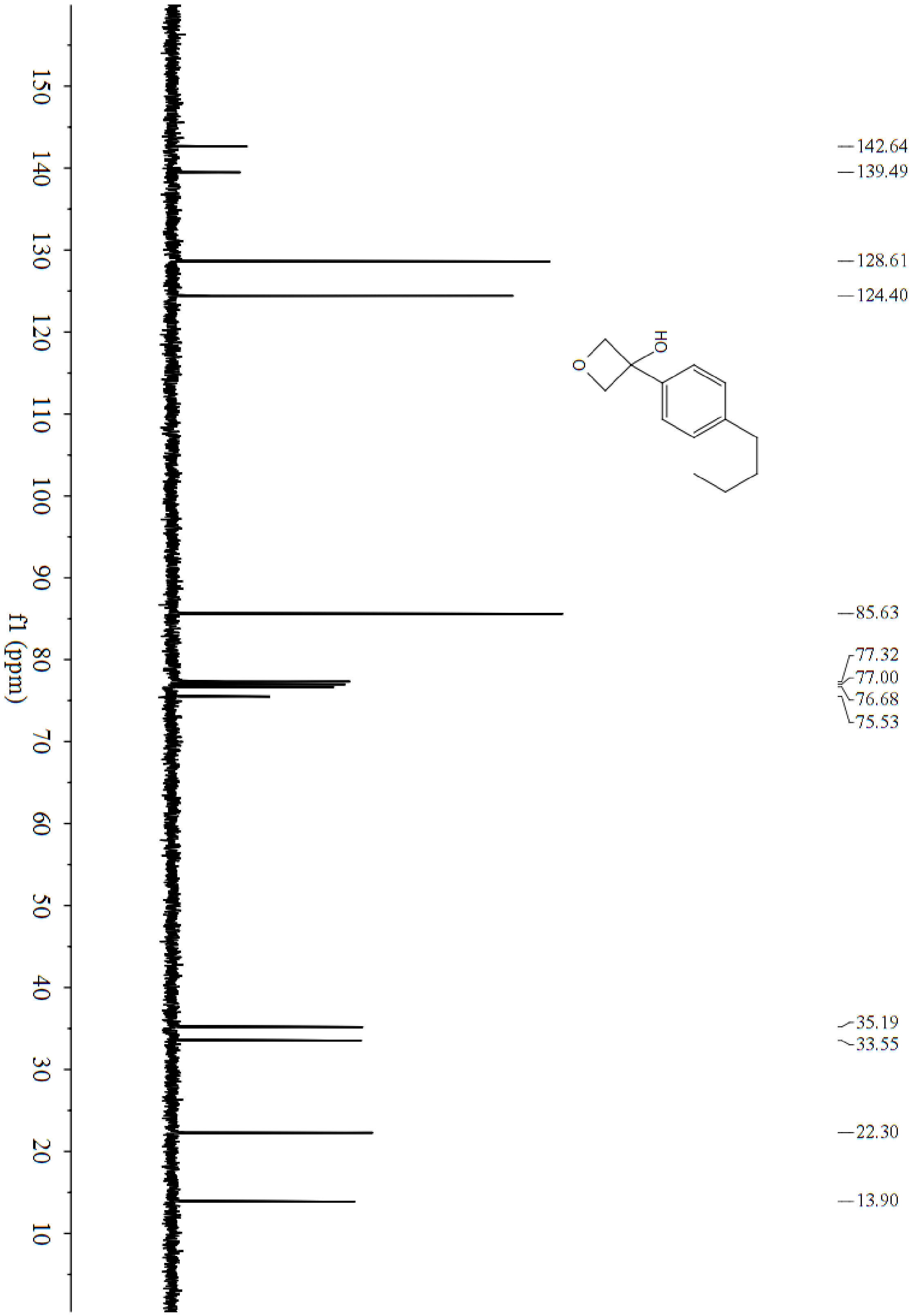

^{1}H NMR (400MHz,$CDCl_3$)   2-phenethyl-3-phenyloxetan-3-ol (3a)

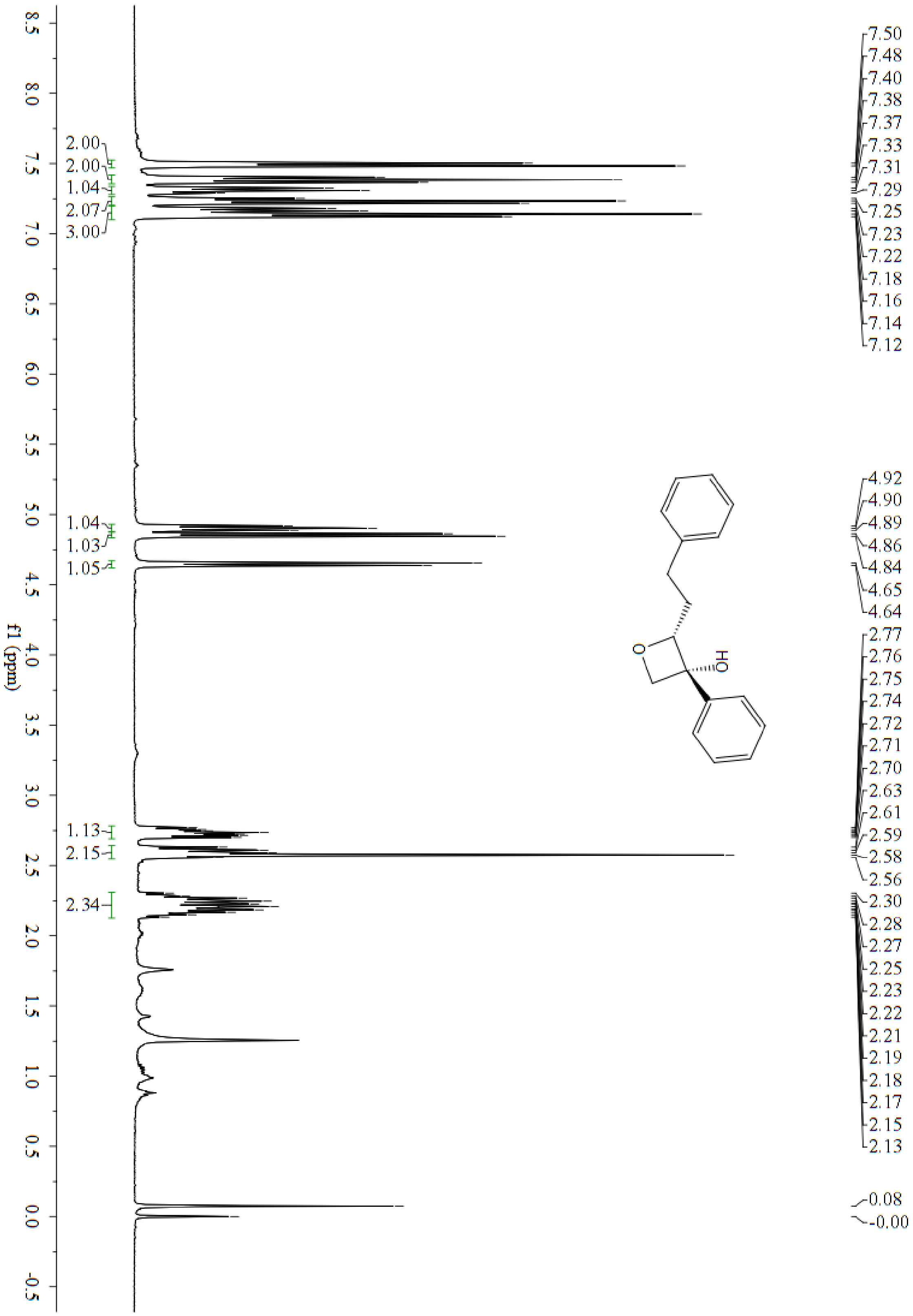

^{13}C NMR (101MHz,CDCl$_3$)   2-phenethyl-3-phenyloxetan-3-ol (3a)

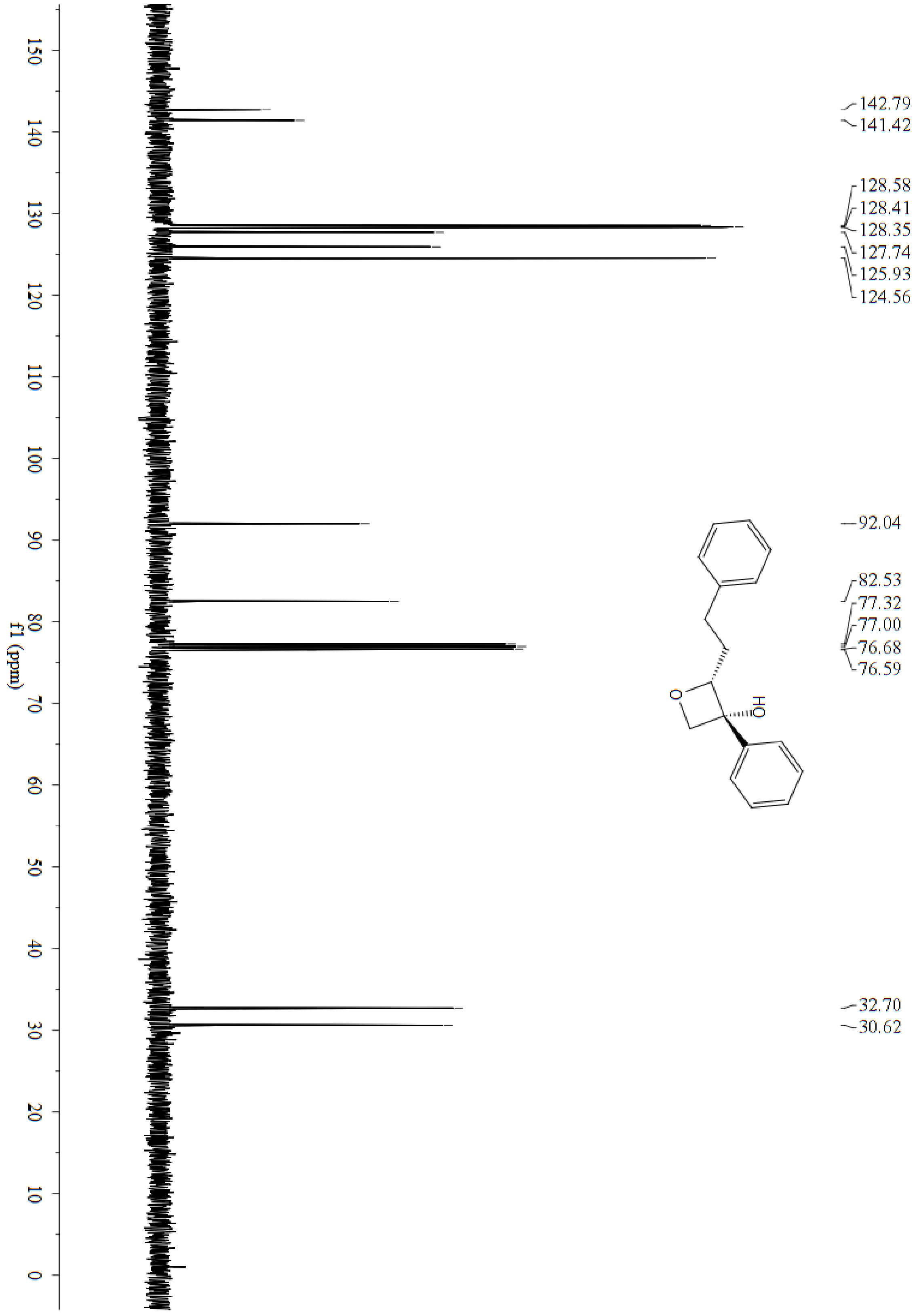

¹H NMR (400MHz,CDCl$_3$) 2-hexyl-3-phenyloxetan-3-ol (3b)

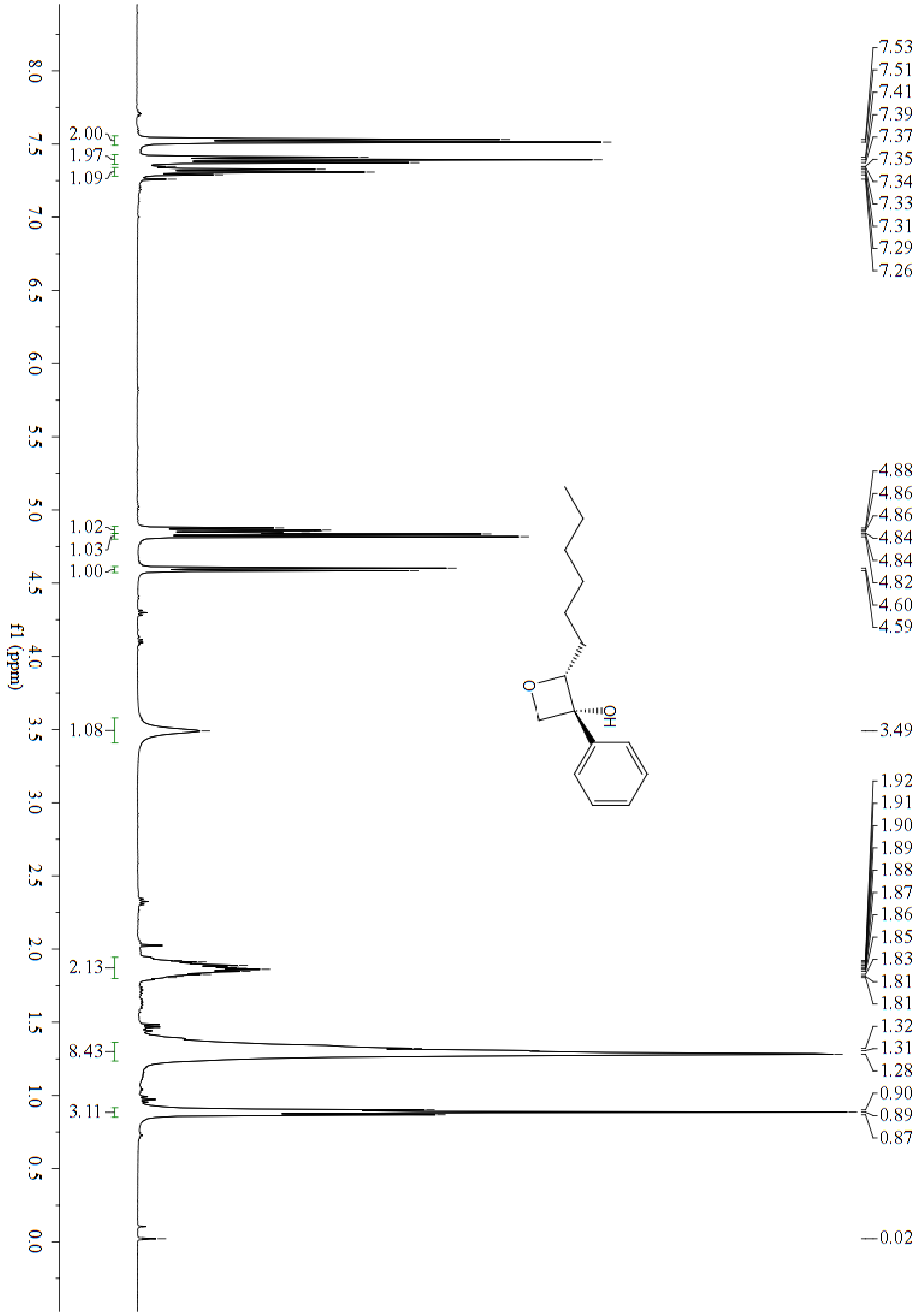

^{13}C NMR (101MHz,CDCl$_3$) 2-hexyl-3-phenyloxetan-3-ol (3b)

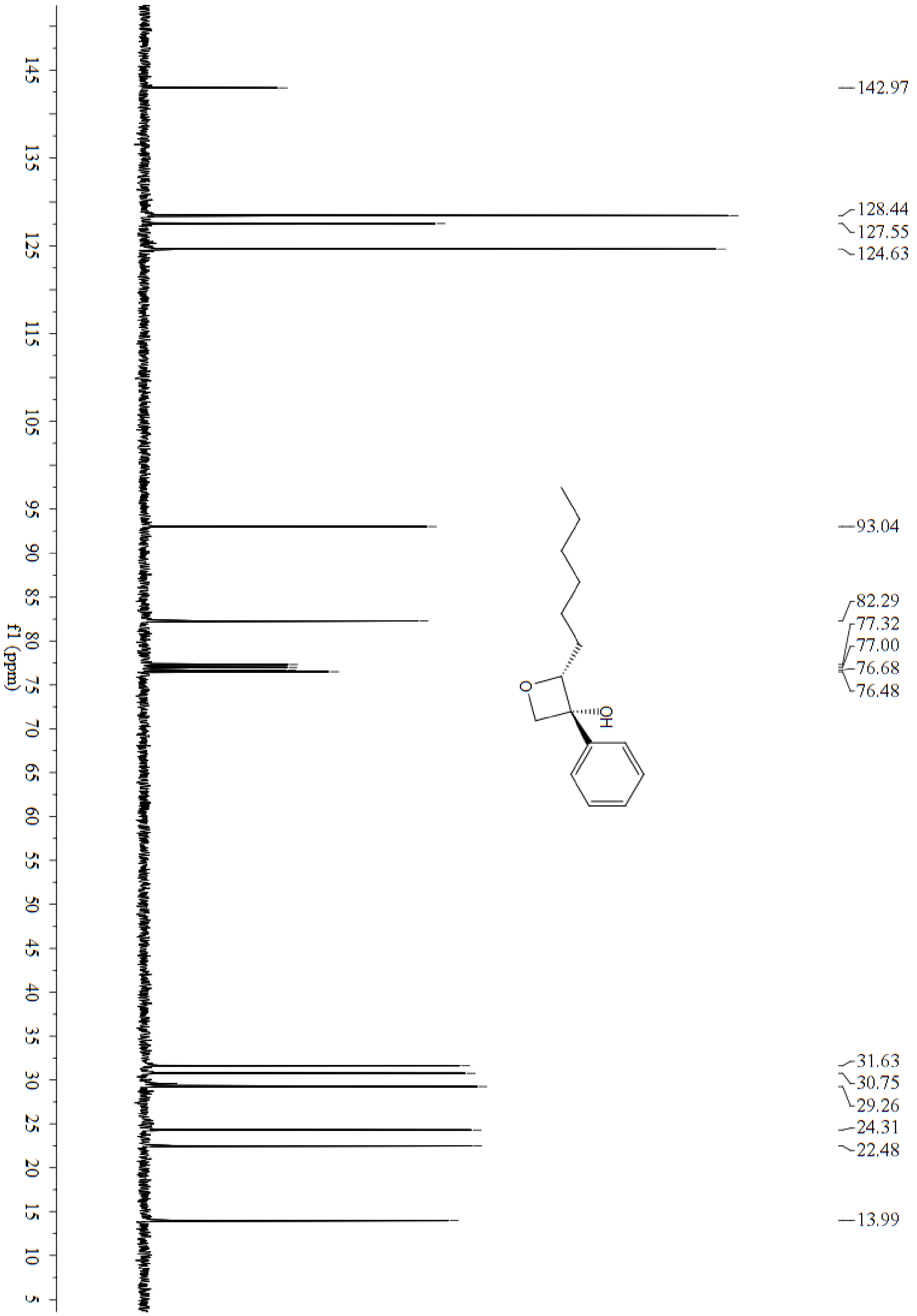

^{1}HNMR (400MHz,$CDCl_3$) 2-(4-chlorobutyl)-3-phenyloxetan-3-ol (3c)

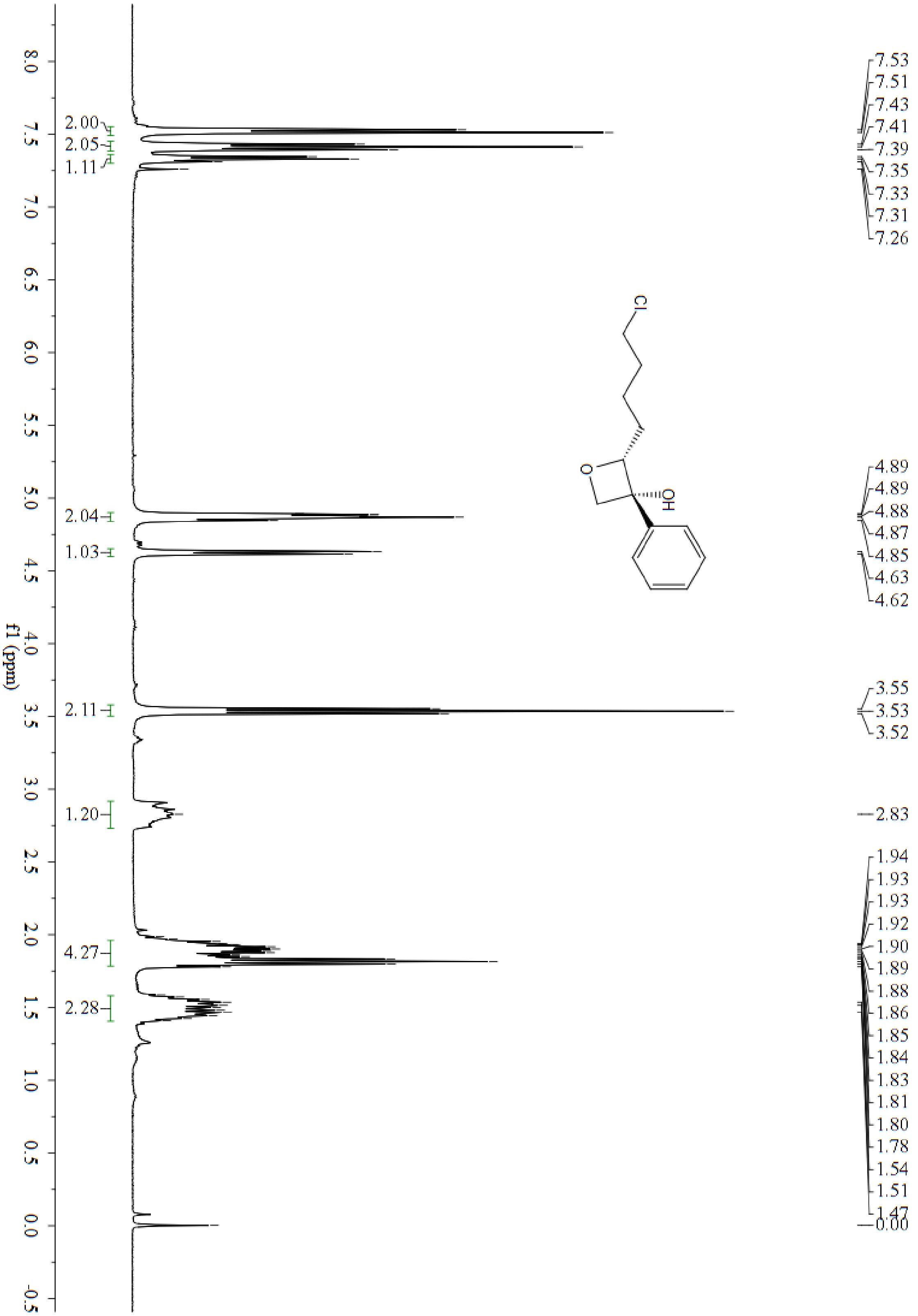

^{13}C NMR (101 MHz, $CDCl_3$)   2-(4-chlorobutyl)-3-phenyloxetan-3-ol (3c)

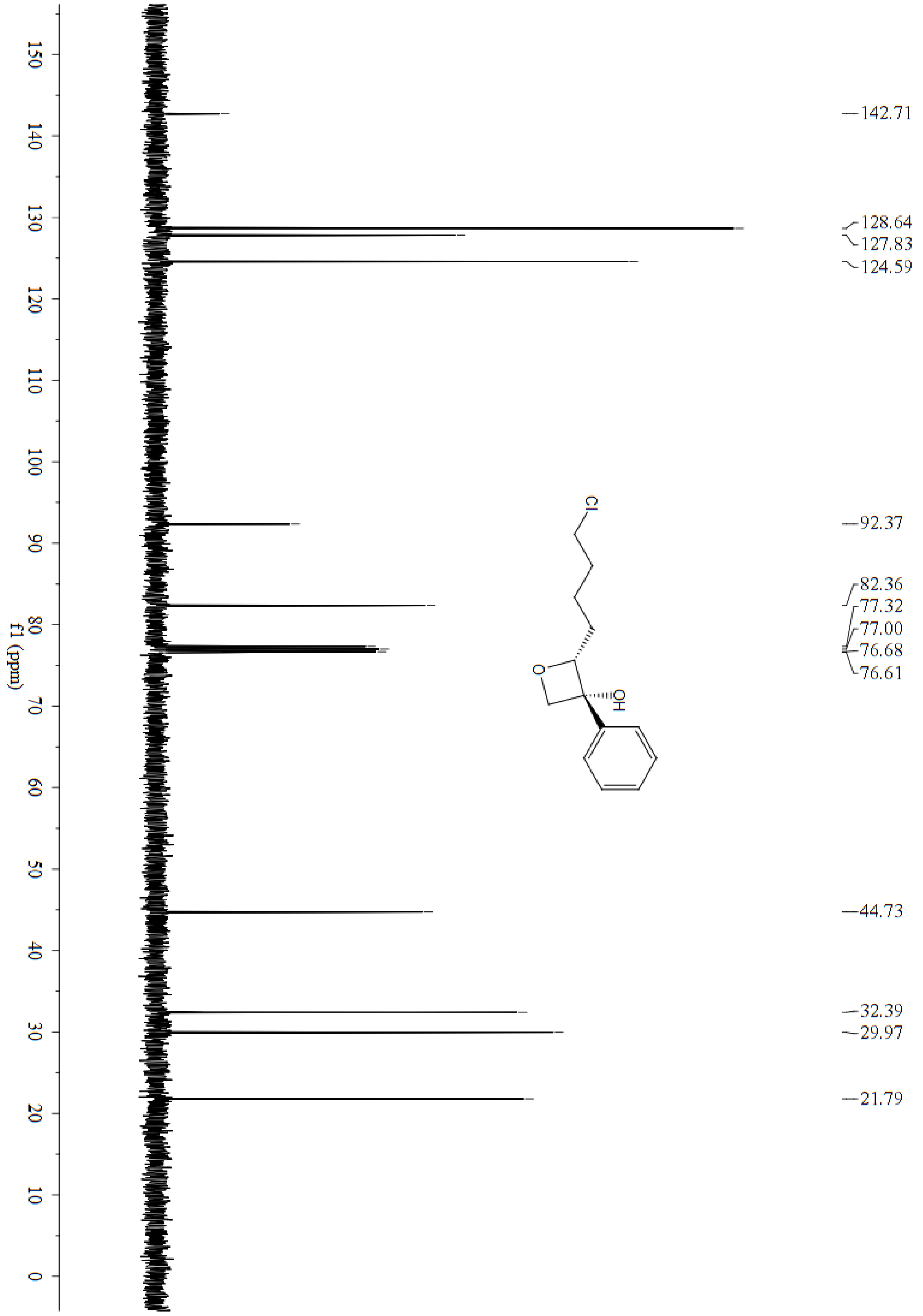

^{1}HNMR (400MHz,$CDCl_3$) 2-(but-3-en-1-yl)-3-phenyloxetan-3-ol (3d)

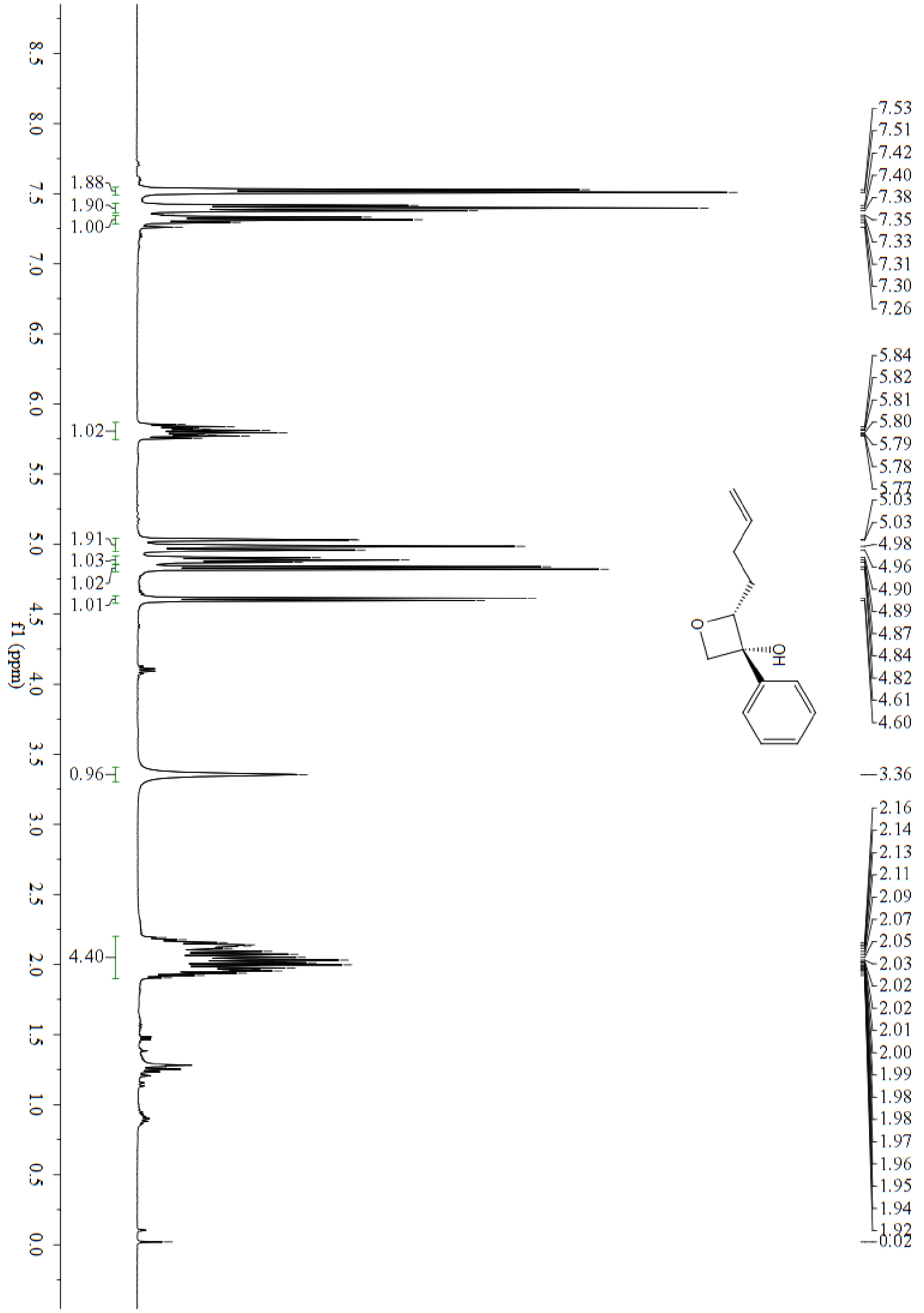

^{13}C NMR (101 MHz, $CDCl_3$) 2-(but-3-en-1-yl)-3-phenyloxetan-3-ol (3d)

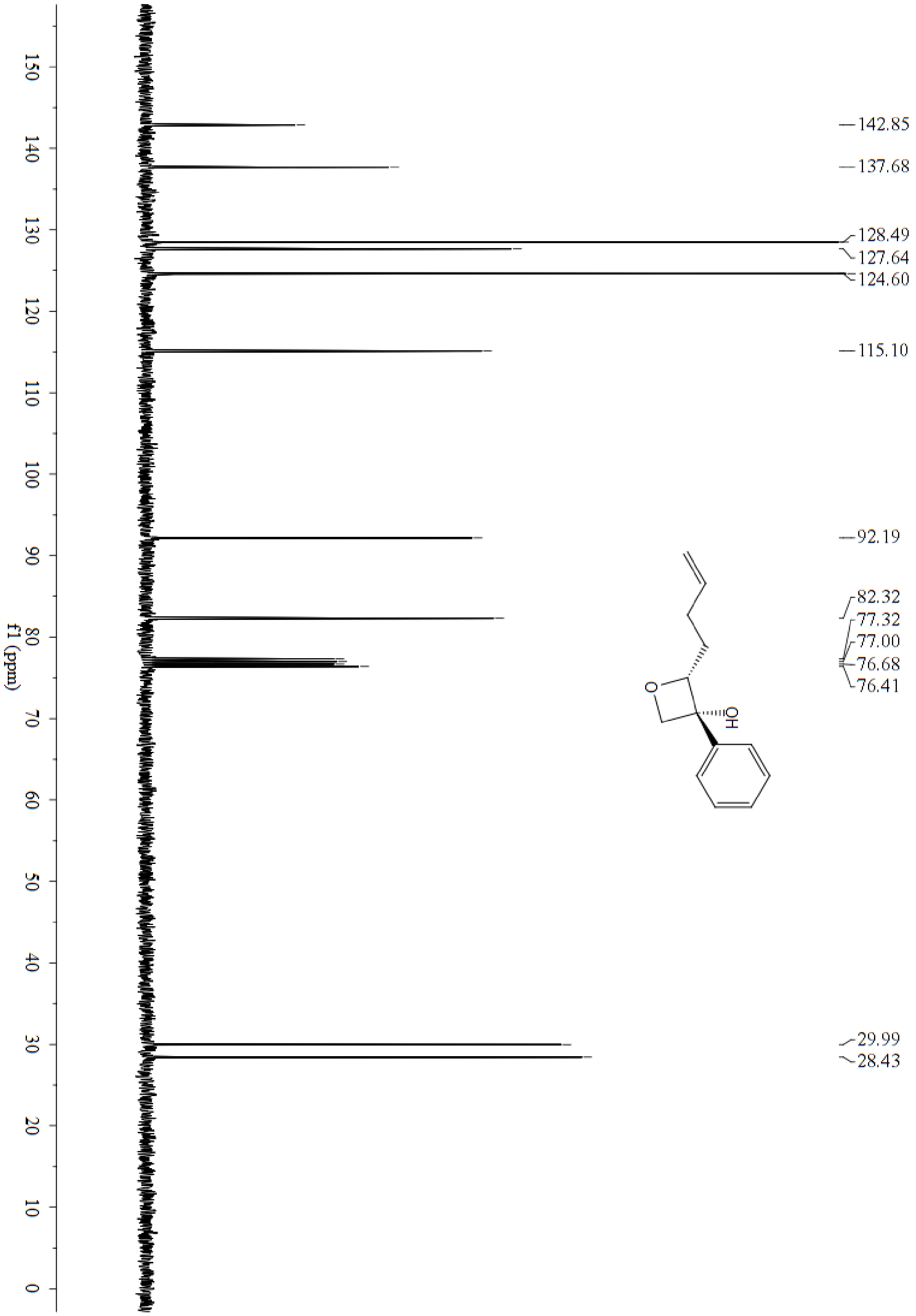

^{1}HNMR (400MHz,$CDCl_3$) 2-cyclopropyl-3-phenyloxetan-3-ol (3e)

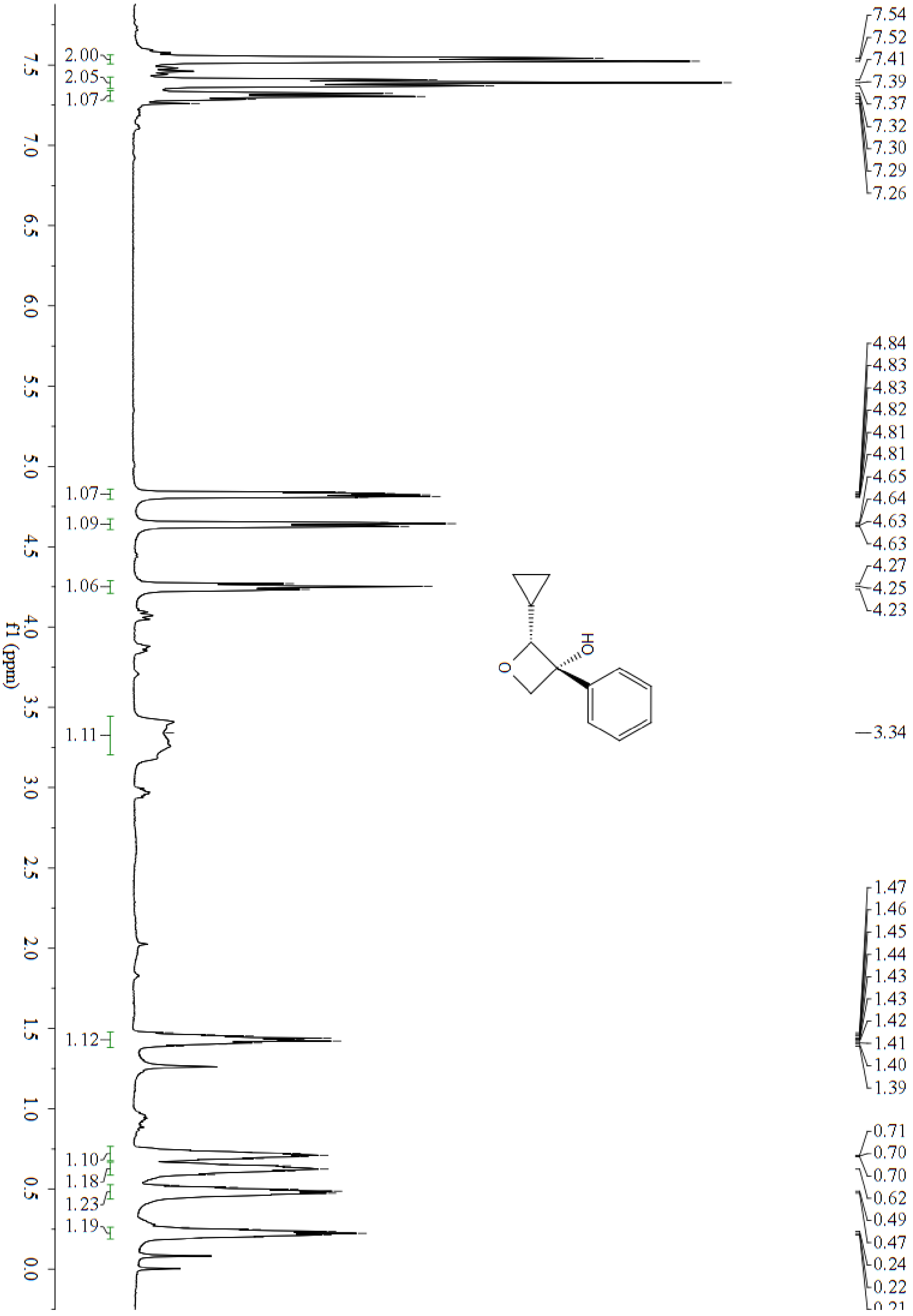

^{13}C NMR (101 MHz, $CDCl_3$) 2-cyclopropyl-3-phenyloxetan-3-ol (3e)

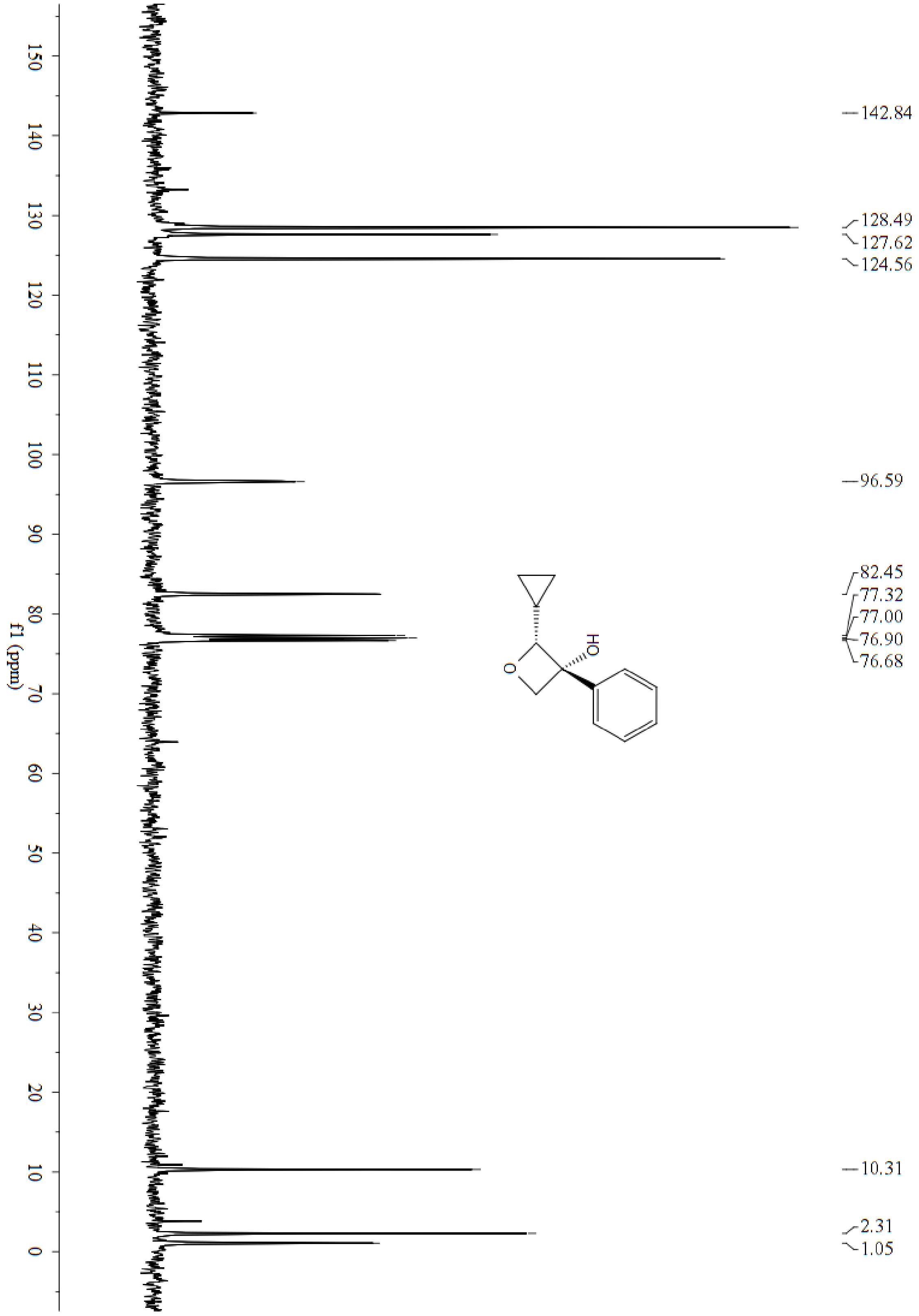

^{1}HNMR (400MHz,$CDCl_3$) 2-cyclohexyl-3-phenyloxetan-3-ol (3f)

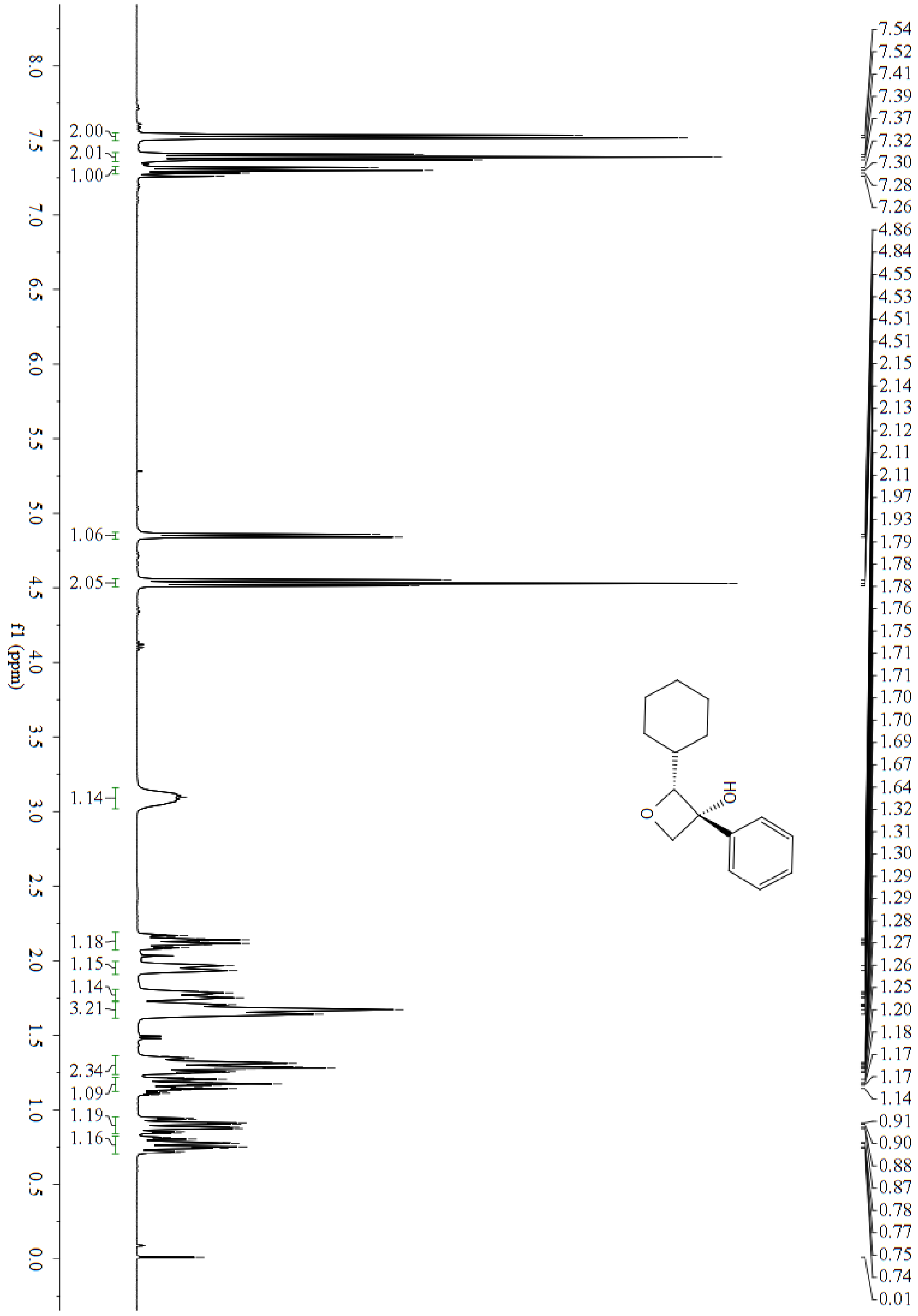

^{13}C NMR (101 MHz, $CDCl_3$) 2-cyclohexyl-3-phenyloxetan-3-ol (3f)

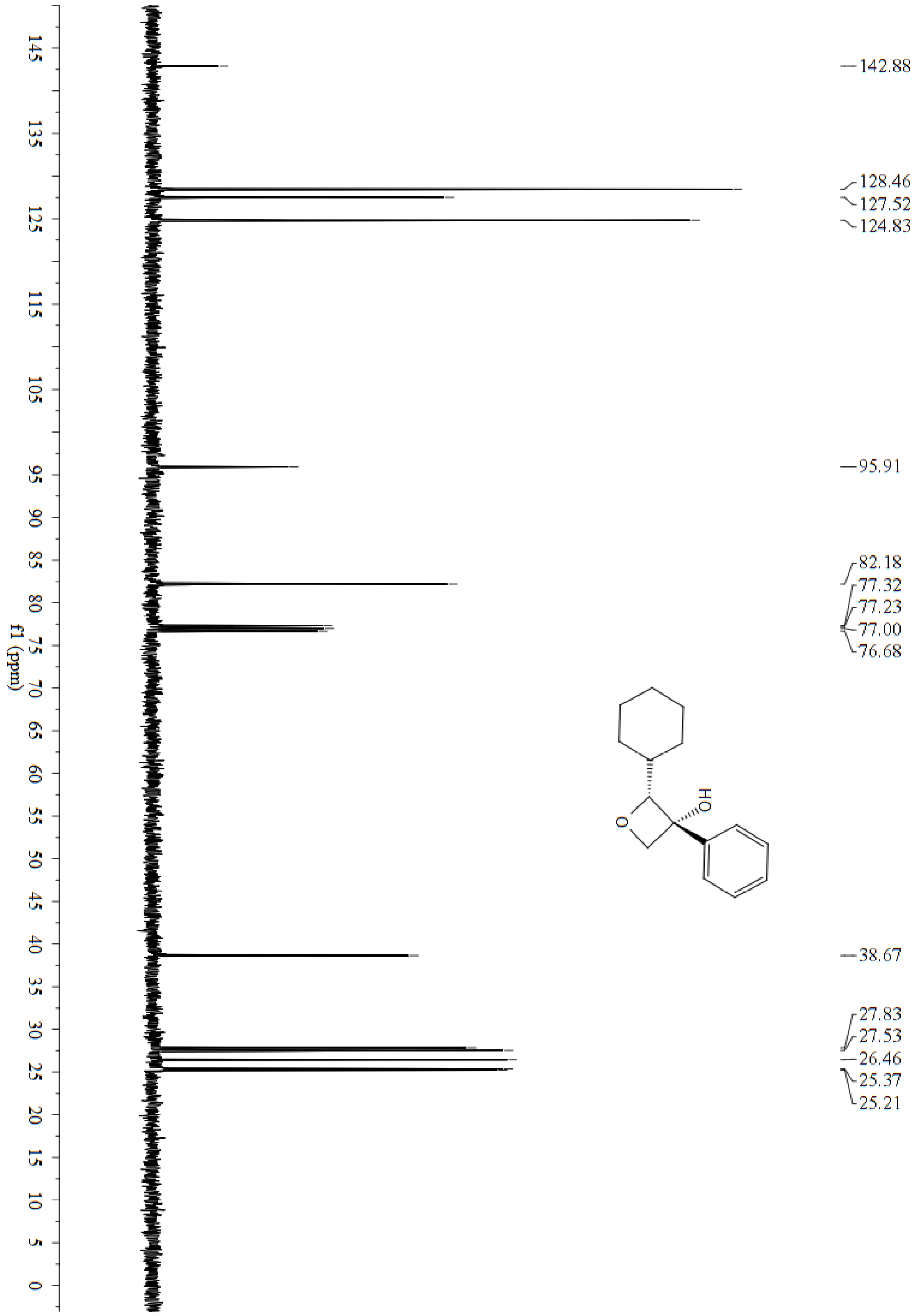

^{1}HNMR (400MHz,$CDCl_3$) 2-(2-(methylthio)ethyl)-3-phenyloxetan-3-ol (3g)

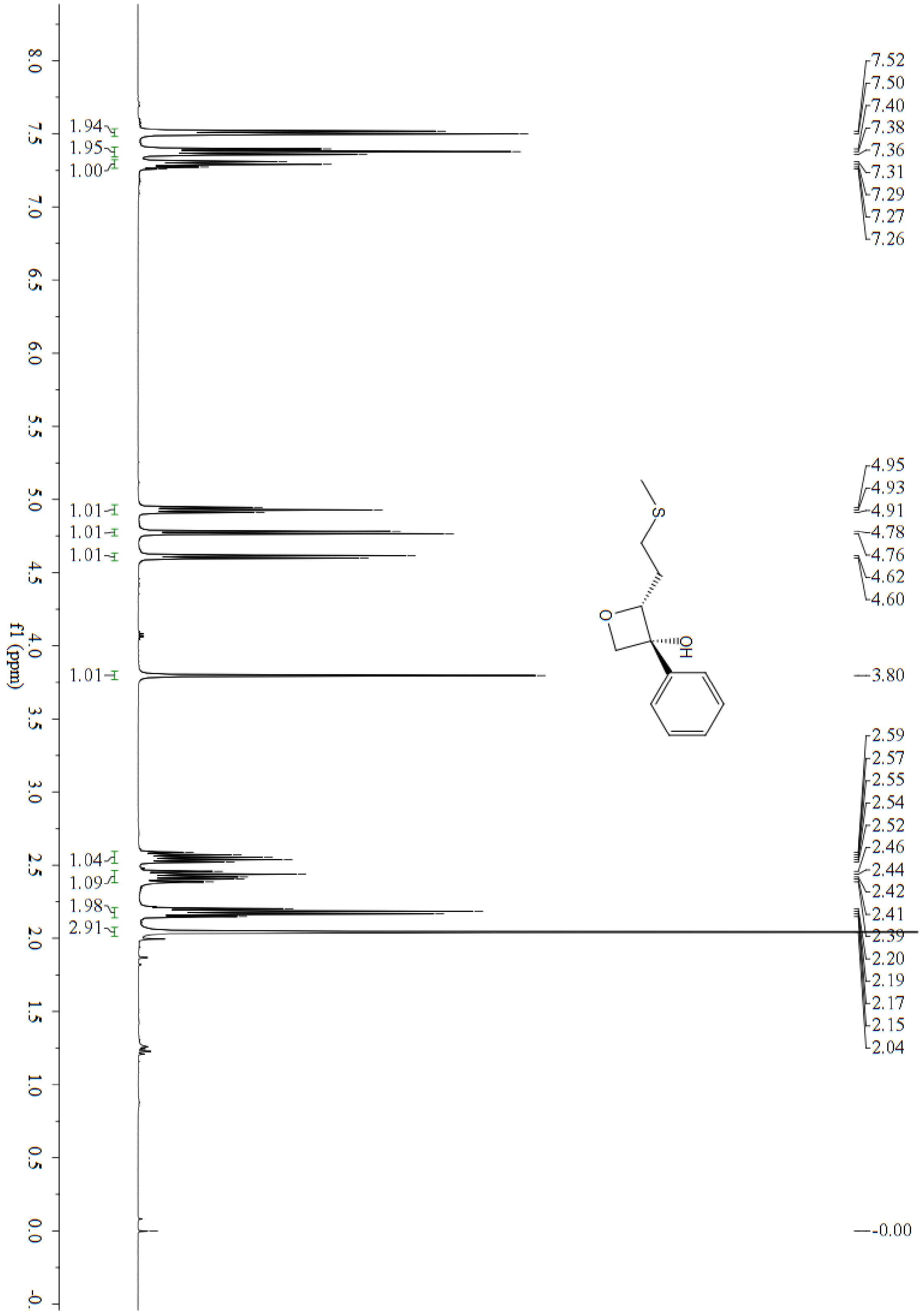

^{13}C NMR (101 MHz,$CDCl_3$) 2-(2-(methylthio)ethyl)-3-phenyloxetan-3-ol (3g)

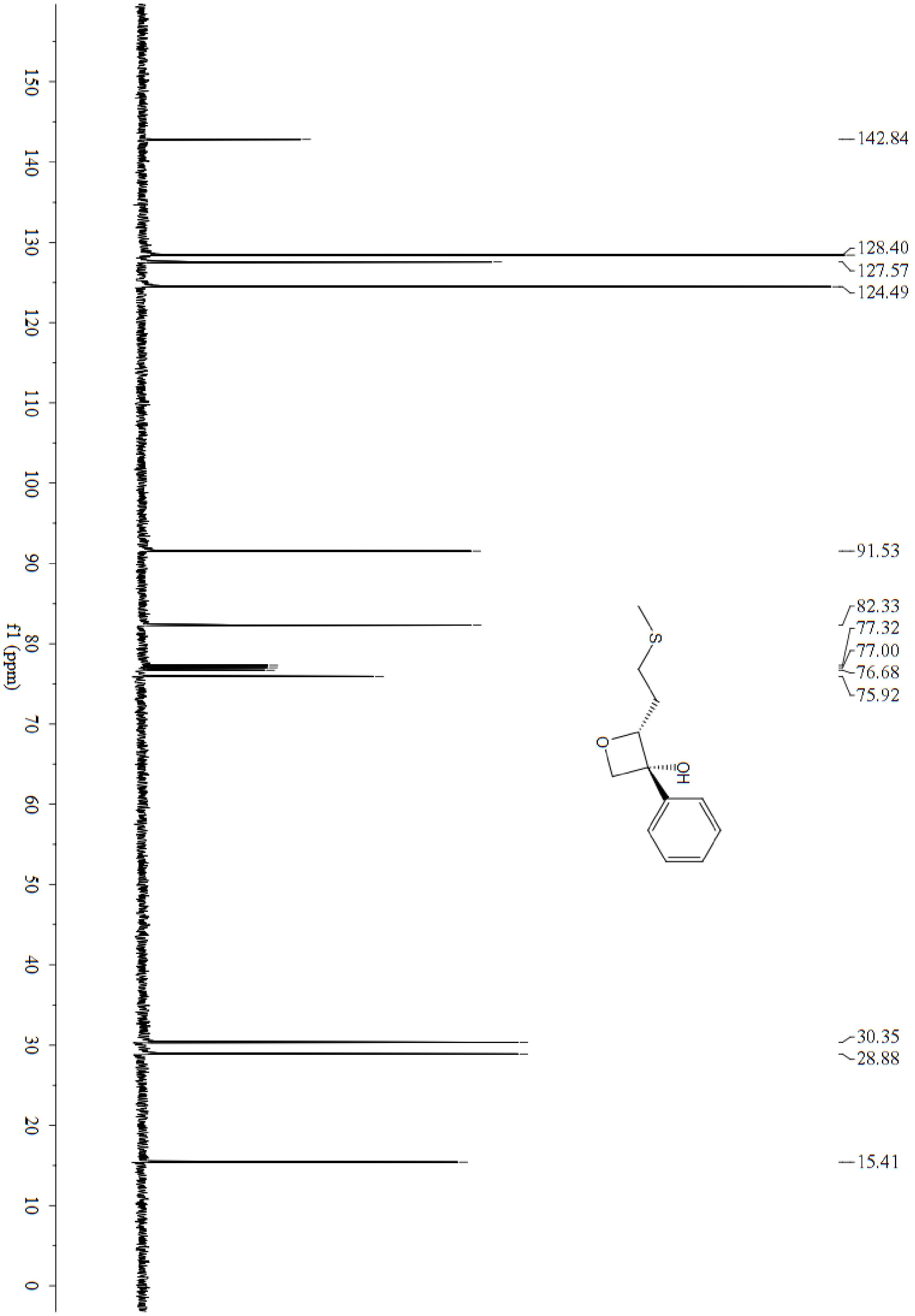

^{1}HNMR (400MHz,CDCl$_3$) 2,3-diphenyloxetan-3-ol (3h)

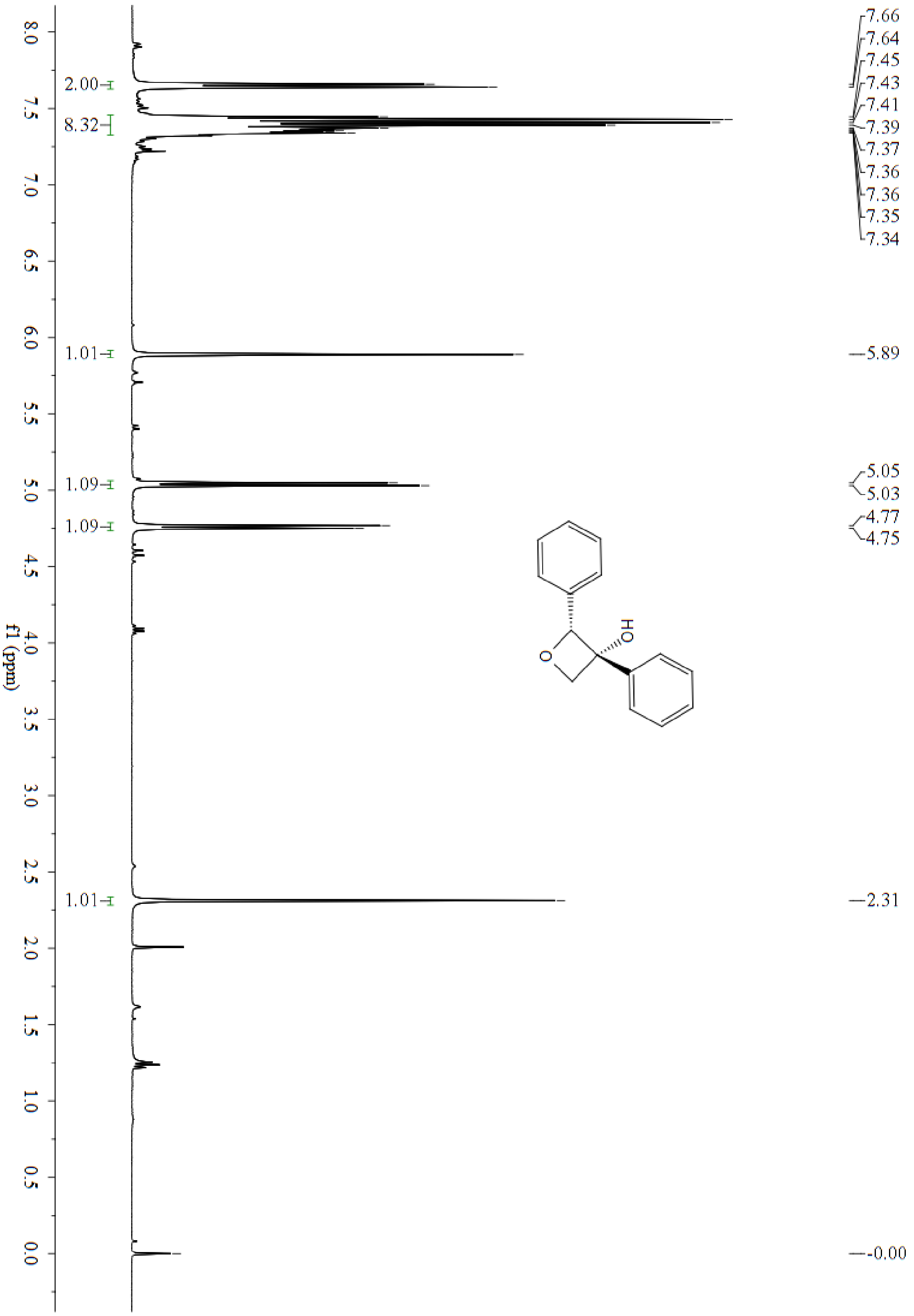

^{13}C NMR (101 MHz,CDCl$_3$) 2,3-diphenyloxetan-3-ol (3h)

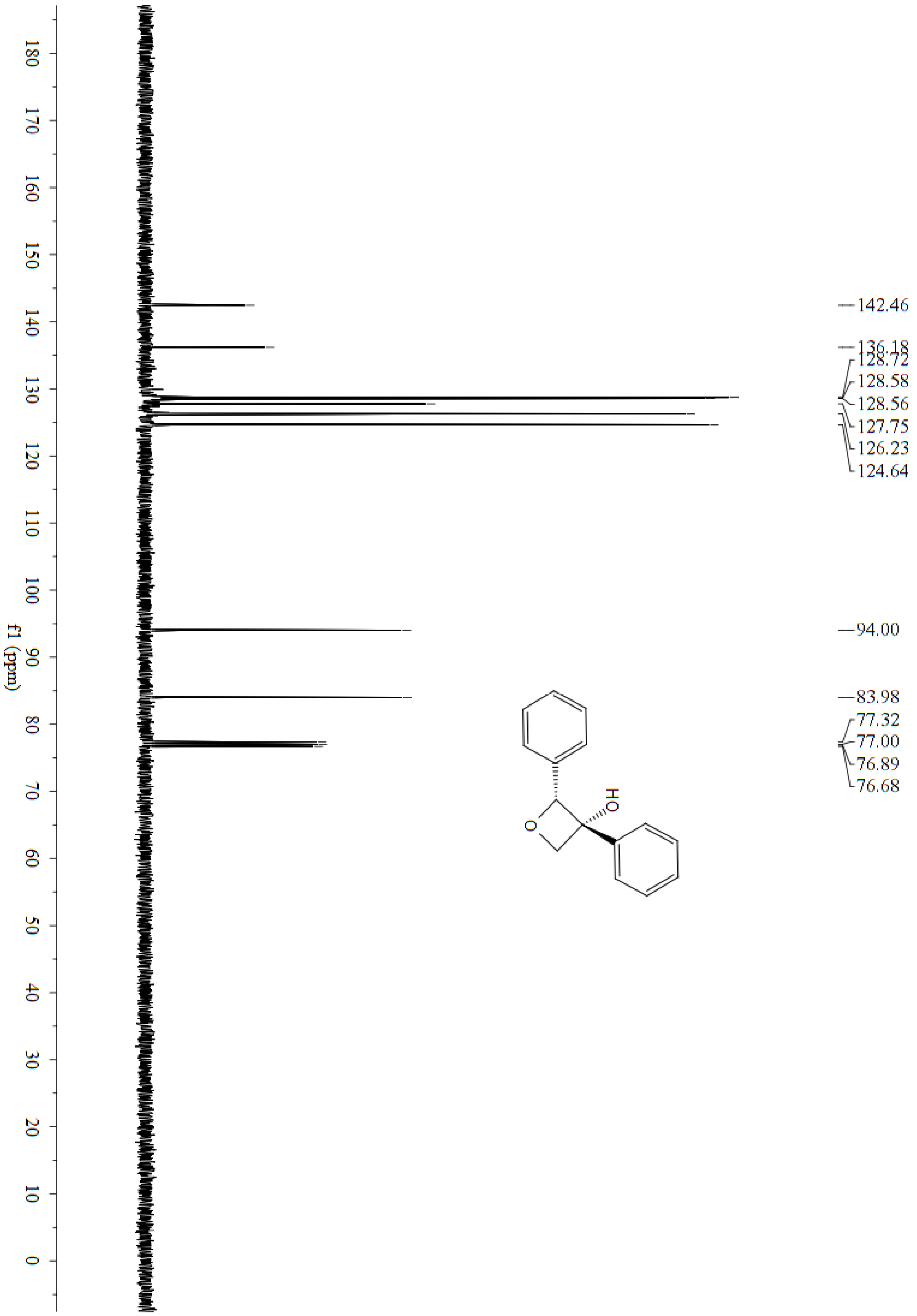

^{1}HNMR (400MHz,CDCl$_3$) 3-phenyl-2-(2-(trifluoromethyl)phenyl)oxetan-3-ol (3i)

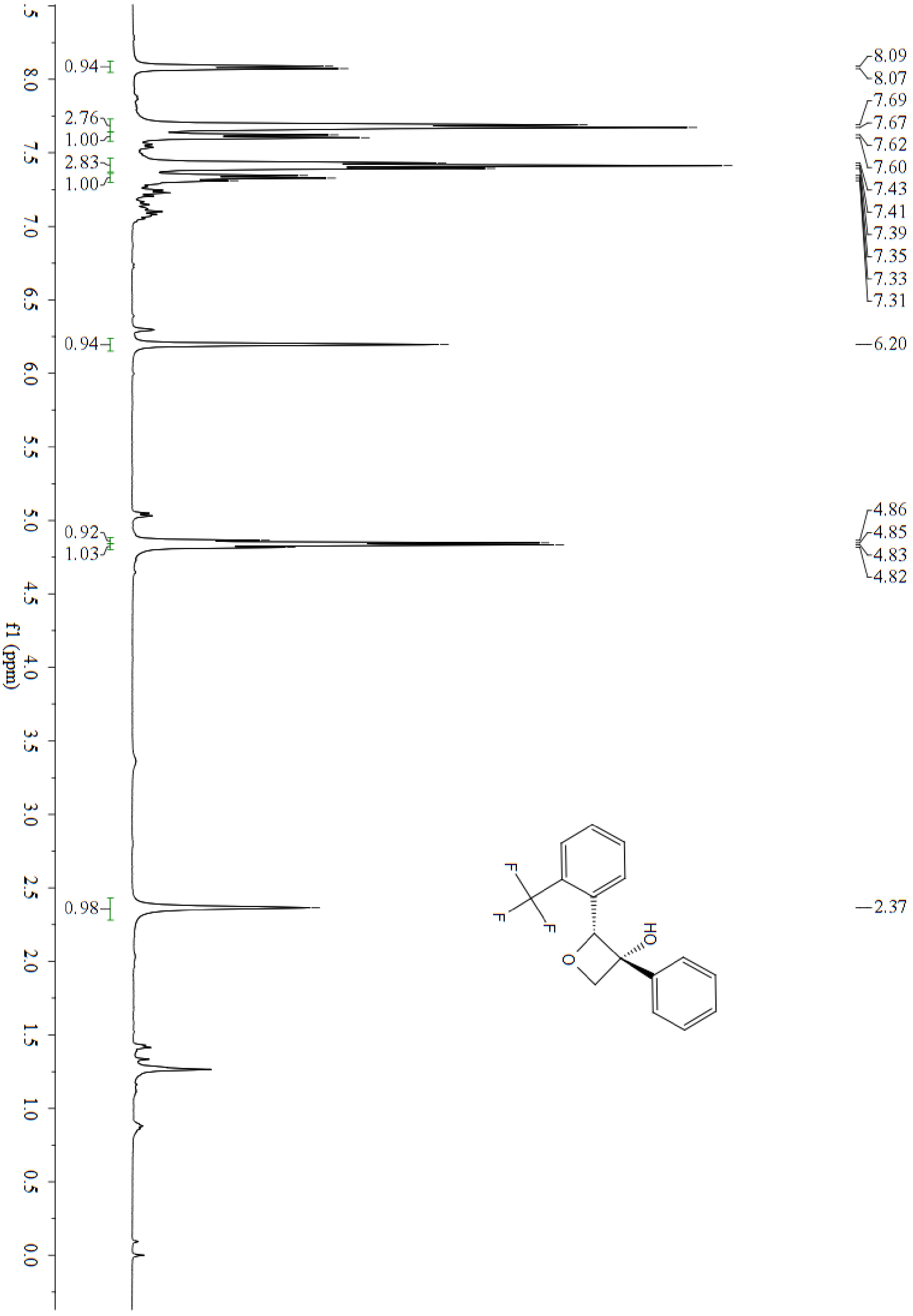

^{13}CNMR(101MHz,CDCl$_3$) 3-phenyl-2-(2-(trifluoromethyl)phenyl)oxetan-3-ol (3i)

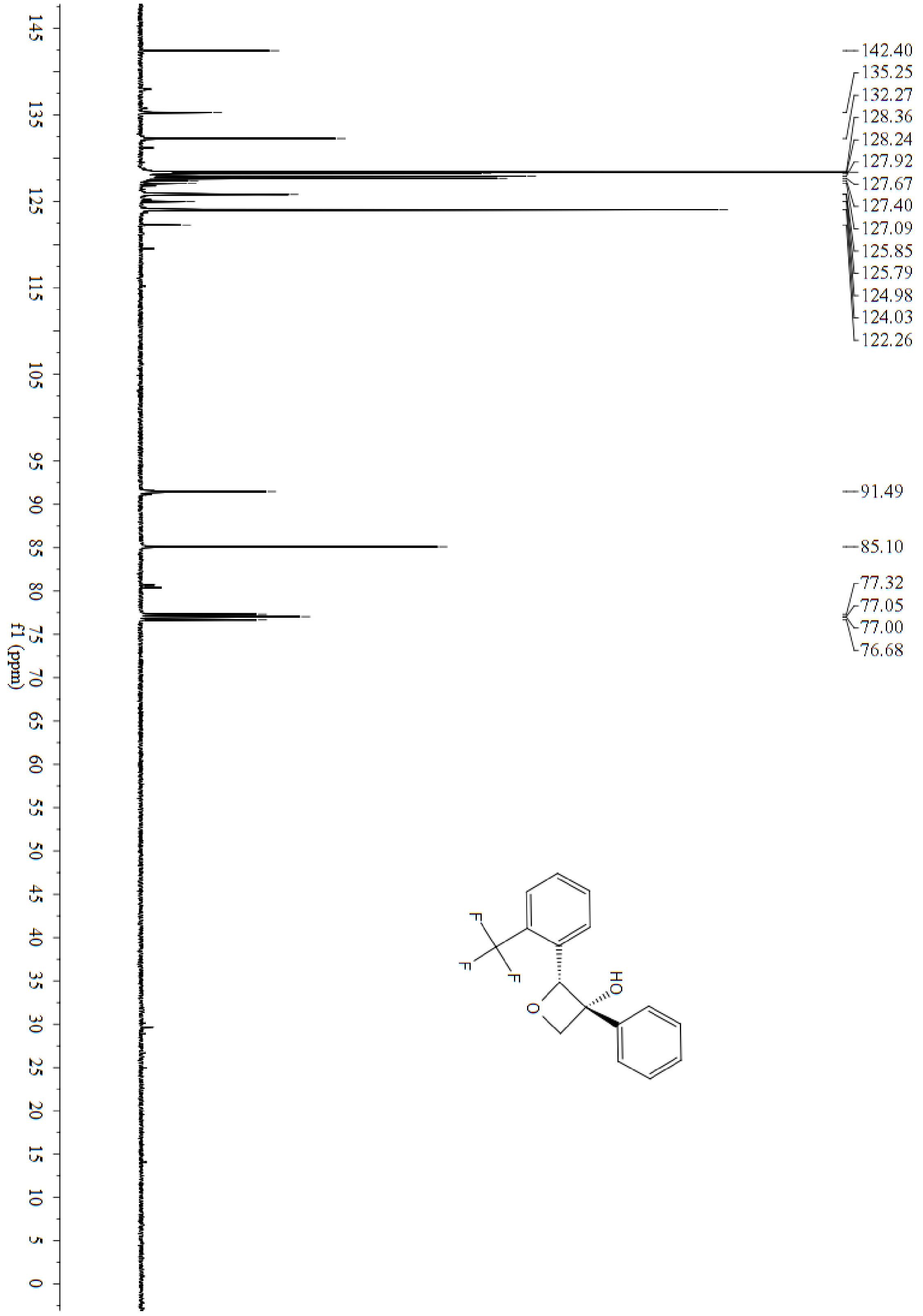

^{1}HNMR (400MHz,$CDCl_3$) 3-(naphthalen-1-yl)-2-phenethyloxetan-3-ol (3n)

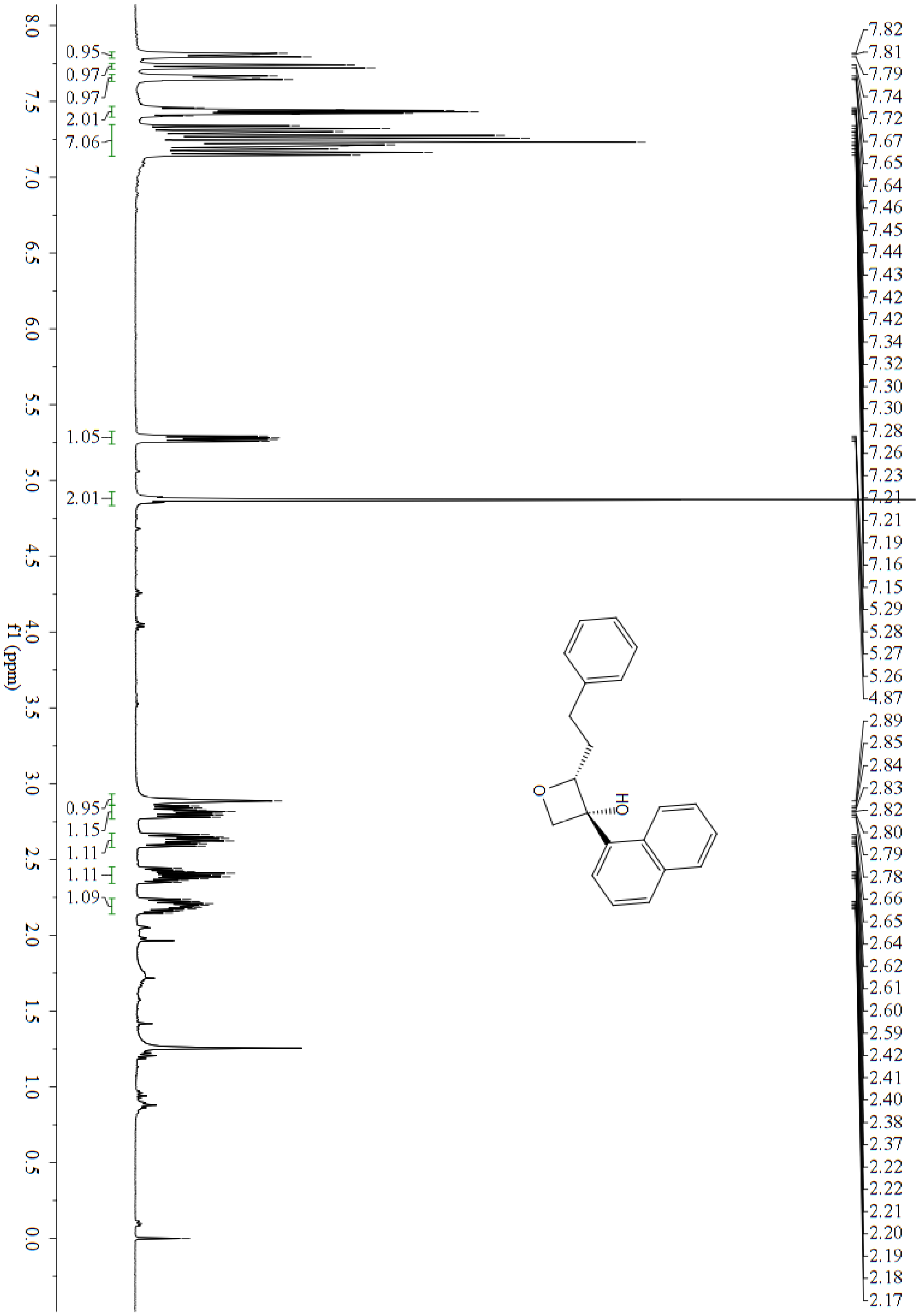

^{13}CNMR(101MHz,$CDCl_3$) 3-(naphthalen-1-yl)-2-phenethyloxetan-3-ol (3n)

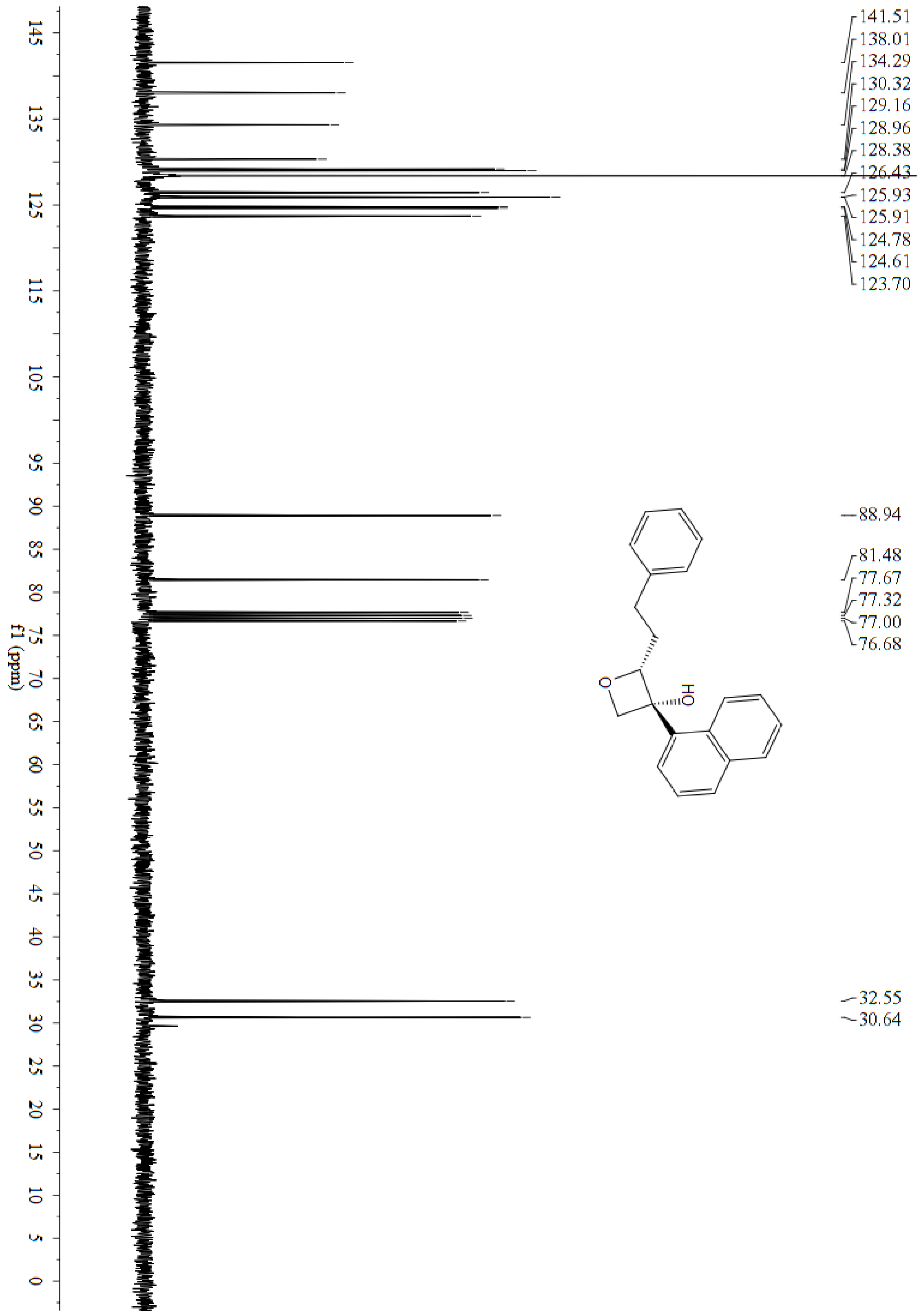

^{1}H NMR (400MHz,$CDCl_3$) 3-(4-methoxyphenyl)-2-phenethyloxetan-3-ol (3o)

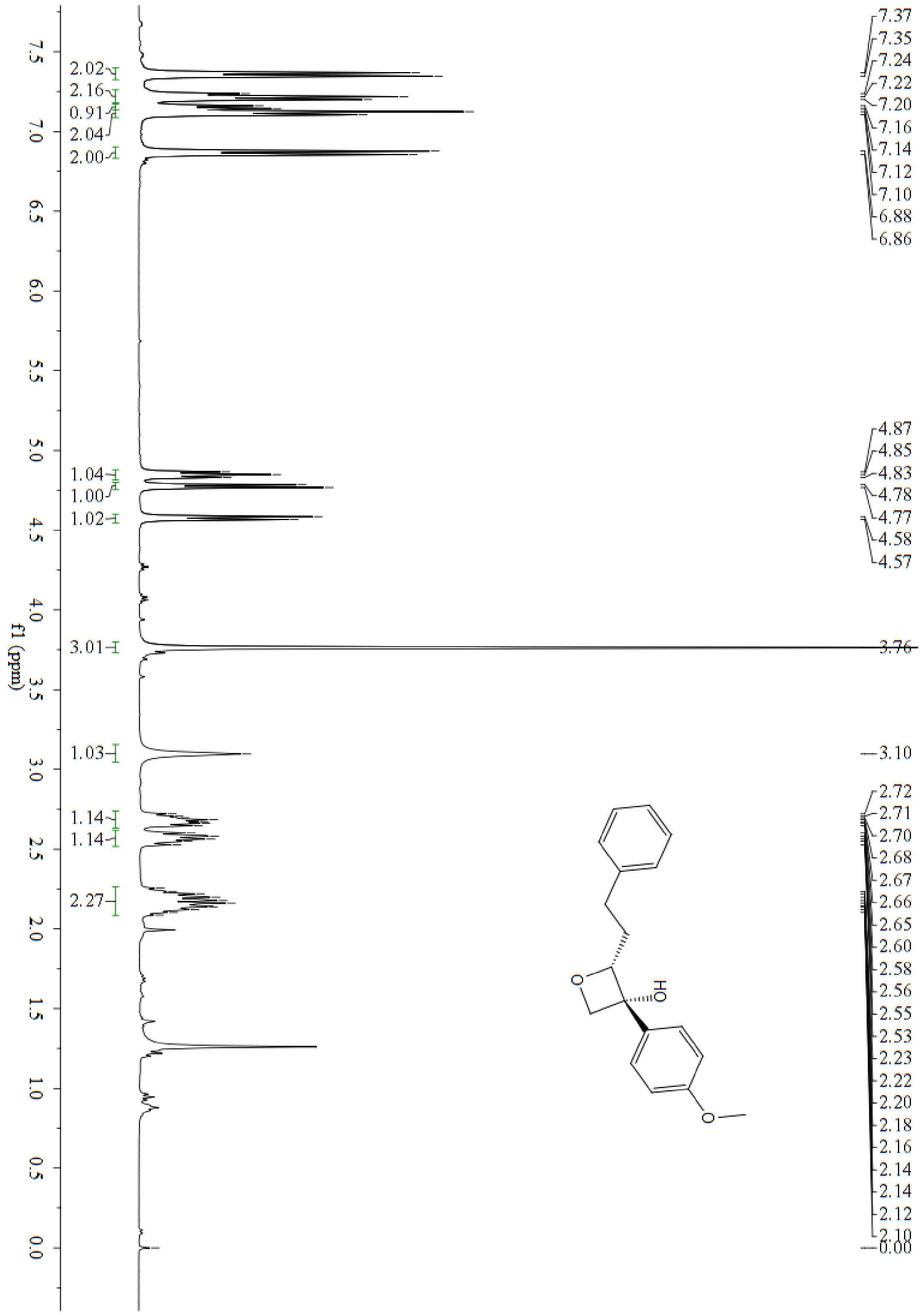

^{13}C NMR (101MHz,CDCl$_3$) 3-(4-methoxyphenyl)-2-phenethyloxetan-3-ol (3o)

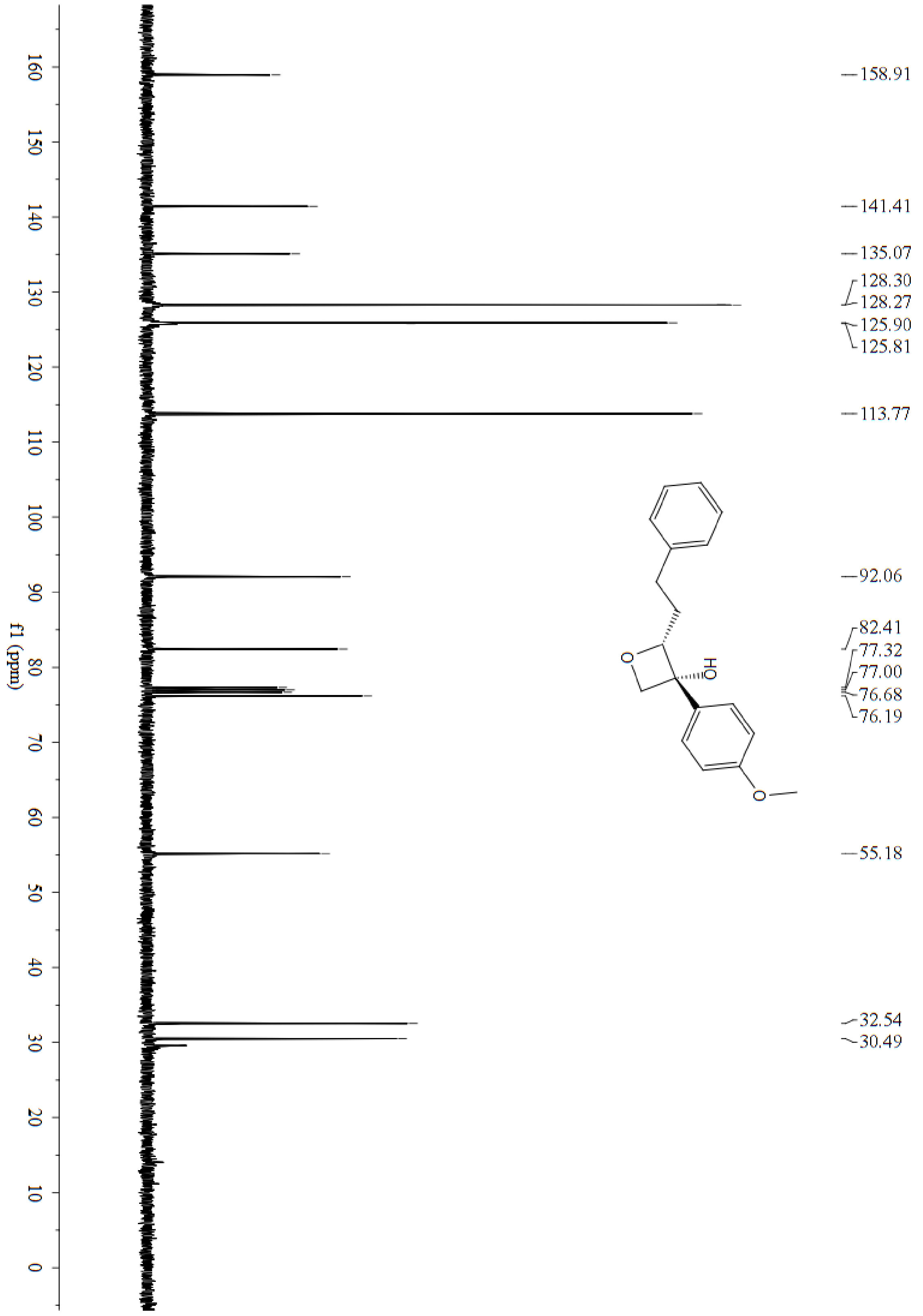

^{1}H NMR (400MHz,CDCl$_3$) 2-phenethyl-3-(o-tolyl)oxetan-3-ol (3p)

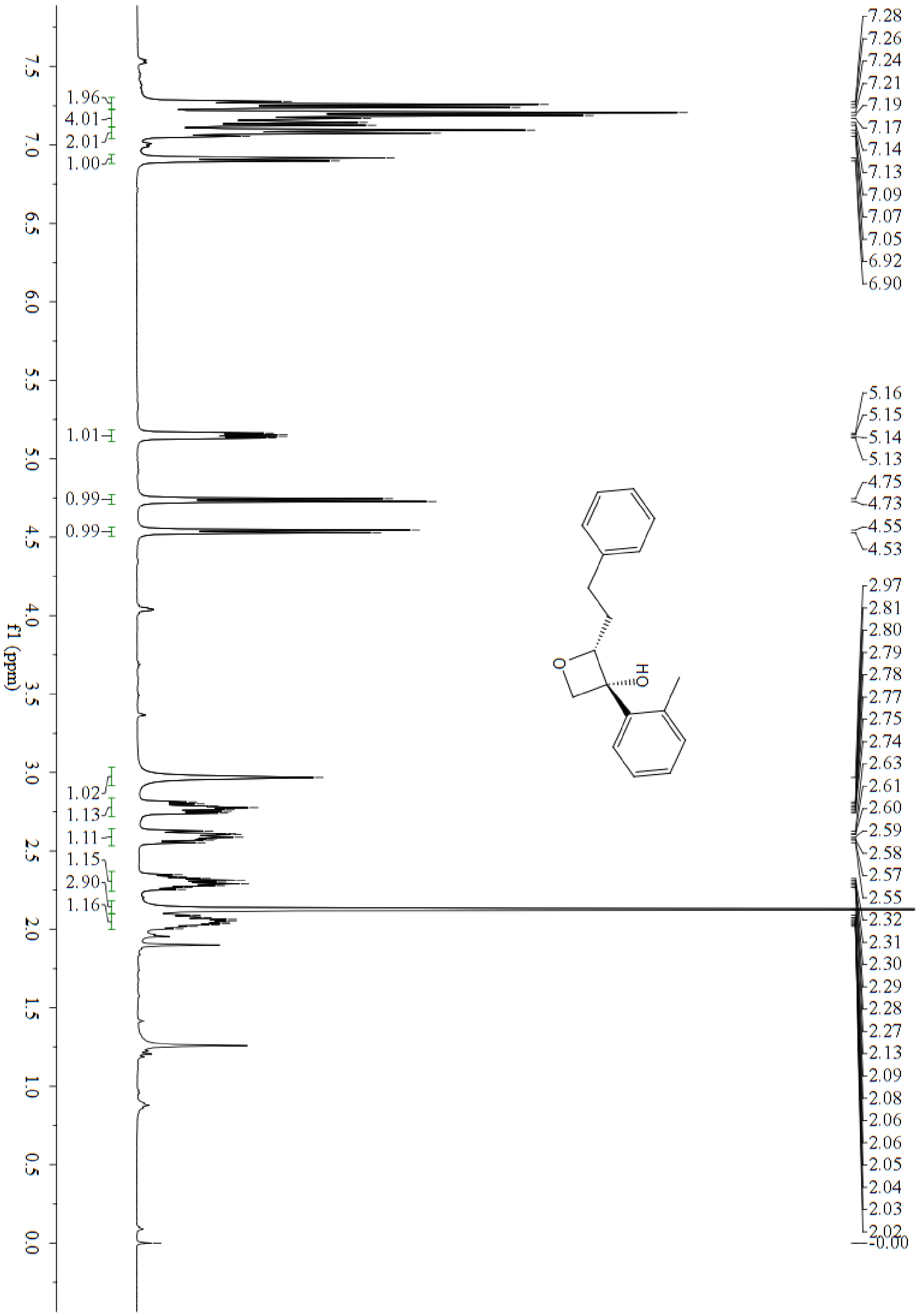

^{13}C NMR (101MHz,CDCl$_3$) 2-phenethyl-3-(o-tolyl)oxetan-3-ol (3p)

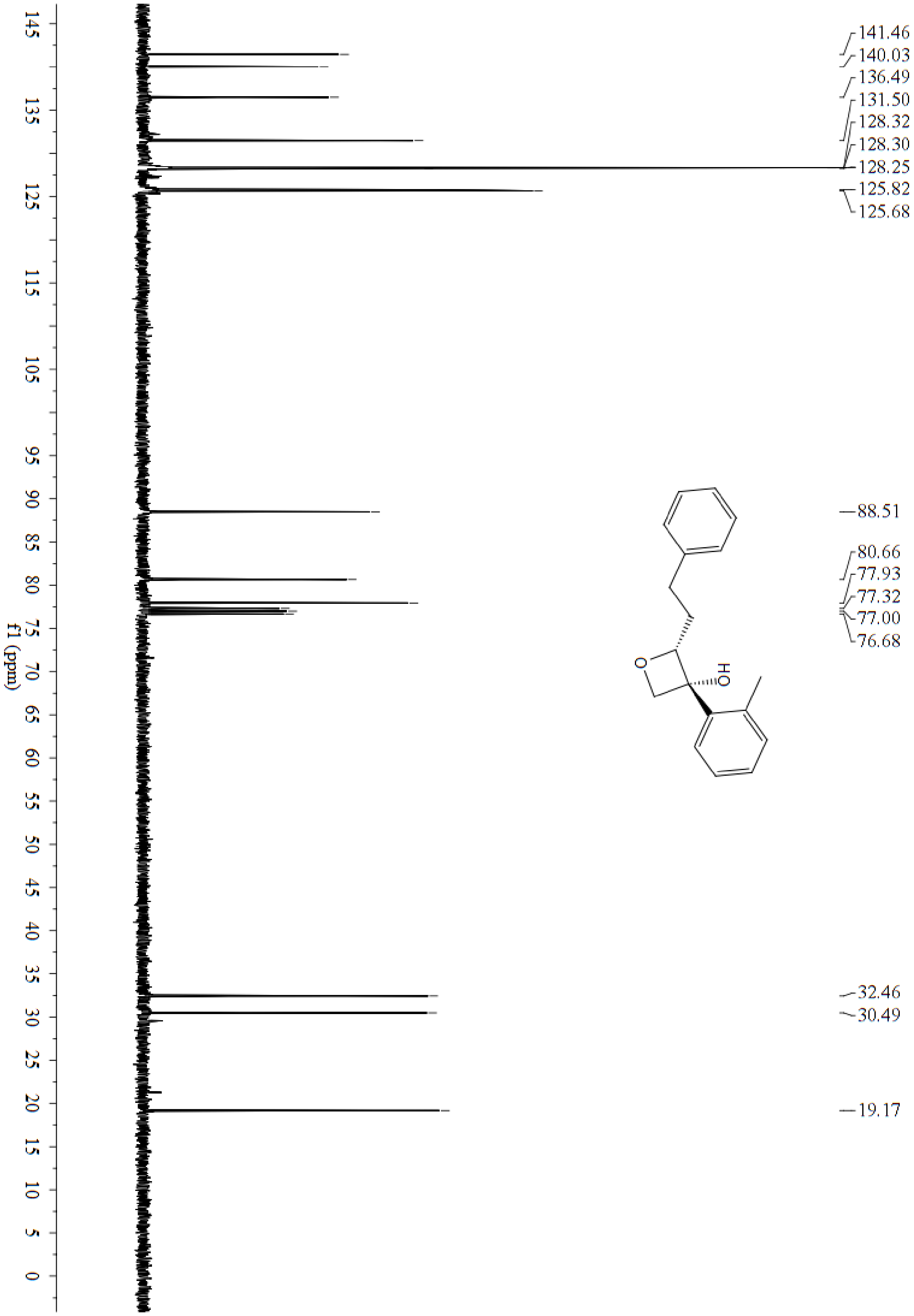

^{1}H NMR (400MHz,$CDCl_3$) 3-(4-fluorophenyl)-2-phenethyloxetan-3-ol (3q)

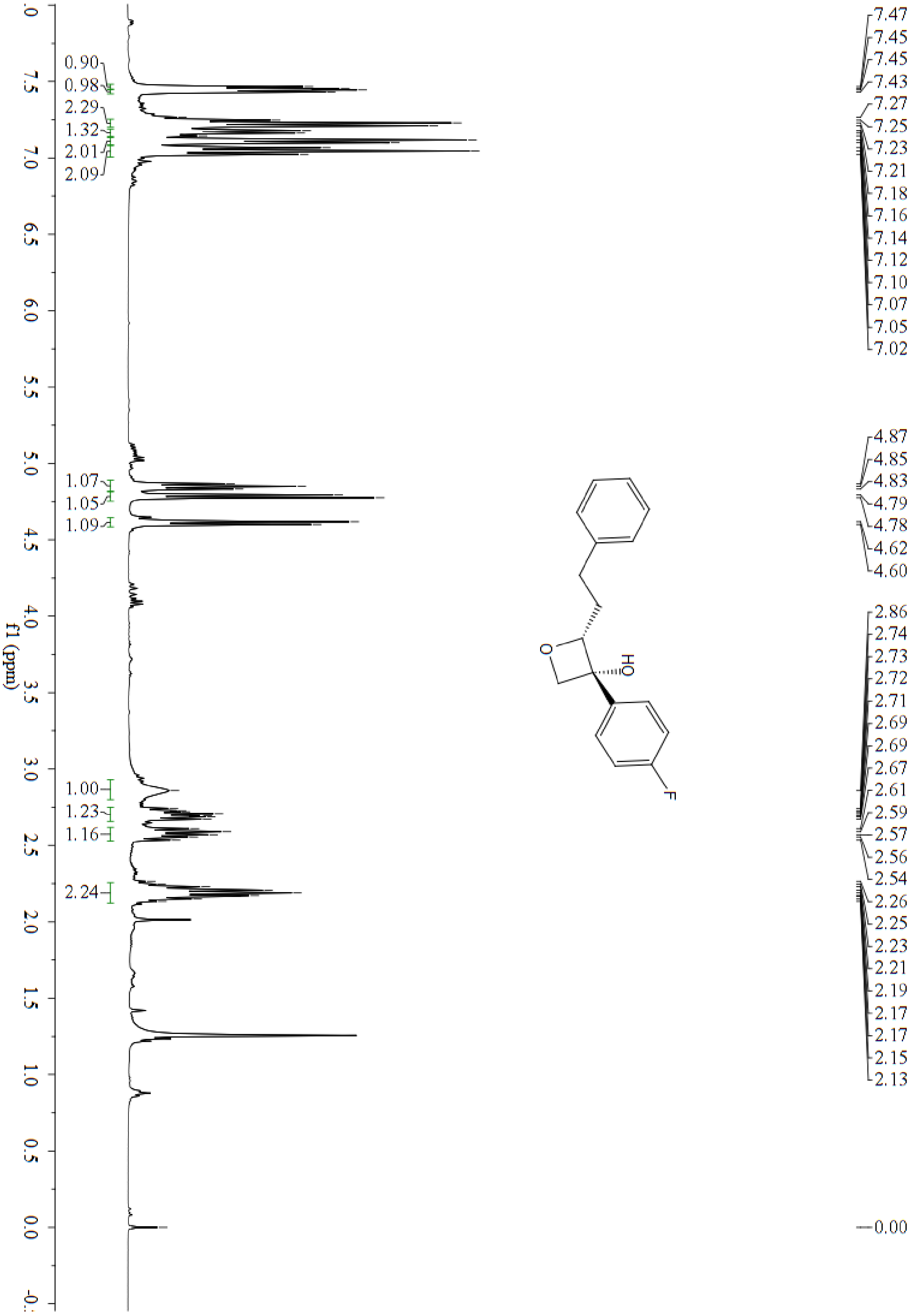

^{13}C NMR (101MHz,$CDCl_3$) 3-(4-fluorophenyl)-2-phenethyloxetan-3-ol (3q)

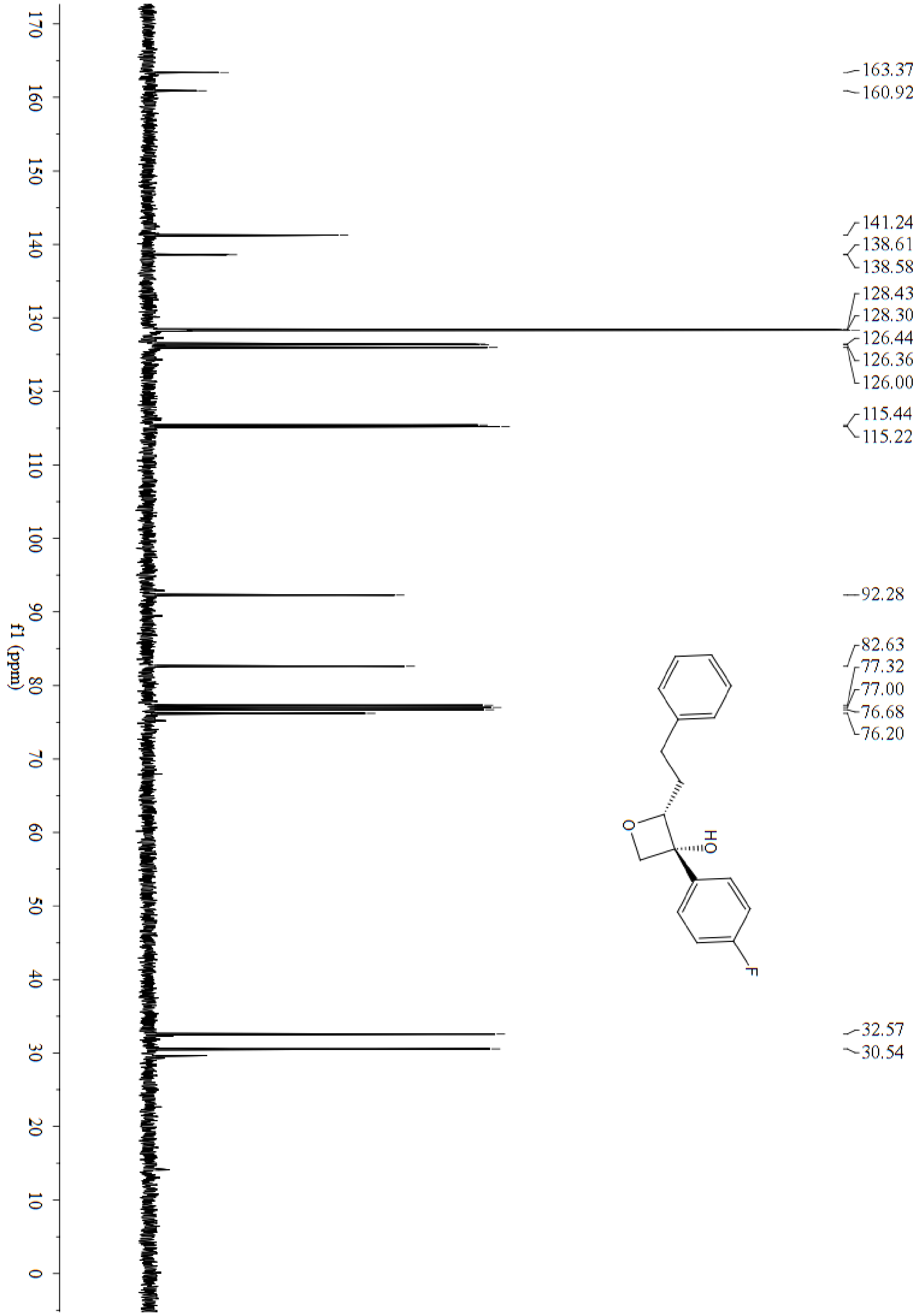

^{1}H NMR (400MHz,CDCl$_3$) 2-pentyl-3-phenyloxetan-3-ol (3r)

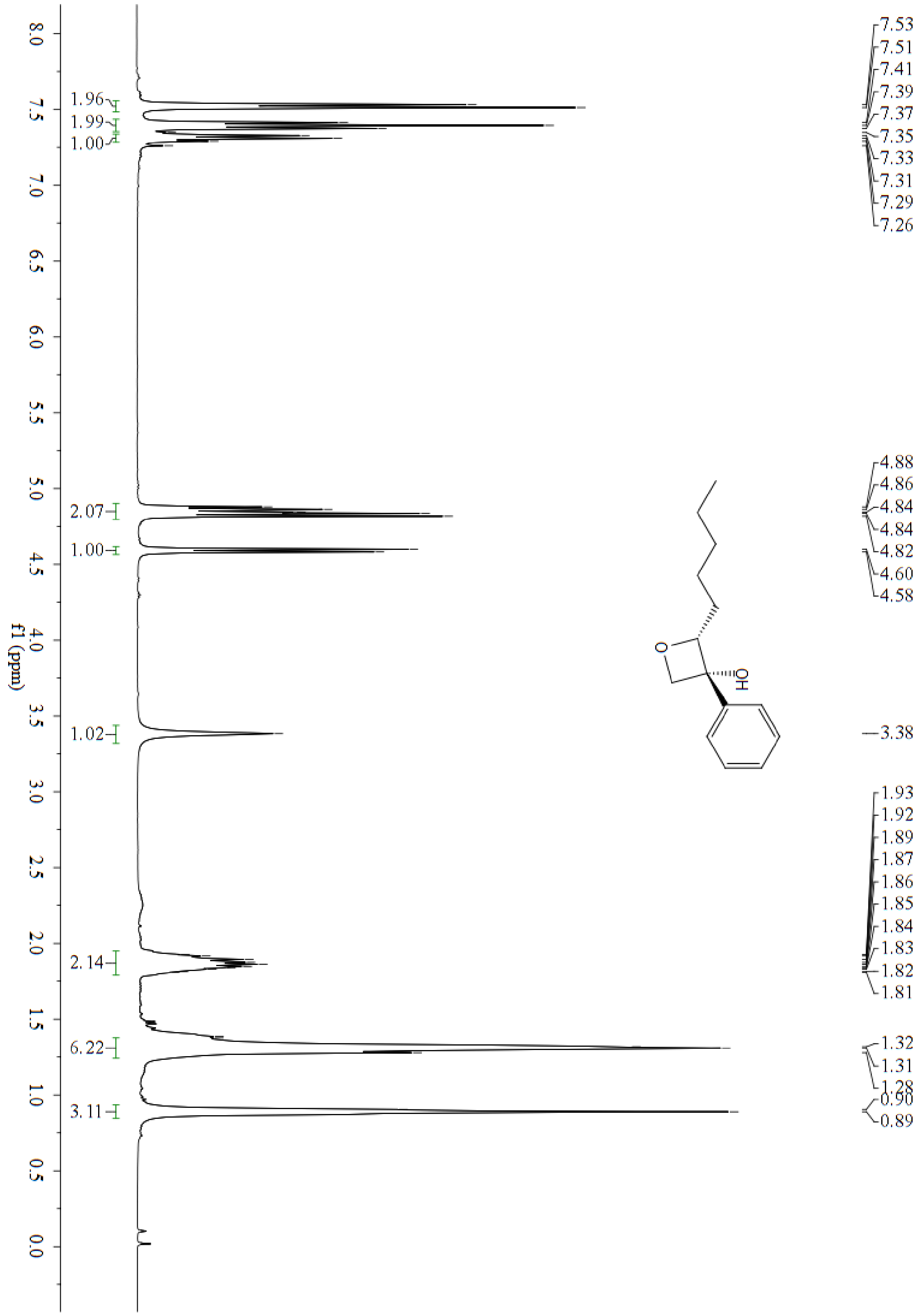

^{13}C NMR (101MHz,CDCl$_3$) 2-pentyl-3-phenyloxetan-3-ol (3r)

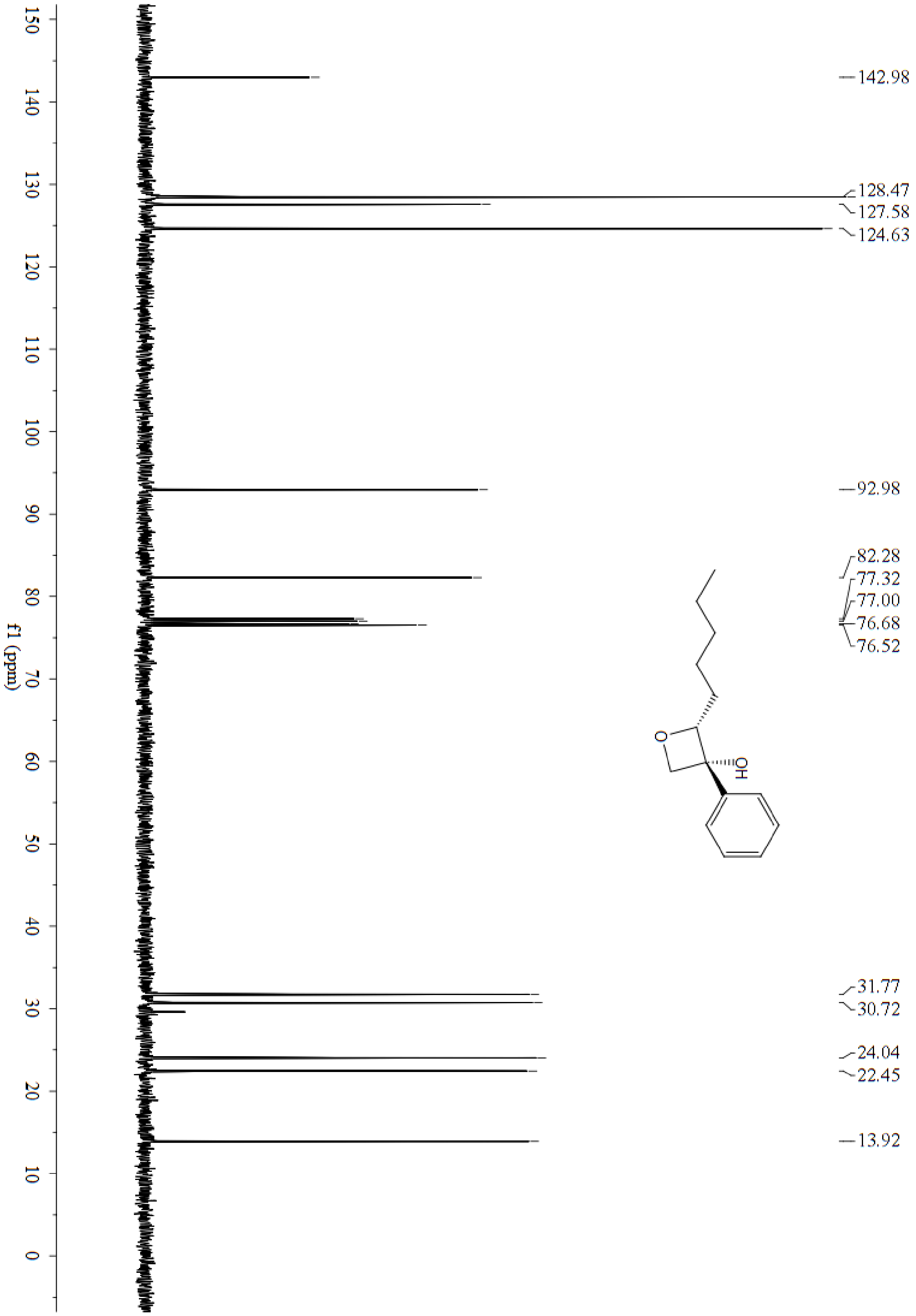

¹HNMR (400MHz,CDCl$_3$) (R)-3,4,5-triphenyl-3,6-dihydro-2H-pyran-3-ol (2a)

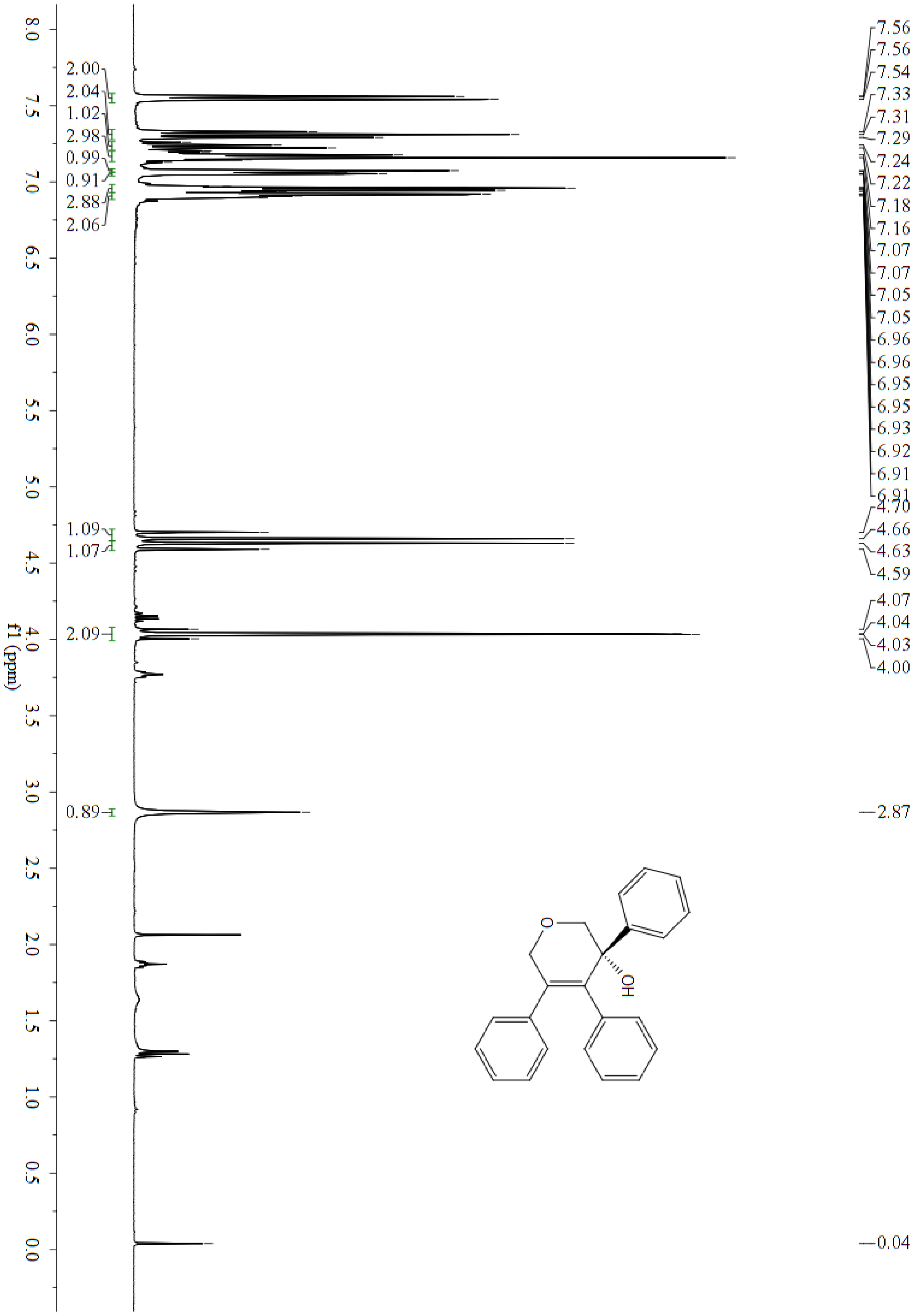

^{13}CNMR(101MHz,$CDCl_3$) (R)-3,4,5-triphenyl-3,6-dihydro-2H-pyran-3-ol (2a)

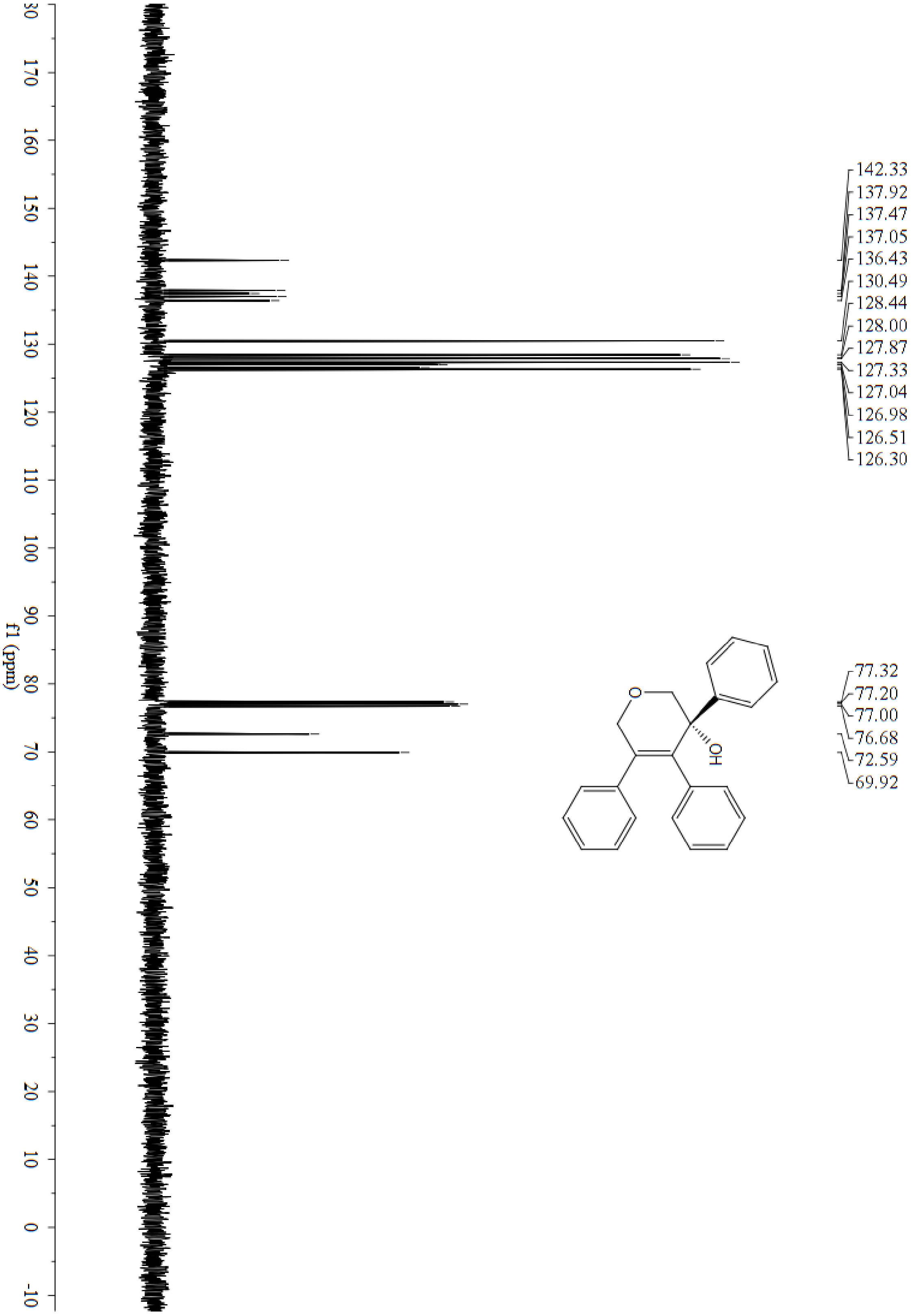

HPLC (R)-3,4,5-triphenyl-3,6-dihydro-2H-pyran-3-ol (2a, Racemic)

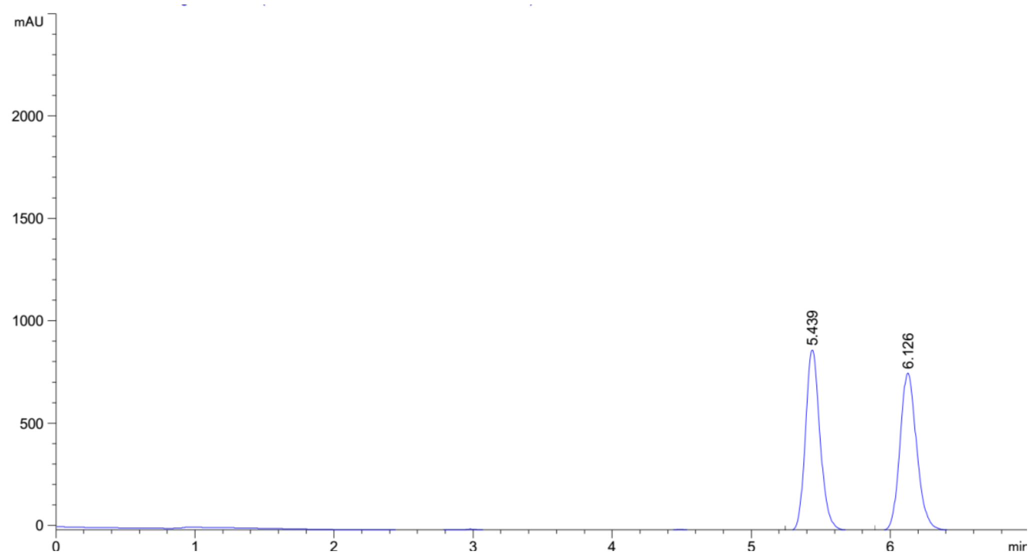

Peak #	RetTime [min]	Type	Width [min]	Area [mAU*s]	Height [mAU]	Area %
1	5.439	BB	0.1111	6445.32520	883.28857	49.8424
2	6.126	BB	0.1304	6486.08496	769.16547	50.1576

Totals : 1.29314e4 1652.45404

HPLC (R)-3,4,5-triphenyl-3,6-dihydro-2H-pyran-3-ol (2a, 96.5 : 3.5 er)

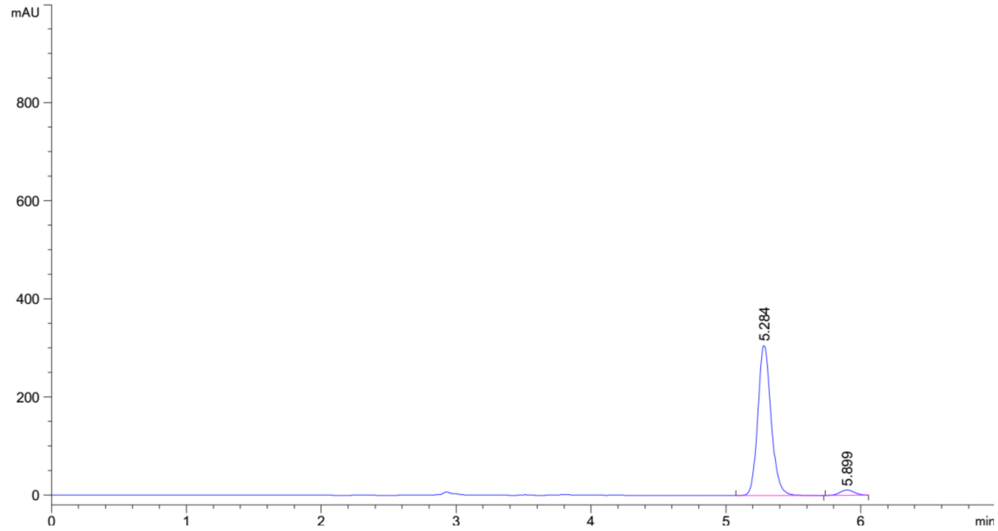

Peak #	RetTime [min]	Type	Width [min]	Area [mAU*s]	Height [mAU]	Area %
1	5.284	BB	0.1046	2086.41089	305.65848	96.4467
2	5.899	BBA	0.1132	76.86755	10.65185	3.5533

Totals : 2163.27843 316.31033

^{1}H NMR (400 MHz, $CDCl_3$) (R)-4,5-diphenyl-3-(p-tolyl)-3,6-dihydro-2H-pyran-3-ol (2b)

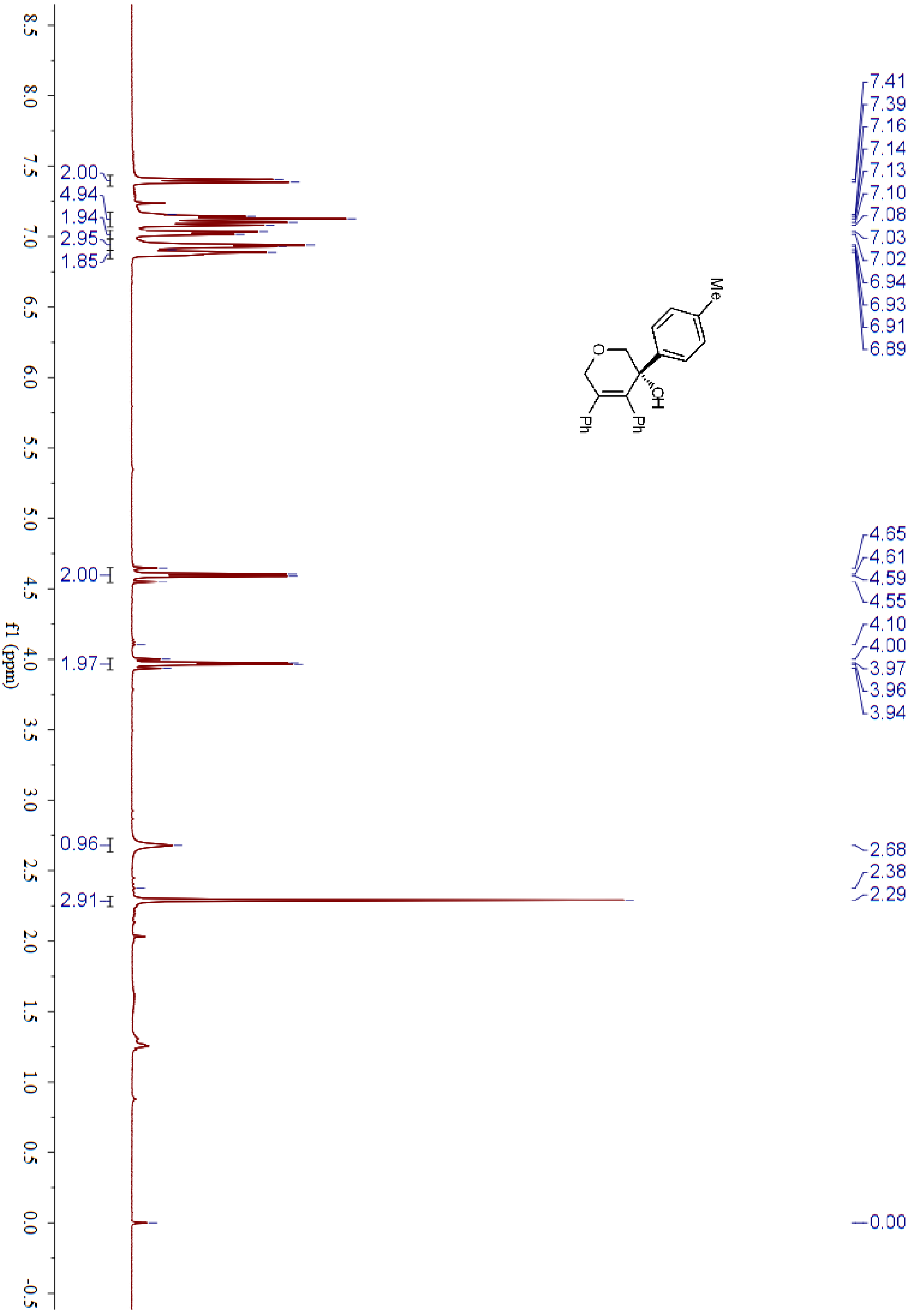

^{13}C NMR (100 MHz, $CDCl_3$) (R)-4,5-diphenyl-3-(p-tolyl)-3,6-dihydro-2H-pyran-3-ol (2b)

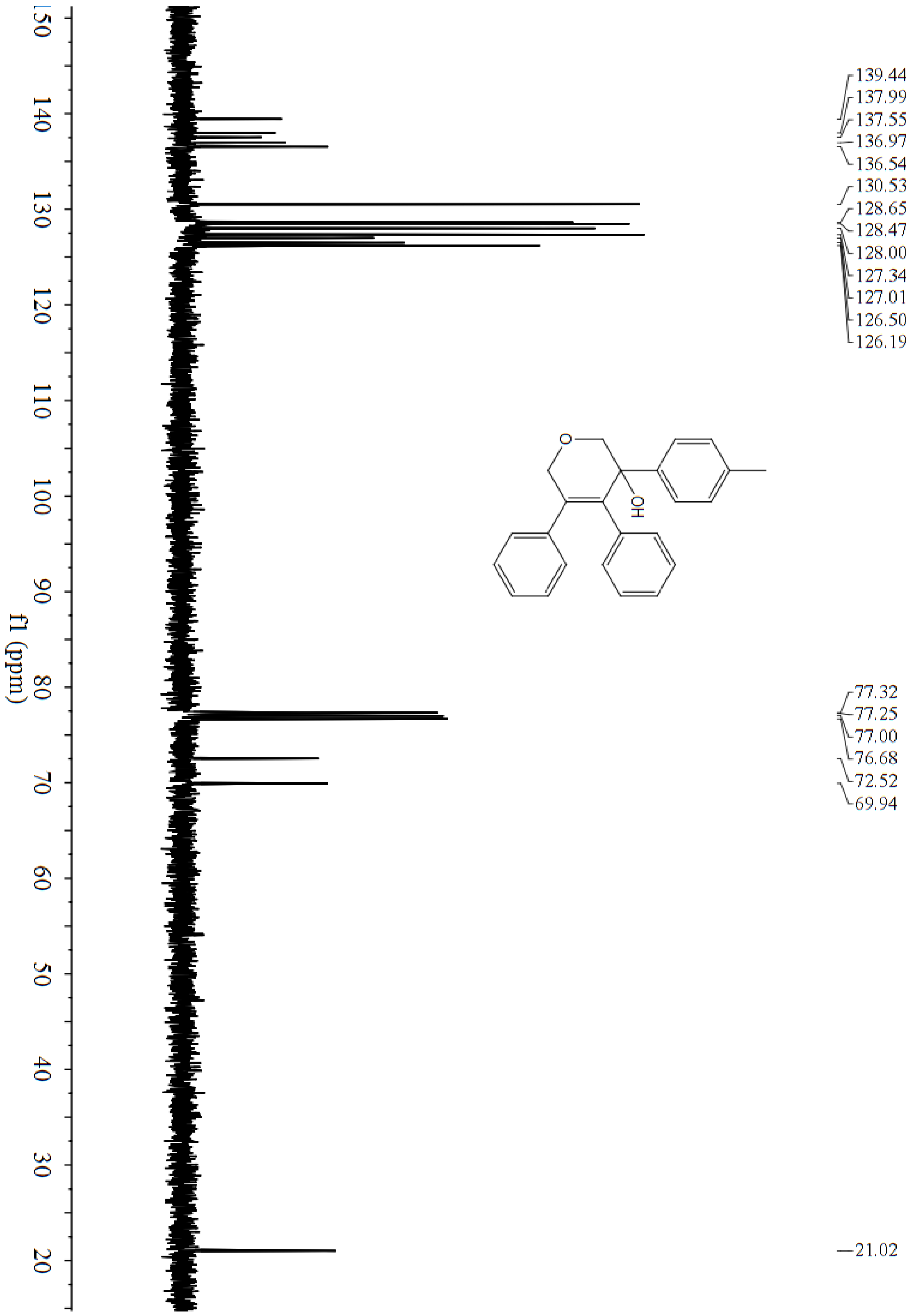

HPLC (R)-4,5-diphenyl-3-(p-tolyl)-3,6-dihydro-2H-pyran-3-ol (2b, Racemic)

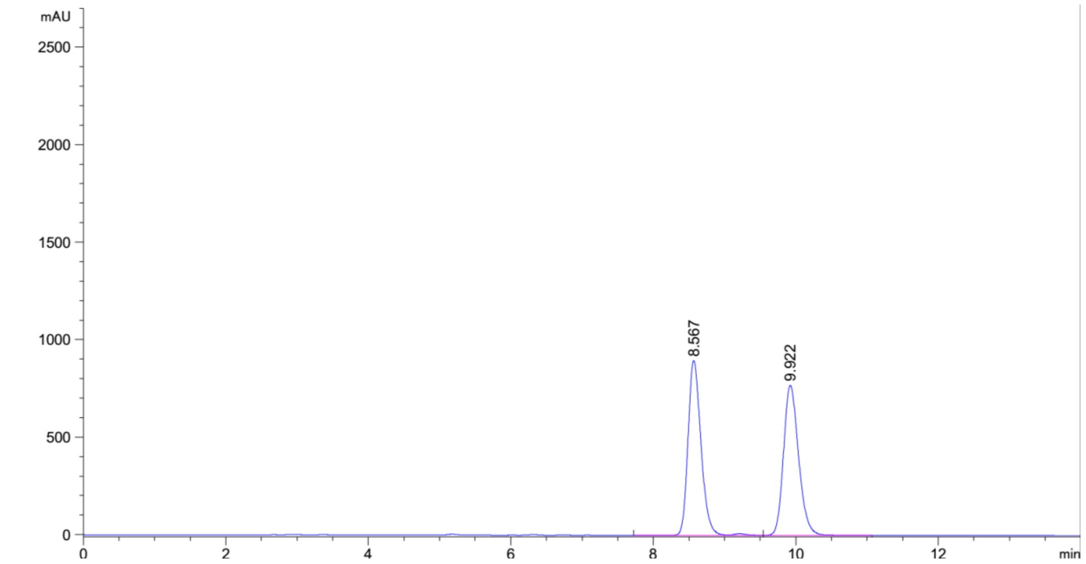

Peak #	RetTime [min]	Type	Width [min]	Area [mAU*s]	Height [mAU]	Area %
1	8.567	VV R	0.1936	1.13638e4	896.06091	50.1548
2	9.922	VB	0.2263	1.12937e4	769.27997	49.8452
Totals :				2.26575e4	1665.34088	

HPLC (R)-4,5-diphenyl-3-(p-tolyl)-3,6-dihydro-2H-pyran-3-ol (2b, 96.5 : 3.5 er)

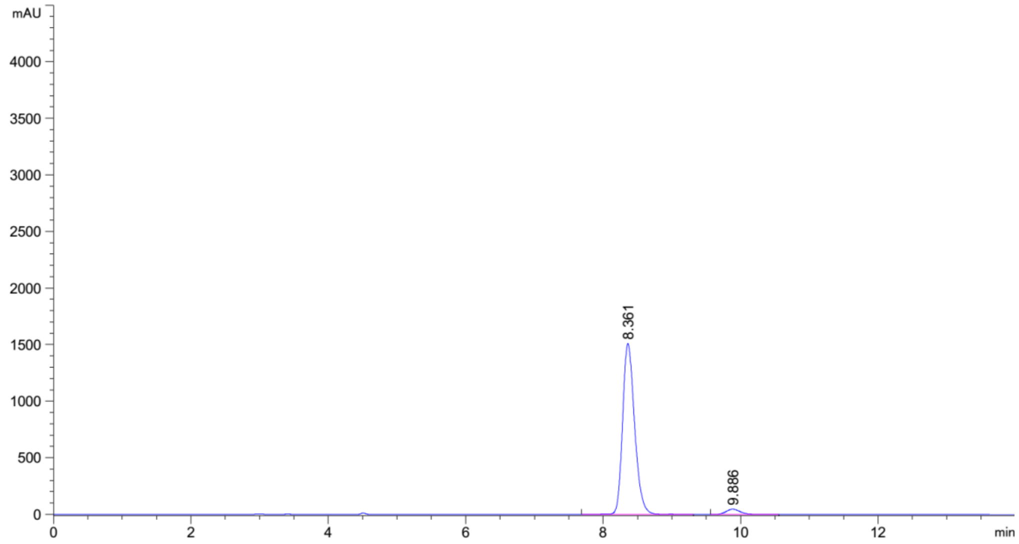

Peak #	RetTime [min]	Type	Width [min]	Area [mAU*s]	Height [mAU]	Area %
1	8.361	VV R	0.1860	1.83204e4	1510.06543	96.5121
2	9.886	BB	0.2197	662.08765	46.32725	3.4879
Totals :				1.89825e4	1556.39268	

^{1}H NMR(400MHz,$CDCl_3$)

(R)-3-(4-butylphenyl)-4,5-diphenyl-3,6-dihydro-2H-pyran-3-ol (2c)

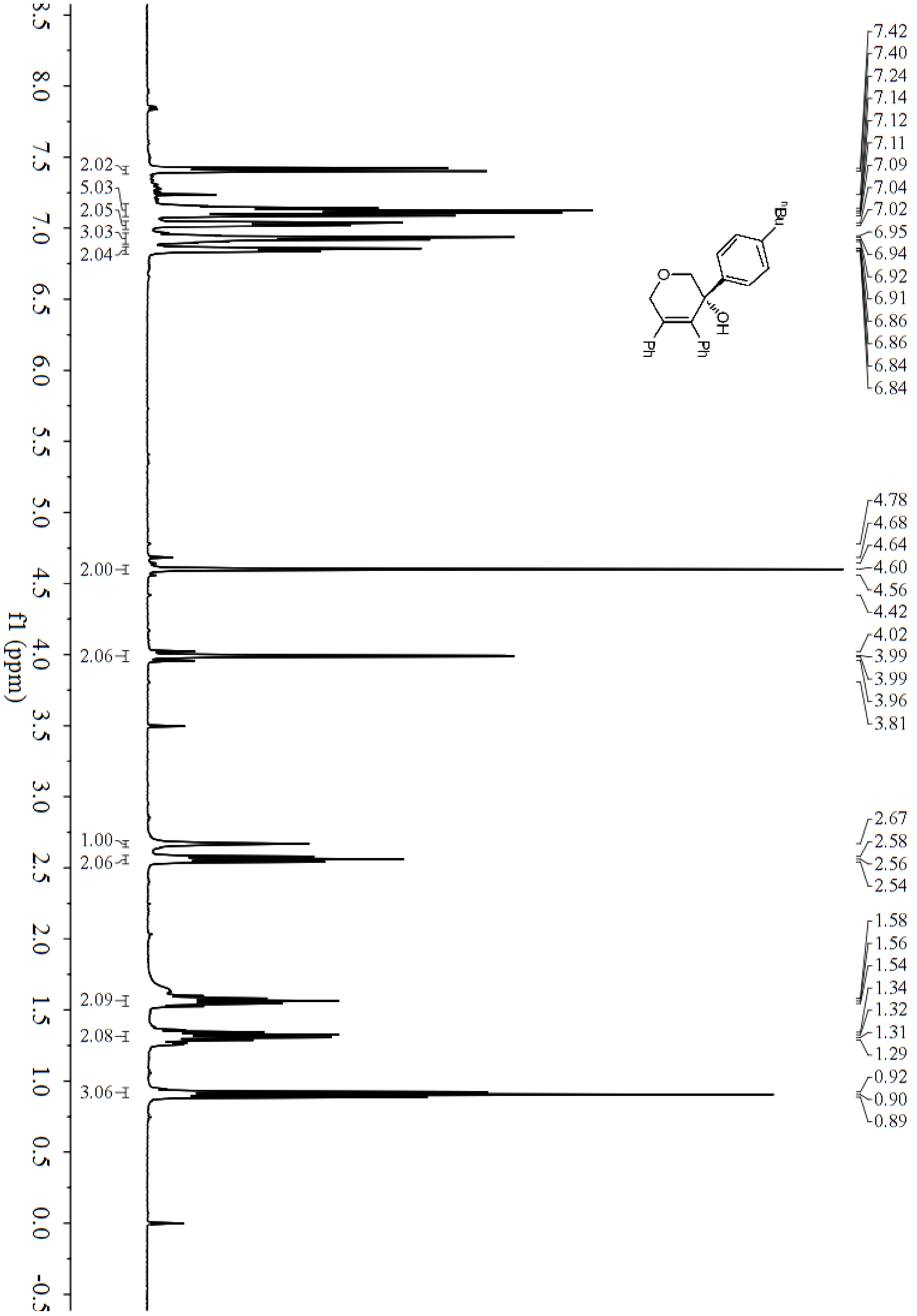

^{13}CNMR(100MHz,$CDCl_3$)

(R)-3-(4-butylphenyl)-4,5-diphenyl-3,6-dihydro-2H-pyran-3-ol (2c)

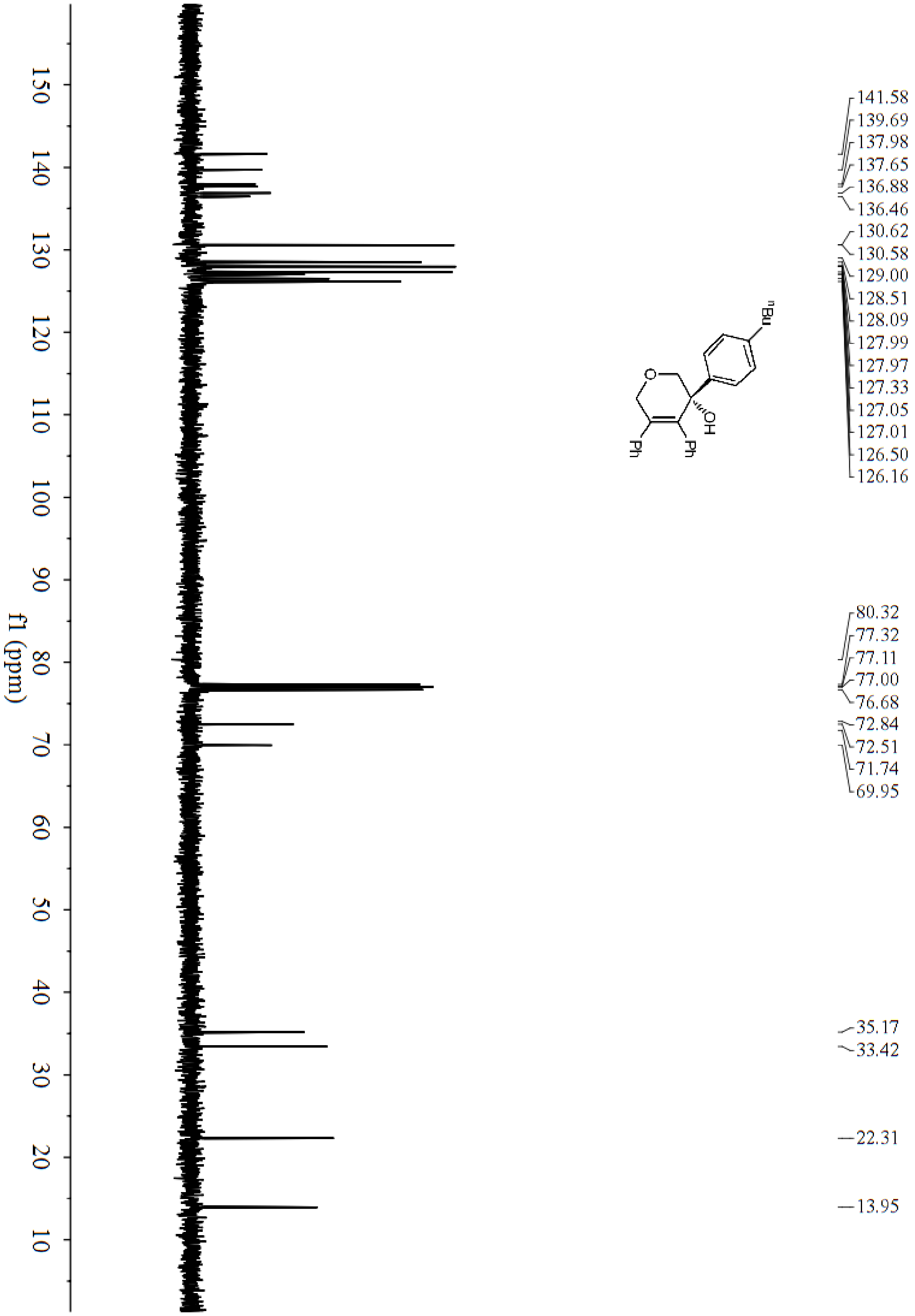

HPLC (R)-3-(4-butylphenyl)-4,5-diphenyl-3,6-dihydro-2H-pyran-3-ol (2c, Racemic)

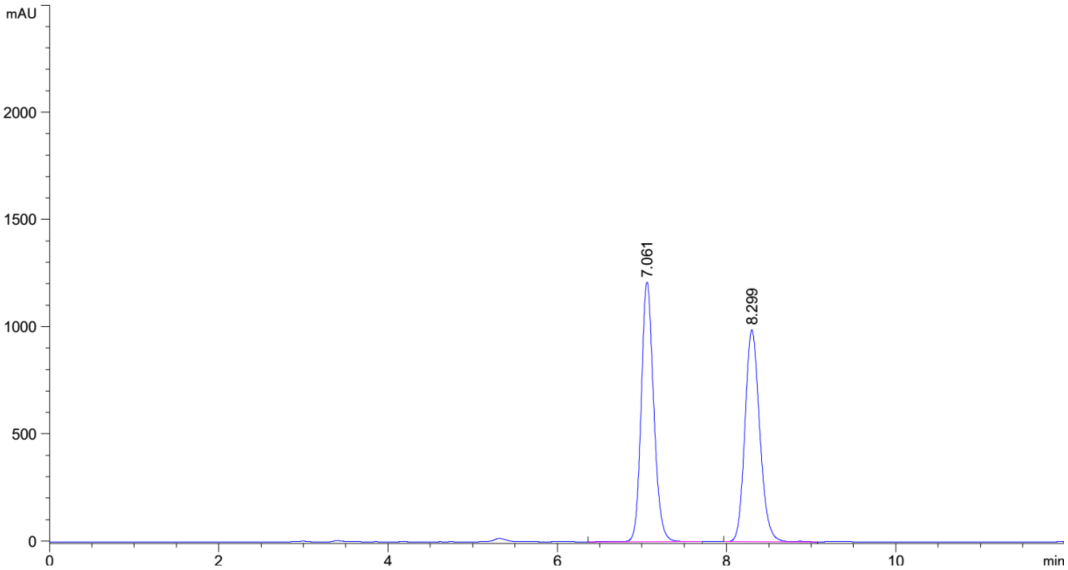

Peak #	RetTime [min]	Type	Width [min]	Area [mAU*s]	Height [mAU]	Area %
1	7.061	VB R	0.1573	1.24017e4	1211.38477	50.7768
2	8.299	BV R	0.1875	1.20222e4	989.21436	49.2232
Totals :				2.44239e4	2200.59912	

HPLC (R)-3-(4-butylphenyl)-4,5-diphenyl-3,6-dihydro-2H-pyran-3-ol (2c, 95.5 : 4.5 er)

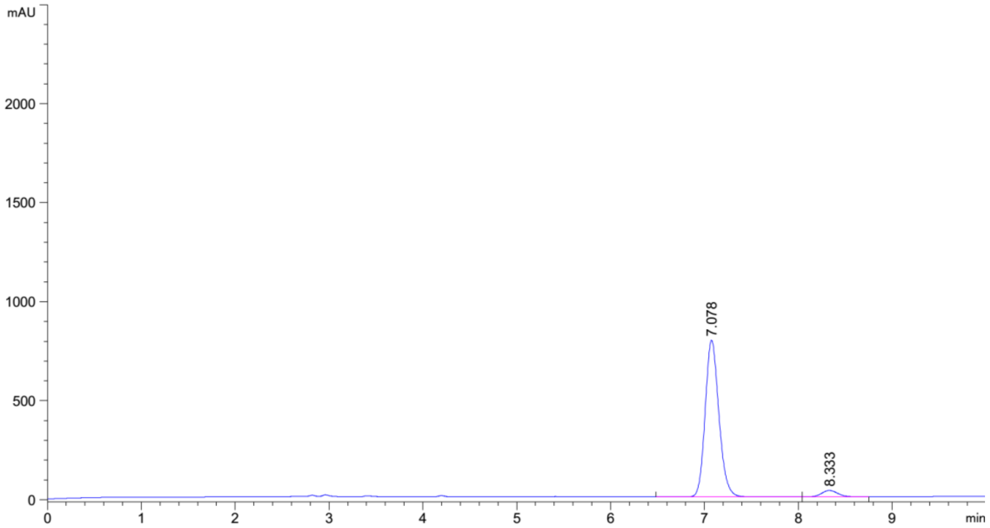

Peak #	RetTime [min]	Type	Width [min]	Area [mAU*s]	Height [mAU]	Area %
1	7.078	VV R	0.1567	8048.26807	791.01093	95.3195
2	8.333	BB	0.1869	395.19852	32.72651	4.6805
Totals :				8443.46658	823.73743	

^{1}HNMR(400MHz,$CDCl_3$)

(R)-3-(4-fluorophenyl)-4,5-diphenyl-3,6-dihydro-2H-pyran-3-ol (2d)

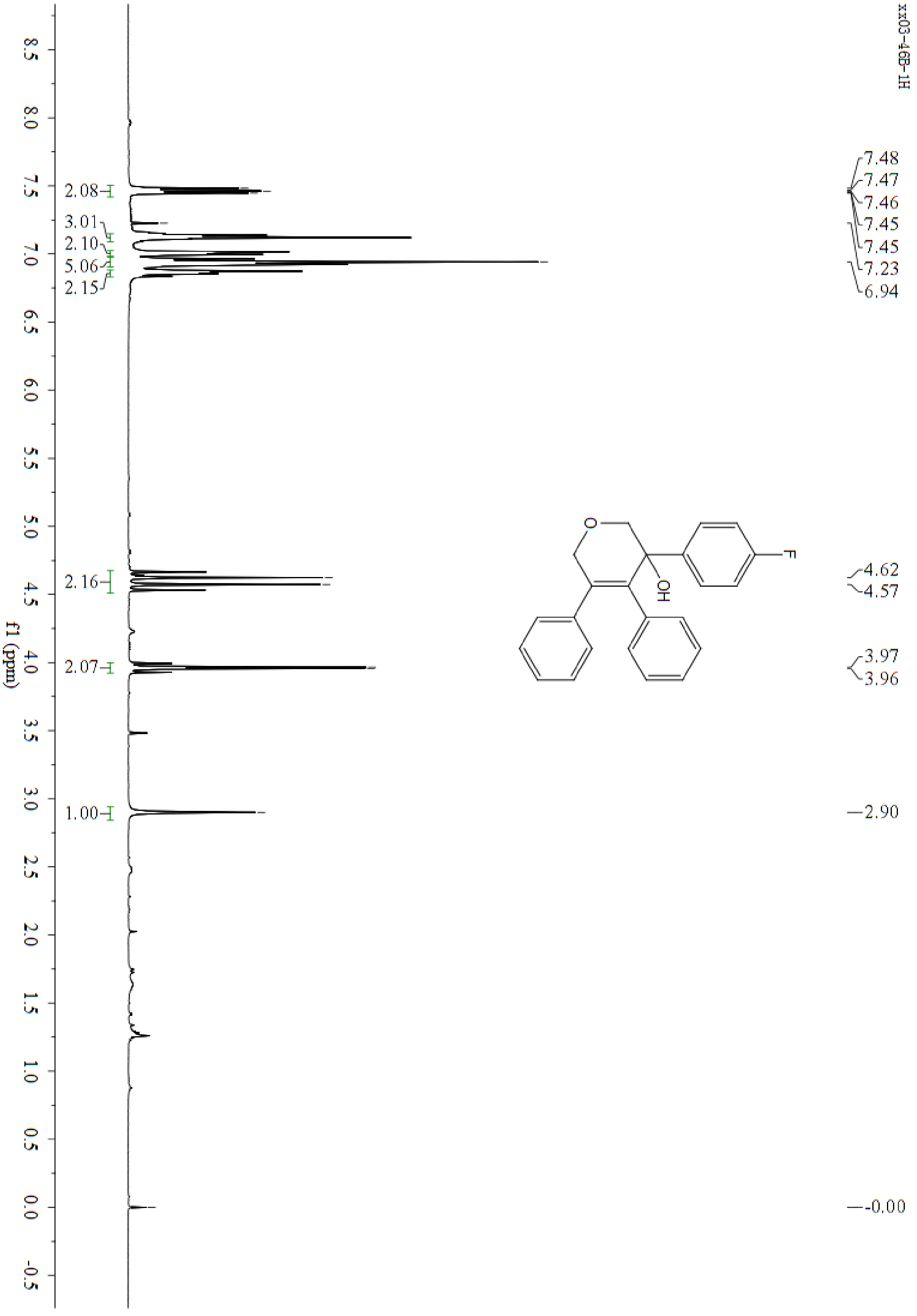

^{13}CNMR(100MHz,$CDCl_3$)

(R)-3-(4-fluorophenyl)-4,5-diphenyl-3,6-dihydro-2H-pyran-3-ol (2d)

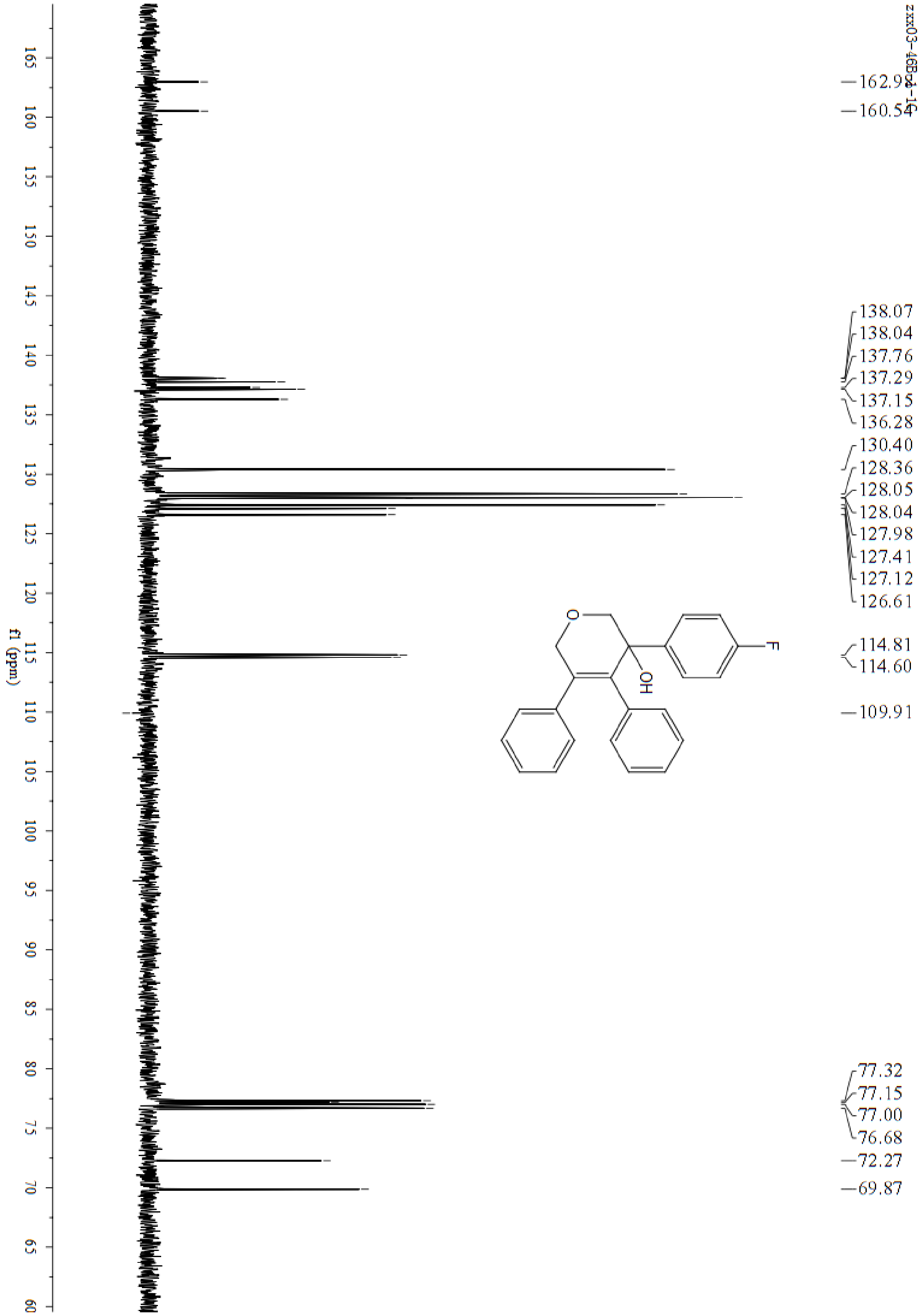

HPLC (R)-3-(4-fluorophenyl)-4,5-diphenyl-3,6-dihydro-2H-pyran-3-ol (2d, Racemic)

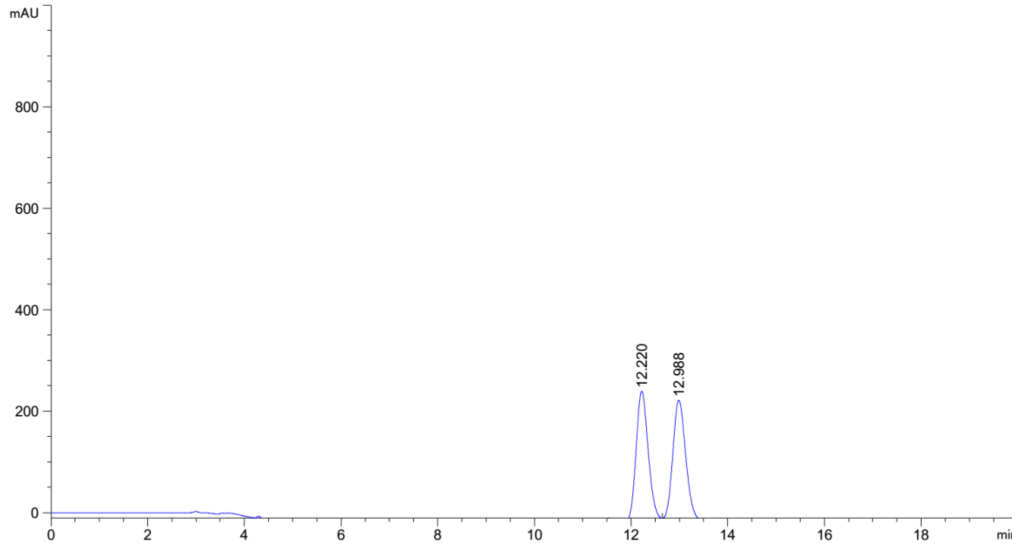

Peak #	RetTime [min]	Type	Width [min]	Area [mAU*s]	Height [mAU]	Area %
1	12.220	BV	0.2739	4625.63281	259.58408	49.8290
2	12.988	VB	0.2965	4657.38037	242.02448	50.1710
Totals :				9283.01318	501.60855	

HPLC (R)-3-(4-fluorophenyl)-4,5-diphenyl-3,6-dihydro-2H-pyran-3-ol (2d, 95 : 5 er)

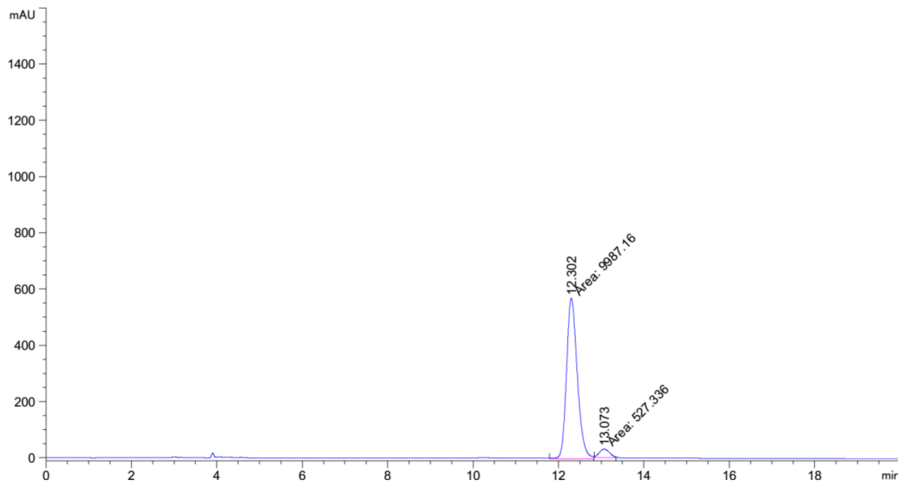

Peak #	RetTime [min]	Type	Width [min]	Area [mAU*s]	Height [mAU]	Area %
1	12.302	MM	0.2916	9987.15723	570.76788	94.9847
2	13.073	MM	0.2856	527.33618	30.77063	5.0153
Totals :				1.05145e4	601.53852	

^{1}HNMR(400MHz,CDCl$_3$) (S)-3-(2-fluorophenyl)-4,5-diphenyl-3,6-dihydro-2H-pyran-3-ol (2e)

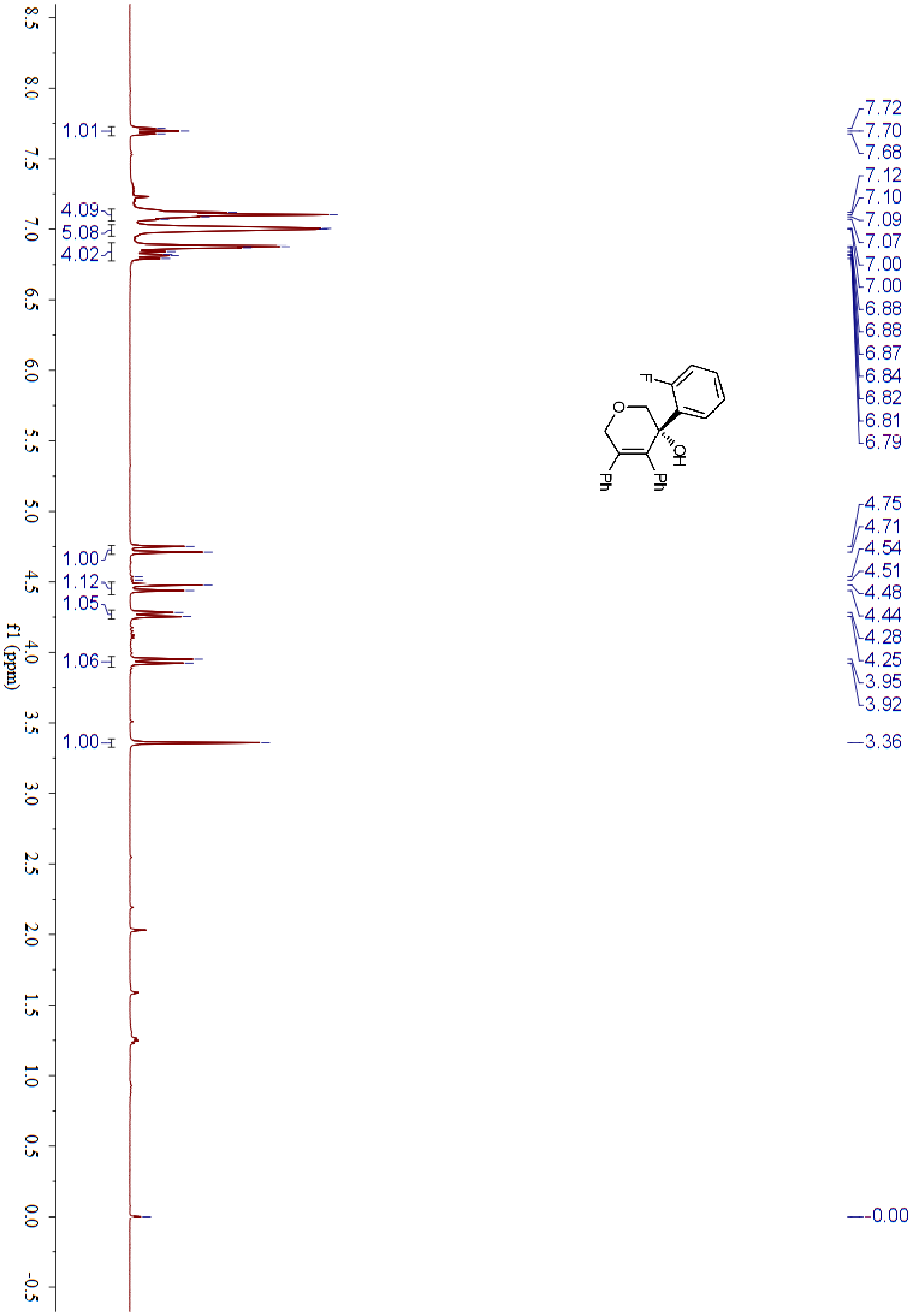

^{13}CNMR(100MHz,$CDCl_3$)(S)-3-(2-fluorophenyl)-4,5-diphenyl-3,6-dihydro-2H-pyran-3-ol (2e)

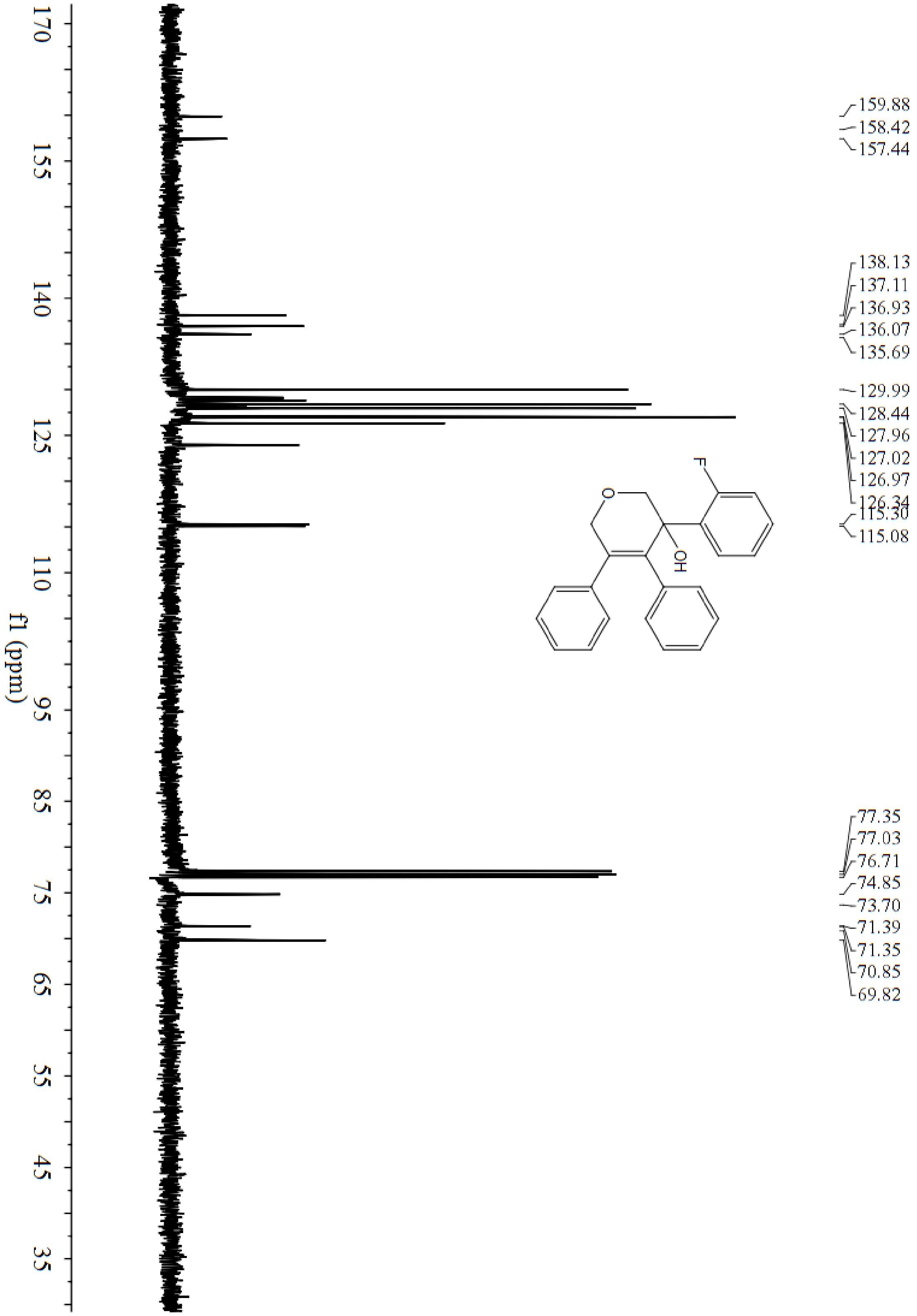

HPLC (S)-3-(2-fluorophenyl)-4,5-diphenyl-3,6-dihydro-2H-pyran-3-ol (2e, Racemic)

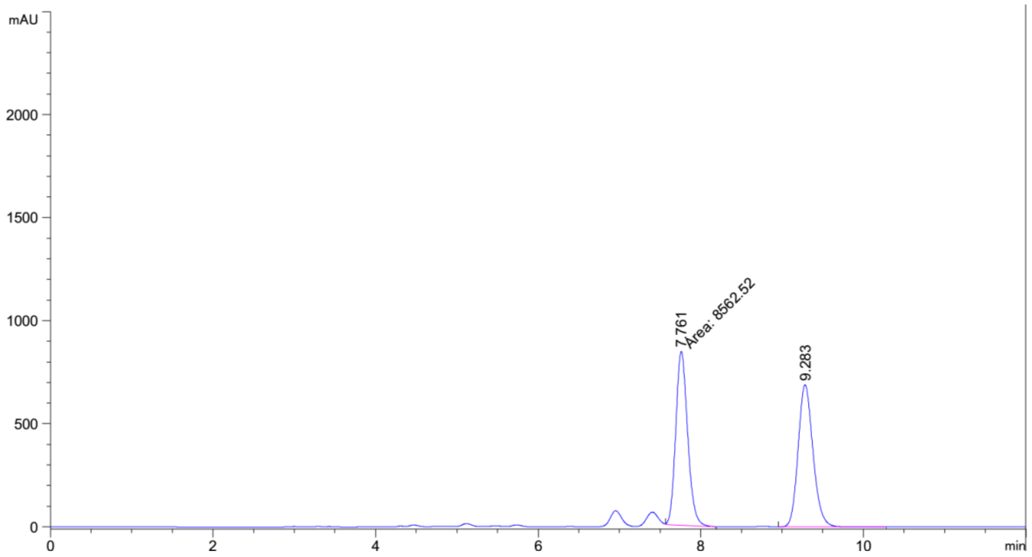

Peak #	RetTime [min]	Type	Width [min]	Area [mAU*s]	Height [mAU]	Area %
1	7.761	MM	0.1692	8562.52344	843.55243	49.4120
2	9.283	BB	0.1964	8766.31055	688.98444	50.5880

Totals : 1.73288e4 1532.53687

HPLC (S)-3-(2-fluorophenyl)-4,5-diphenyl-3,6-dihydro-2H-pyran-3-ol (2e, 96 : 4 er)

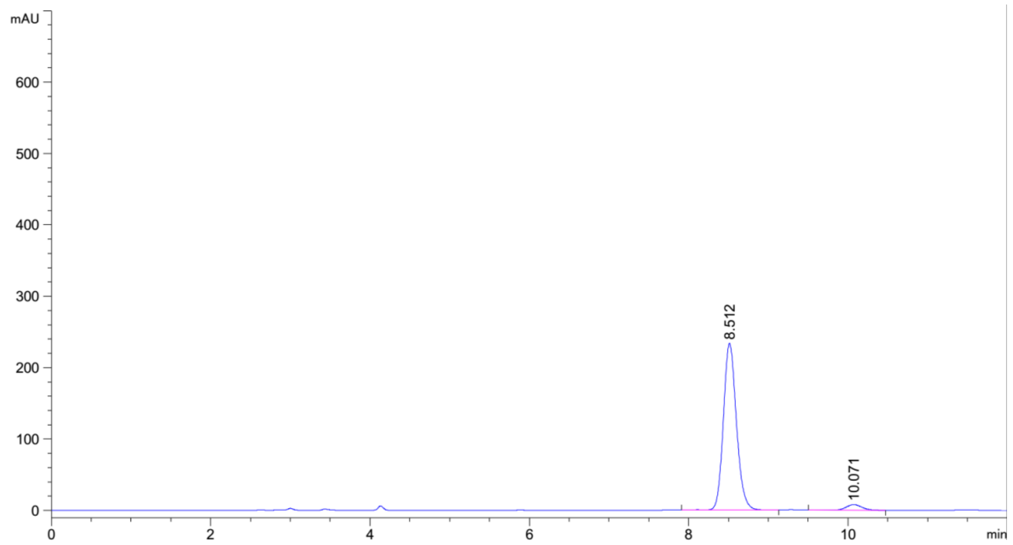

Peak #	RetTime [min]	Type	Width [min]	Area [mAU*s]	Height [mAU]	Area %
1	8.512	VB R	0.1750	2663.86133	233.63075	96.0658
2	10.071	BB	0.2117	109.09466	7.91866	3.9342

Totals : 2772.95599 241.54942

^{1}HNMR(400MHz,$CDCl_3$)(R)-3-(4-methoxyphenyl)-4,5-diphenyl-3,6-dihydro-2H-pyran-3-ol (2f)

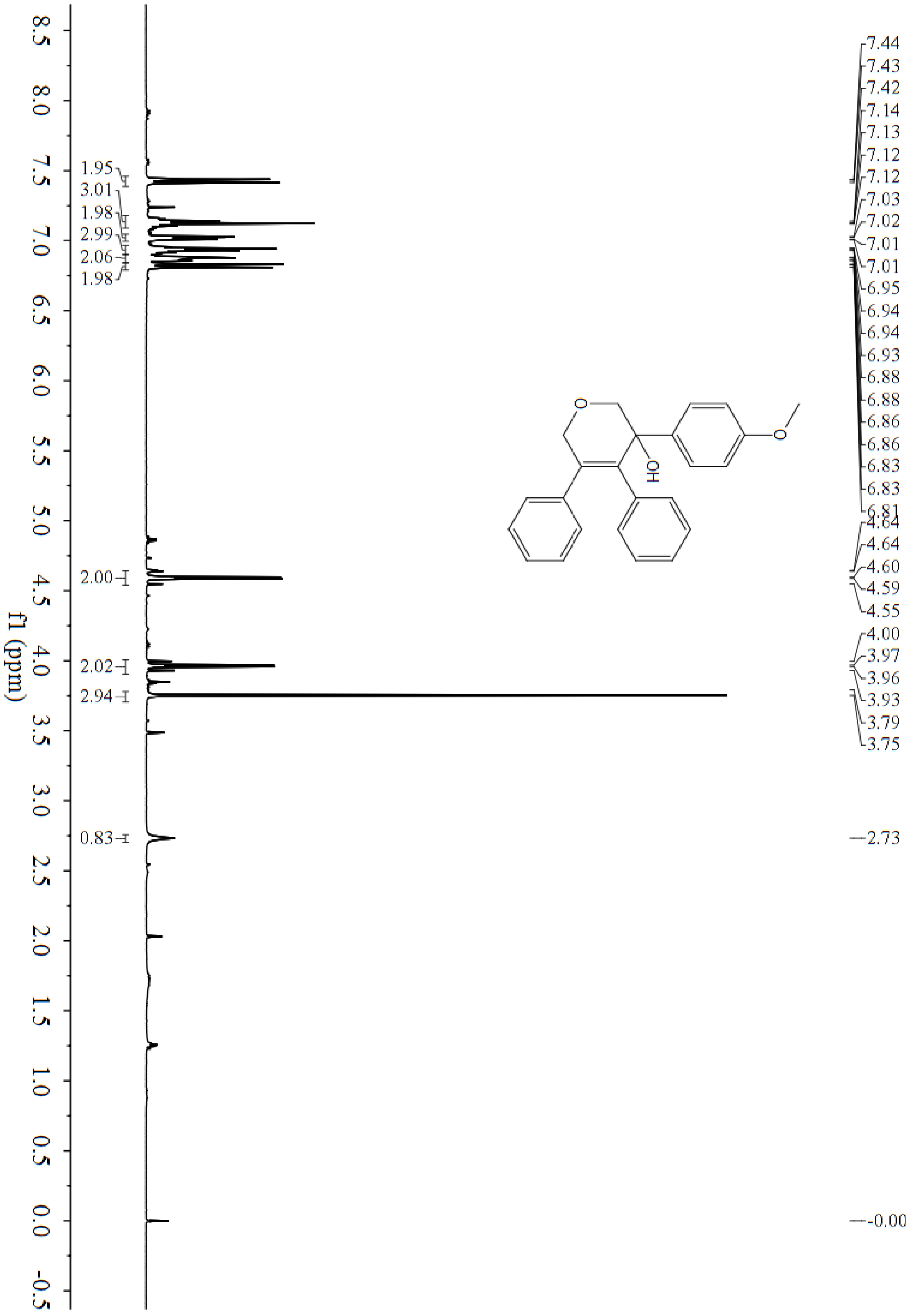

^{13}CNMR(100MHz,CDCl$_3$)(R)-3-(4-methoxyphenyl)-4,5-diphenyl-3,6-dihydro-2H-pyran-3-ol (2f)

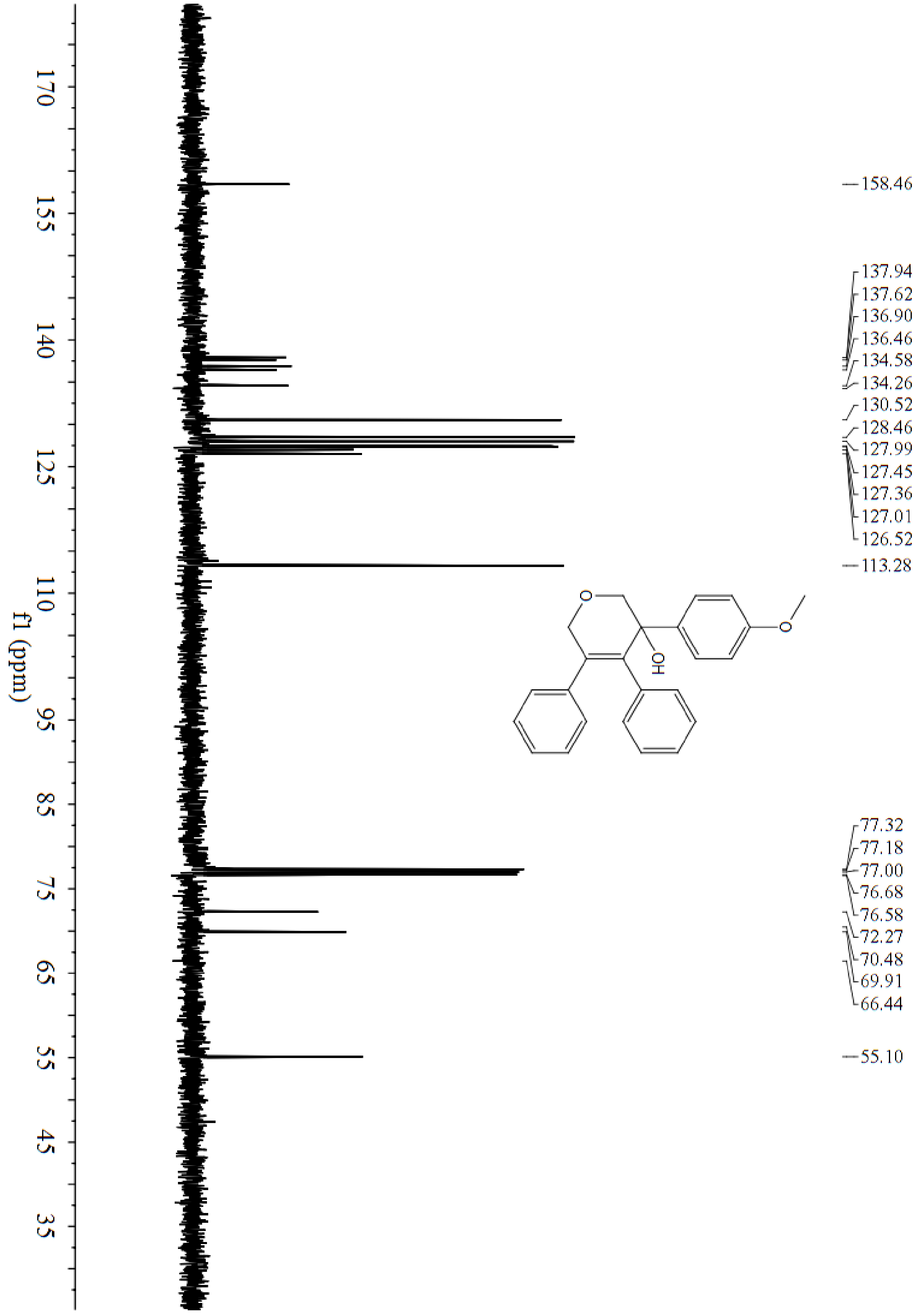

HPLC (R)-3-(4-methoxyphenyl)-4,5-diphenyl-3,6-dihydro-2H-pyran-3-ol (2f, Racemic)

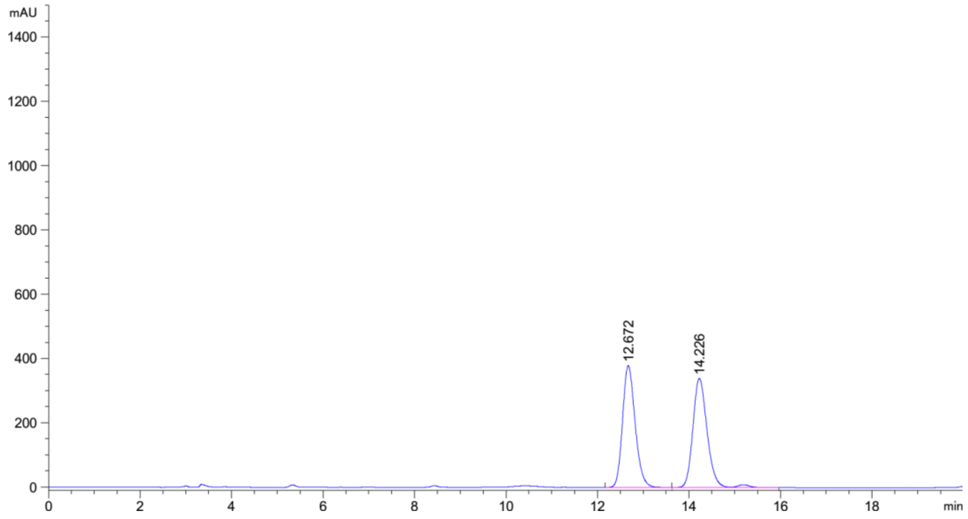

Peak #	RetTime [min]	Type	Width [min]	Area [mAU*s]	Height [mAU]	Area %
1	12.672	BB	0.2941	7241.06445	378.64798	49.5024
2	14.226	BV R	0.3288	7386.63721	339.01538	50.4976
Totals :				1.46277e4	717.66336	

HPLC (R)-3-(4-methoxyphenyl)-4,5-diphenyl-3,6-dihydro-2H-pyran-3-ol (2f, 95.5 : 4.5 er)

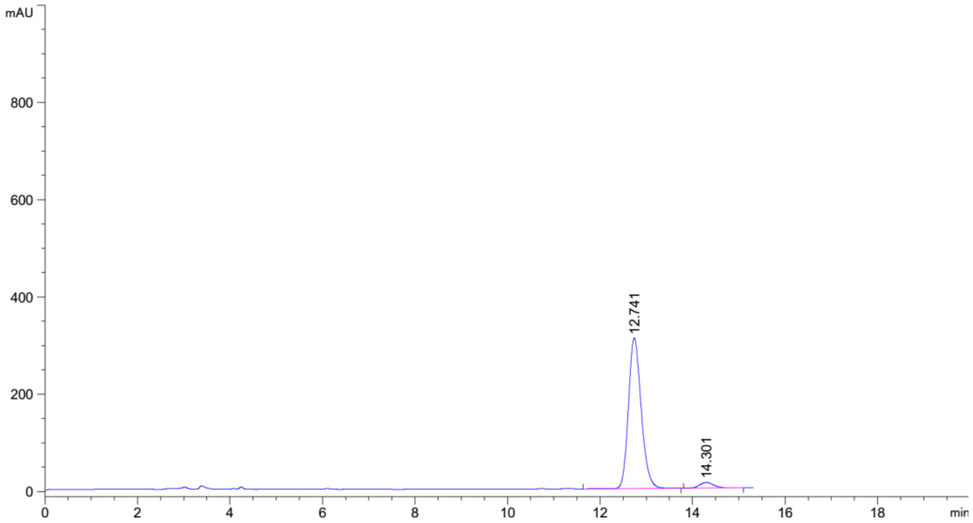

Peak #	RetTime [min]	Type	Width [min]	Area [mAU*s]	Height [mAU]	Area %
1	12.741	BB	0.2946	5930.71094	309.48645	95.9460
2	14.301	BB	0.3332	250.59274	11.54757	4.0540
Totals :				6181.30368	321.03402	

^{1}HNMR(400MHz,CDCl$_3$)(R)-3-(3-methoxyphenyl)-4,5-diphenyl-3,6-dihydro-2H-pyran-3-ol (2g)

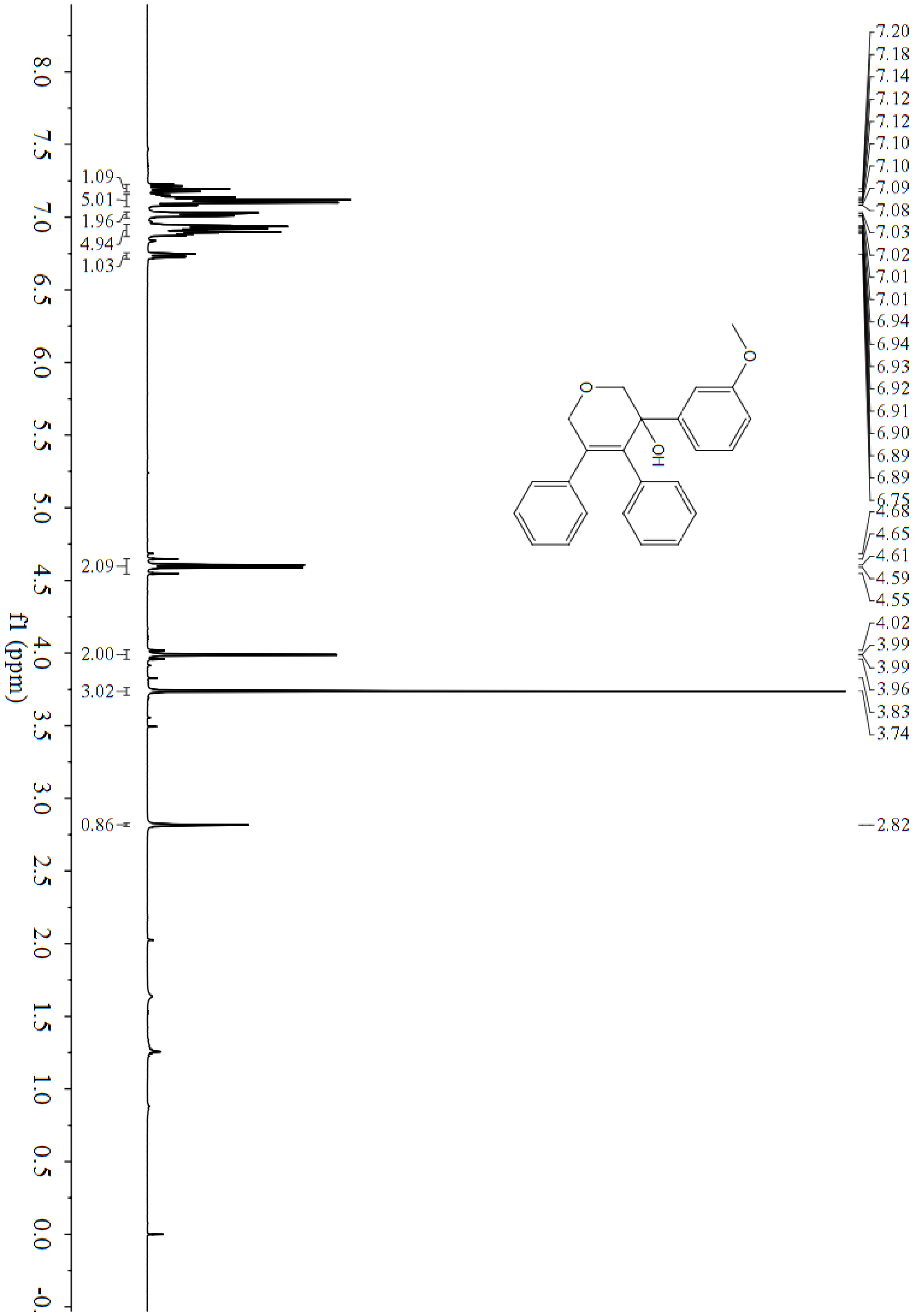

^{13}CNMR(100MHz,$CDCl_3$)(R)-3-(3-methoxyphenyl)-4,5-diphenyl-3,6-dihydro-2H-pyran-3-ol (2g)

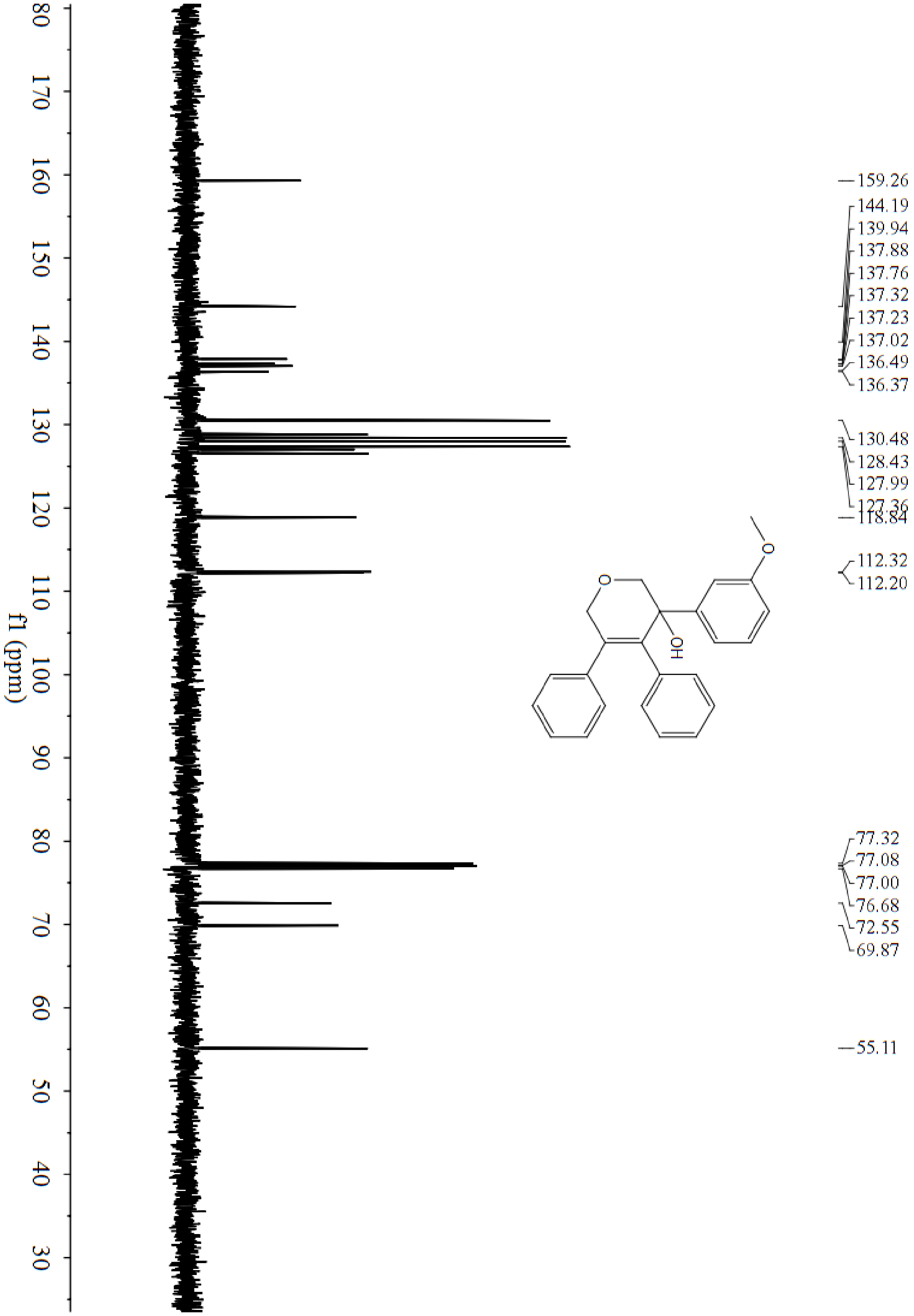

HPLC (R)-3-(3-methoxyphenyl)-4,5-diphenyl-3,6-dihydro-2H-pyran-3-ol (2g, Racemic)

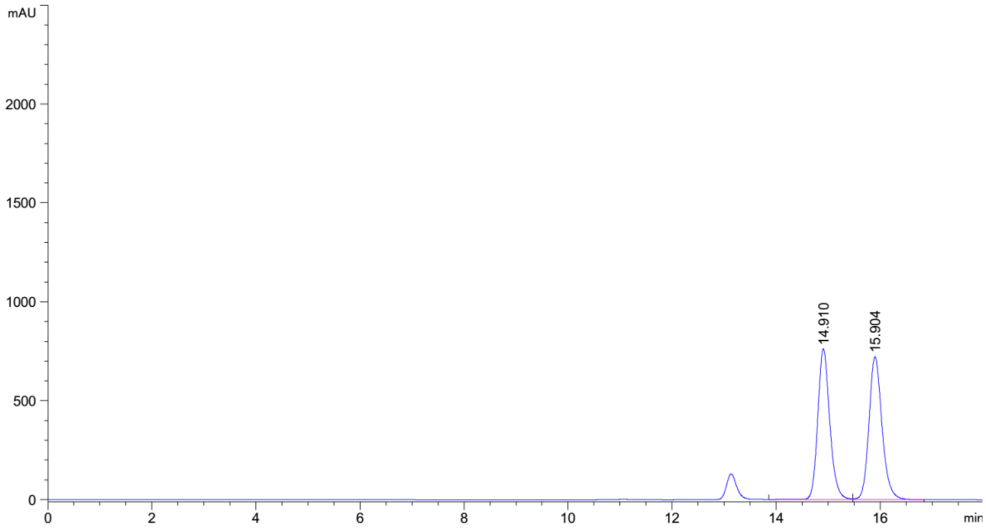

Peak #	RetTime [min]	Type	Width [min]	Area [mAU*s]	Height [mAU]	Area %
1	14.910	VV R	0.2455	1.22522e4	762.34613	49.2522
2	15.904	VB	0.2667	1.26242e4	722.95612	50.7478
Totals :				2.48764e4	1485.30225	

HPLC (R)-3-(3-methoxyphenyl)-4,5-diphenyl-3,6-dihydro-2H-pyran-3-ol (2g, 96 : 4 er)

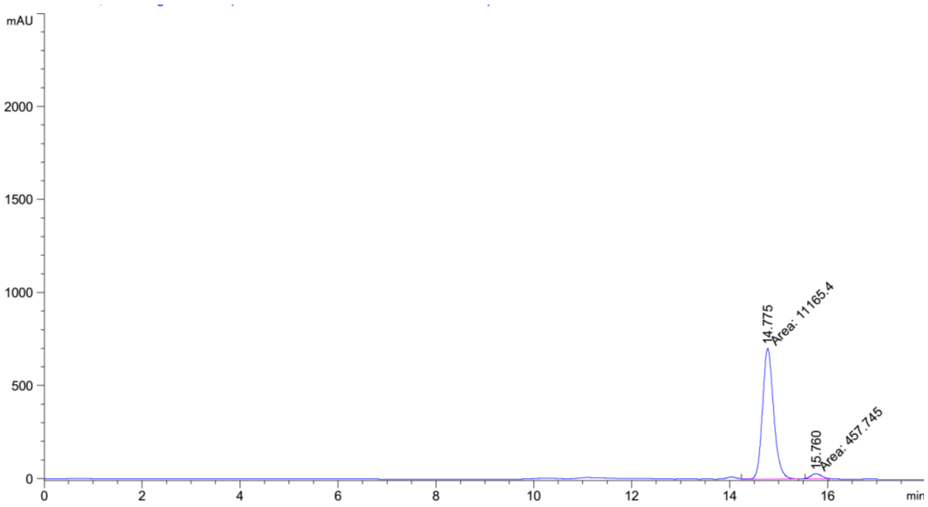

Peak #	RetTime [min]	Type	Width [min]	Area [mAU*s]	Height [mAU]	Area %
1	14.775	MM	0.2647	1.11654e4	702.89313	96.0618
2	15.760	MM	0.2671	457.74493	28.56676	3.9382
Totals :				1.16232e4	731.45989	

¹HNMR(400MHz,CDCl$_3$)(R)-3-(3,4-dimethoxyphenyl)-4,5-diphenyl-3,6-dihydro-2H-pyran-3-ol (2h)

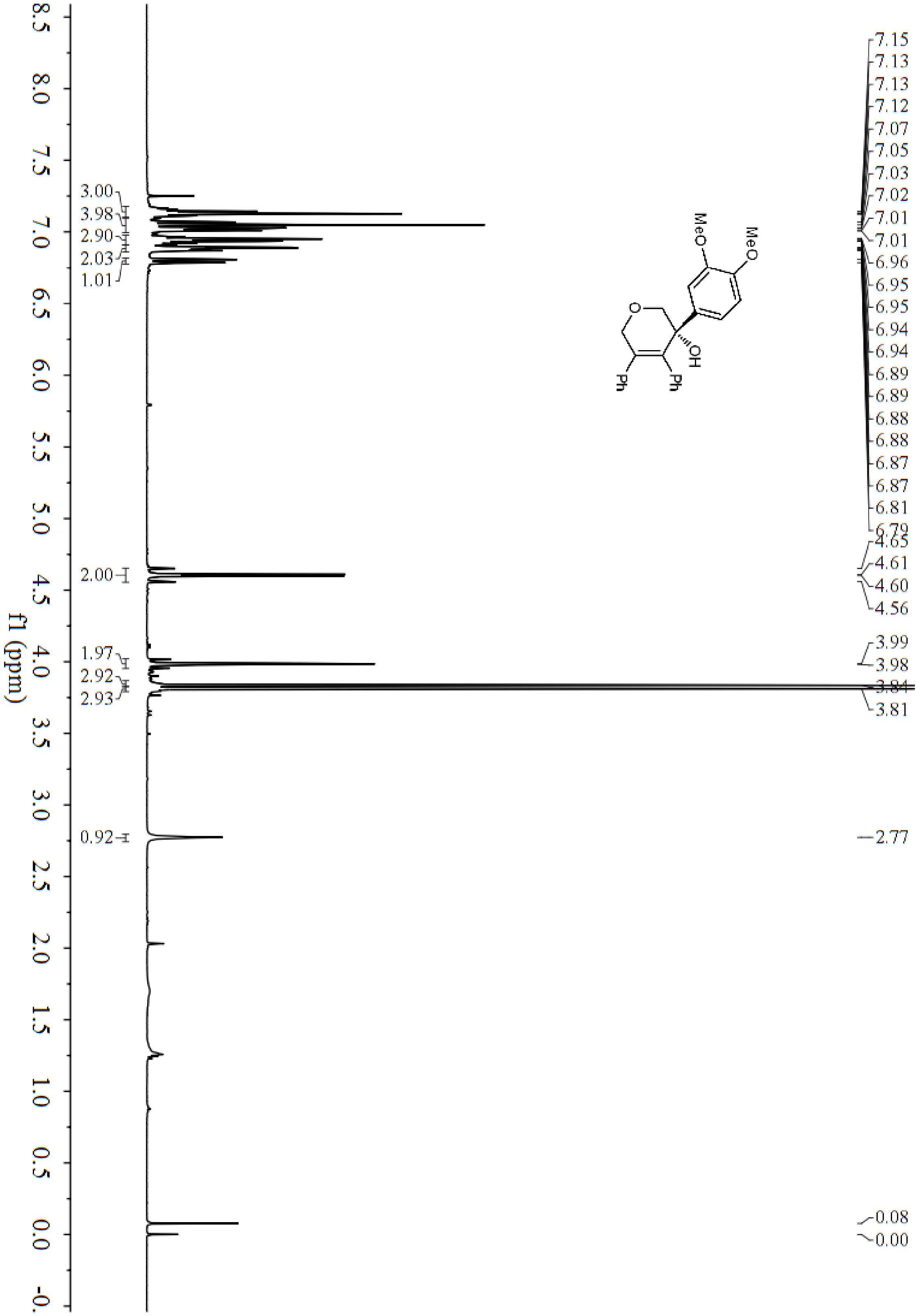

^{13}CNMR(100MHz,$CDCl_3$)(R)-3-(3,4-dimethoxyphenyl)-4,5-diphenyl-3,6-dihydro-2H-pyran-3-ol (2h)

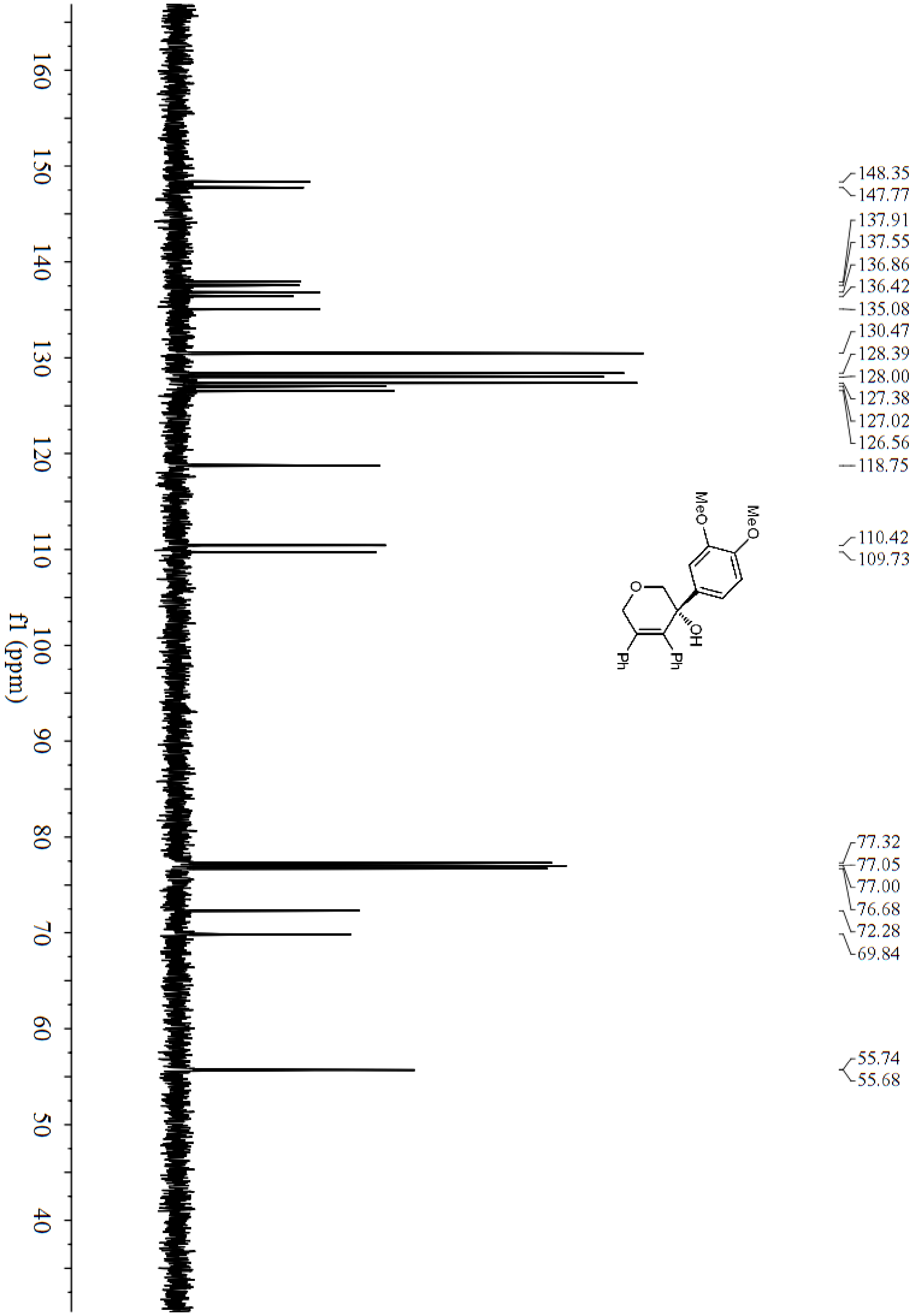

HPLC (R)-3-(3,4-dimethoxyphenyl)-4,5-diphenyl-3,6-dihydro-2H-pyran-3-ol (2h, Racemic)

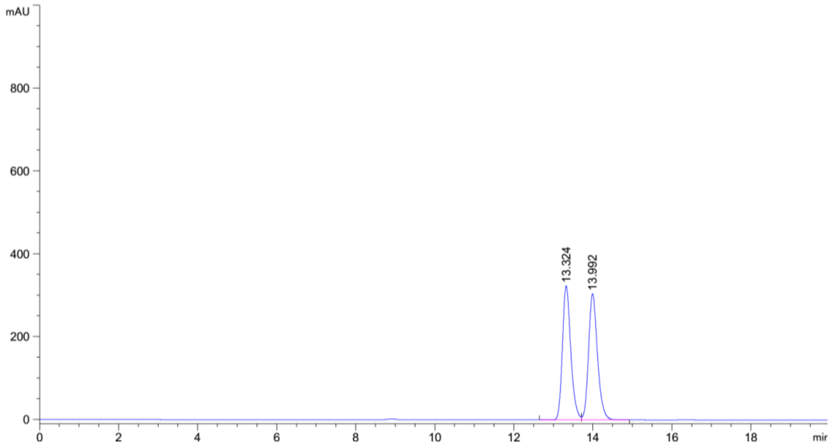

Peak #	RetTime [min]	Type	Width [min]	Area [mAU*s]	Height [mAU]	Area %
1	13.324	BV	0.2246	4758.46631	323.54788	49.8701
2	13.992	VB	0.2391	4783.25293	304.71332	50.1299

Totals : 9541.71924 628.26120

HPLC (R)-3-(3,4-dimethoxyphenyl)-4,5-diphenyl-3,6-dihydro-2H-pyran-3-ol (2h, 95.5 : 4.5 er)

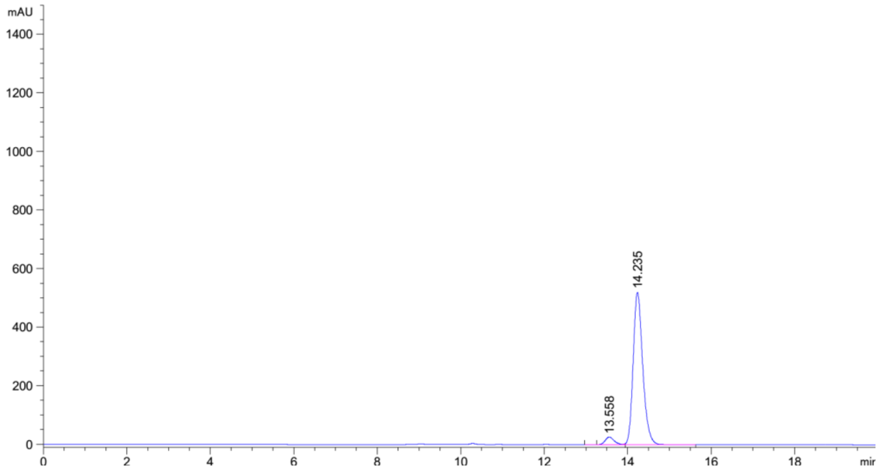

Peak #	RetTime [min]	Type	Width [min]	Area [mAU*s]	Height [mAU]	Area %
1	13.558	VV E	0.2262	389.23523	26.22248	4.4794
2	14.235	VB R	0.2430	8300.12695	520.62964	95.5206

Totals : 8689.36218 546.85212

^{1}HNMR(400MHz,CDCl$_3$)(R)-3-(3-isopropoxyphenyl)-4,5-diphenyl-3,6-dihydro-2H-pyran-3-ol (2i)

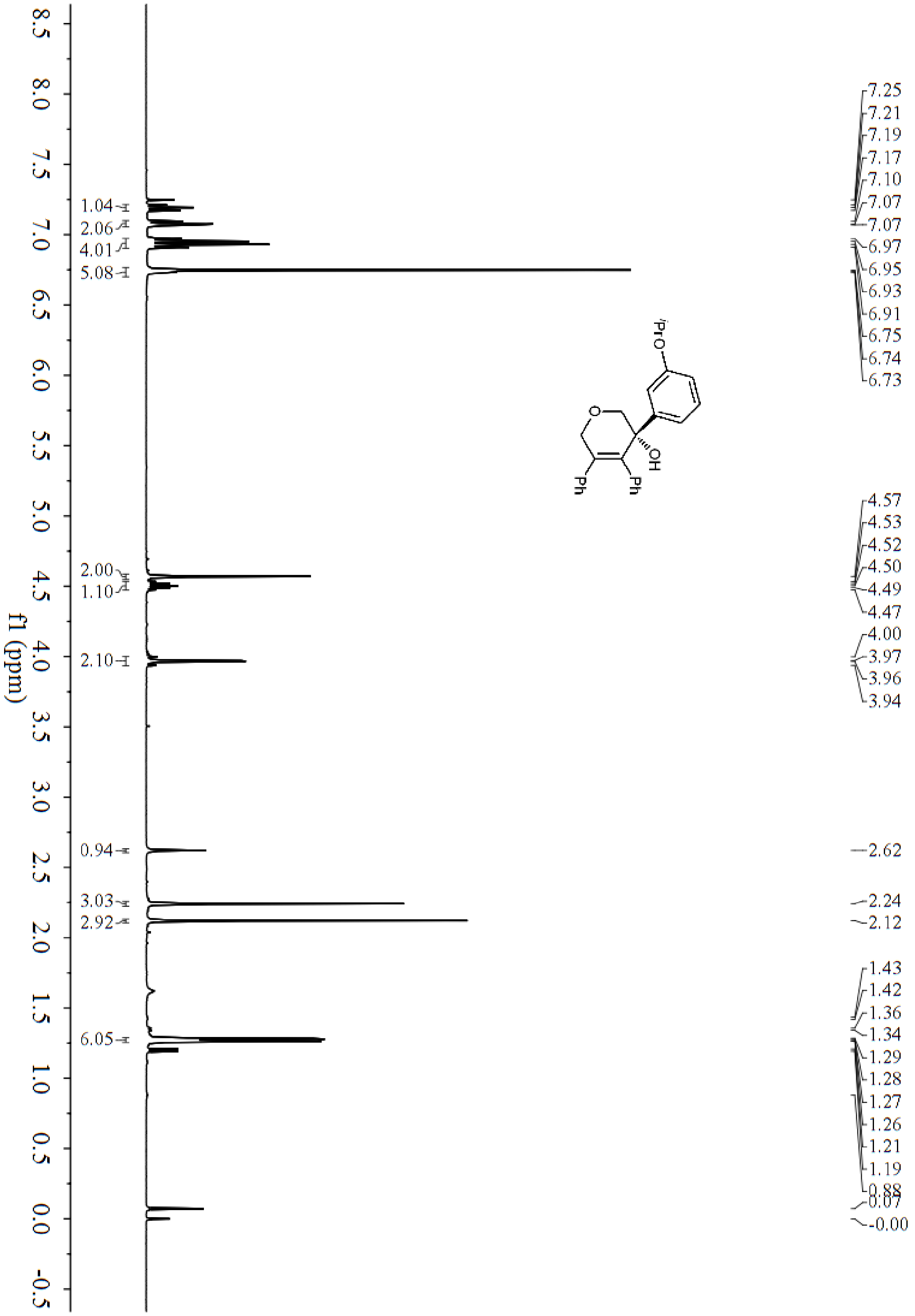

^{13}CNMR(100MHz,$CDCl_3$)(R)-3-(3-isopropoxyphenyl)-4,5-diphenyl-3,6-dihydro-2H-pyran-3-ol (2i)

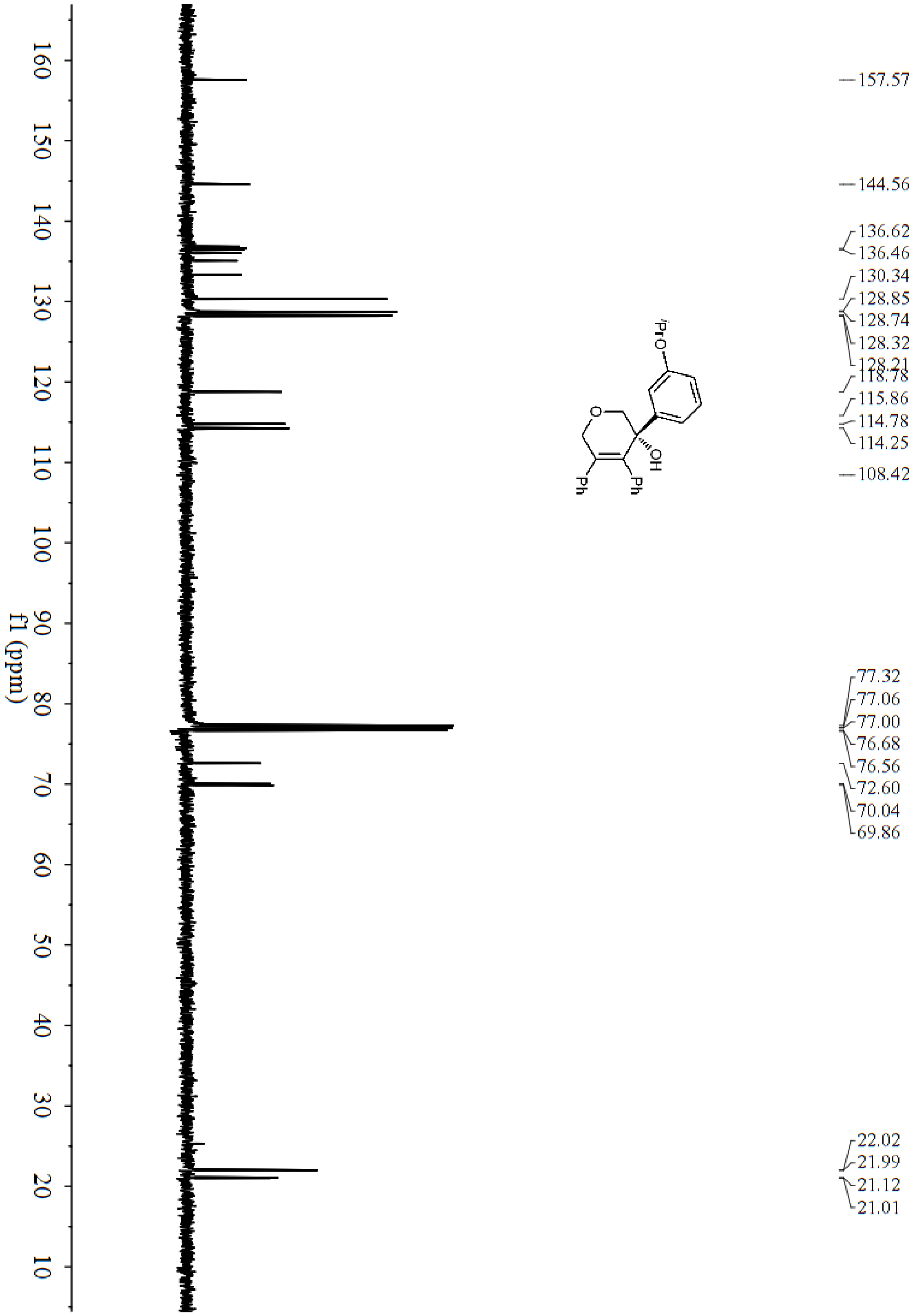

HPLC (R)-3-(3-isopropoxyphenyl)-4,5-diphenyl-3,6-dihydro-2H-pyran-3-ol (2i, Racemic)

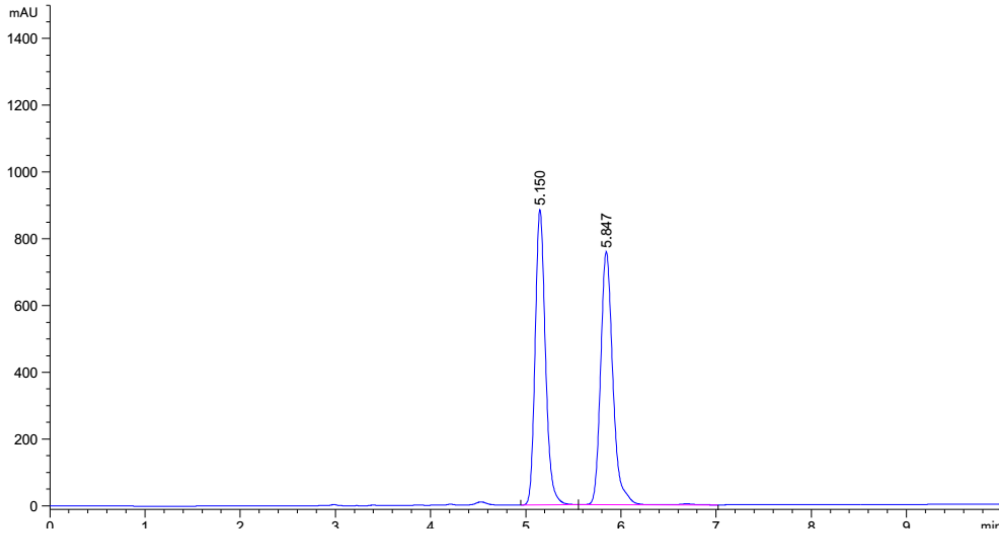

Peak #	RetTime [min]	Type	Width [min]	Area [mAU*s]	Height [mAU]	Area %
1	5.150	BB	0.1152	6617.44922	885.45374	49.4281
2	5.847	BV R	0.1357	6770.58447	757.61890	50.5719

Totals : 1.33880e4 1643.07263

HPLC (R)-3-(3-isopropoxyphenyl)-4,5-diphenyl-3,6-dihydro-2H-pyran-3-ol (2i, 96:4 er)

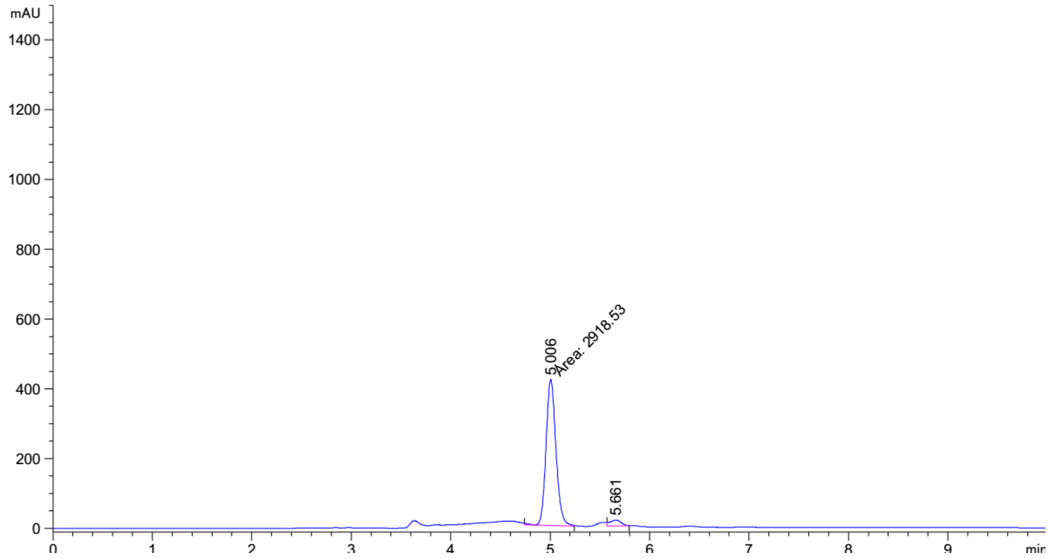

Peak #	RetTime [min]	Type	Width [min]	Area [mAU*s]	Height [mAU]	Area %
1	5.006	MM	0.1160	2918.52539	419.45670	95.8029
2	5.661	VB	0.1171	127.86050	16.55579	4.1971

Totals : 3046.38589 436.01249

^{1}H NMR (400 MHz, $CDCl_3$) (R)-3-phenyl-4,5-di-p-tolyl-3,6-dihydro-2H-pyran-3-ol (2j)

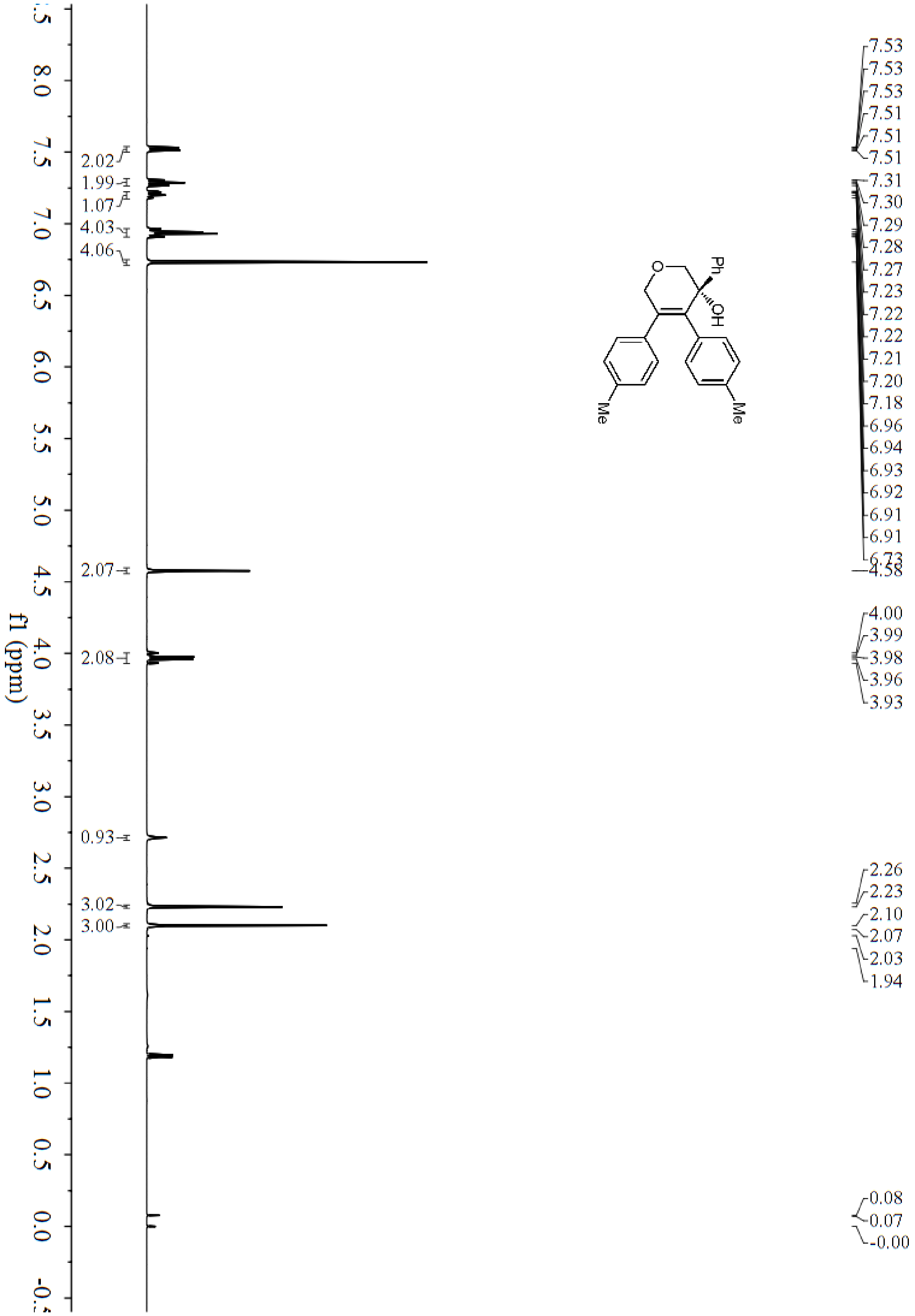

^{13}C NMR (100 MHz, $CDCl_3$) (R)-3-phenyl-4,5-di-p-tolyl-3,6-dihydro-2H-pyran-3-ol (2j)

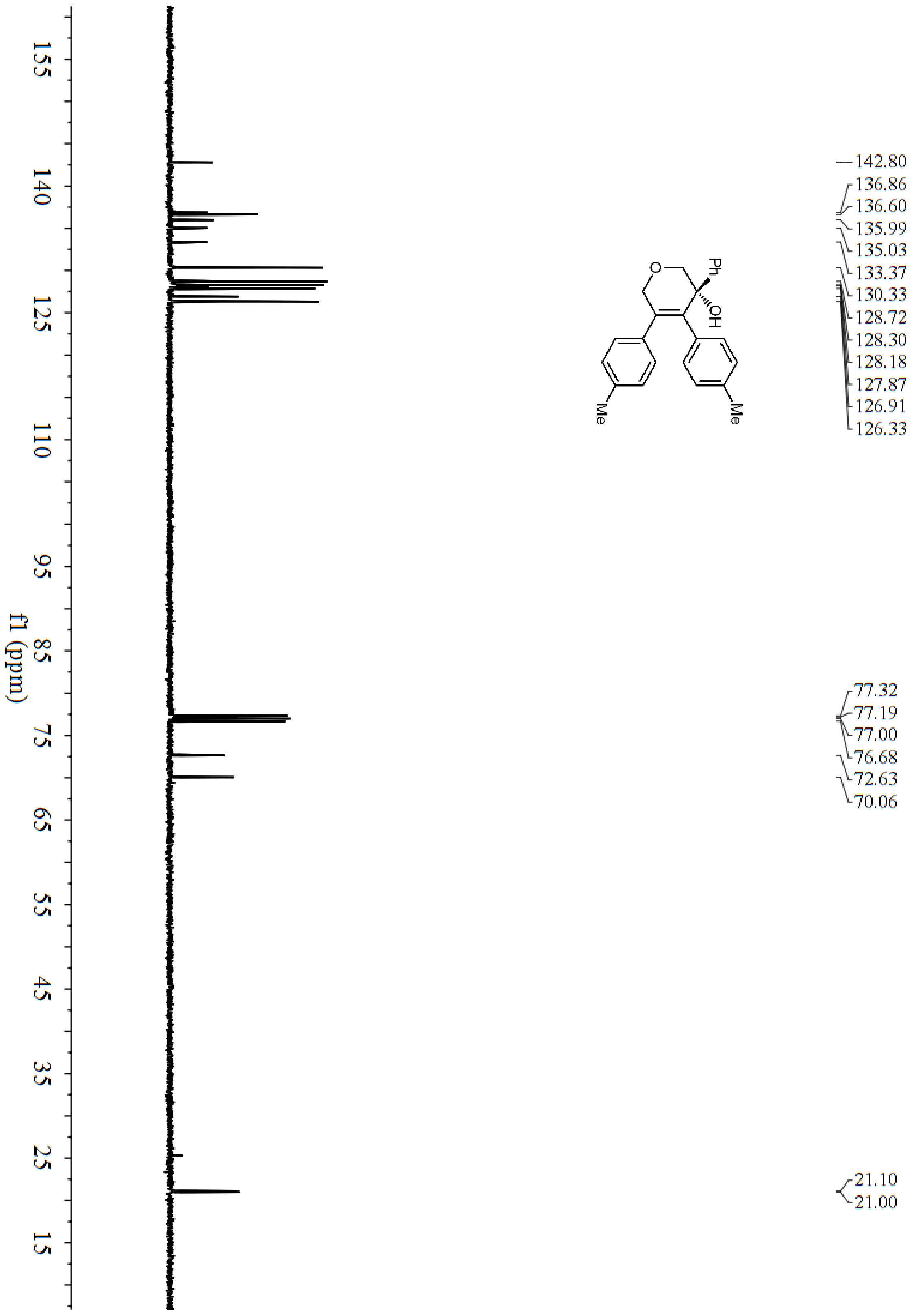

HPLC (R)-3-phenyl-4,5-di-p-tolyl-3,6-dihydro-2H-pyran-3-ol (2j, Racemic)

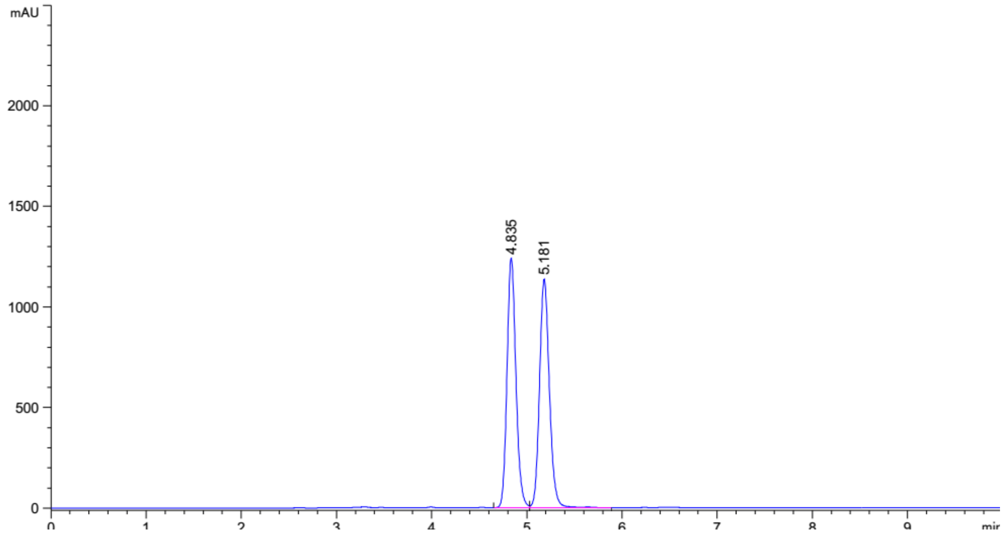

Peak #	RetTime [min]	Type	Width [min]	Area [mAU*s]	Height [mAU]	Area %
1	4.835	BV	0.0997	8043.72510	1240.13586	49.6085
2	5.181	VV R	0.1097	8170.67969	1136.28943	50.3915

Totals : 1.62144e4 2376.42529

HPLC (R)-3-phenyl-4,5-di-p-tolyl-3,6-dihydro-2H-pyran-3-ol (2j, 97 : 3 er)

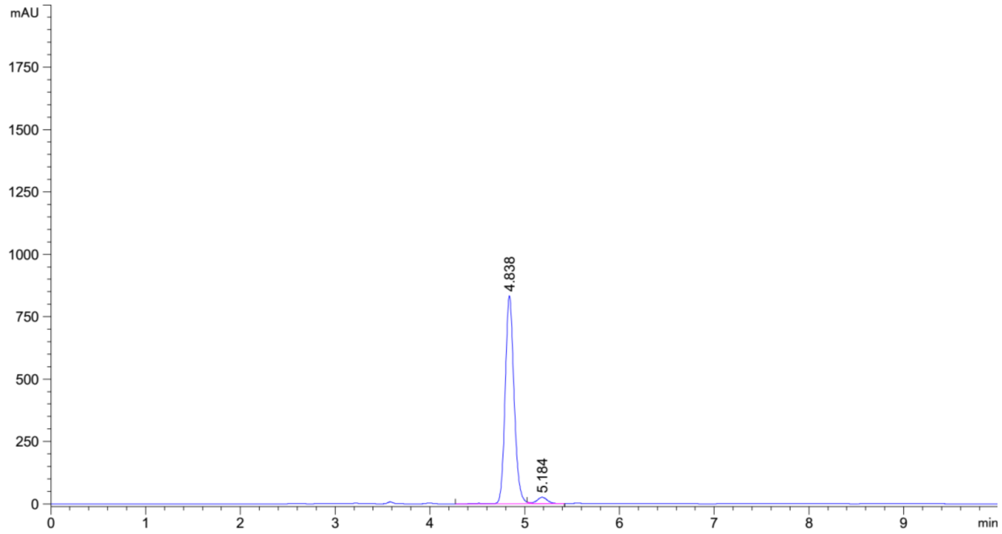

Peak #	RetTime [min]	Type	Width [min]	Area [mAU*s]	Height [mAU]	Area %
1	4.838	VV R	0.0999	5421.06152	833.47021	96.7171
2	5.184	VB E	0.1108	184.00722	25.30326	3.2829

Totals : 5605.06874 858.77348

^{1}HNMR(400MHz,$CDCl_3$)

(R)-3-(3-isopropoxyphenyl)-4,5-di-p-tolyl-3,6-dihydro-2H-pyran-3-ol (2k)

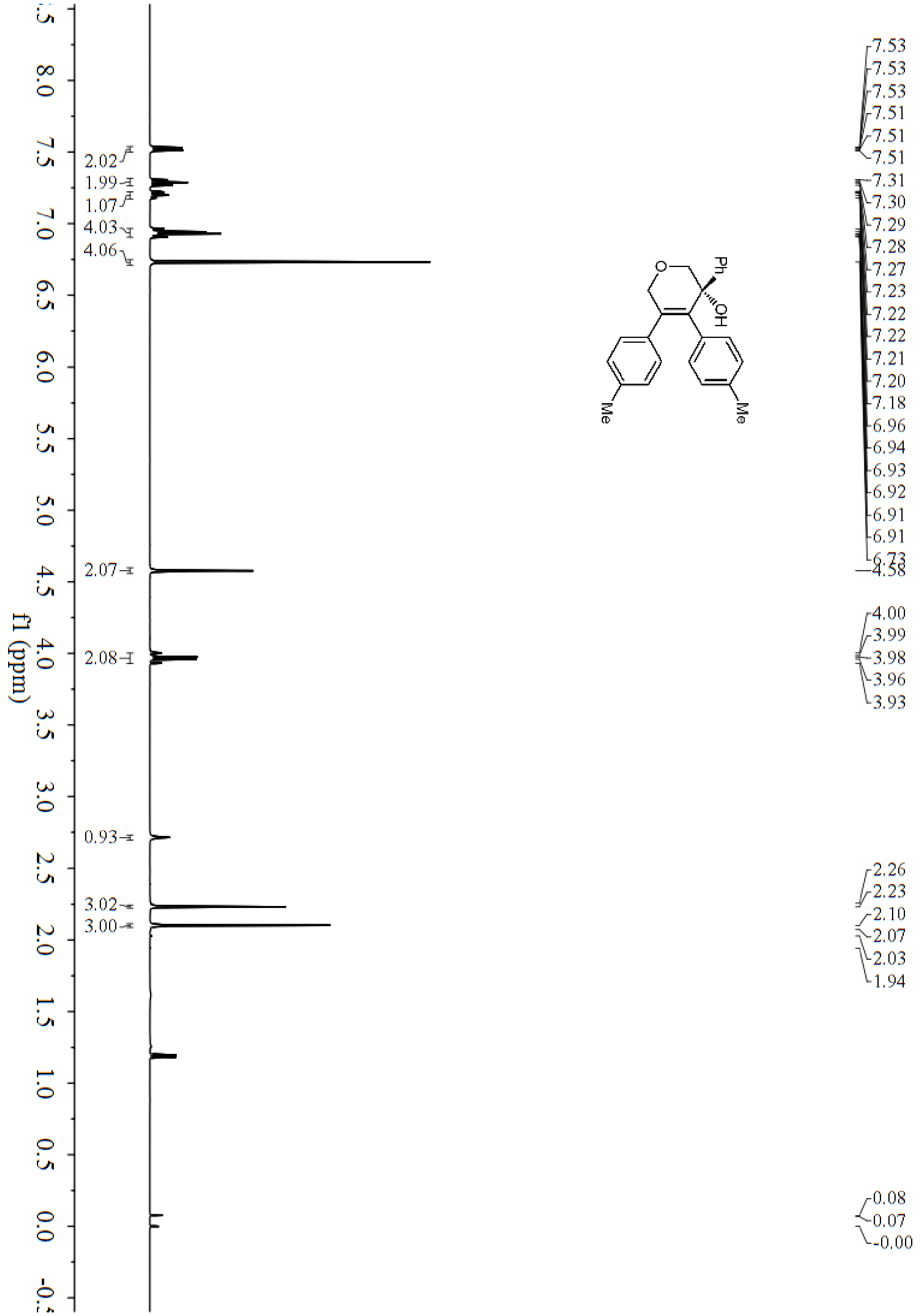

^{13}CNMR(100MHz,$CDCl_3$)

(R)-3-(3-isopropoxyphenyl)-4,5-di-p-tolyl-3,6-dihydro-2H-pyran-3-ol (2k)

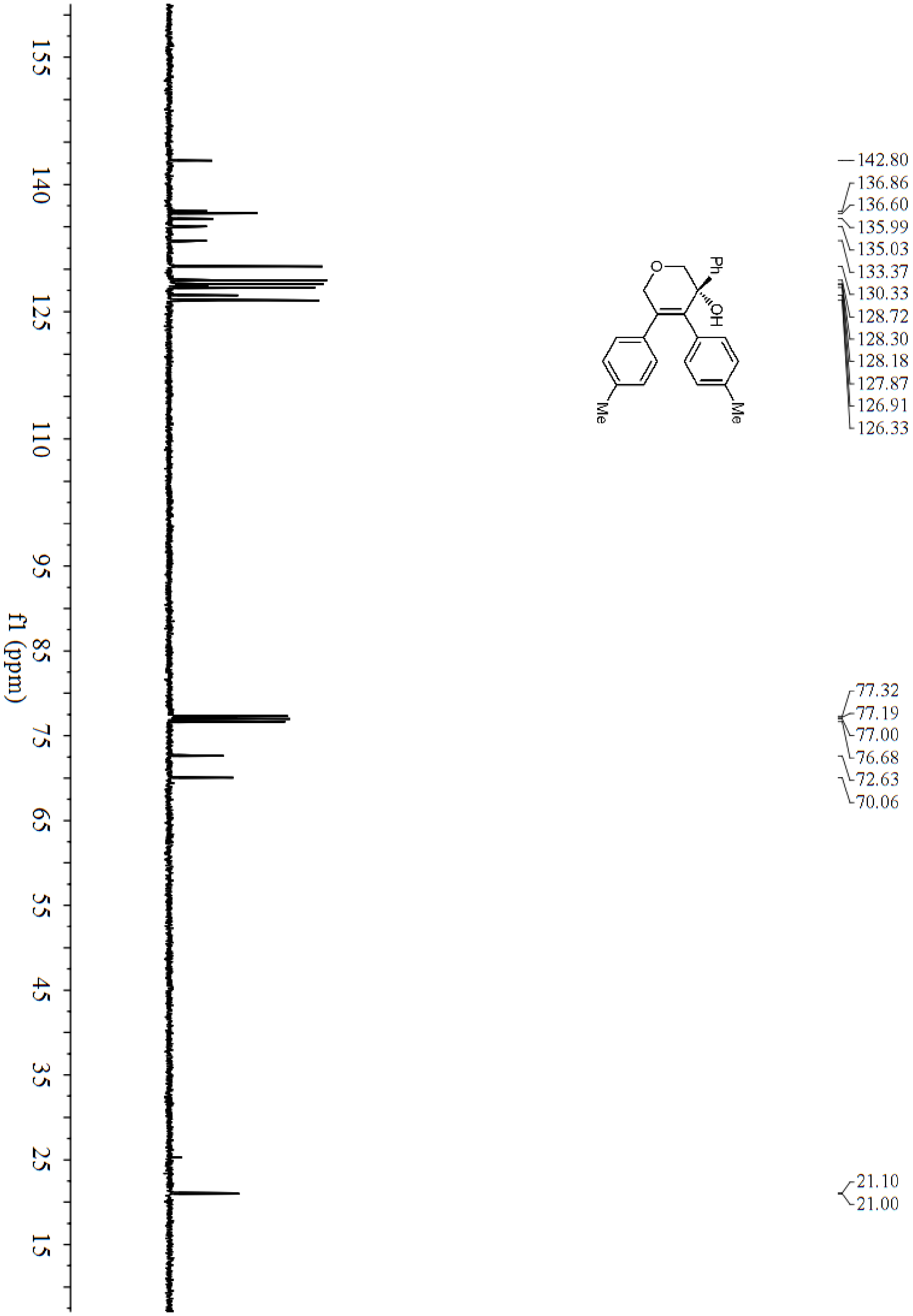

PHLC (R)-3-(3-isopropoxyphenyl)-4,5-di-p-tolyl-3,6-dihydro-2H-pyran-3-ol (2k, Racemic)

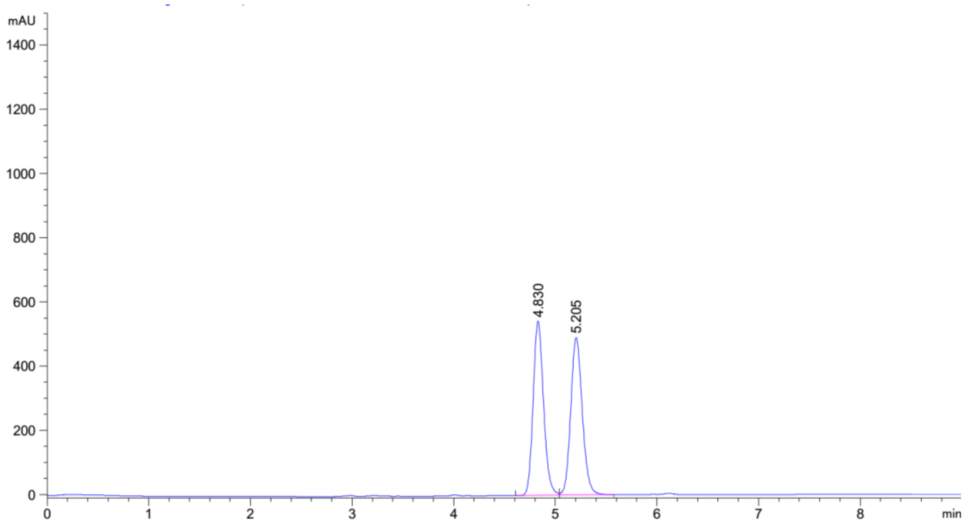

Peak #	RetTime [min]	Type	Width [min]	Area [mAU*s]	Height [mAU]	Area %
1	4.830	BV	0.1091	3866.78564	542.74298	49.8830
2	5.205	VB	0.1224	3884.92065	490.60883	50.1170

Totals : 7751.70630 1033.35181

PHLC (R)-3-(3-isopropoxyphenyl)-4,5-di-p-tolyl-3,6-dihydro-2H-pyran-3-ol (2k, 96 : 4 er)

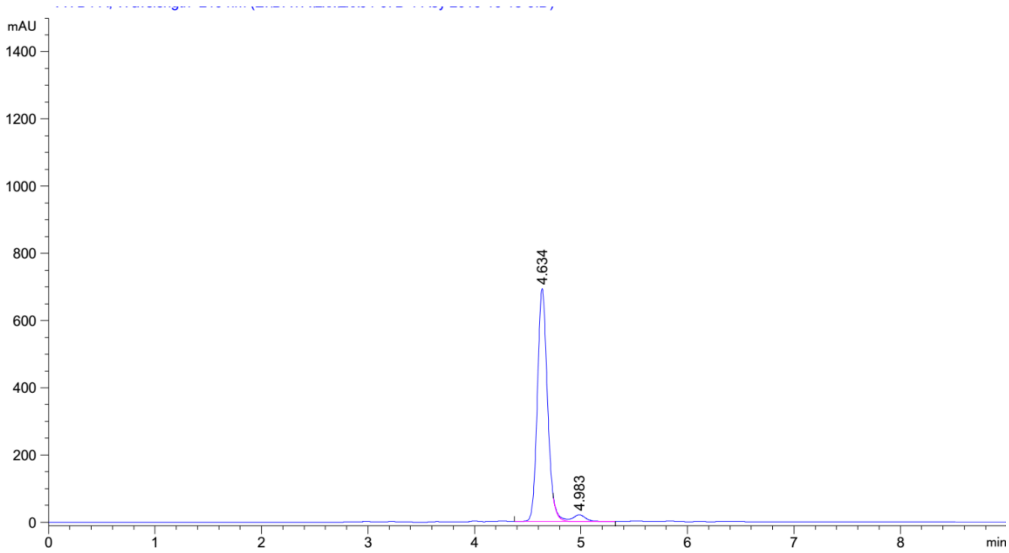

Peak #	RetTime [min]	Type	Width [min]	Area [mAU*s]	Height [mAU]	Area %
1	4.634	BV R	0.1013	4587.54102	692.82184	95.8864
2	4.983	VB E	0.1403	196.80942	20.26716	4.1136

Totals : 4784.35043 713.08900

^{1}H NMR (400 MHz, $CDCl_3$)

(R)-4,5-bis(4-methoxyphenyl)-3-phenyl-3,6-dihydro-2H-pyran-3-ol (2l)

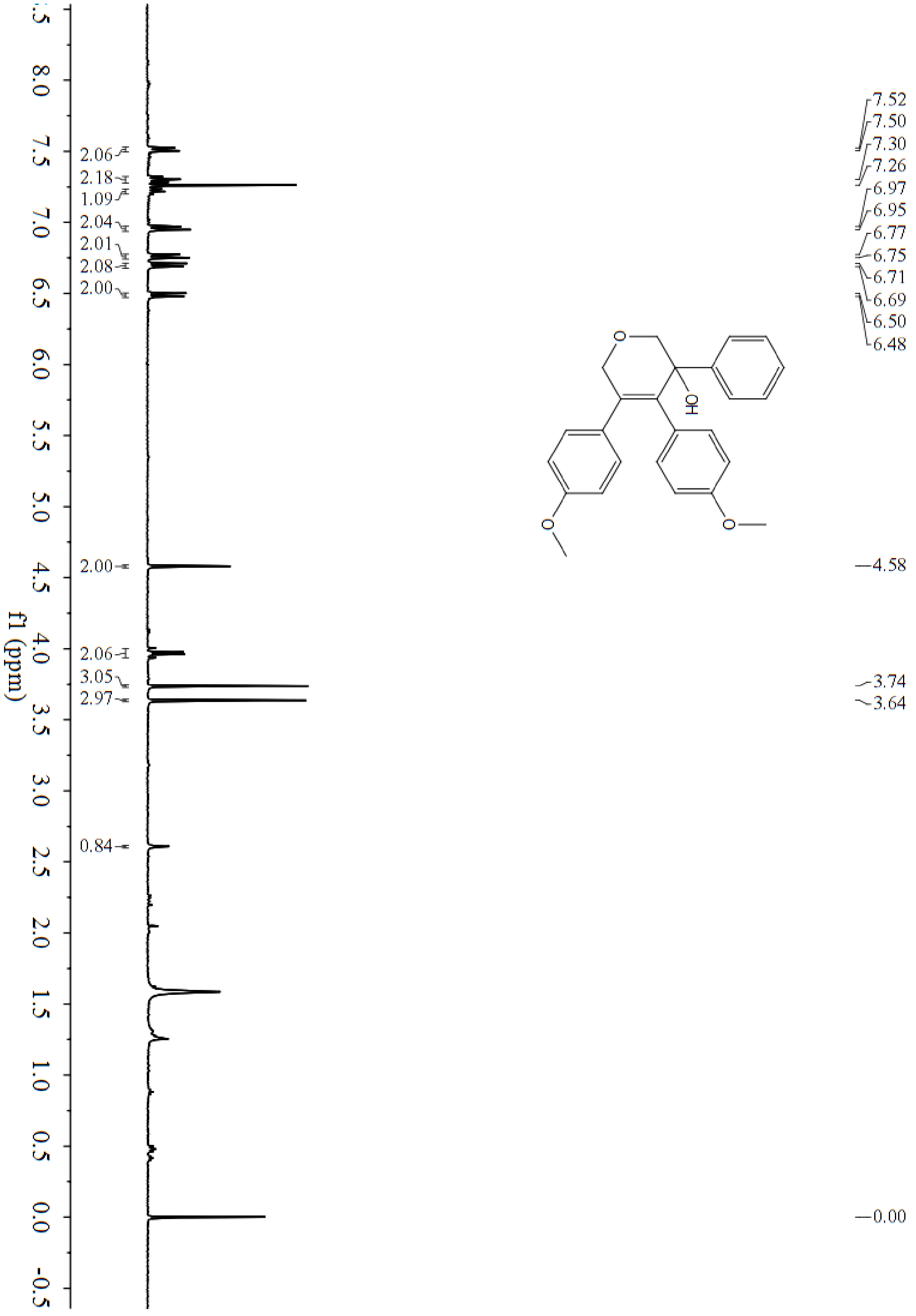

^{13}C NMR (100 MHz, $CDCl_3$)

(R)-4,5-bis(4-methoxyphenyl)-3-phenyl-3,6-dihydro-2H-pyran-3-ol (2l)

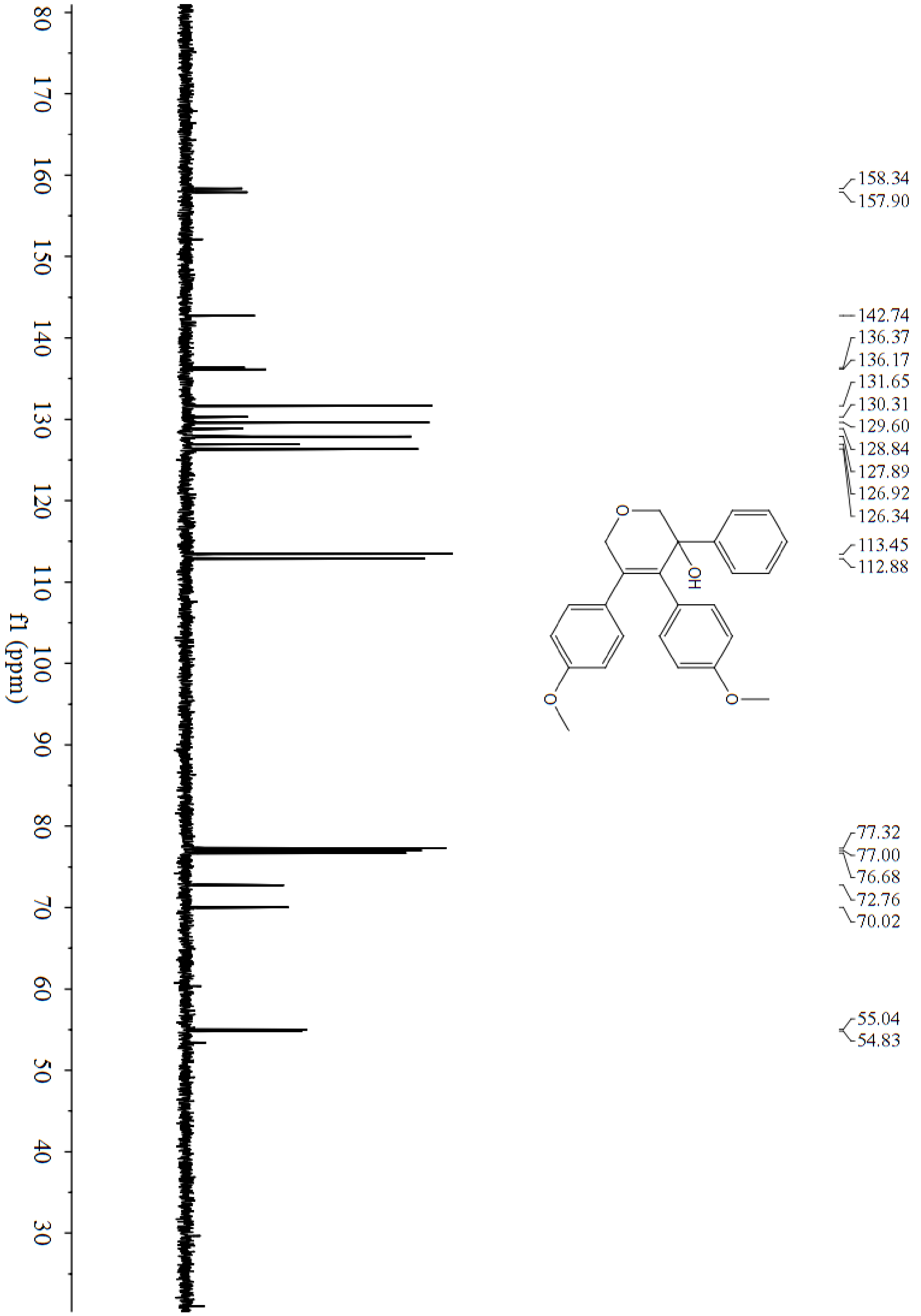

HPLC (R)-4,5-bis(4-methoxyphenyl)-3-phenyl-3,6-dihydro-2H-pyran-3-ol (2l, Racemic)

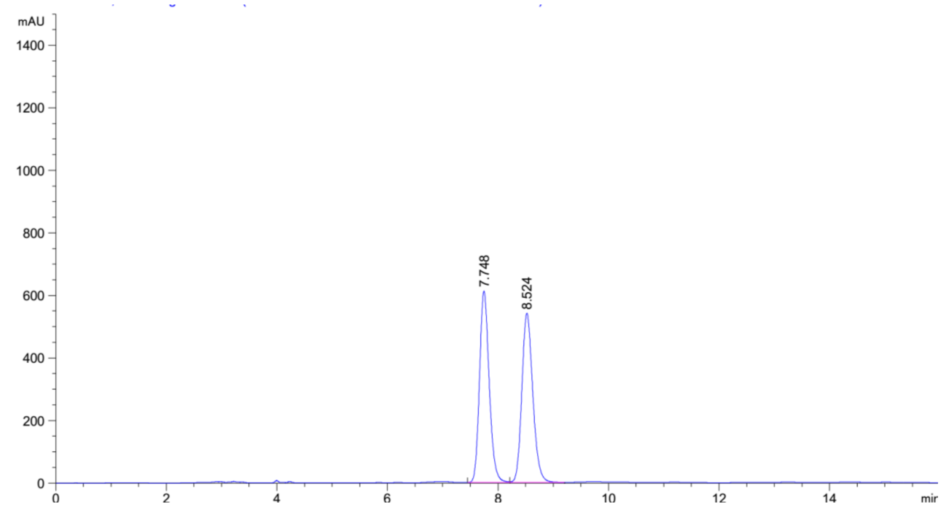

Peak #	RetTime [min]	Type	Width [min]	Area [mAU*s]	Height [mAU]	Area %
1	7.748	BV	0.1868	7433.05615	611.55951	49.9952
2	8.524	VB	0.2113	7434.47607	540.91547	50.0048

Totals : 1.48675e4 1152.47498

HPLC (R)-4,5-bis(4-methoxyphenyl)-3-phenyl-3,6-dihydro-2H-pyran-3-ol (2l, 95 : 5 er)

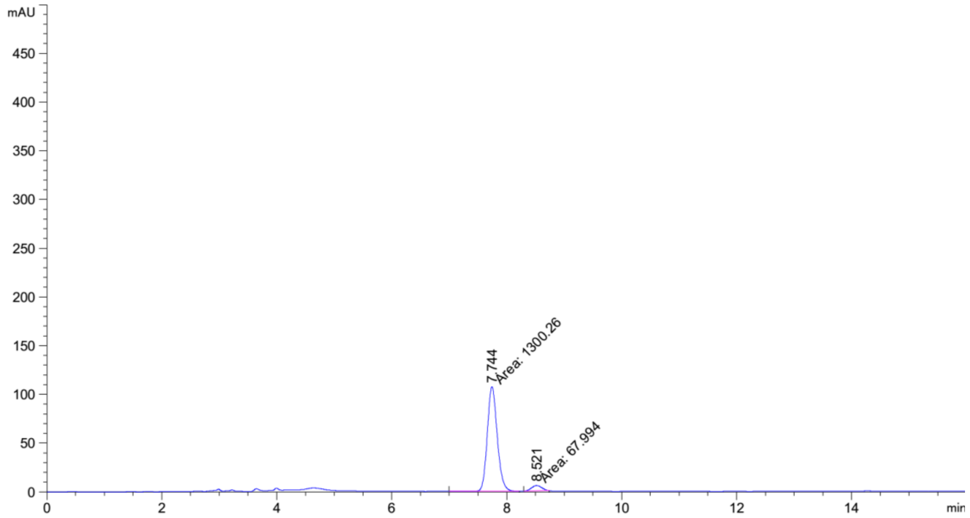

Peak #	RetTime [min]	Type	Width [min]	Area [mAU*s]	Height [mAU]	Area %
1	7.744	MM	0.2018	1300.25525	107.37928	95.0306
2	8.521	MM	0.2059	67.99400	5.50448	4.9694

Totals : 1368.24924 112.88376

^{1}HNMR (400MHz,$CDCl_3$)

5-(4-fluorophenyl)-4-(4-methoxyphenyl)-3-phenyl-3,6-dihydro-2H-pyran-3-ol

4-(4-fluorophenyl)-5-(4-methoxyphenyl)-3-phenyl-3,6-dihydro-2H-pyran-3-ol (2o)

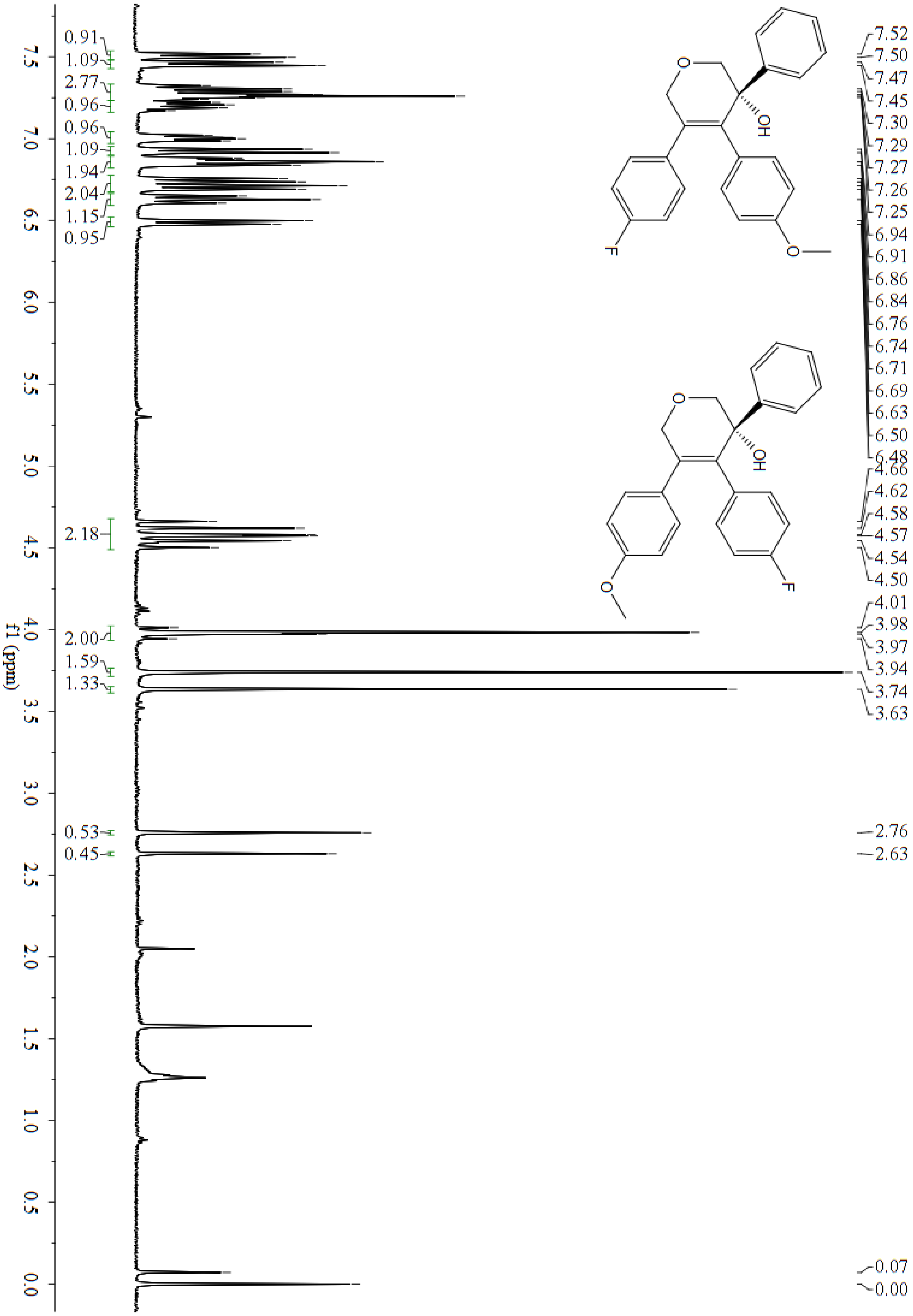

^{1}HNMR (400MHz,$CDCl_3$) 4,5-diethyl-3-phenyl-3,6-dihydro-2H-pyran-3-ol (2p)

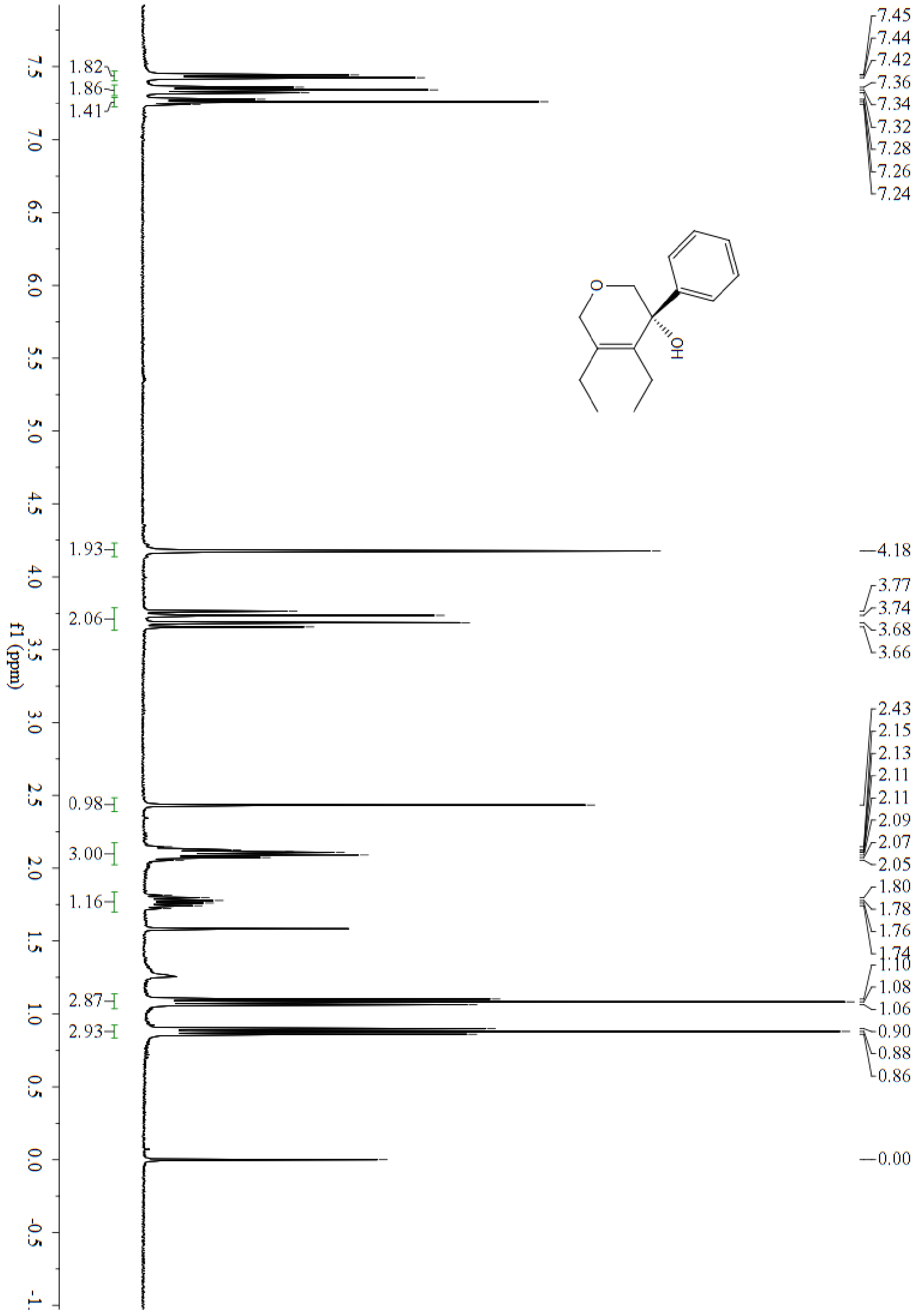

^{1}HNMR (400MHz,$CDCl_3$) 2-phenethyl-3,4,5-triphenyl-3,6-dihydro-2H-pyran-3-ol (4a)

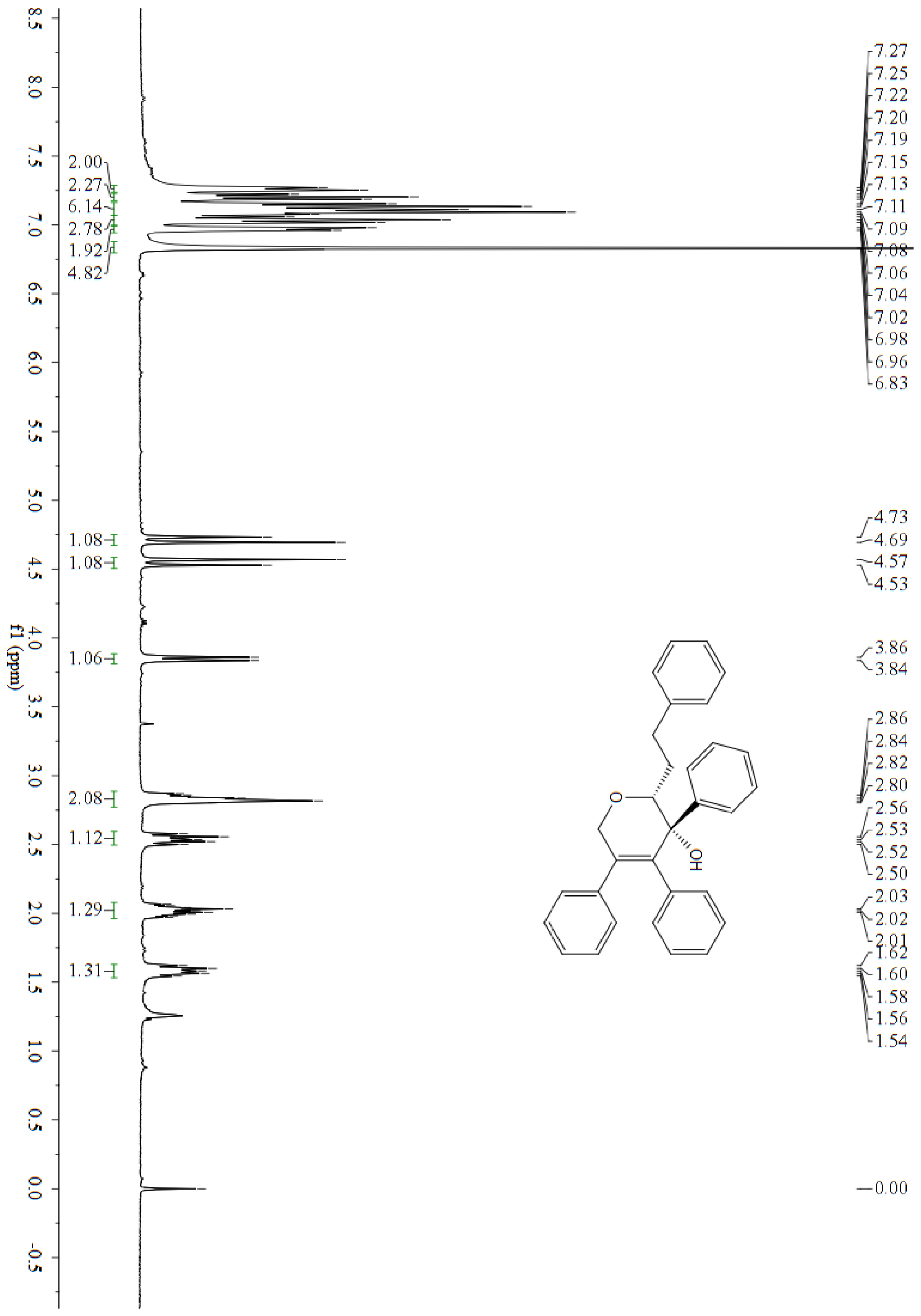

^{13}CNMR(101MHz,$CDCl_3$) 2-phenethyl-3,4,5-triphenyl-3,6-dihydro-2H-pyran-3-ol (4a)

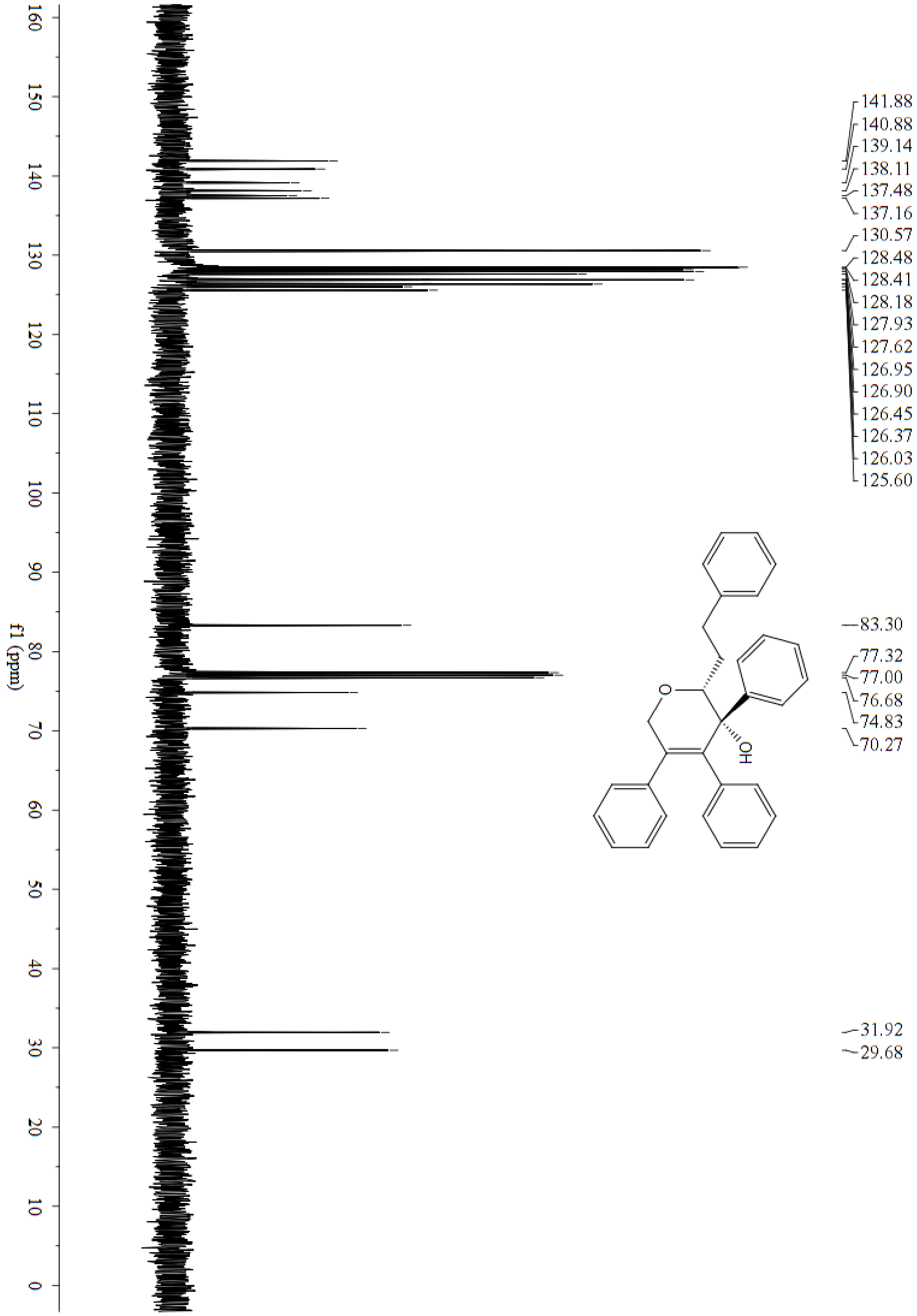

^{1}HNMR (400MHz,$CDCl_3$) 2-hexyl-3,4,5-triphenyl-3,6-dihydro-2H-pyran-3-ol (4b)

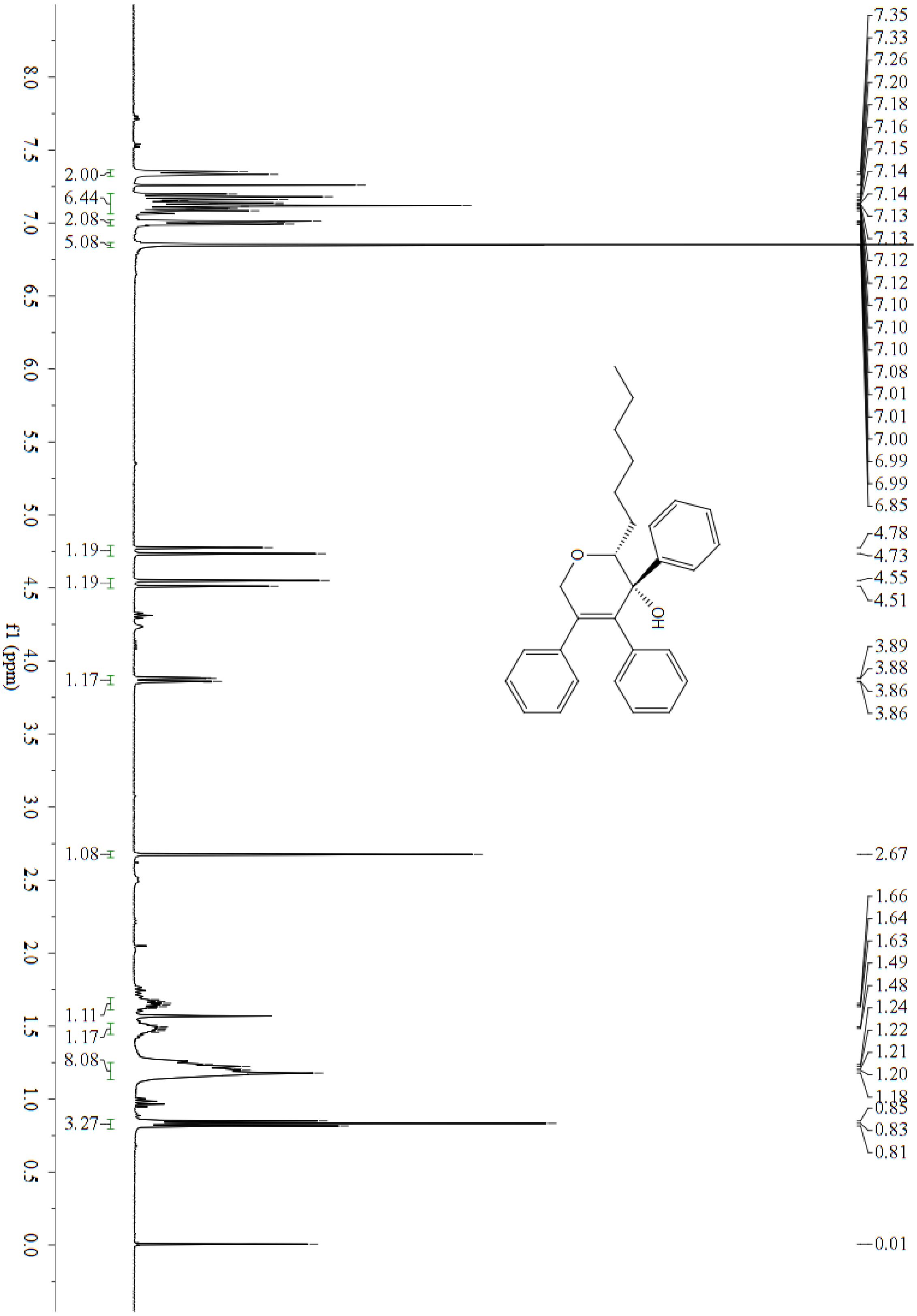

^{13}C NMR (101 MHz, $CDCl_3$) 2-hexyl-3,4,5-triphenyl-3,6-dihydro-2H-pyran-3-ol (4b)

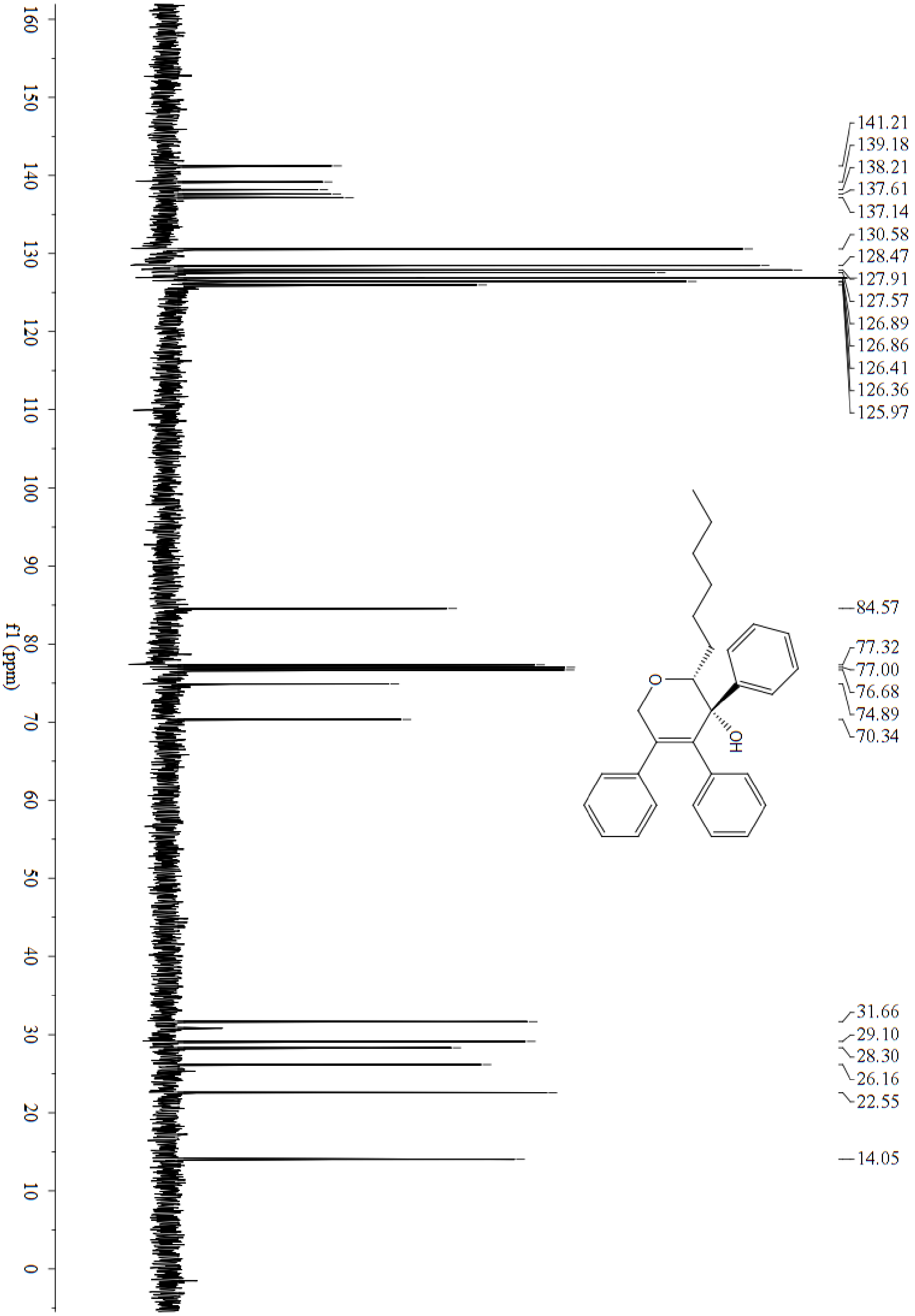

^{1}H NMR (400MHz,$CDCl_3$)

2-(4-chlorobutyl)-3,4,5-triphenyl-3,6-dihydro-2H-pyran-3-ol (4c)

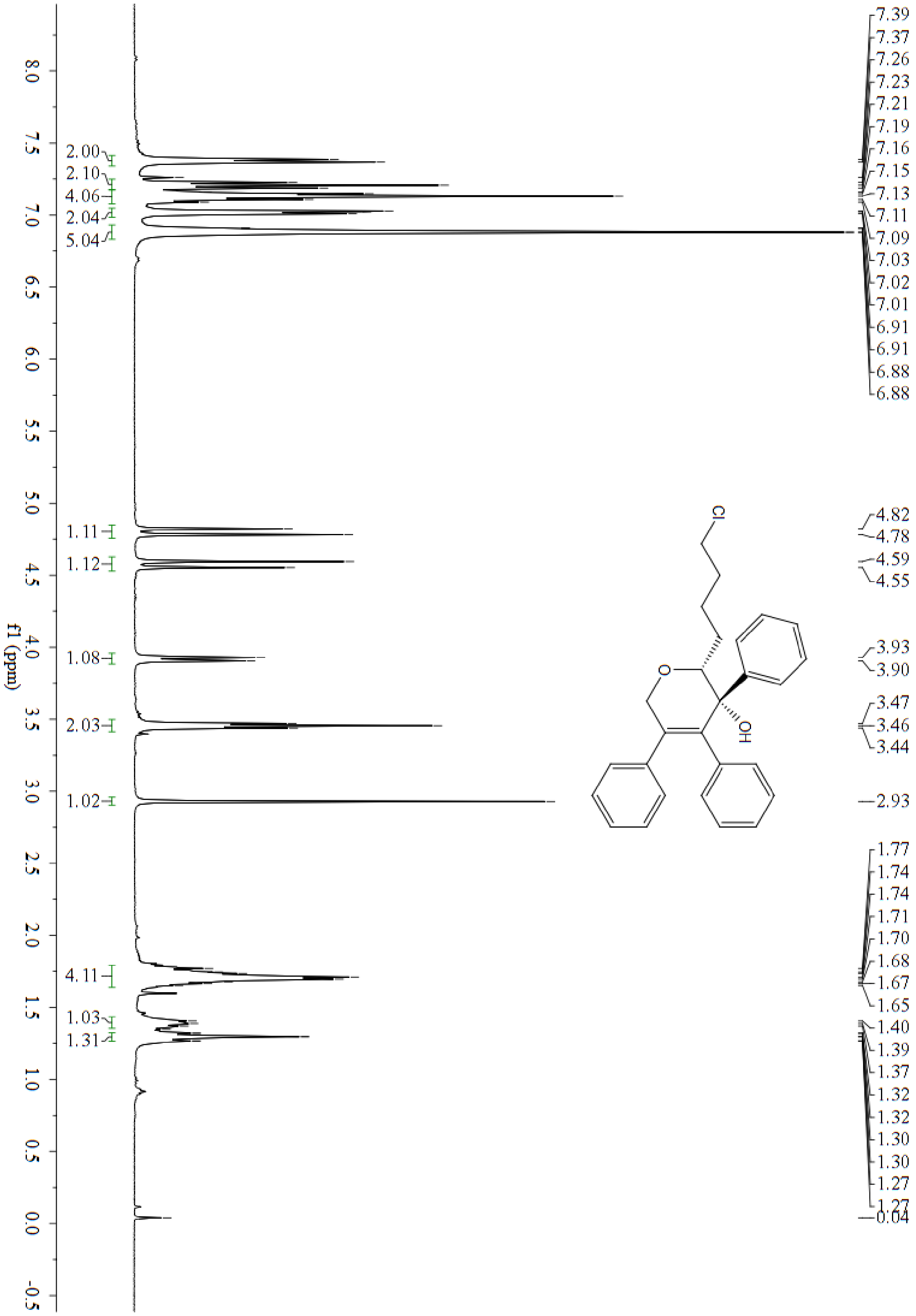

^{13}C NMR (101MHz,$CDCl_3$)

2-(4-chlorobutyl)-3,4,5-triphenyl-3,6-dihydro-2H-pyran-3-ol (4c)

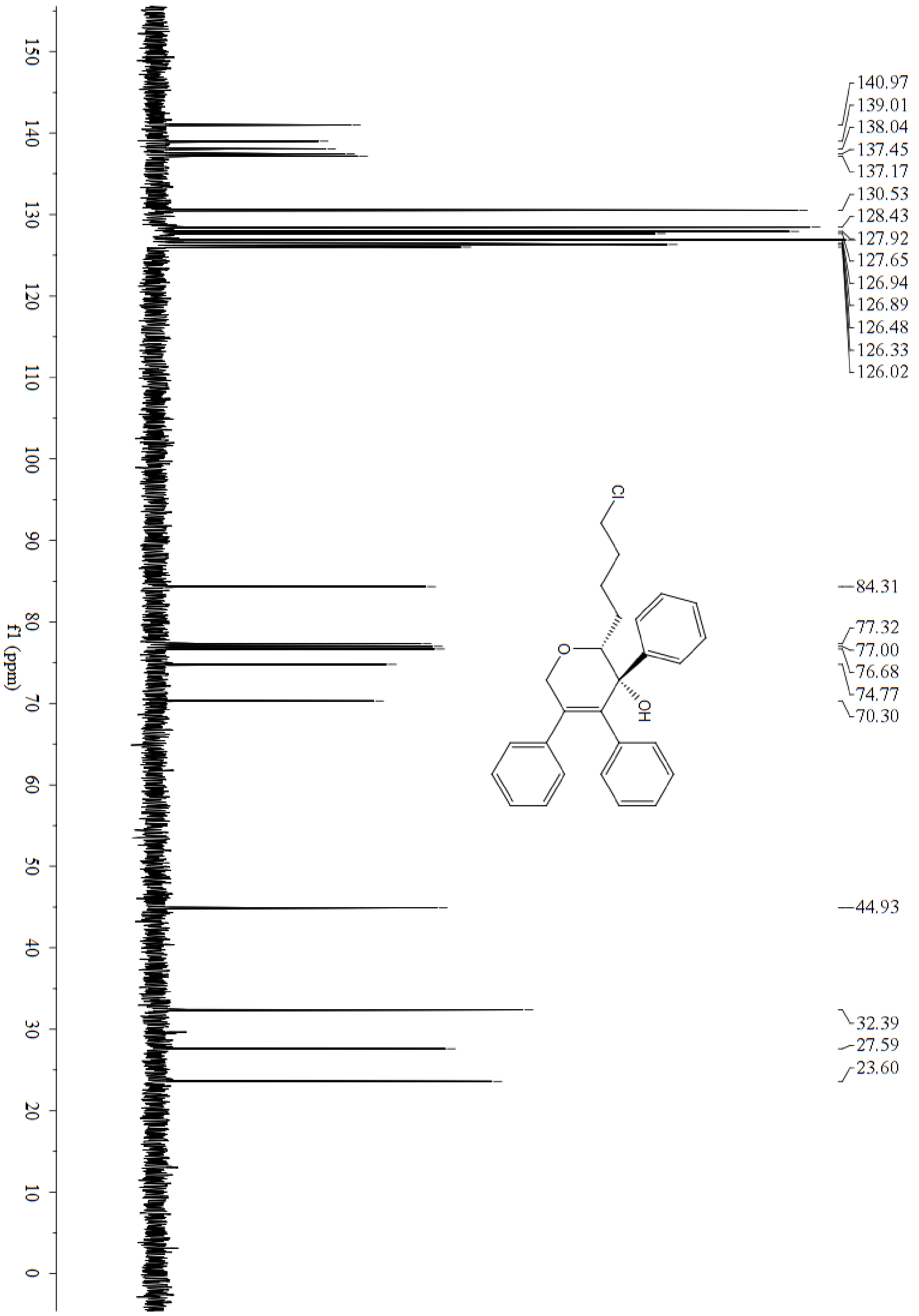

^{1}H NMR (400MHz,CDCl$_3$)

2-(but-3-en-1-yl)-3,4,5-triphenyl-3,6-dihydro-2H-pyran-3-ol (4d)

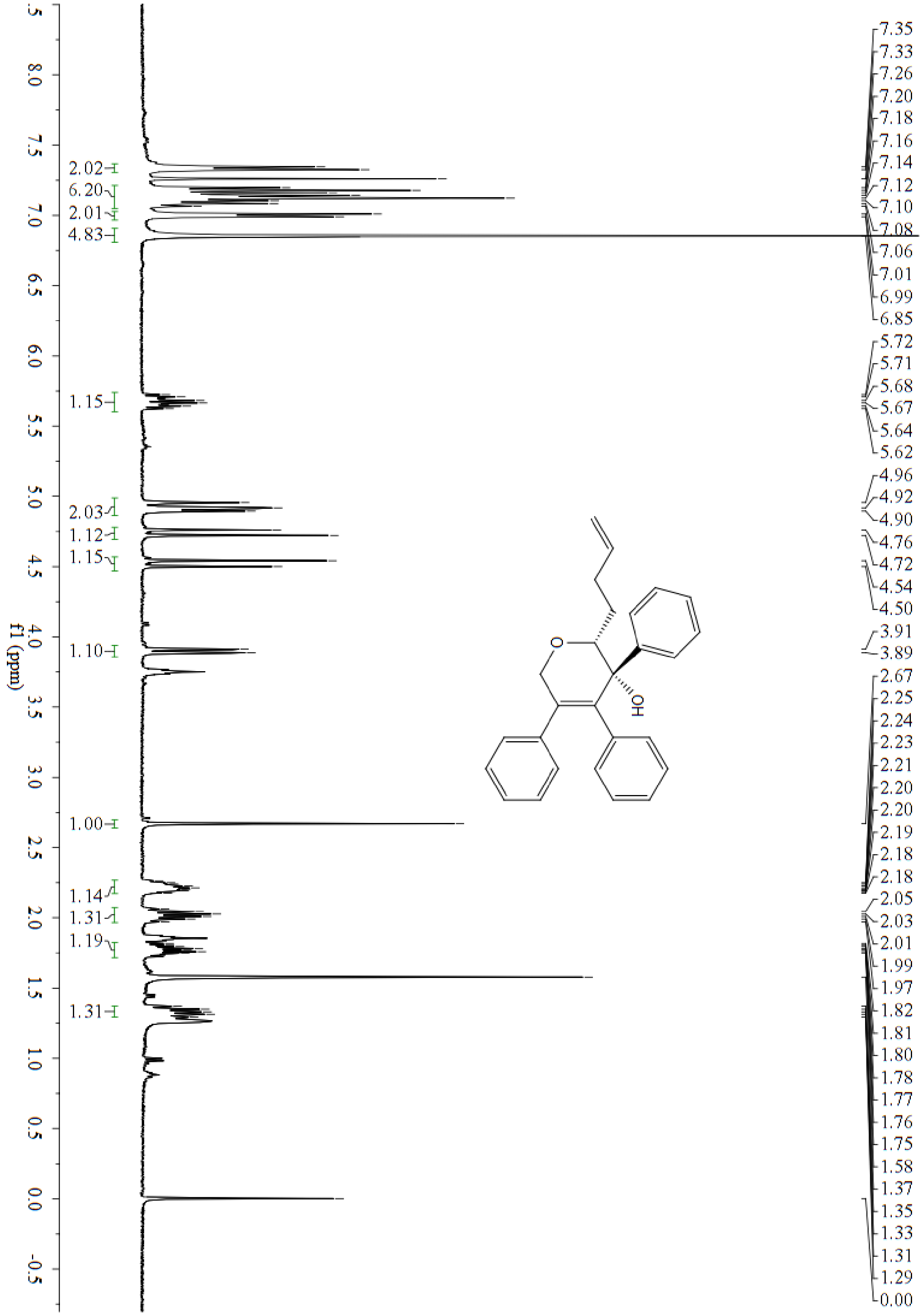

^{13}C NMR (101MHz,CDCl$_3$)

2-(but-3-en-1-yl)-3,4,5-triphenyl-3,6-dihydro-2H-pyran-3-ol (4d)

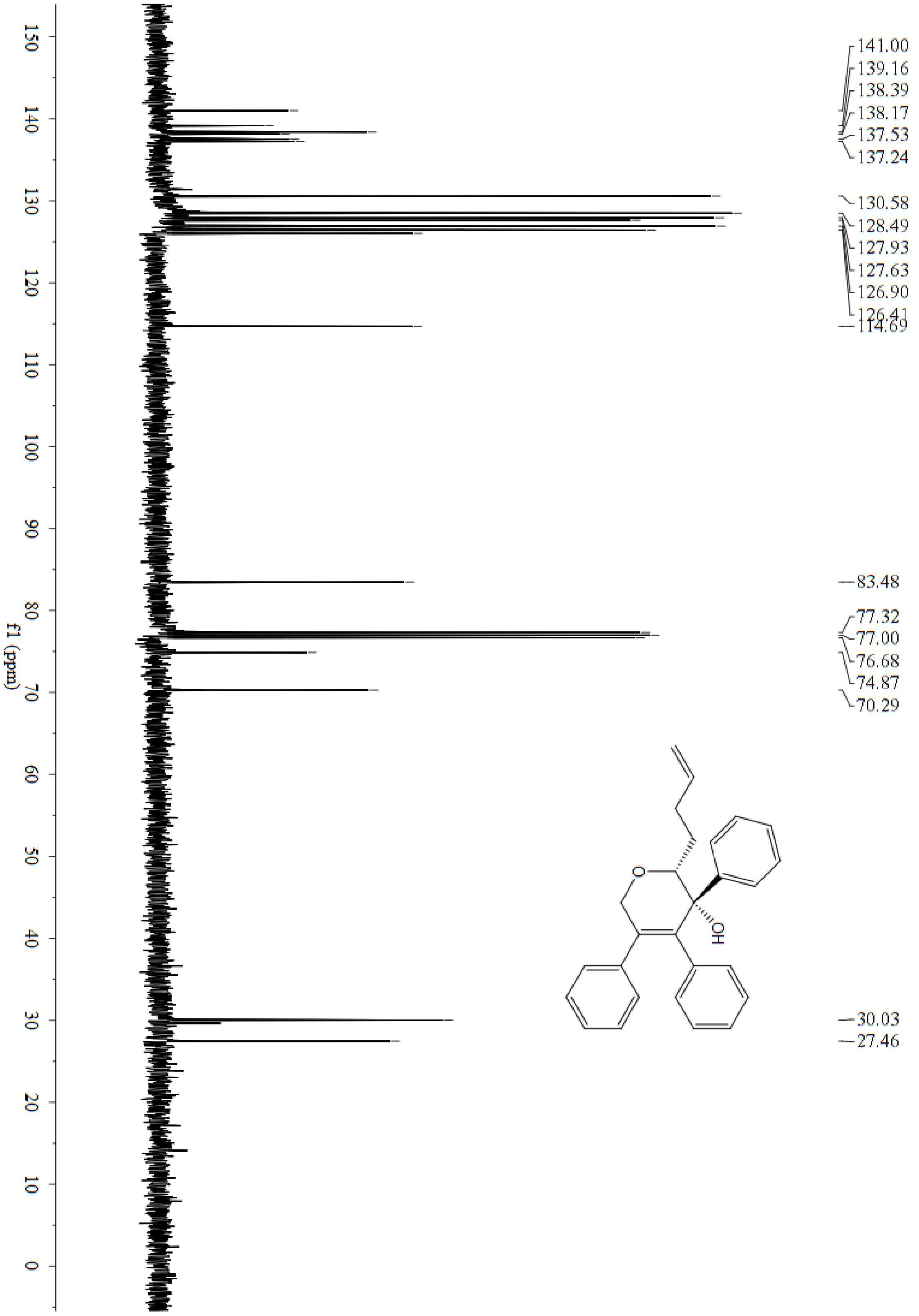

^{1}H NMR (400MHz,CDCl$_3$)

2-cyclopropyl-3,4,5-triphenyl-3,6-dihydro-2H-pyran-3-ol (4e)

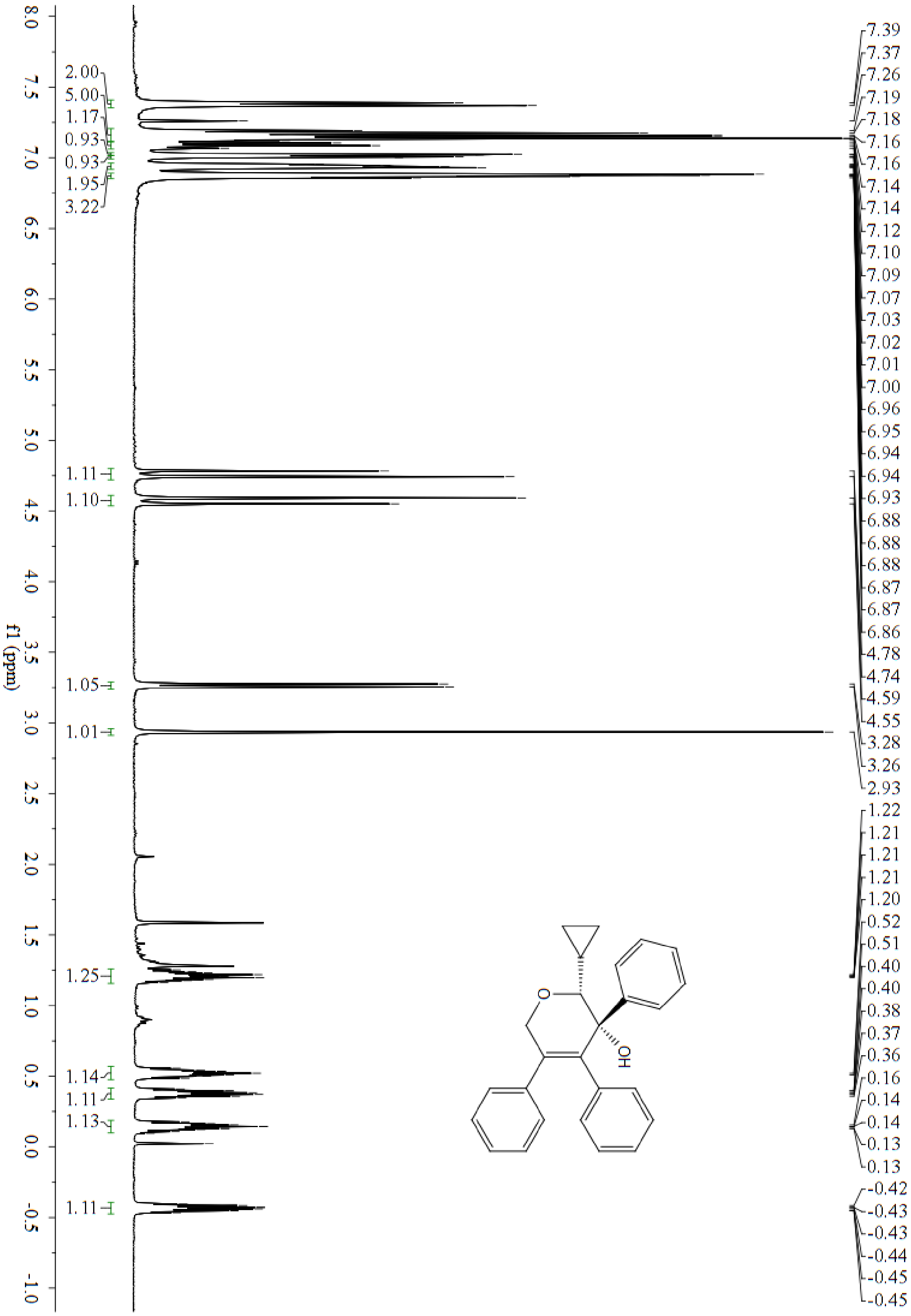

^{13}C NMR (101MHz,$CDCl_3$)

2-cyclopropyl-3,4,5-triphenyl-3,6-dihydro-2H-pyran-3-ol (4e)

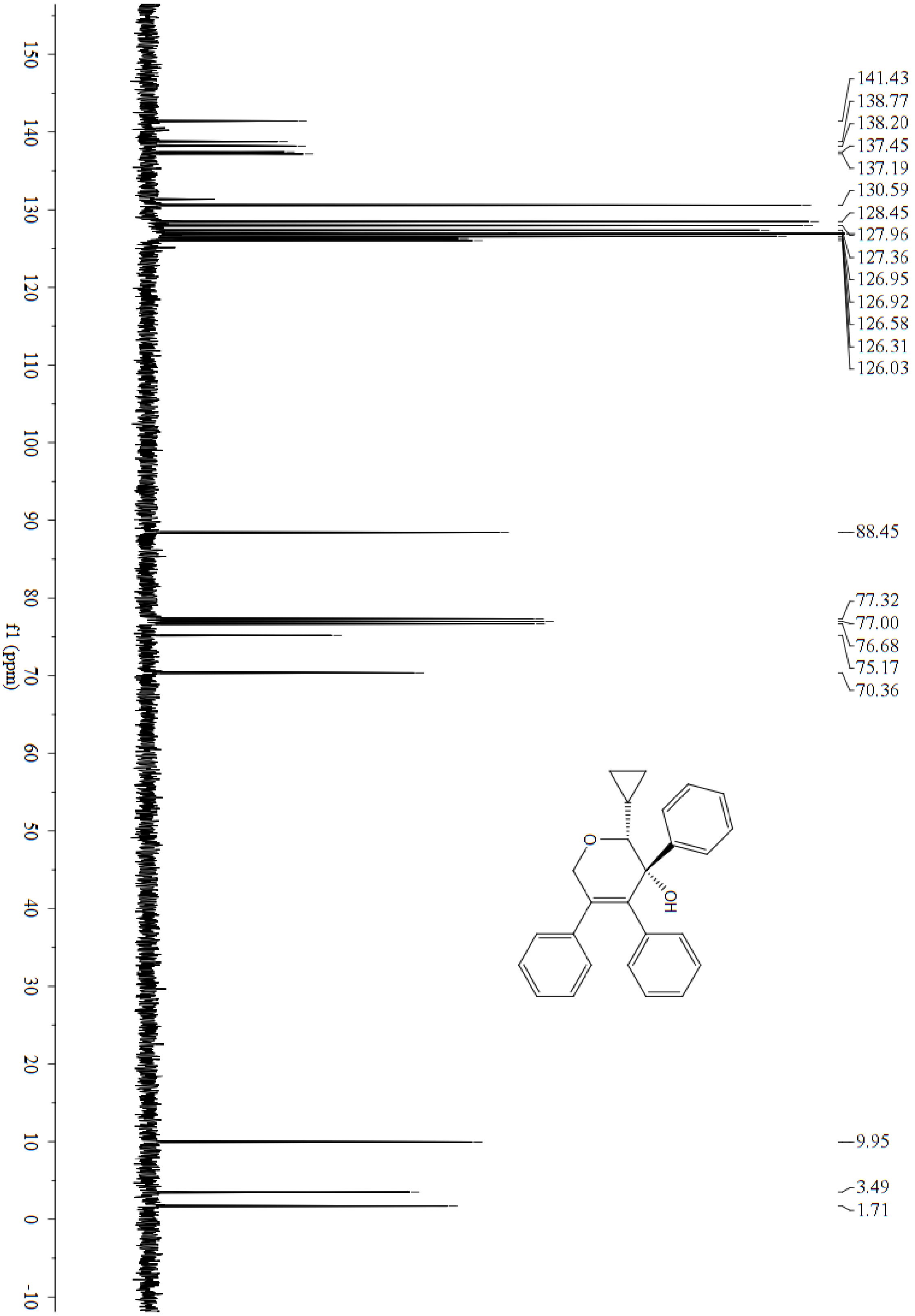

^{1}H NMR (400MHz,$CDCl_3$) 2-cyclohexyl-3,4,5-triphenyl-3,6-dihydro-2H-pyran-3-ol(4f)

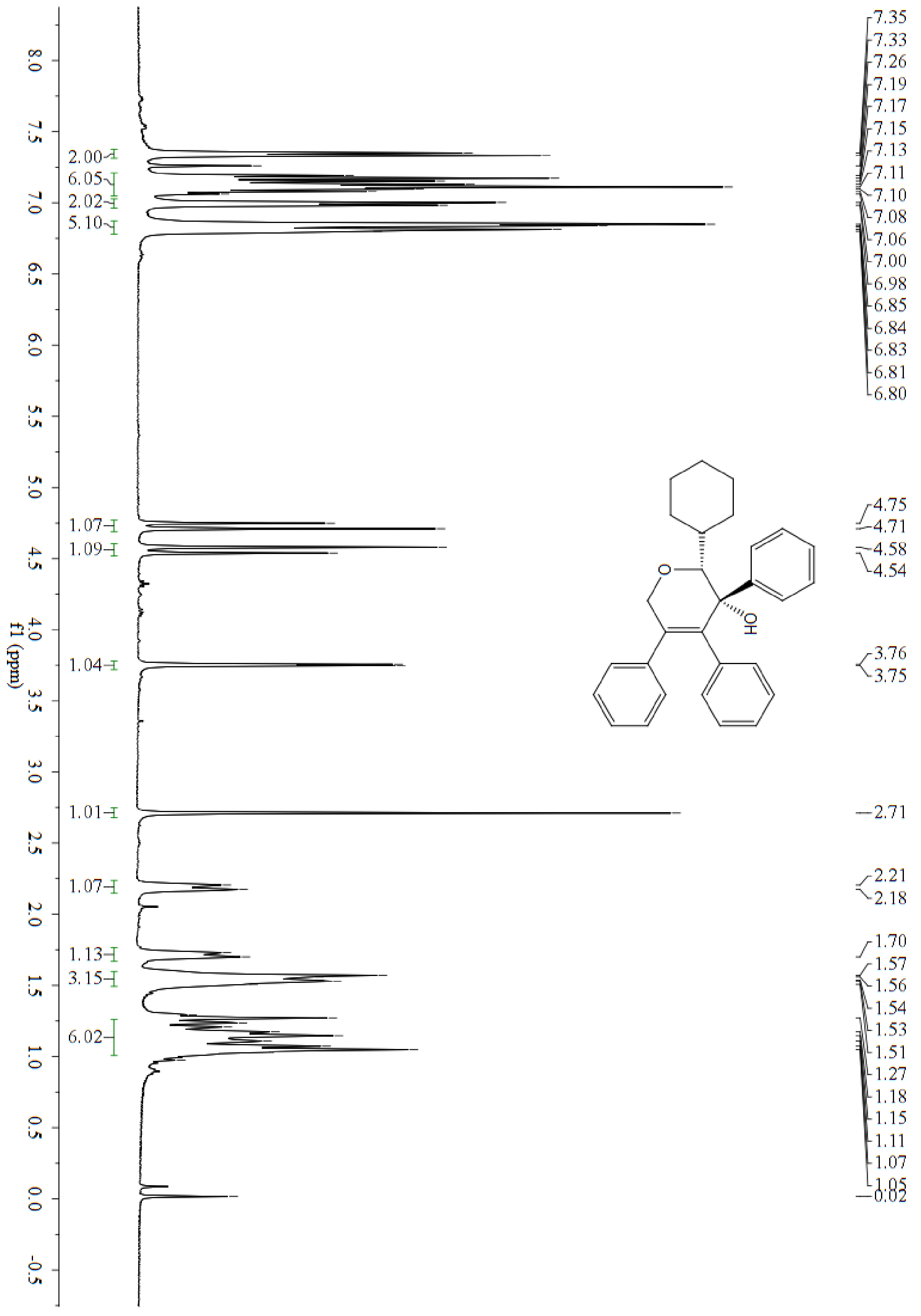

^{13}C NMR (101MHz,$CDCl_3$) 2-cyclohexyl-3,4,5-triphenyl-3,6-dihydro-2H-pyran-3-ol(4f)

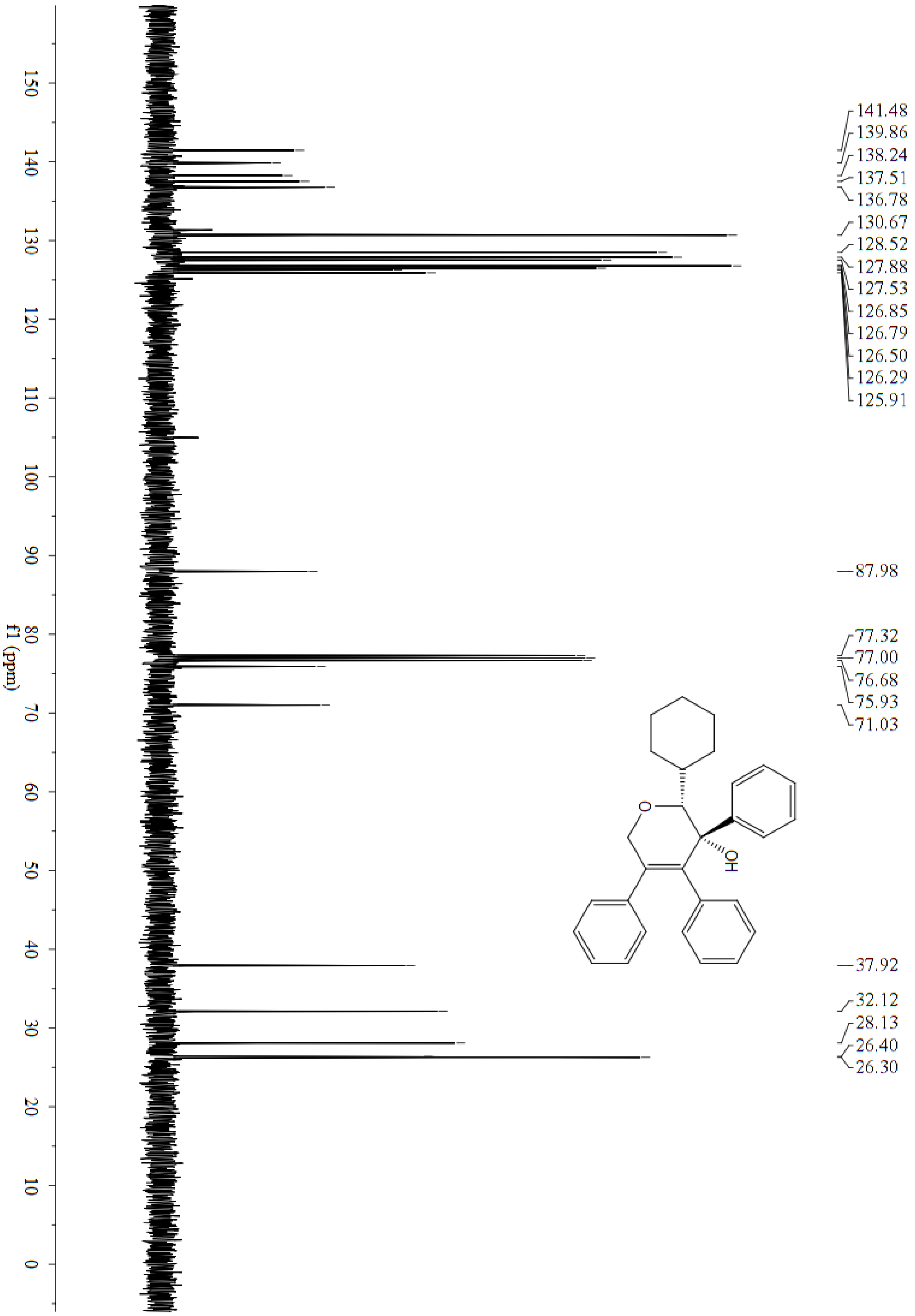
141.48
139.86
138.24
137.51
136.78
130.67
128.52
127.88
127.53
126.85
126.79
126.50
126.29
125.91
87.98
77.32
77.00
76.68
75.93
71.03
37.92
32.12
28.13
26.40
26.30
f1 (ppm)

^{1}H NMR (400MHz,$CDCl_3$)

2-(2-(methylthio)ethyl)-3,4,5-triphenyl-3,6-dihydro-2H-pyran-3-ol (4g)

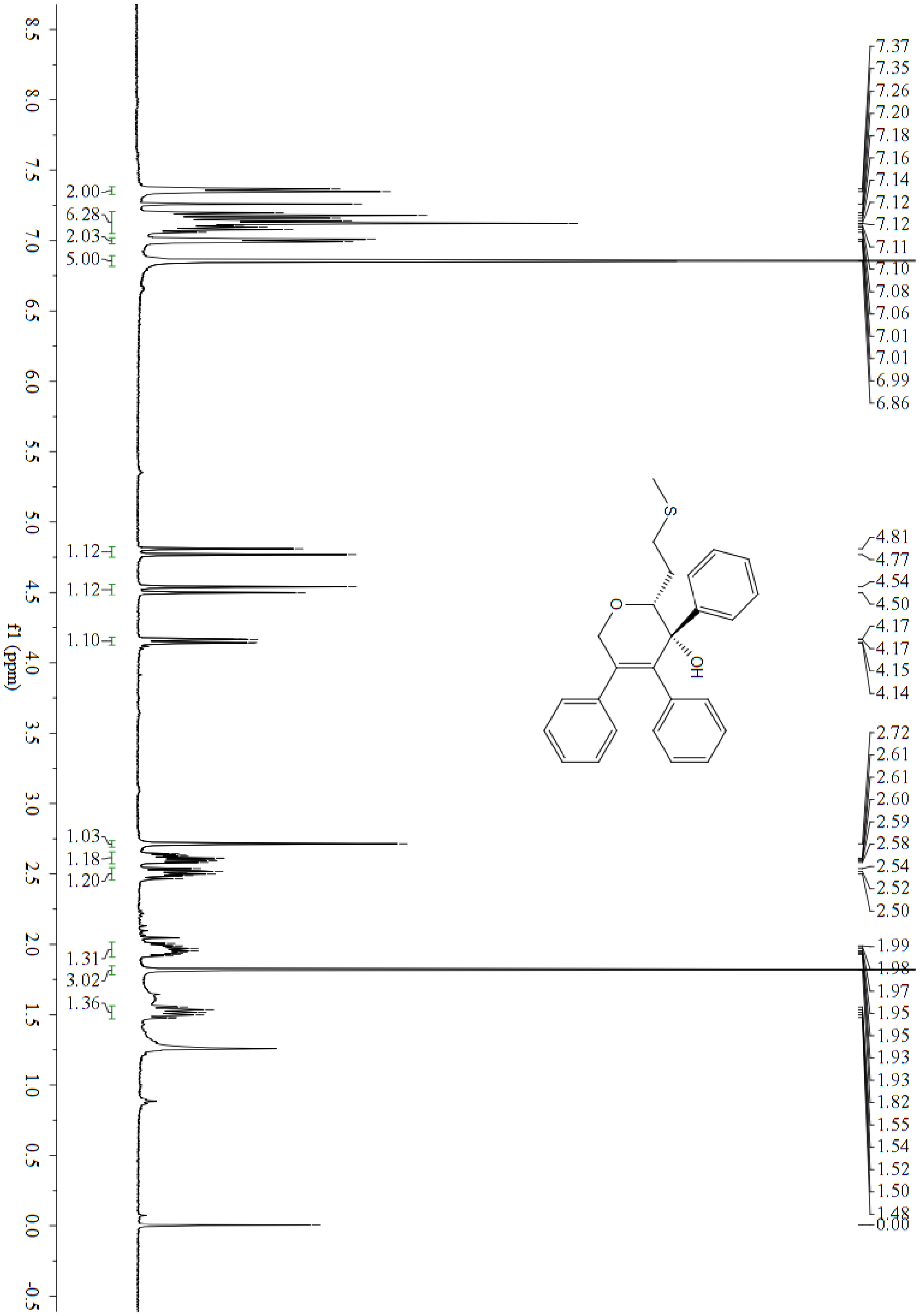

^{13}C NMR (101MHz,$CDCl_3$)

2-(2-(methylthio)ethyl)-3,4,5-triphenyl-3,6-dihydro-2H-pyran-3-ol (4g)

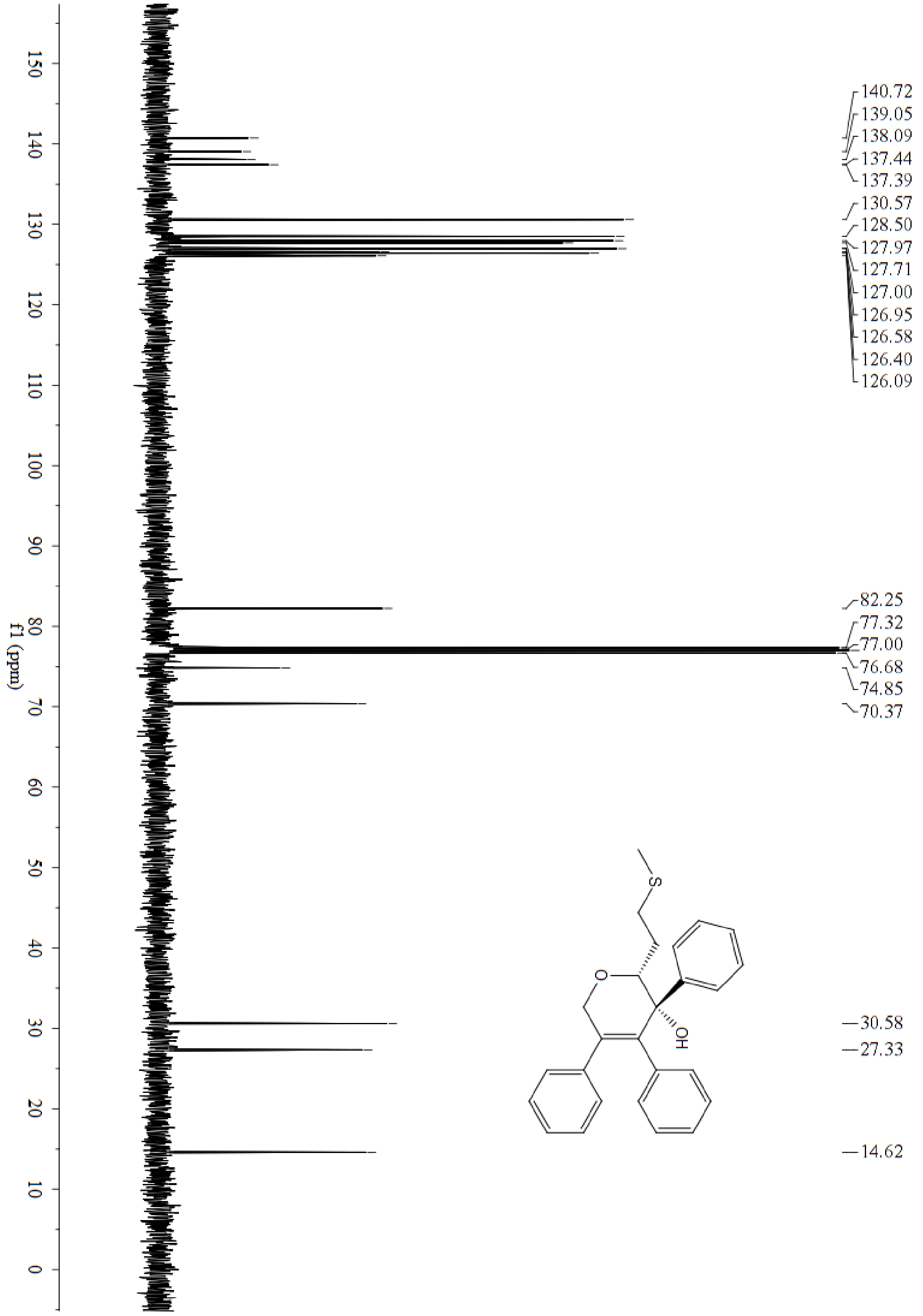

^{1}H NMR (400MHz,$CDCl_3$) 2,3,4,5-tetraphenyl-3,6-dihydro-2H-pyran-3-ol (4h)

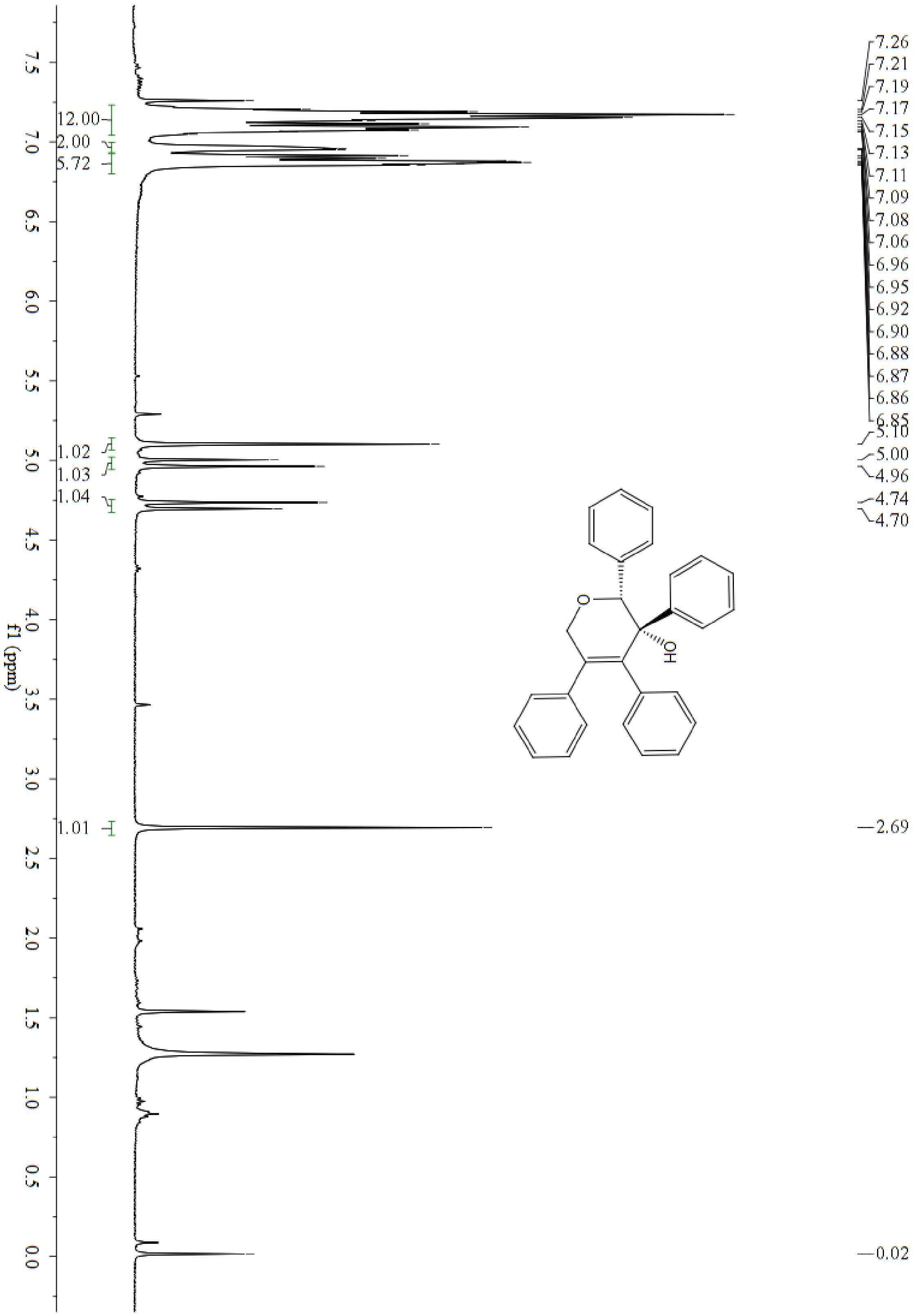

^{13}C NMR (101MHz,$CDCl_3$) 2,3,4,5-tetraphenyl-3,6-dihydro-2H-pyran-3-ol (4h)

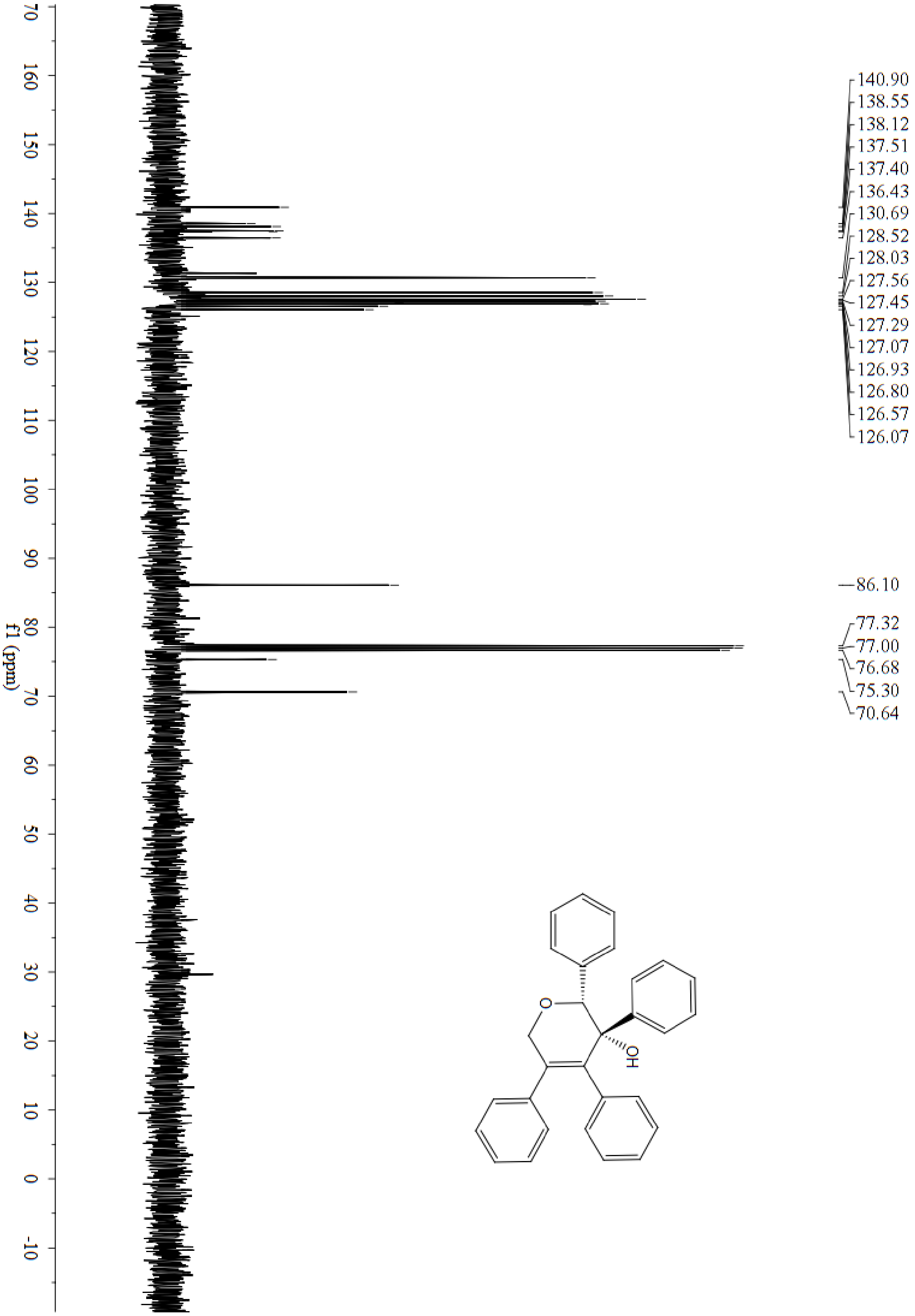

^{1}H NMR (400MHz,$CDCl_3$)

3,4,5-triphenyl-2-(2-(trifluoromethyl)phenyl)-3,6-dihydro-2H-pyran-3-ol (4i)

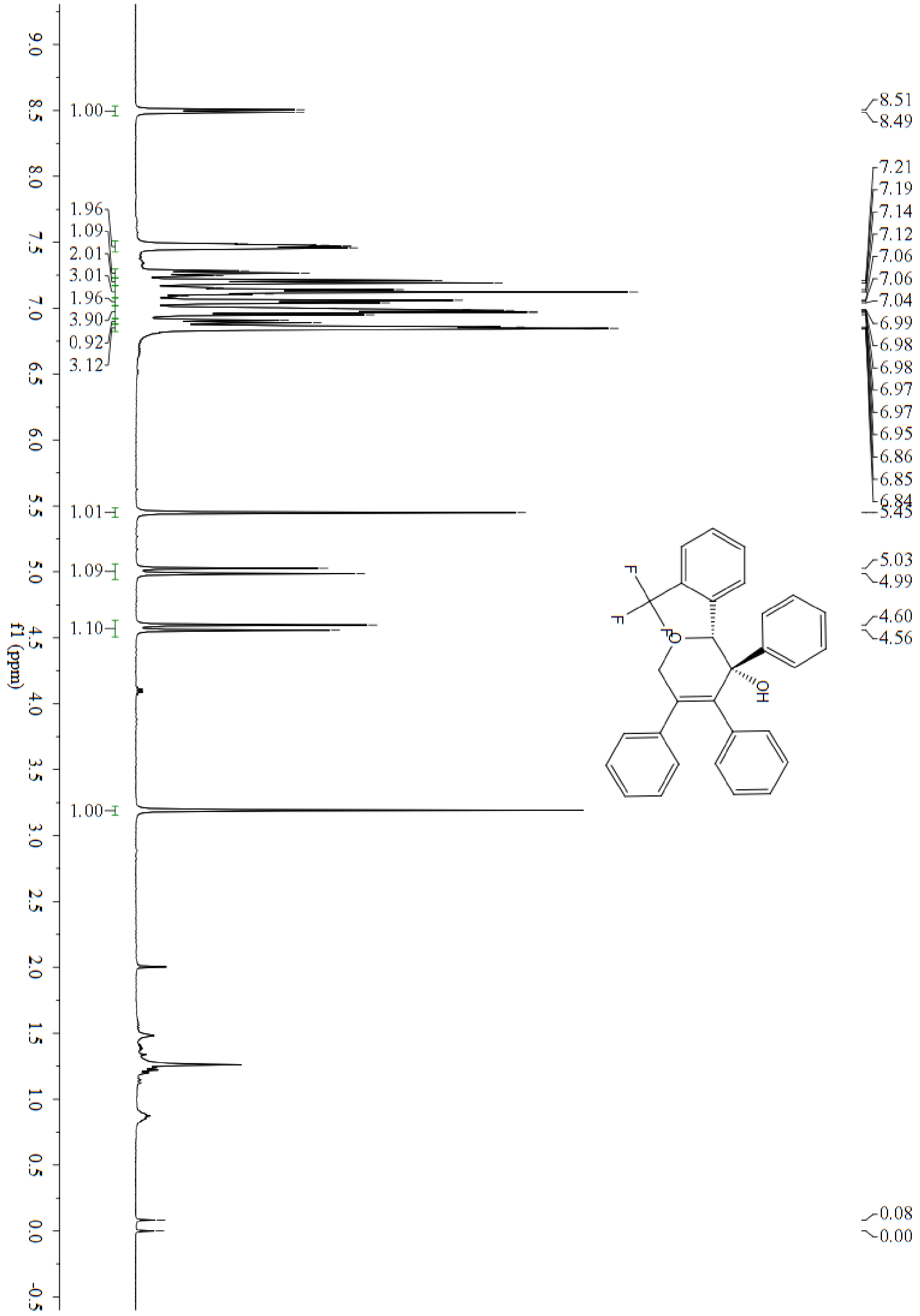

^{13}C NMR (101MHz,$CDCl_3$)

3,4,5-triphenyl-2-(2-(trifluoromethyl)phenyl)-3,6-dihydro-2H-pyran-3-ol (4i)

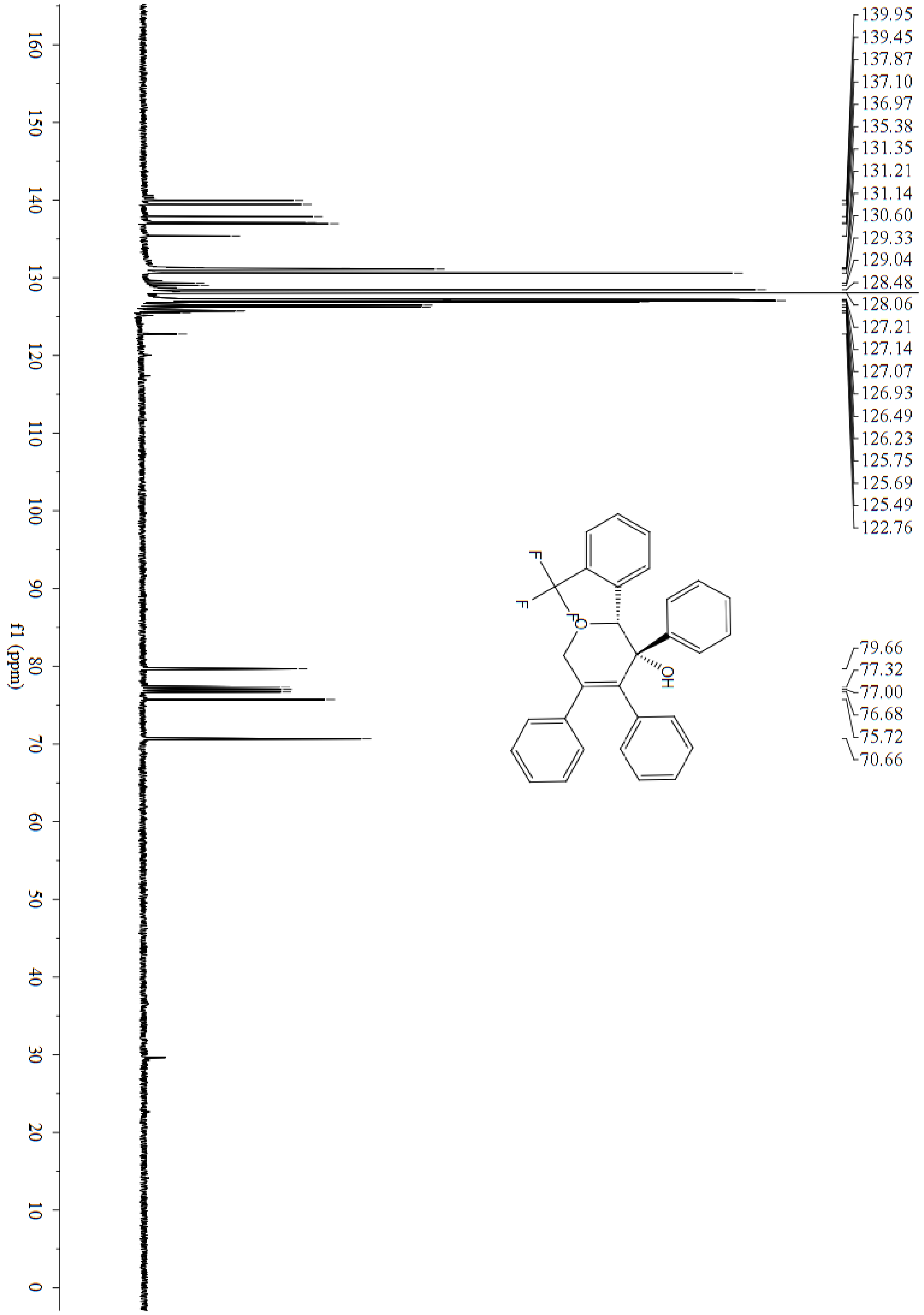

^{1}H NMR (400MHz,CDCl$_3$)

4,5-diethyl-2-phenethyl-3-phenyl-3,6-dihydro-2H-pyran-3-ol (4j)

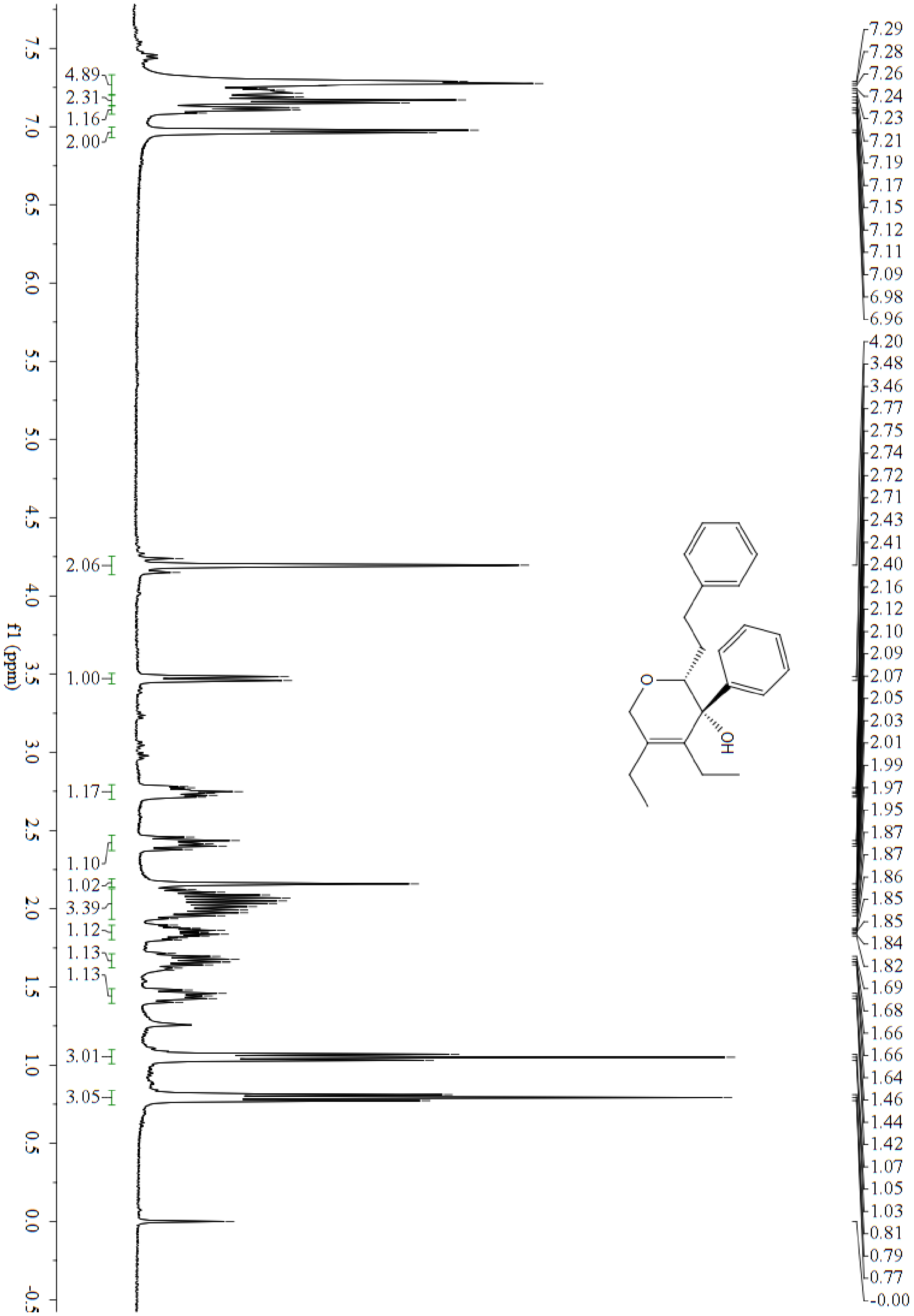

^{13}C NMR (101MHz,$CDCl_3$)

4,5-diethyl-2-phenethyl-3-phenyl-3,6-dihydro-2H-pyran-3-ol (4j)

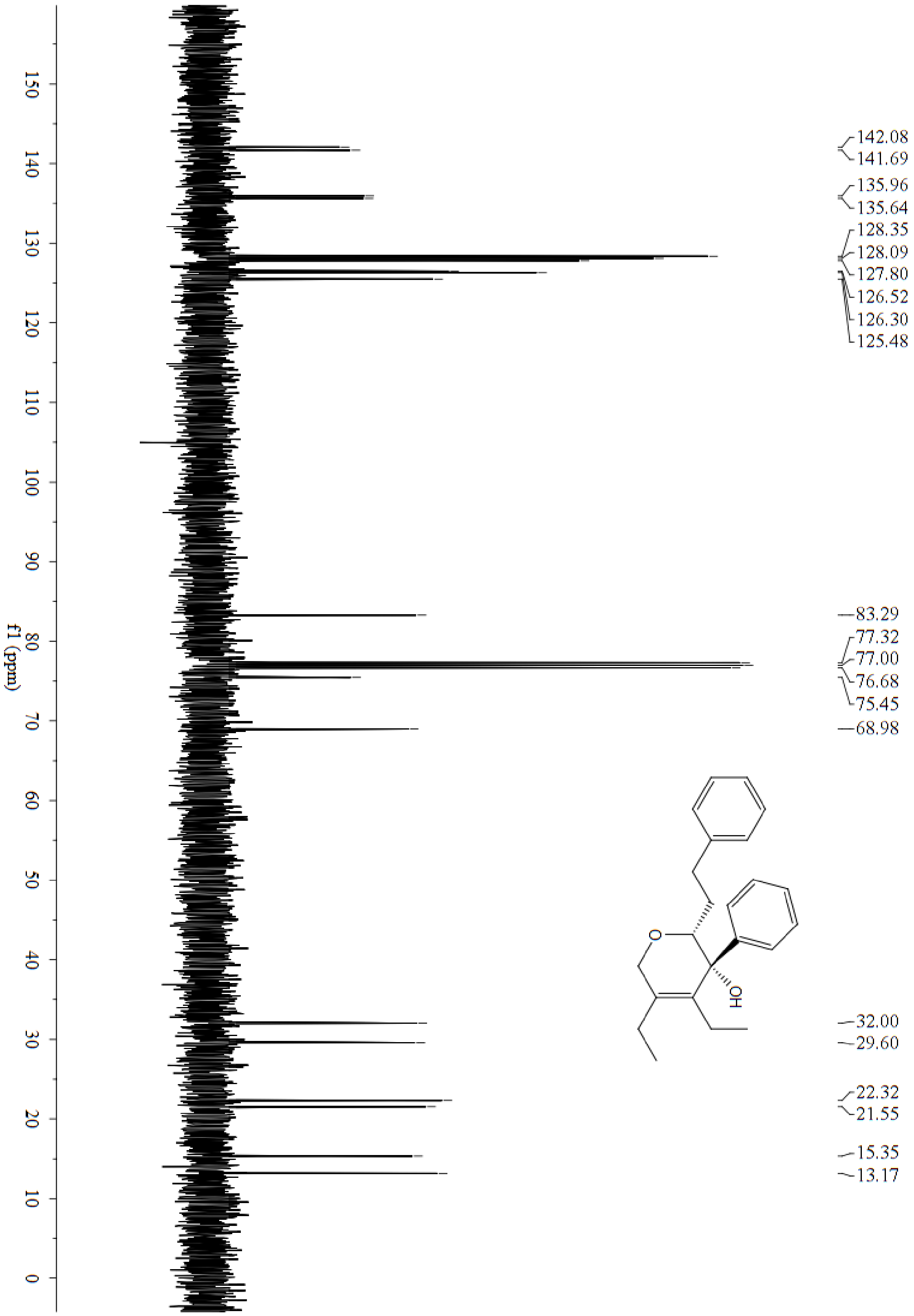

^{1}H NMR (400MHz,CDCl$_3$)

5-methyl-2-phenethyl-3,4-diphenyl-3,6-dihydro-2H-pyran-3-ol (4k)

4-methyl-2-phenethyl-3,5-diphenyl-3,6-dihydro-2H-pyran-3-ol (4k')

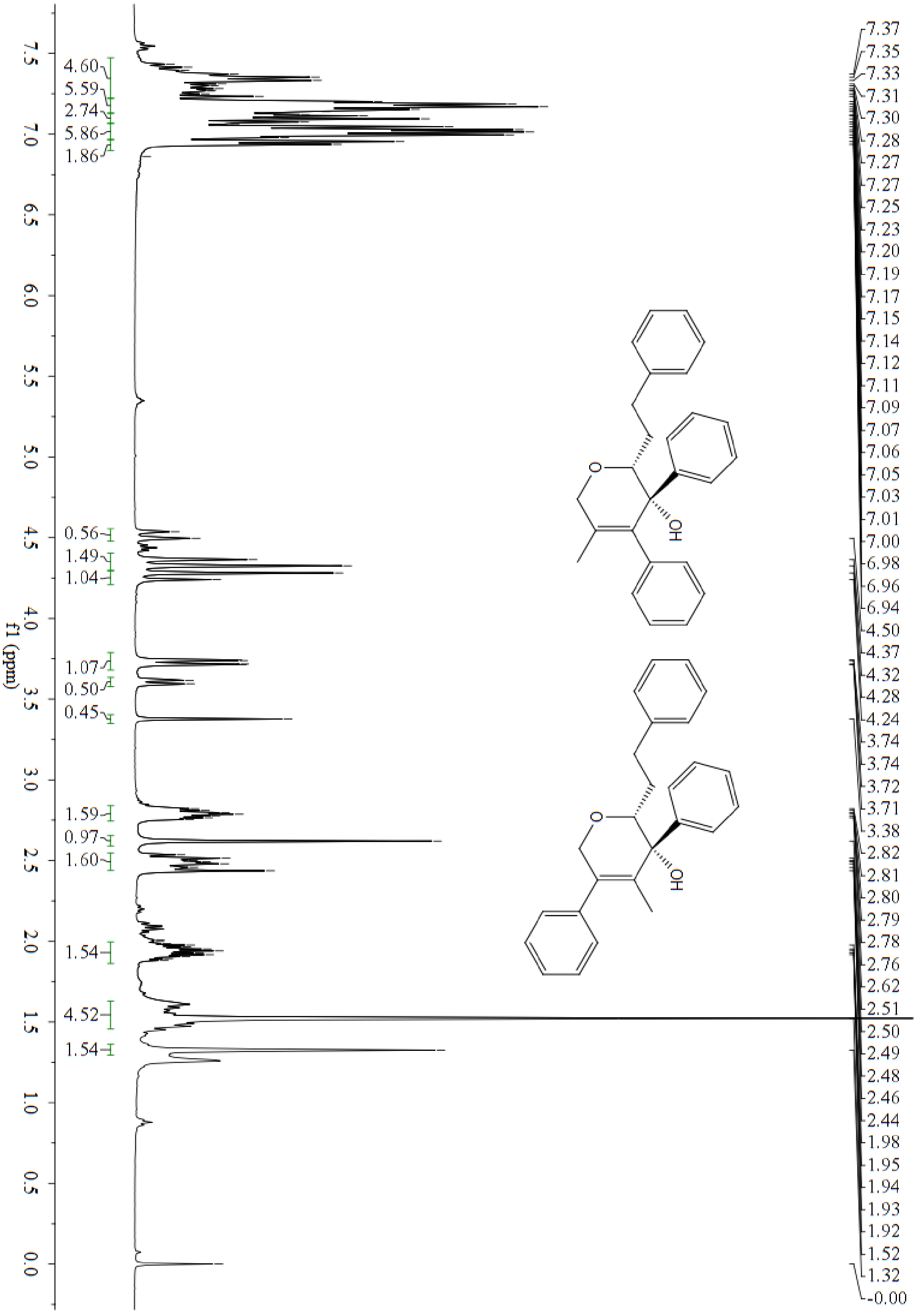

^{13}C NMR (101MHz,$CDCl_3$) (4k)

5-methyl-2-phenethyl-3,4-diphenyl-3,6-dihydro-2H-pyran-3-ol (4k)

4-methyl-2-phenethyl-3,5-diphenyl-3,6-dihydro-2H-pyran-3-ol (4k')

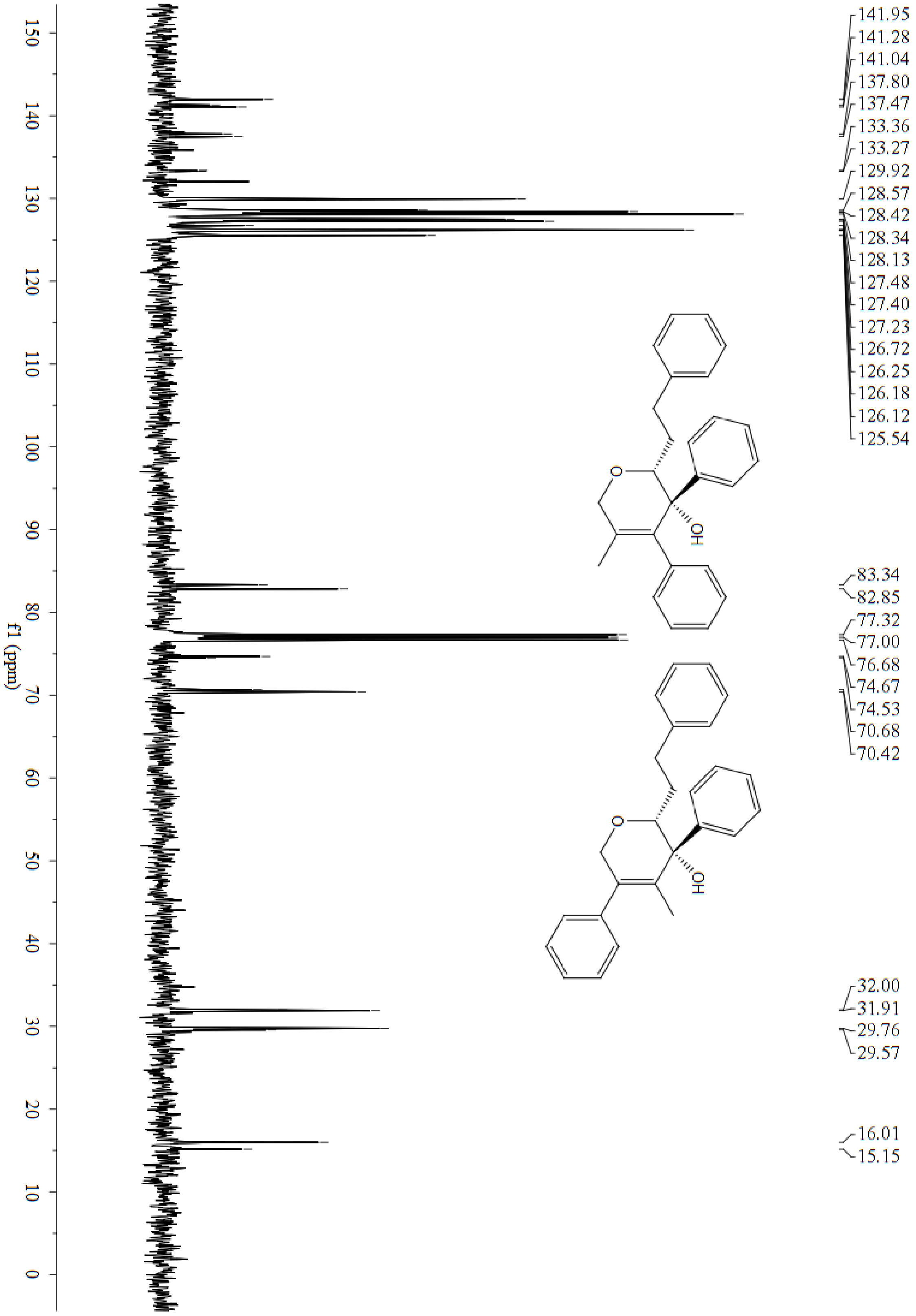

^{1}H NMR (400MHz,$CDCl_3$)

4,5-bis(4-methoxyphenyl)-2-phenethyl-3-phenyl-3,6-dihydro-2H-pyran-3-ol (4l)

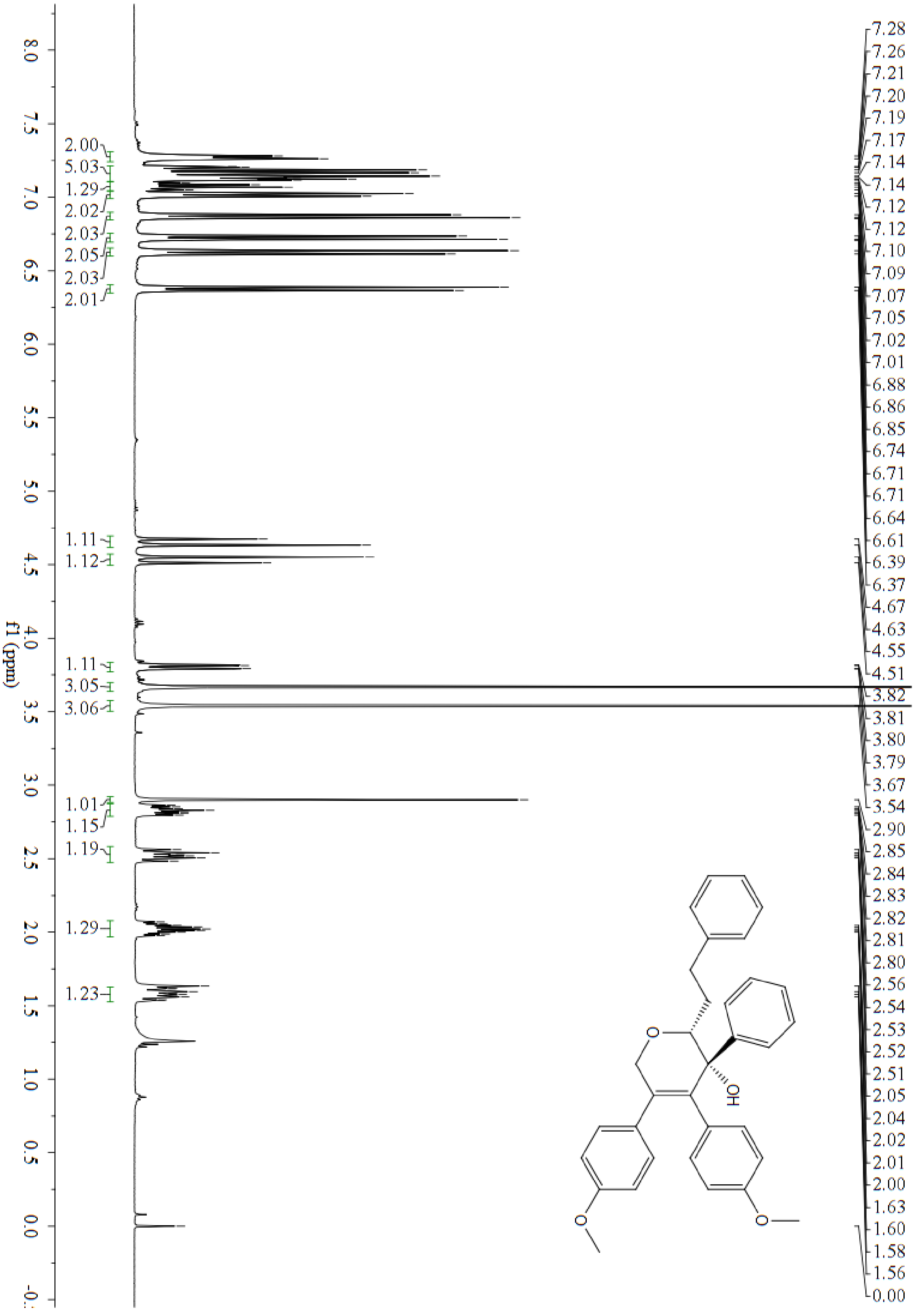

^{13}C NMR (101MHz,$CDCl_3$)

4,5-bis(4-methoxyphenyl)-2-phenethyl-3-phenyl-3,6-dihydro-2H-pyran-3-ol (4l)

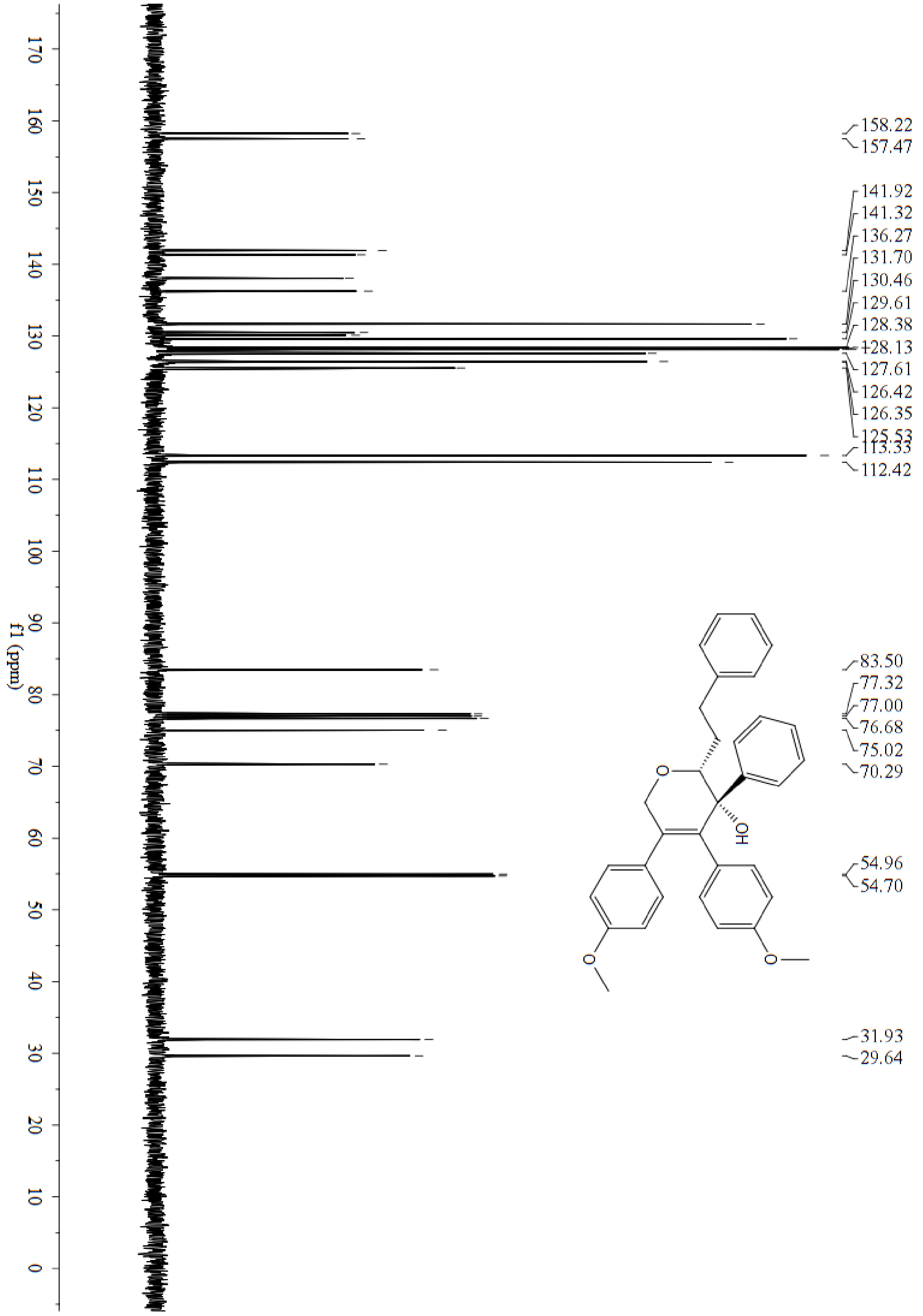

^1H NMR (400MHz,$CDCl_3$)

4,5-bis(3-chlorophenyl)-2-phenethyl-3-phenyl-3,6-dihydro-2H-pyran-3-ol (4m)

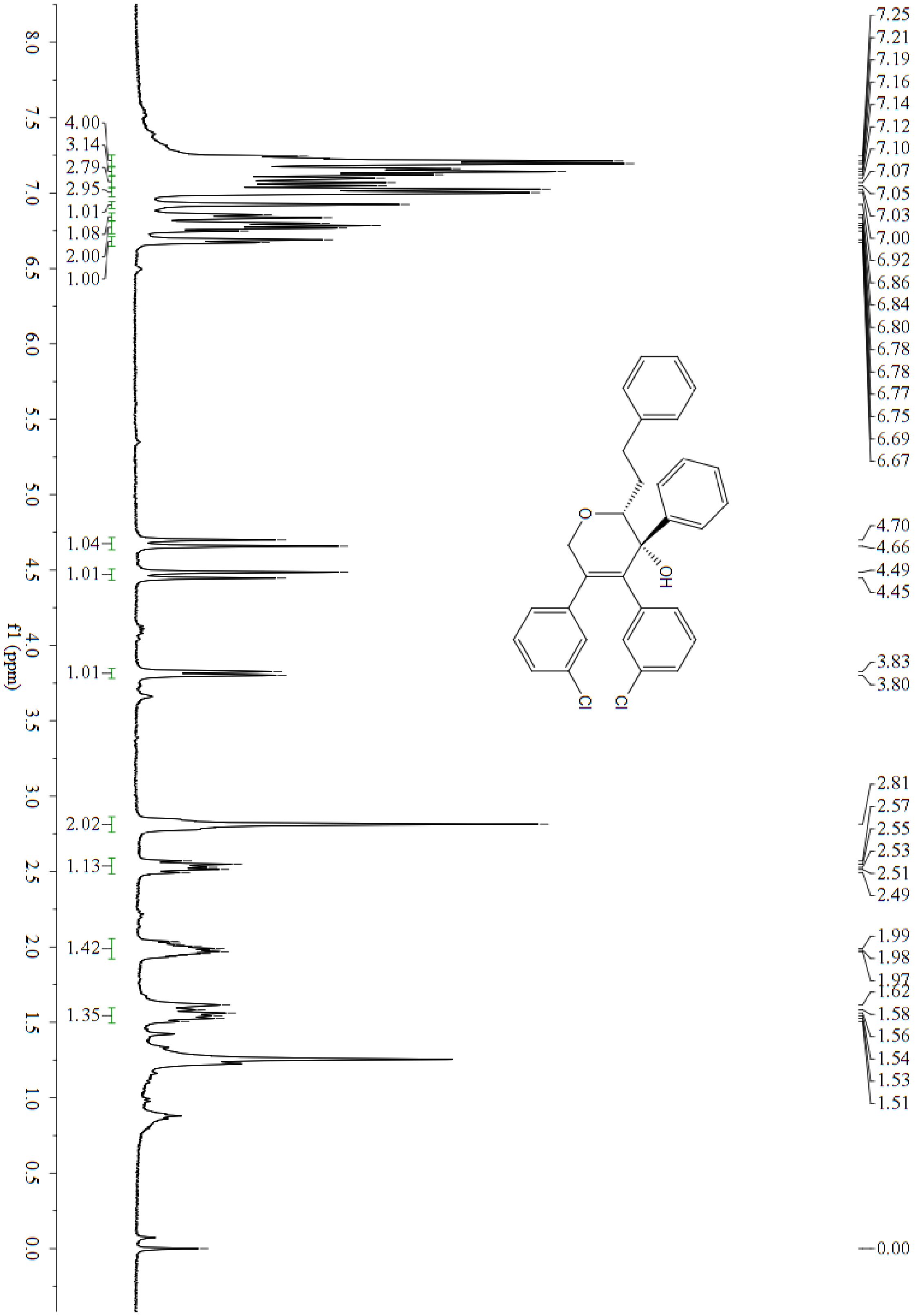

^{13}C NMR (101MHz,$CDCl_3$)

4,5-bis(3-chlorophenyl)-2-phenethyl-3-phenyl-3,6-dihydro-2H-pyran-3-ol (4m)

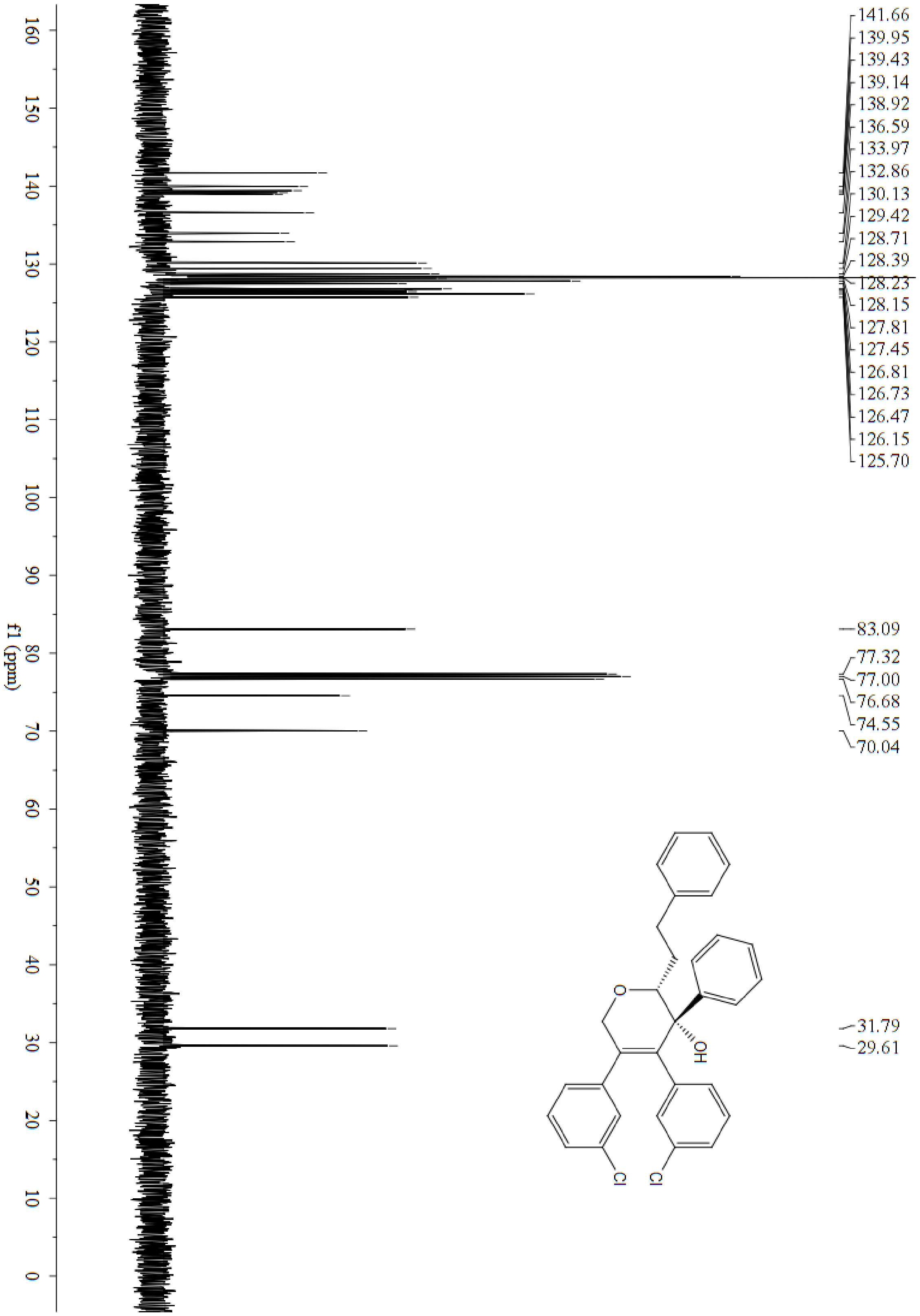

^{1}H NMR (400MHz,CDCl$_3$)

3-(naphthalen-1-yl)-2-phenethyl-4,5-diphenyl-3,6-dihydro-2H-pyran-3-ol (4n)

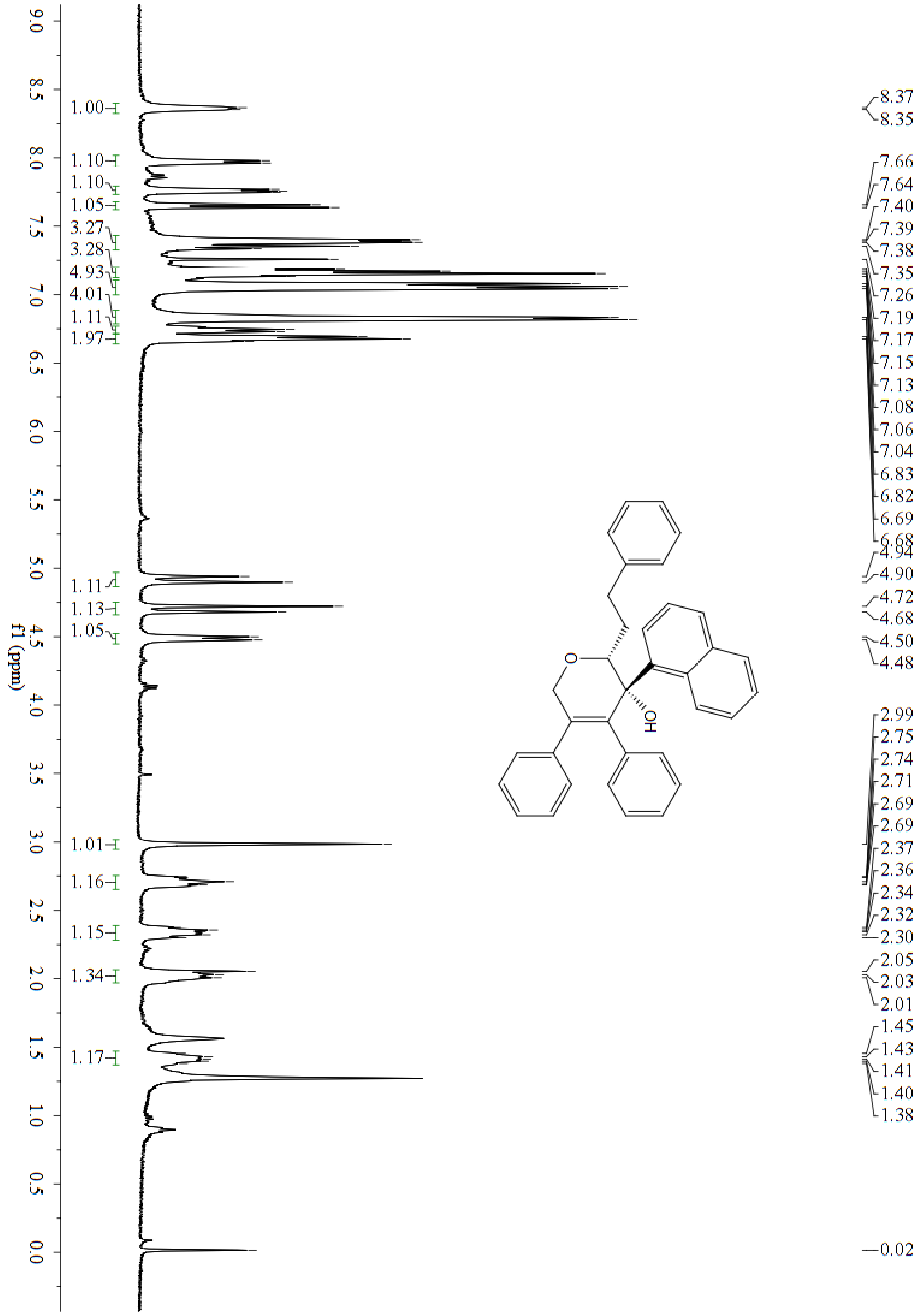

^{13}C NMR (101MHz, $CDCl_3$)

3-(naphthalen-1-yl)-2-phenethyl-4,5-diphenyl-3,6-dihydro-2H-pyran-3-ol (4n)

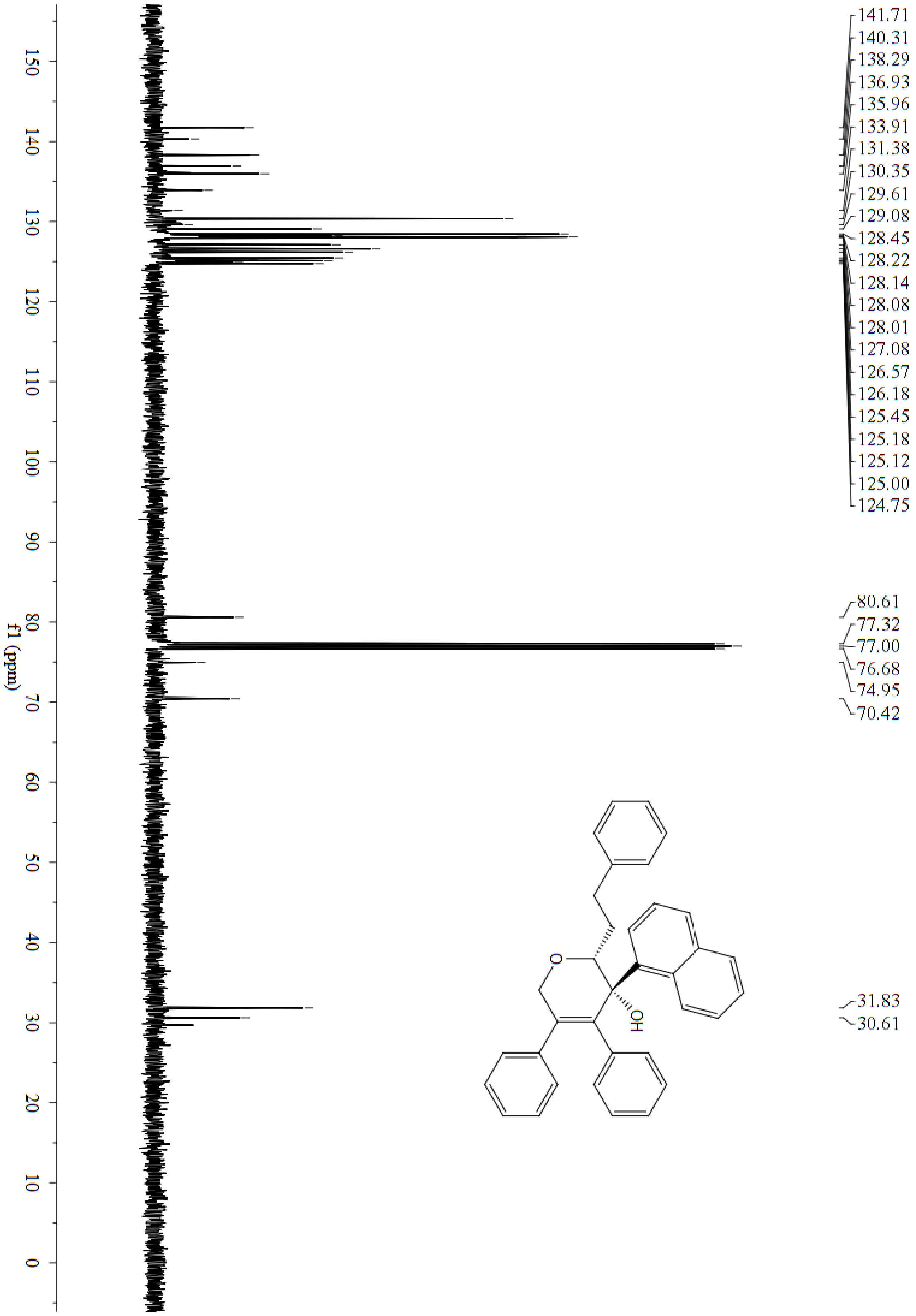

^{1}H NMR (400MHz,$CDCl_3$)
3-(4-methoxyphenyl)-2-phenethyl-4,5-diphenyl-3,6-dihydro-2H-pyran-3-ol (4o)

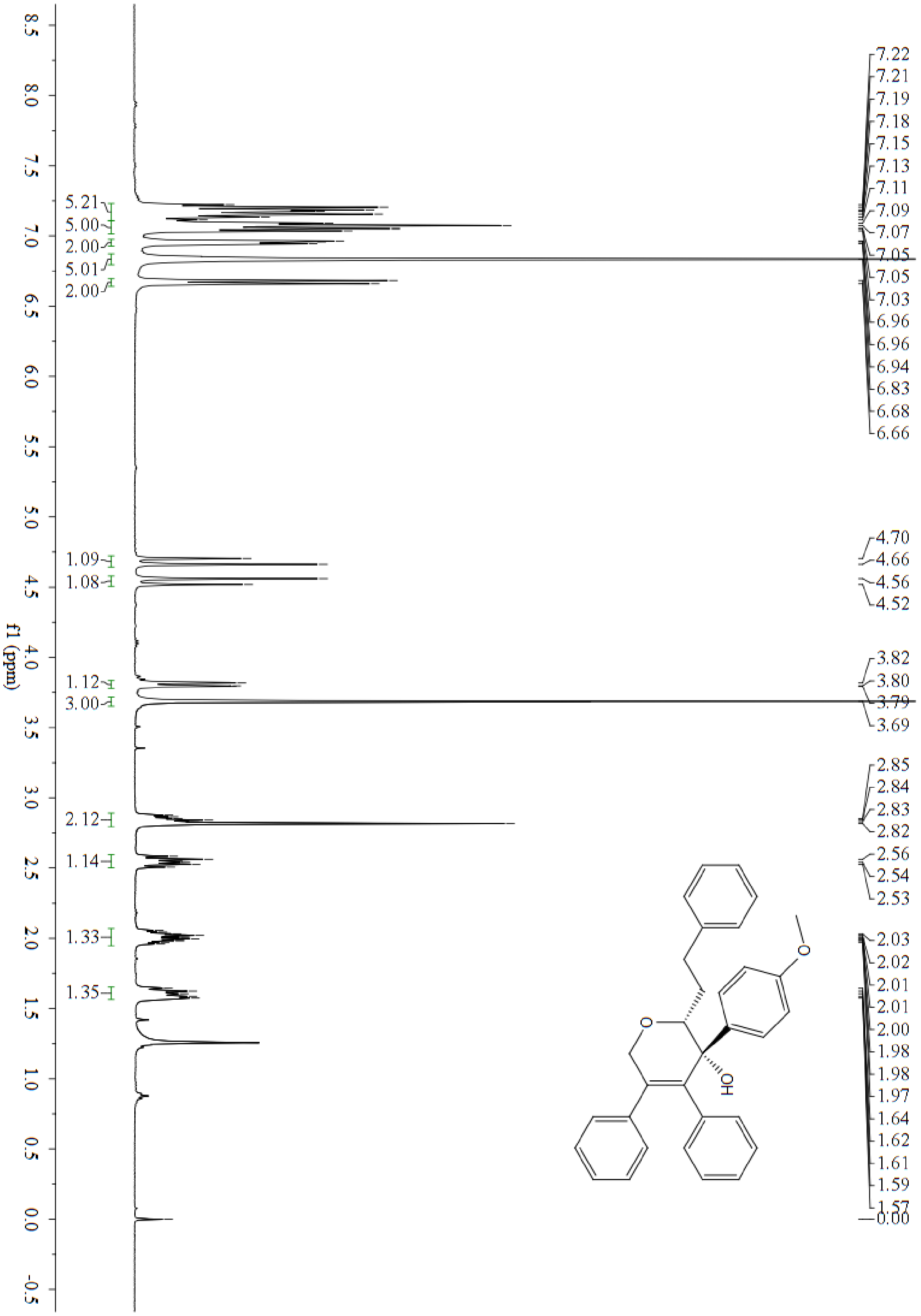

^{13}C NMR (101MHz,$CDCl_3$)

3-(4-methoxyphenyl)-2-phenethyl-4,5-diphenyl-3,6-dihydro-2H-pyran-3-ol (4o)

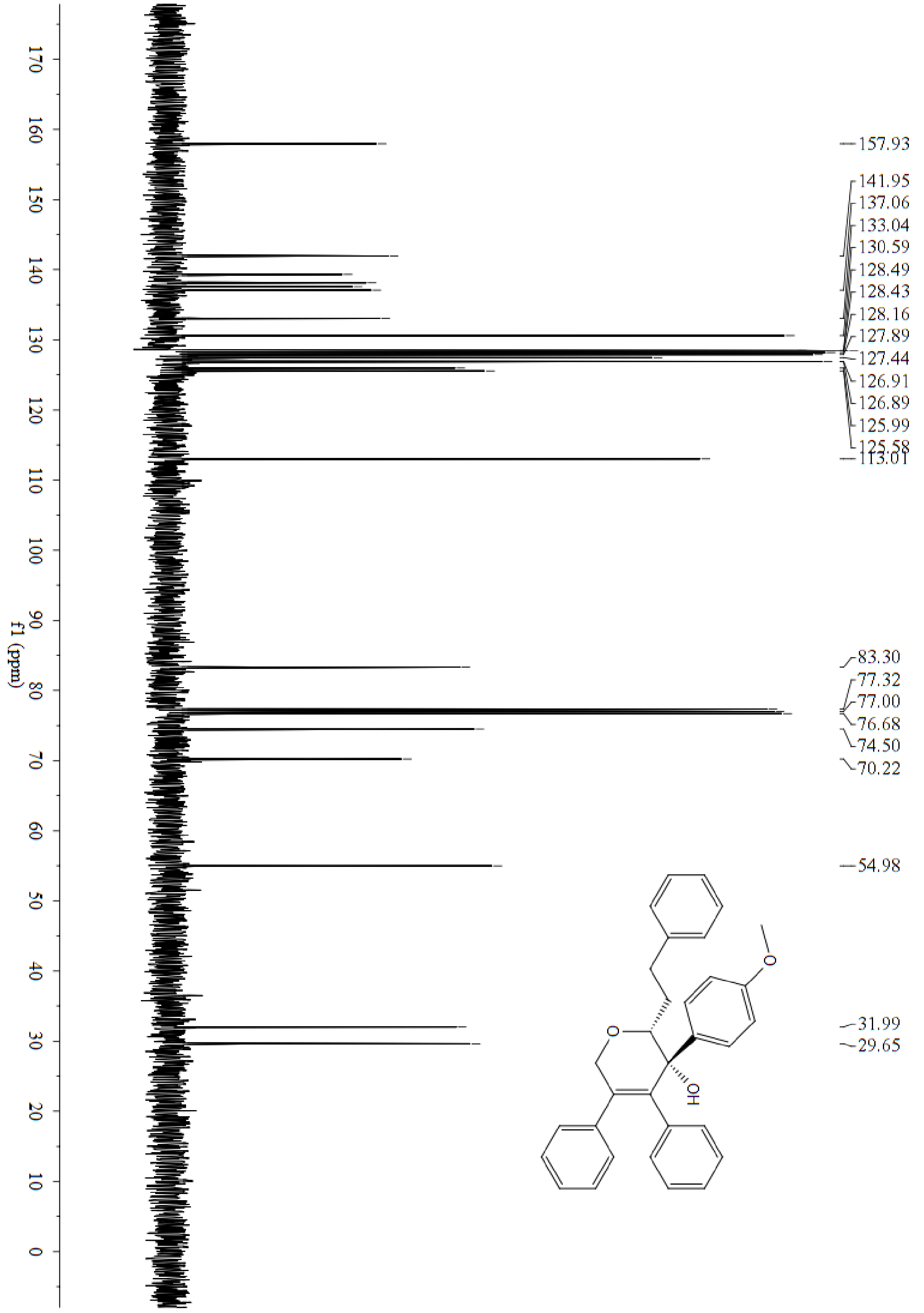

^{1}H NMR (400MHz,DMSO-D6)

2-phenethyl-4,5-diphenyl-3-(o-tolyl)-3,6-dihydro-2H-pyran-3-ol (4p)

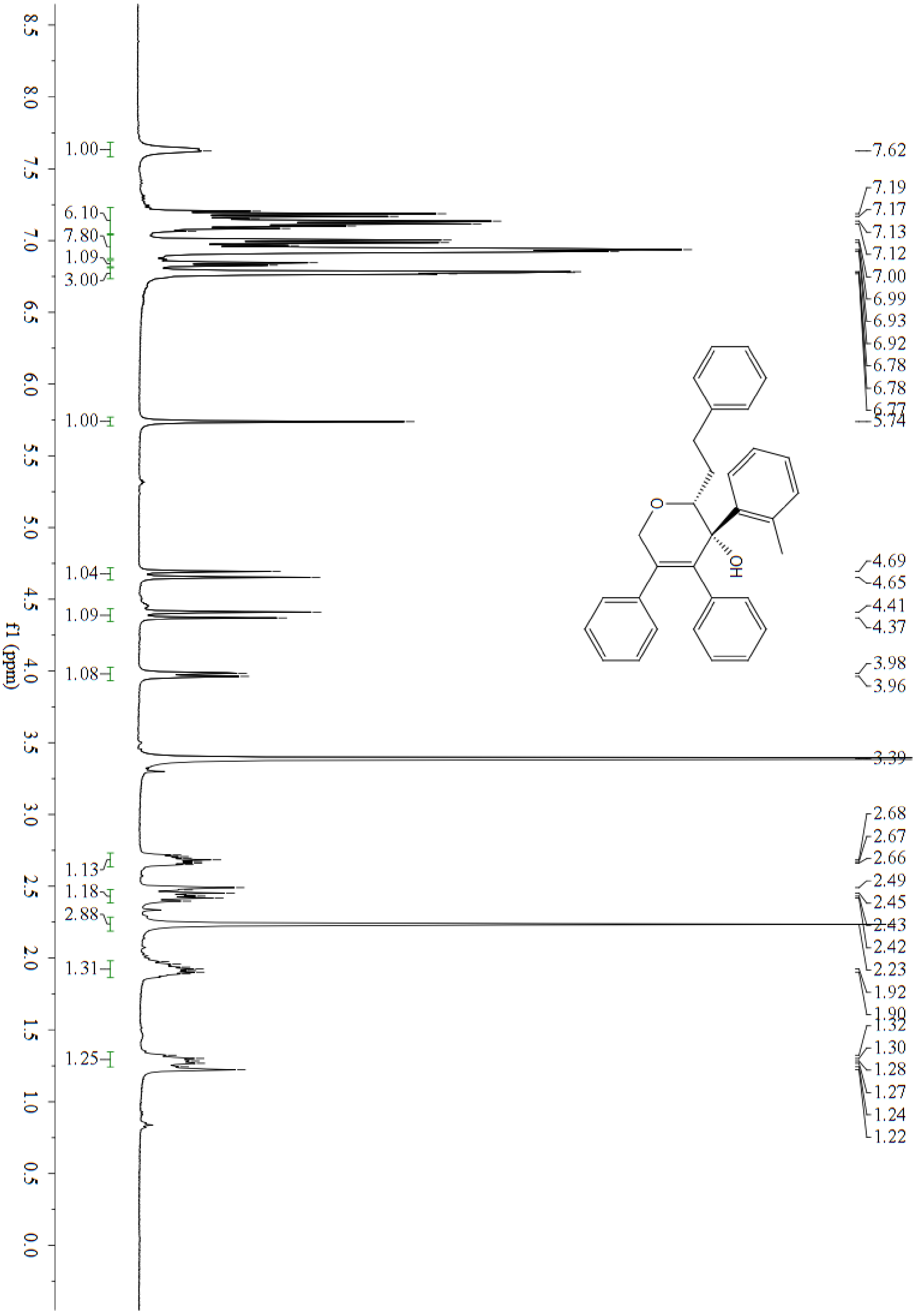

^{13}C NMR (101MHz,DMSO-D6)

2-phenethyl-4,5-diphenyl-3-(o-tolyl)-3,6-dihydro-2H-pyran-3-ol (4p)

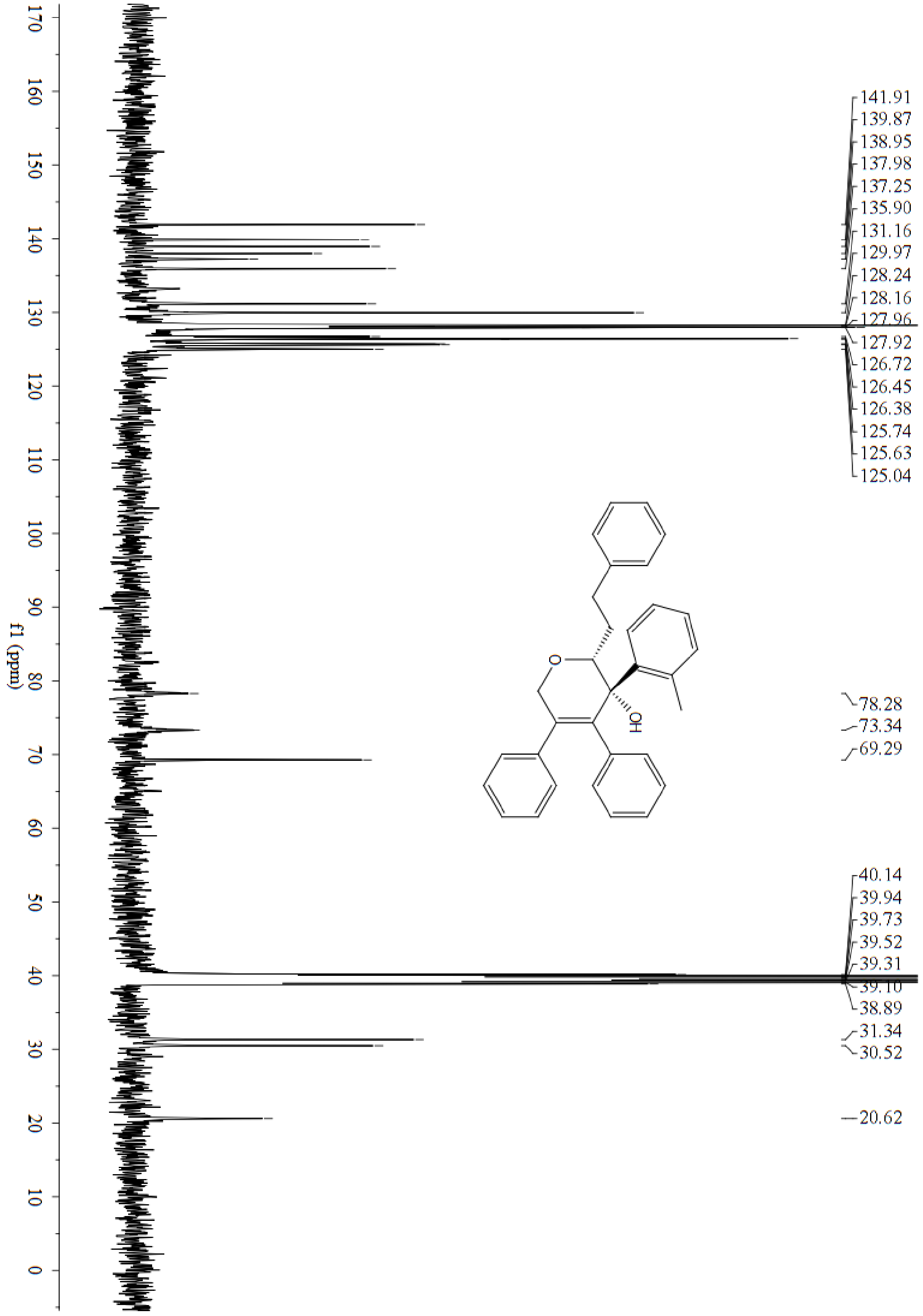

^{1}H NMR (400MHz,CDCl$_3$)

3-(4-fluorophenyl)-2-phenethyl-4,5-diphenyl-3,6-dihydro-2H-pyran-3-ol (4q)

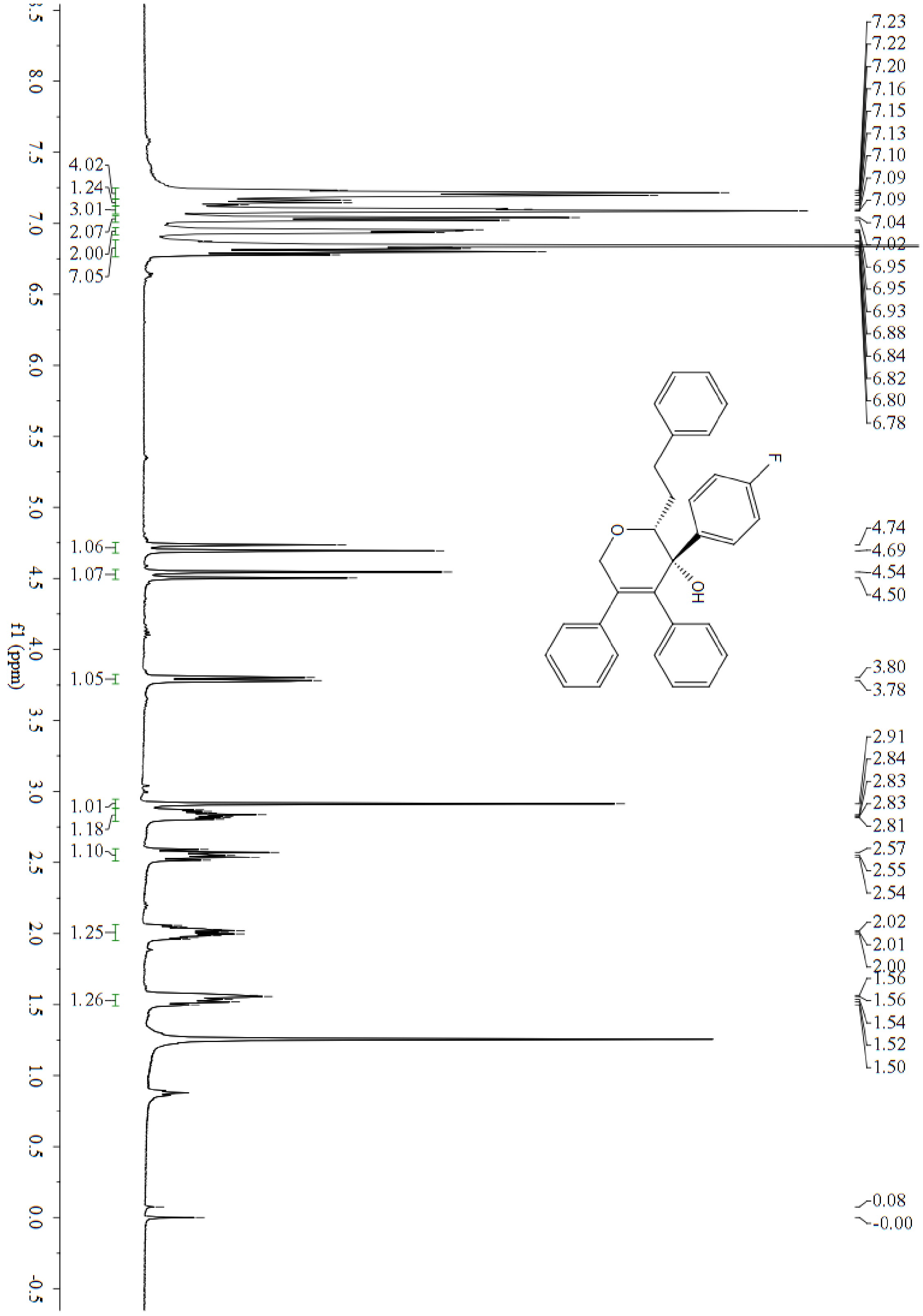

^{13}C NMR (101MHz,$CDCl_3$)

3-(4-fluorophenyl)-2-phenethyl-4,5-diphenyl-3,6-dihydro-2H-pyran-3-ol (4q)

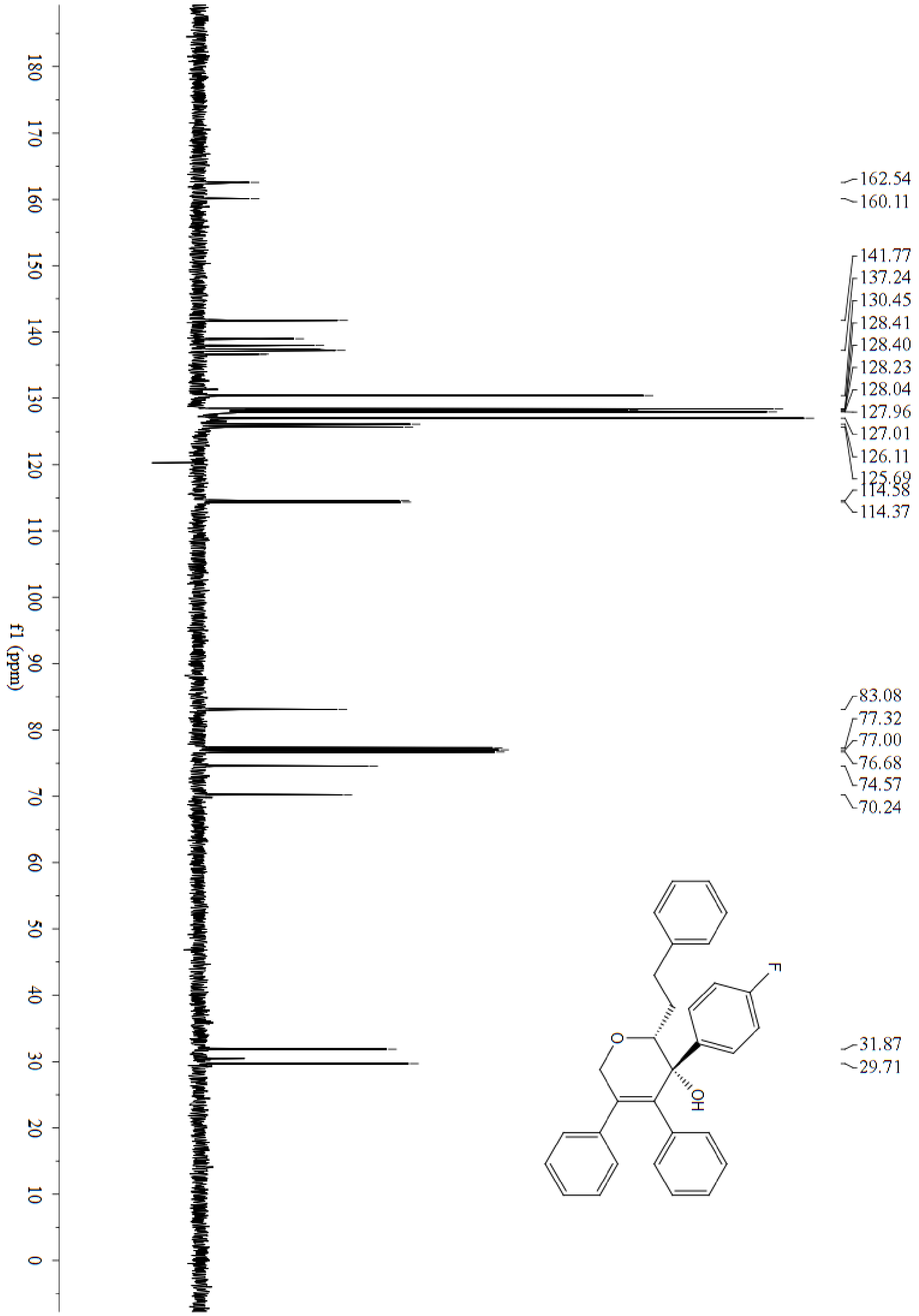

^{1}H NMR (400MHz,$CDCl_3$) 2-pentyl-3,4,5-triphenyl-3,6-dihydro-2H-pyran-3-ol (4r)

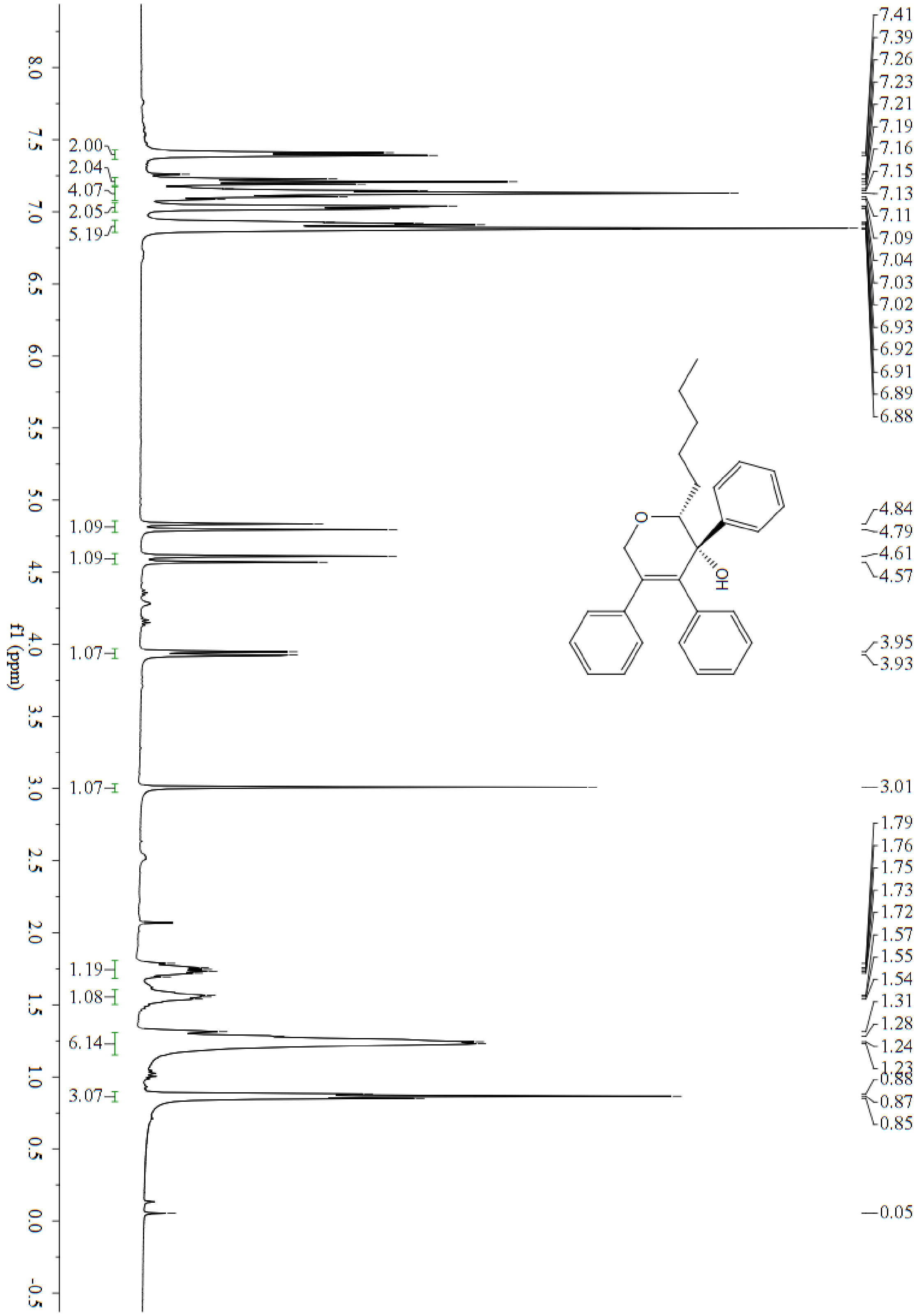

^{13}C NMR (101MHz, $CDCl_3$) 2-pentyl-3,4,5-triphenyl-3,6-dihydro-2H-pyran-3-ol (4r)

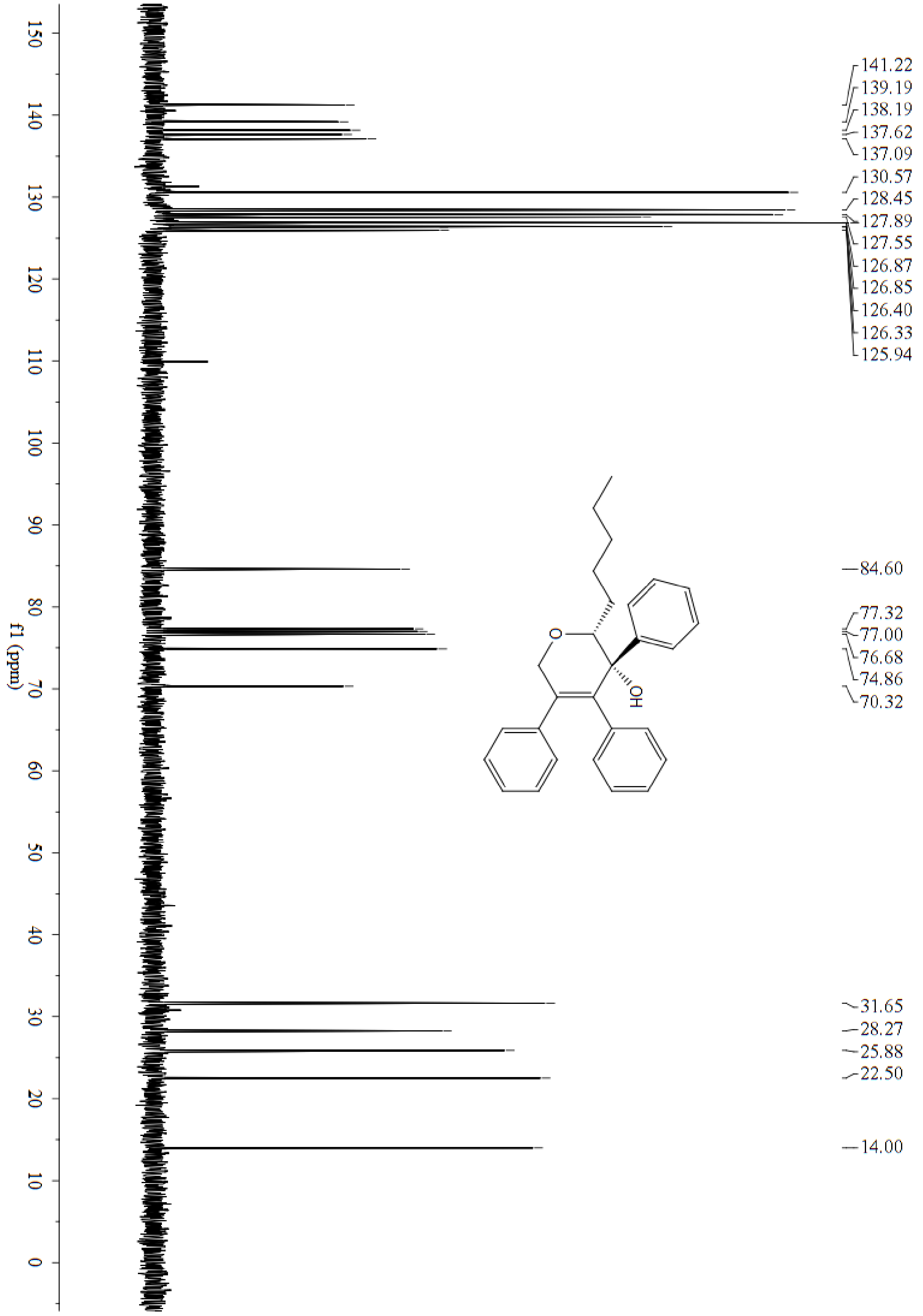

HPLC (2R,3R)-2-phenethyl-3-phenyloxetan-3-ol (3a ,Racemic)

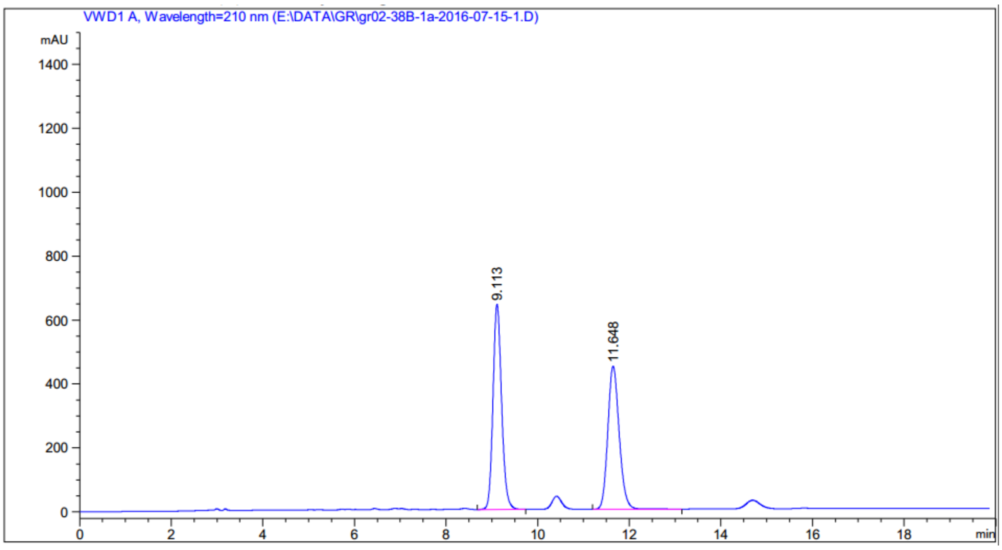

Signal 1: VWD1 A, Wavelength=210 nm

Peak #	RetTime [min]	Type	Width [min]	Area [mAU*s]	Height [mAU]	Area %
1	9.113	BB	0.2002	8377.17285	642.11981	51.9350
2	11.648	BV R	0.2680	7752.95068	447.87415	48.0650

Totals : 1.61301e4 1089.99396

HPLC (2R,3R)-2-phenethyl-3-phenyloxetan-3-ol (3a-*ent* ,99%ee)

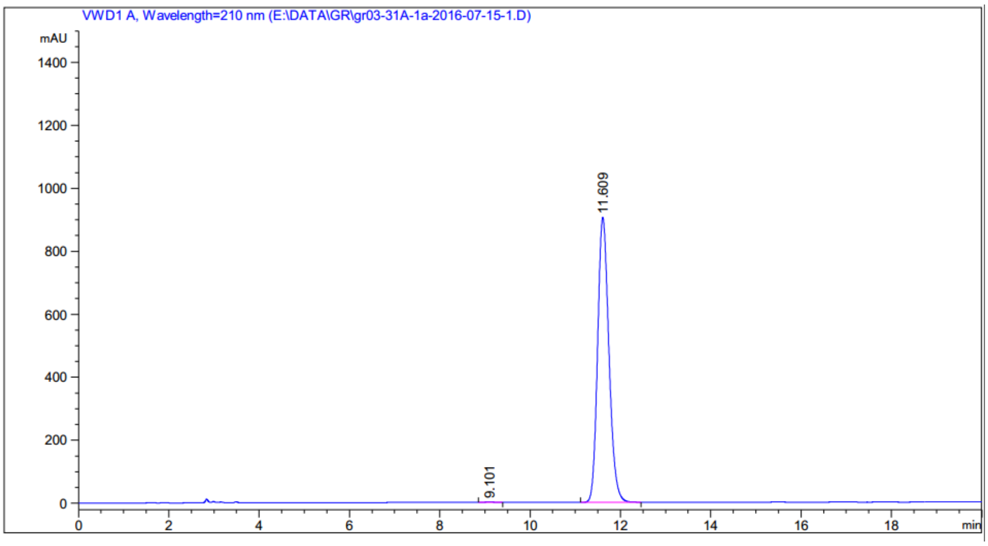

Peak #	RetTime [min]	Type	Width [min]	Area [mAU*s]	Height [mAU]	Area %
1	9.101	BB	0.1912	24.78811	2.00535	0.1579
2	11.609	BB	0.2670	1.56693e4	904.90594	99.8421

Totals : 1.56941e4 906.91129

HPLC (2R,3R)-2-phenethyl-3,4,5-triphenyl-3,6-dihydro-2H-pyran-3-ol (4a, Racemic)

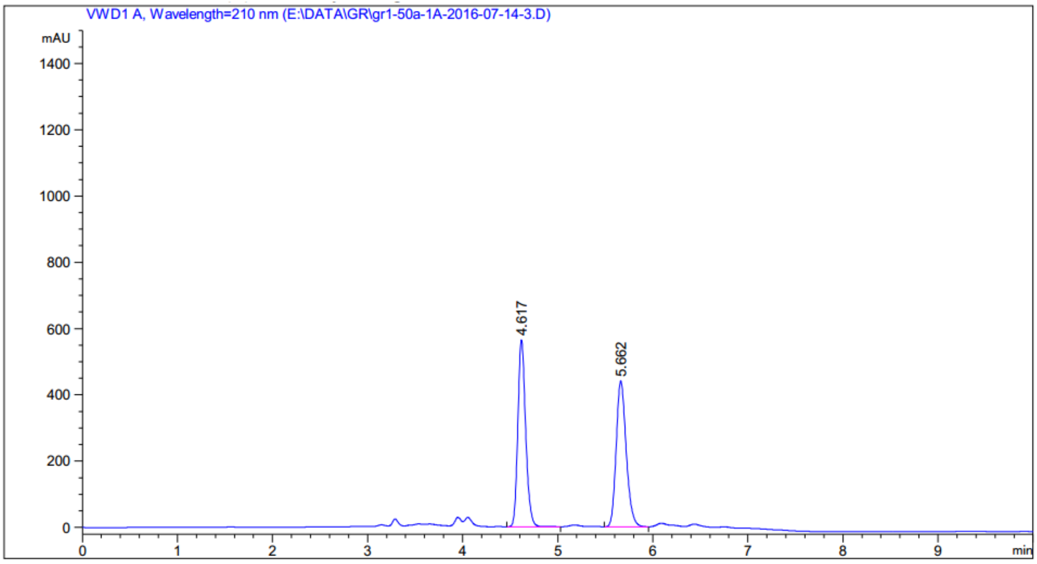

Peak #	RetTime [min]	Type	Width [min]	Area [mAU*s]	Height [mAU]	Area %
1	4.617	BV R	0.0876	3220.52246	564.53503	50.0231
2	5.662	BB	0.1123	3217.55249	439.87198	49.9769

Totals : 6438.07495 1004.40701

HPLC (2R,3R)-2-phenethyl-3,4,5-triphenyl-3,6-dihydro-2H-pyran-3-ol (4a-*ent*, 99%ee)

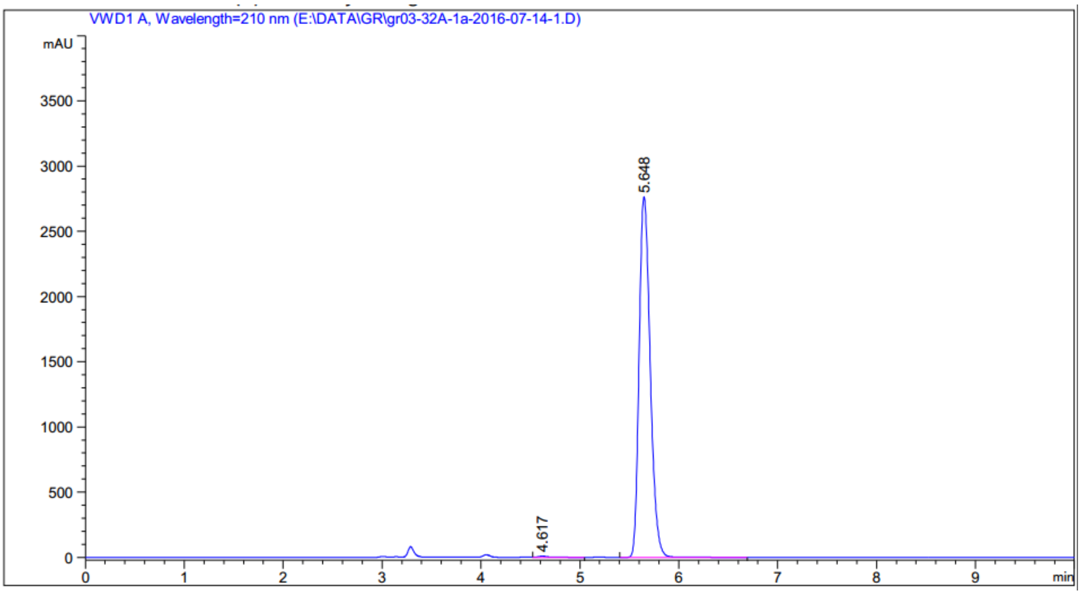

Peak #	RetTime [min]	Type	Width [min]	Area [mAU*s]	Height [mAU]	Area %
1	4.617	VV	0.1269	82.30604	8.98534	0.3809
2	5.648	BV R	0.1216	2.15271e4	2762.73071	99.6191

Totals : 2.16094e4 2771.71606

HPLC (2R,3R)-2,3-diphenyloxetan-3-ol (3h, Racemic)

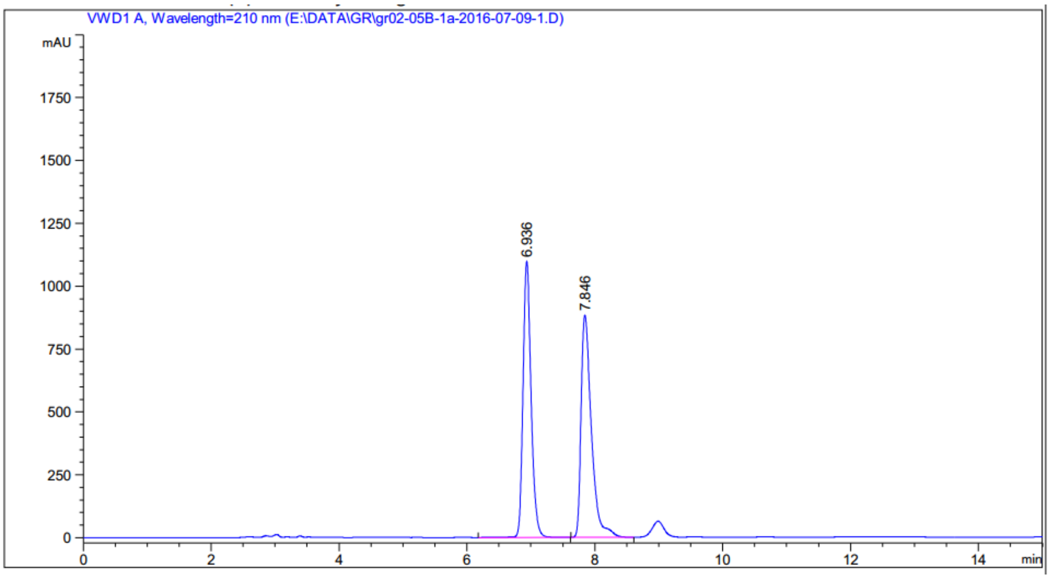

Peak #	RetTime [min]	Type	Width [min]	Area [mAU*s]	Height [mAU]	Area %
1	6.936	VV R	0.1338	9593.78906	1097.91553	49.2276
2	7.846	BB	0.1687	9894.84863	883.34039	50.7724
Totals :				1.94886e4	1981.25592	

HPLC (2R,3R)-2,3-diphenyloxetan-3-ol (3h-*ent*, 96% ee)

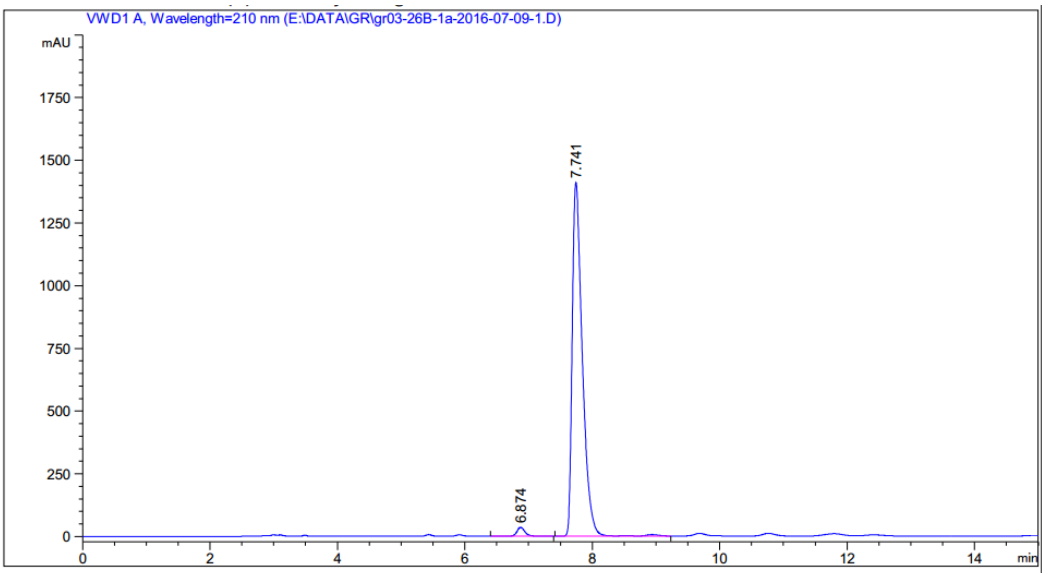

Peak #	RetTime [min]	Type	Width [min]	Area [mAU*s]	Height [mAU]	Area %
1	6.874	VB R	0.1332	311.67560	35.21918	1.9687
2	7.741	BV R	0.1658	1.55195e4	1410.04370	98.0313
Totals :				1.58312e4	1445.26289	

HPLC (2R,3R)-2,3,4,5-tetraphenyl-3,6-dihydro-2H-pyran-3-ol (4h, Racemic)

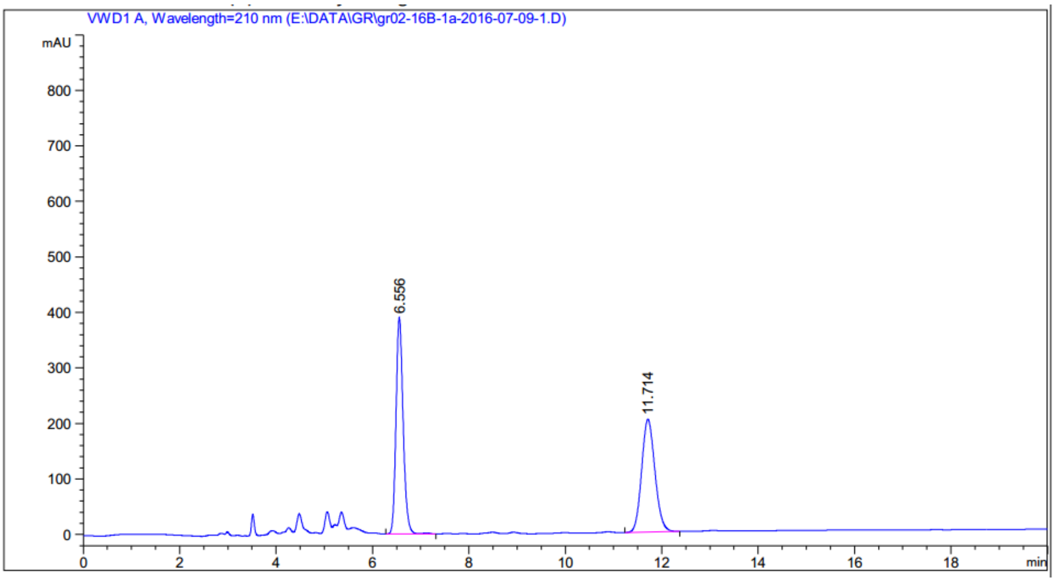

Peak #	RetTime [min]	Type	Width [min]	Area [mAU*s]	Height [mAU]	Area %
1	6.556	BV R	0.1571	4011.37500	390.56256	50.7586
2	11.714	BB	0.2976	3891.47217	202.99805	49.2414
Totals :				7902.84717	593.56061	

HPLC (2R,3R)-2,3,4,5-tetraphenyl-3,6-dihydro-2H-pyran-3-ol (4h-*ent,* 95%ee)

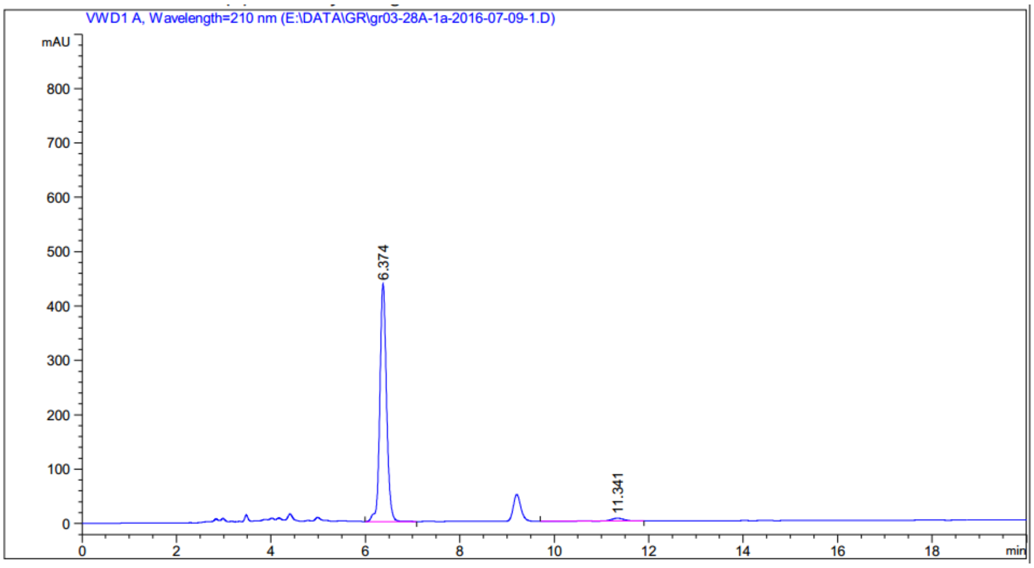

Peak #	RetTime [min]	Type	Width [min]	Area [mAU*s]	Height [mAU]	Area %
1	6.374	BV R	0.1497	4299.40771	437.37769	97.3918
2	11.341	VB R	0.3322	115.13932	5.16336	2.6082
Totals :				4414.54704	442.54104	

HPLC (2S,3S)-2-pentyl-3-phenyloxetan-3-ol (3r, Racemic)

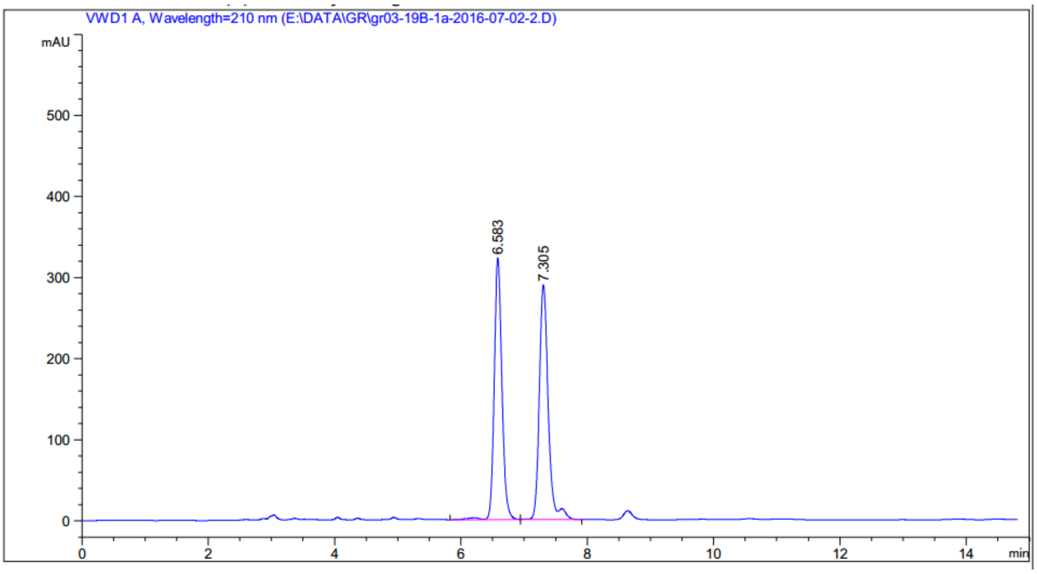

Peak #	RetTime [min]	Type	Width [min]	Area [mAU*s]	Height [mAU]	Area %
1	6.583	VB R	0.1284	2720.40039	322.17178	49.0750
2	7.305	BV R	0.1439	2822.94727	288.97003	50.9250
Totals :				5543.34766	611.14182	

HPLC (2S,3S)-2-pentyl-3-phenyloxetan-3-ol (3r-*ent,* 99%ee)

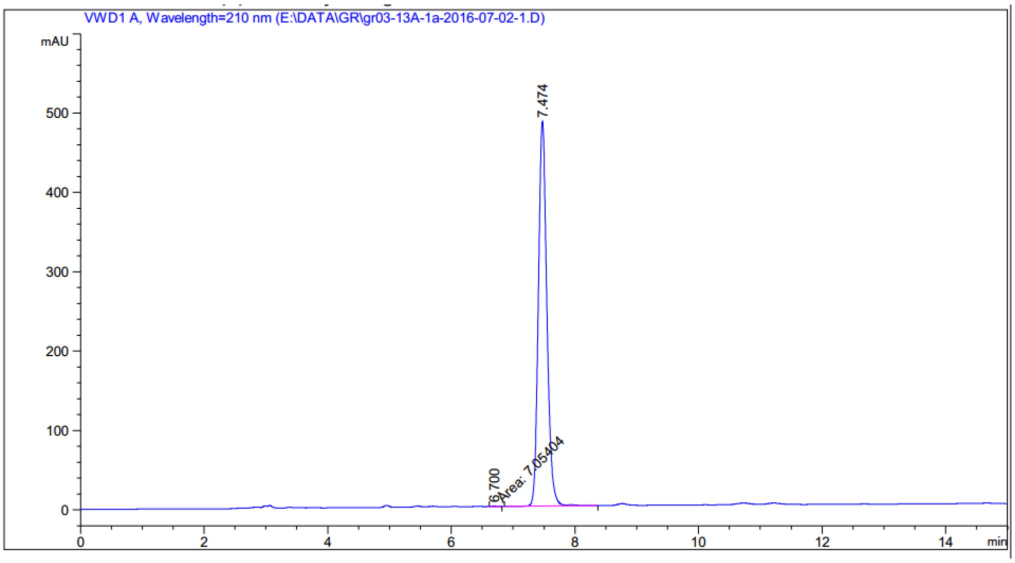

Peak #	RetTime [min]	Type	Width [min]	Area [mAU*s]	Height [mAU]	Area %
1	6.700	MM	0.1536	7.05404	7.65641e-1	0.1520
2	7.474	BV R	0.1466	4634.66357	484.59393	99.8480
Totals :				4641.71761	485.35957	

(2S,3S)-2-pentyl-3,4,5-triphenyl-3,6-dihydro-2H-pyran-3-ol (4r, Racemic)

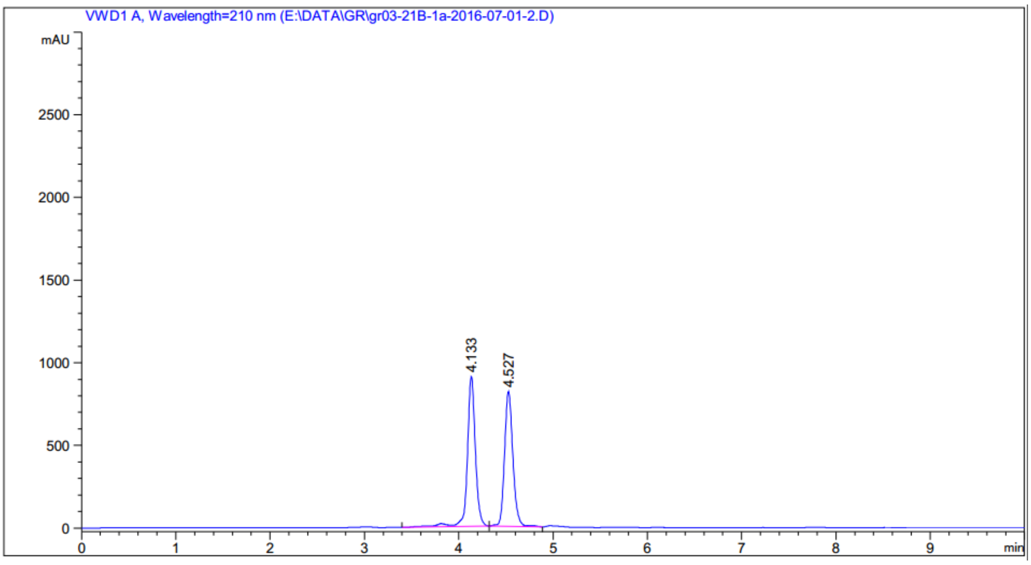

Peak #	RetTime [min]	Type	Width [min]	Area [mAU*s]	Height [mAU]	Area %
1	4.133	VB R	0.0867	5408.98584	906.84277	51.8541
2	4.527	BV R	0.0944	5022.16895	814.17090	48.1459

Totals : 1.04312e4 1721.01367

(2S,3S)-2-pentyl-3,4,5-triphenyl-3,6-dihydro-2H-pyran-3-ol (4r-*ent,* 99%ee)

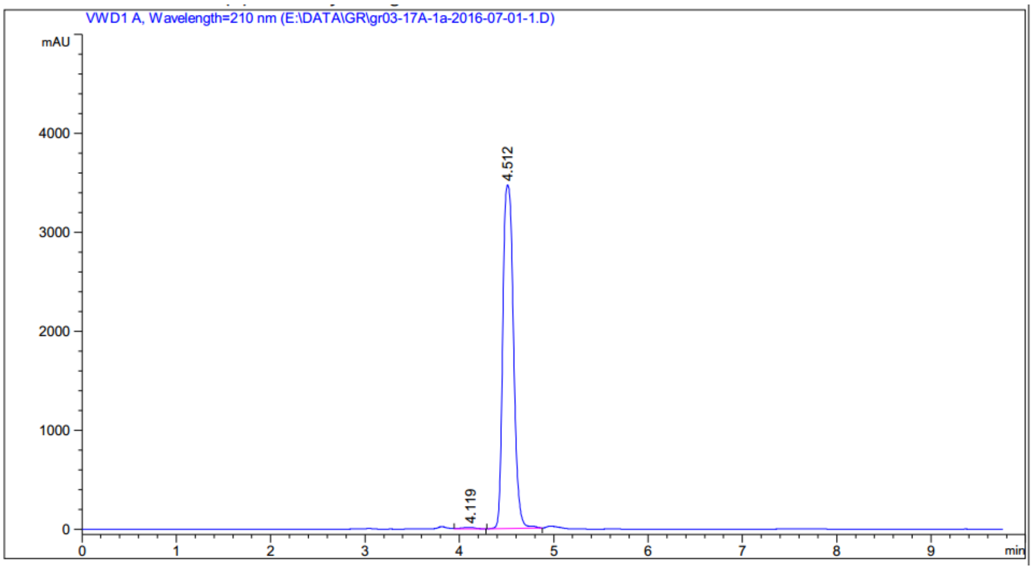

Peak #	RetTime [min]	Type	Width [min]	Area [mAU*s]	Height [mAU]	Area %
1	4.119	VB	0.1371	133.82111	13.22371	0.4923
2	4.512	BV R	0.1239	2.70477e4	3468.51489	99.5077

Totals : 2.71815e4 3481.73860

^{1}H NMR (400MHz,$CDCl_3$) 1,5,6-triphenyl-3,7-dioxabicyclo[4.1.0]heptan-5-ol (5)

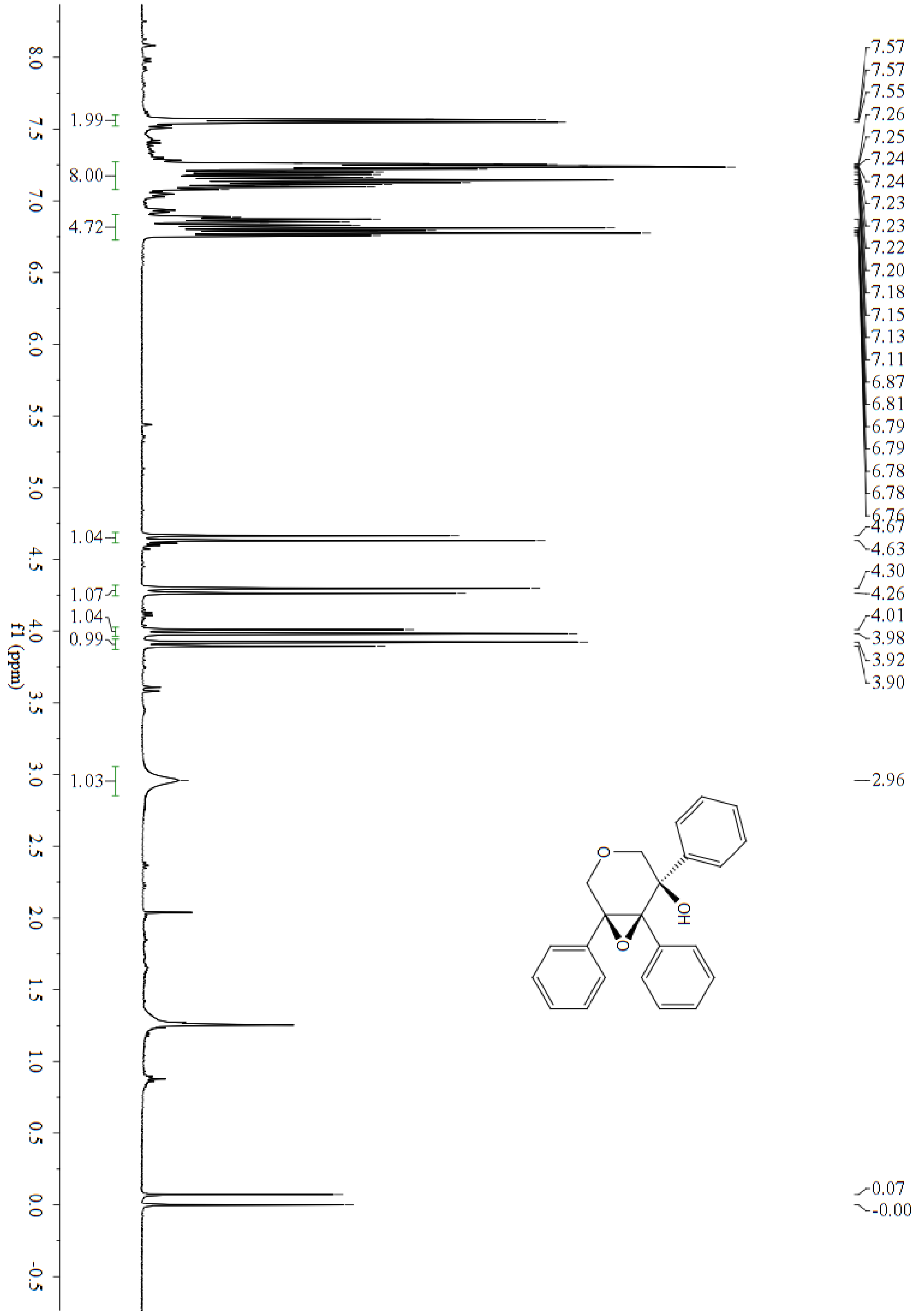

^{13}C NMR (101MHz,$CDCl_3$) 1,5,6-triphenyl-3,7-dioxabicyclo[4.1.0]heptan-5-ol (5)

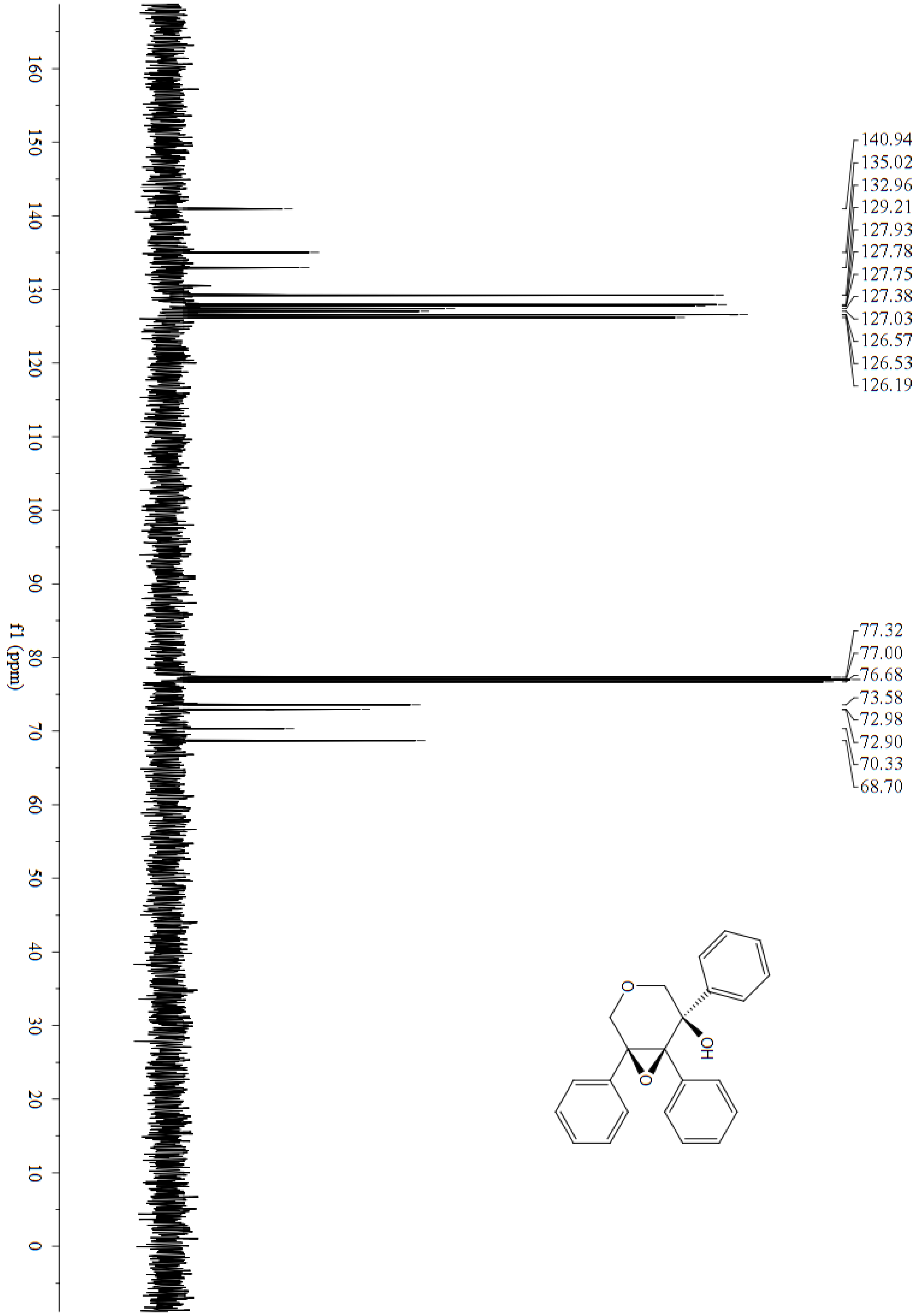

^{1}H NMR (400MHz,CDCl$_3$) 5-hydroxy-4,4,5-triphenyldihydro-2H-pyran-3(4H)-one (6)

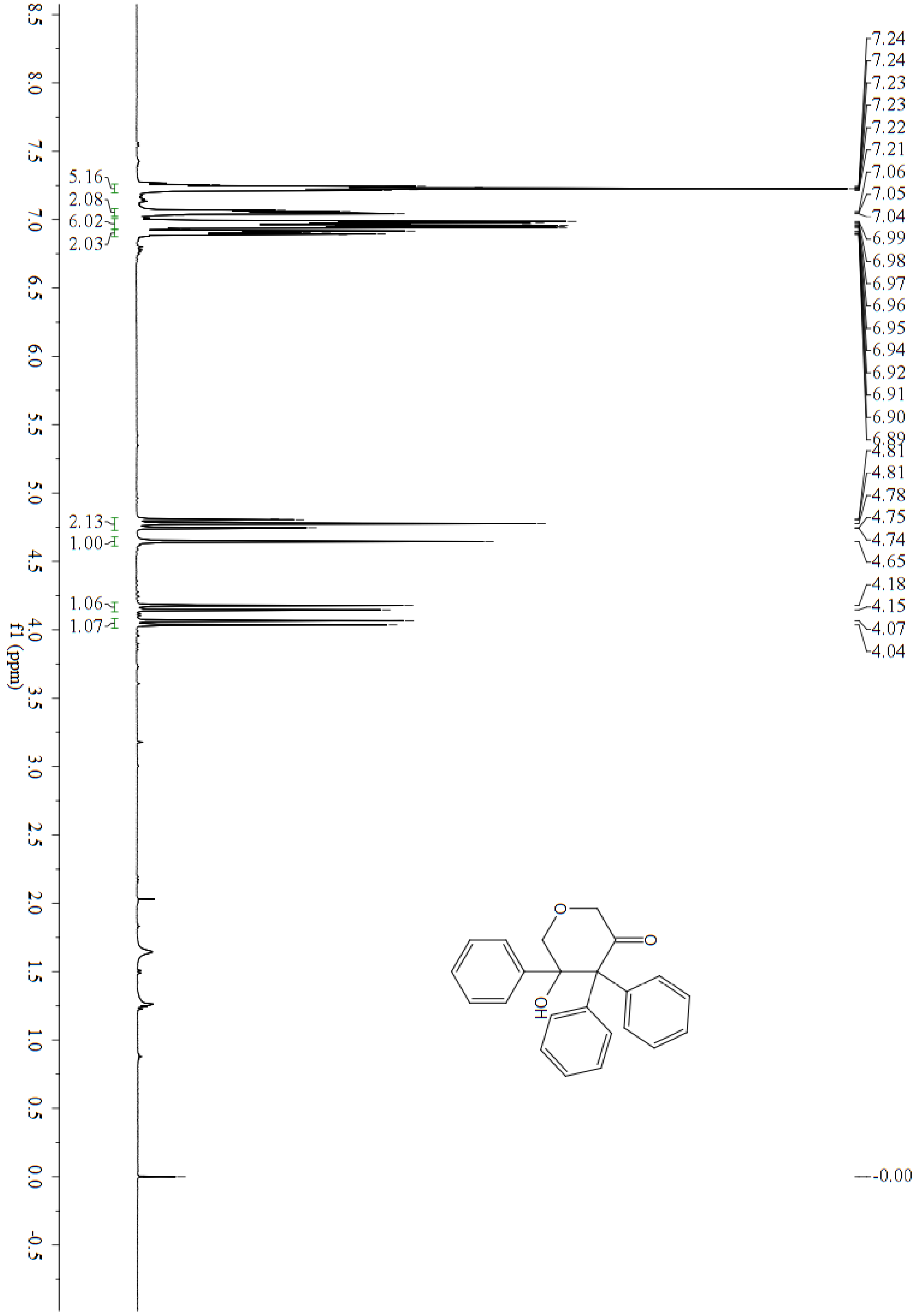

^{13}C NMR (101MHz,$CDCl_3$) 5-hydroxy-4,4,5-triphenyldihydro-2H-pyran-3(4H)-one (6)

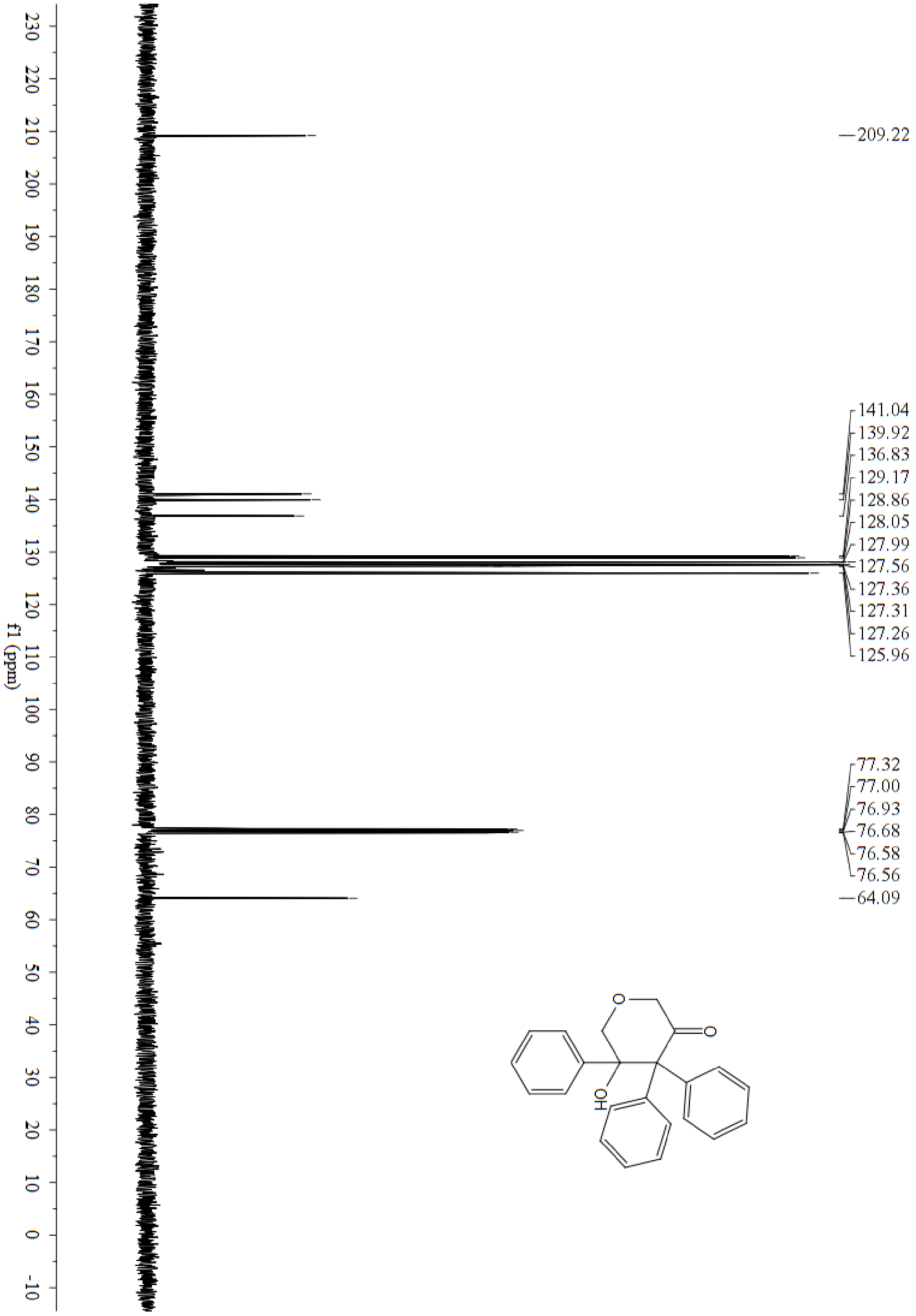

^1H NMR (400MHz,$CDCl_3$) 3,4,5-triphenyl-3,4-dihydro-2H-pyran-3,4-diol (7)

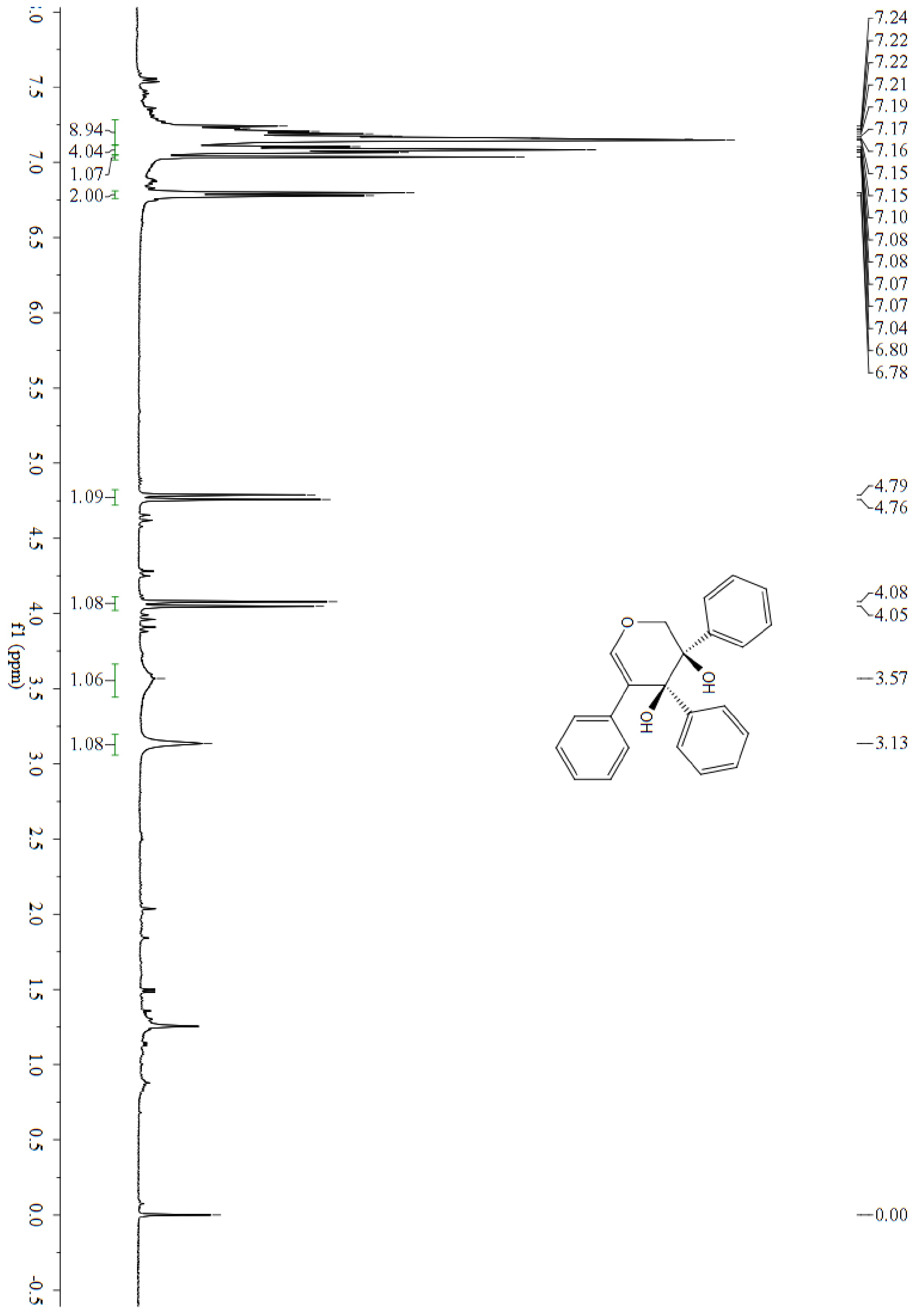

^{13}C NMR (101MHz,$CDCl_3$) 3,4,5-triphenyl-3,4-dihydro-2H-pyran-3,4-diol (7)

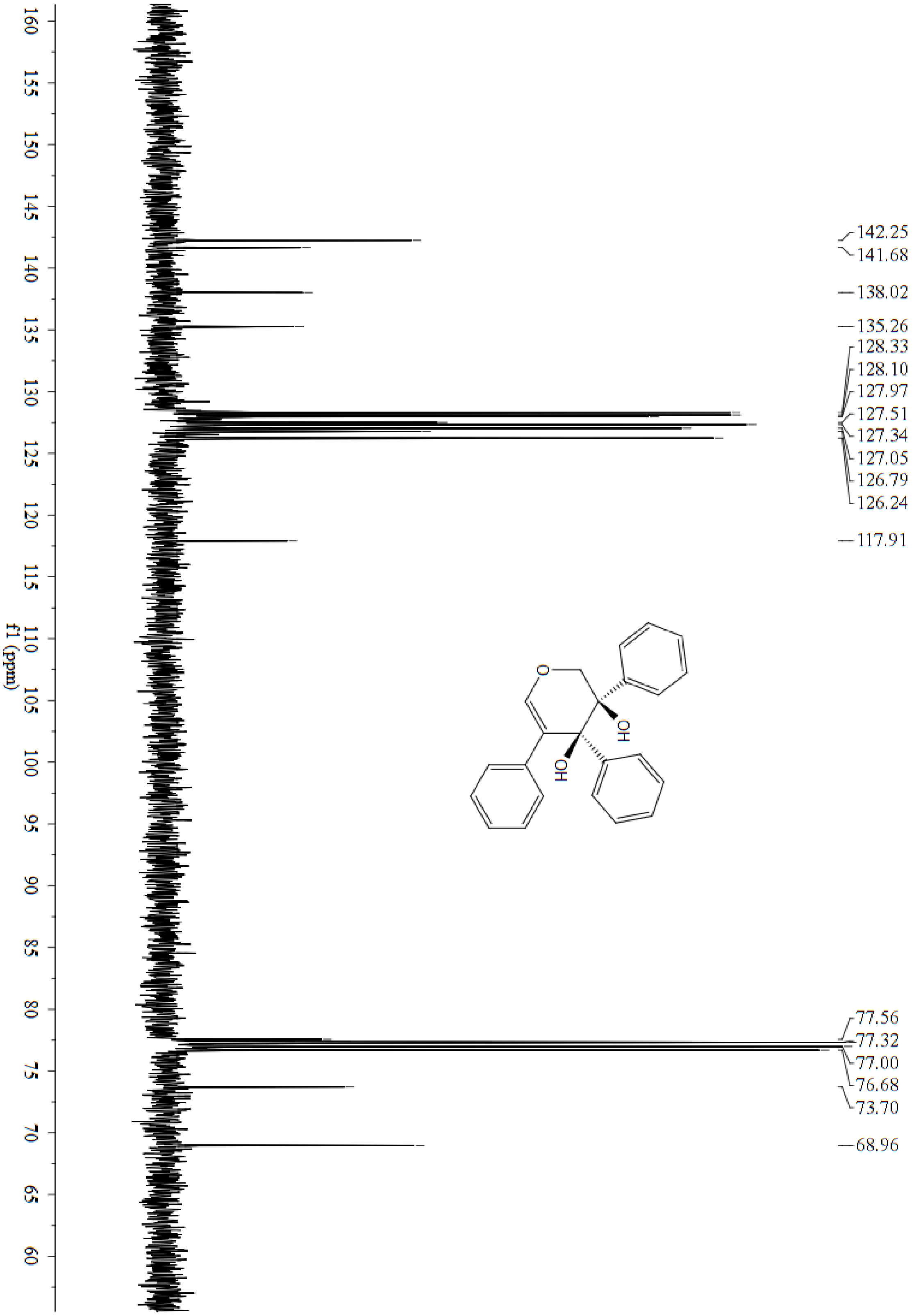

^{1}H NMR (400MHz,DMSO-D6)

5-hydroxy-3,4,5-triphenyl-5,6-dihydro-2H-pyran-2-one (8)

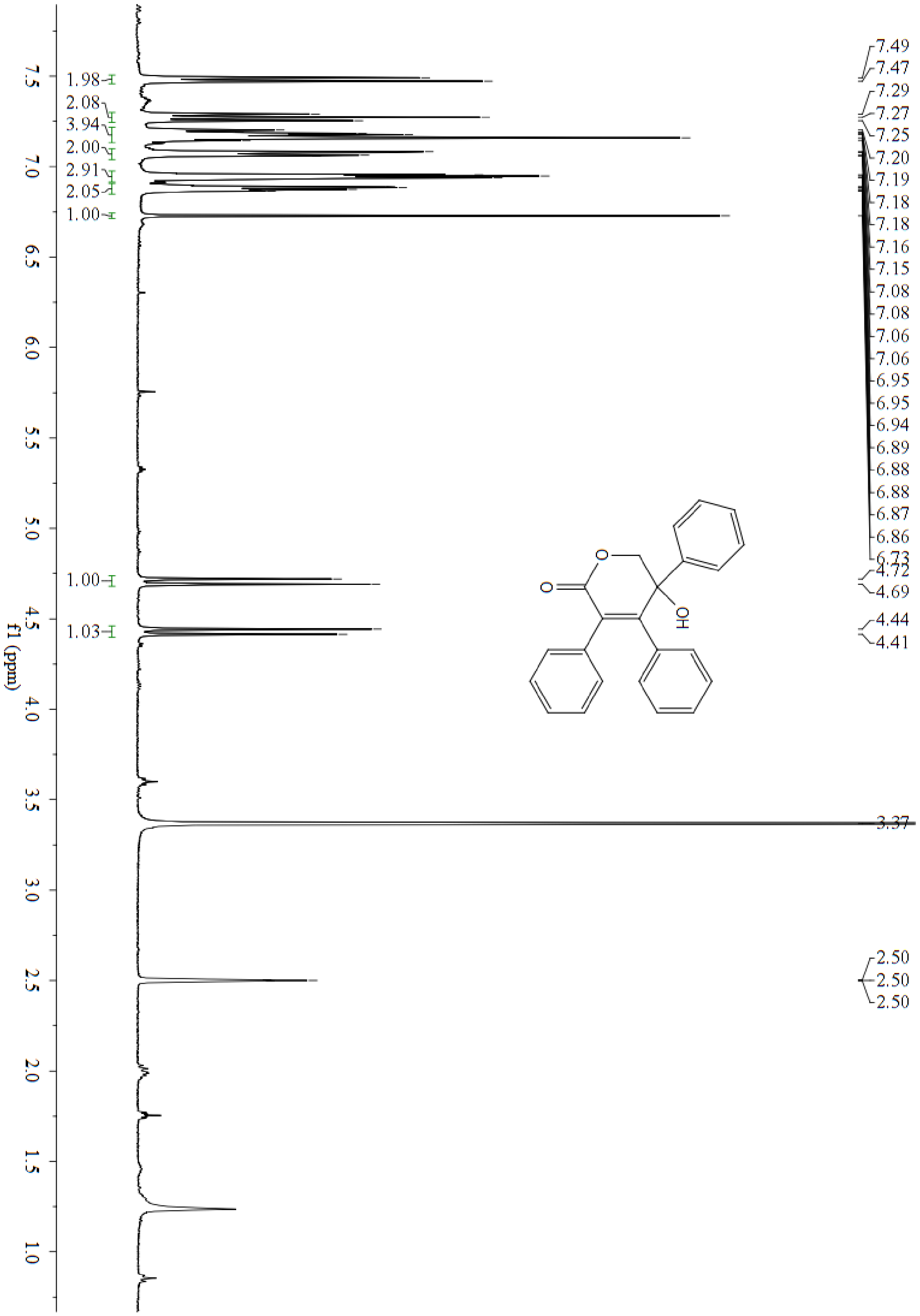

^{13}C NMR (101MHz, DMSO-D6)

5-hydroxy-3,4,5-triphenyl-5,6-dihydro-2H-pyran-2-one (8)

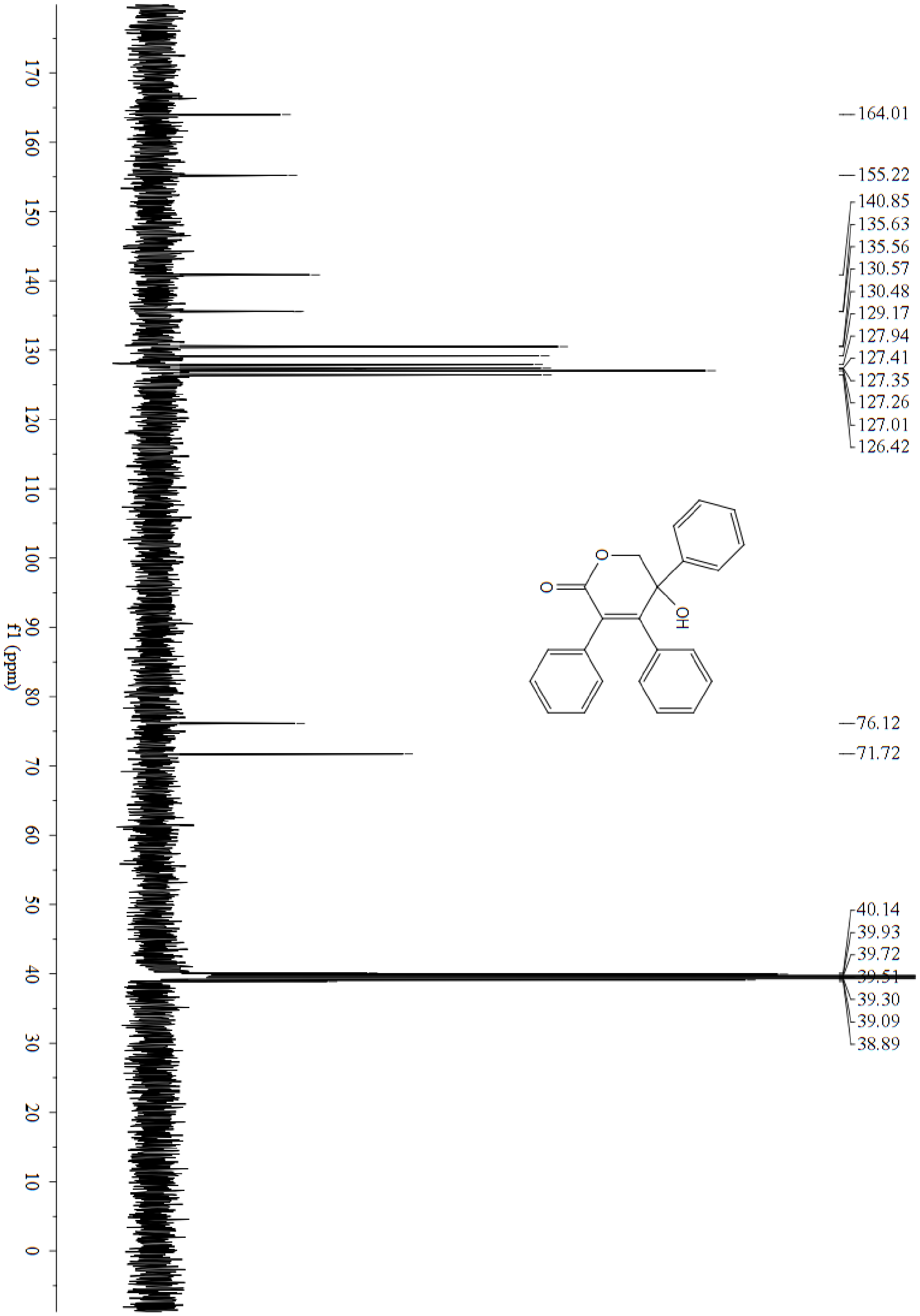

Crystal Structure and Data

1. Crystal Structure and Data for Compound **2i**

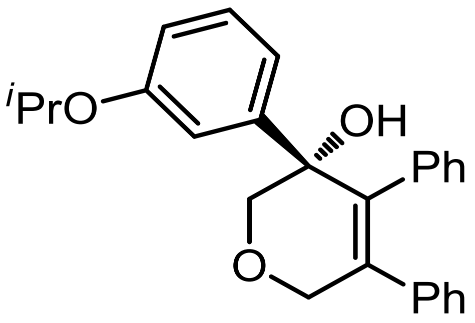

(2i)

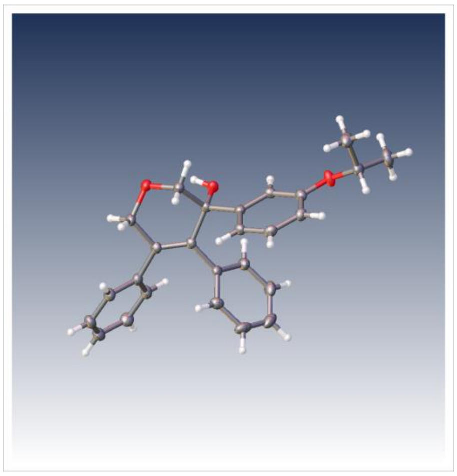

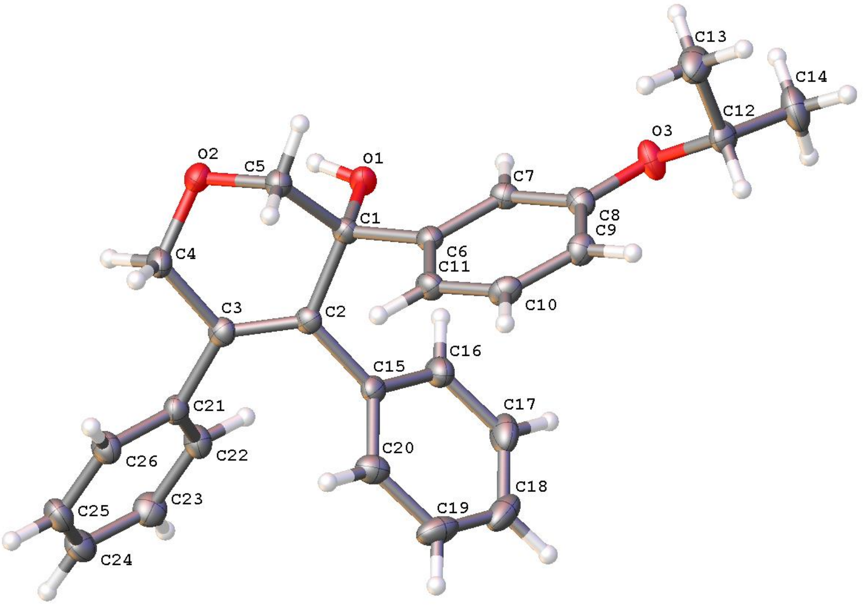

Table 1. Crystal data and structure refinement for cu_dm16687_0m.

Identification code	cu_dm16687_0m	
Empirical formula	C26 H26 O3	
Formula weight	386.47	
Temperature	130 K	
Wavelength	1.54178 Å	
Crystal system	Monoclinic	
Space group	P 1 21 1	
Unit cell dimensions	a = 12.8937(2) Å	α= 90 °.
	b = 6.05140(10) Å	β= 107.5170(10) °.
	c = 14.0680(2) Å	γ = 90 °.

Volume	1046.75(3) Å^3
Z	2
Density (calculated)	1.226 Mg/m^3
Absorption coefficient	0.624 mm^{-1}
F(000)	412
Crystal size	0.18 x 0.15 x 0.12 mm^3
Theta range for data collection	3.294 to 69.828 °.
Index ranges	-15<=h<=14, -7<=k<=7, -15<=l<=16
Reflections collected	7974
Independent reflections	3172 [R(int) = 0.0438]
Completeness to theta = 67.679 °	99.3 %
Absorption correction	Semi-empirical from equivalents
Max. and min. transmission	0.7532 and 0.4779
Refinement method	Full-matrix least-squares on F^2
Data / restraints / parameters	3172 / 1 / 266
Goodness-of-fit on F^2	1.057
Final R indices [I>2sigma(I)]	R1 = 0.0379, wR2 = 0.0942
R indices (all data)	R1 = 0.0383, wR2 = 0.0948
Absolute structure parameter	0.11(12)
Extinction coefficient	0.0200(18)
Largest diff. peak and hole	0.175 and -0.235 e.Å^{-3}

2. Crystal Structure and Data for Compound **4a-*ent***

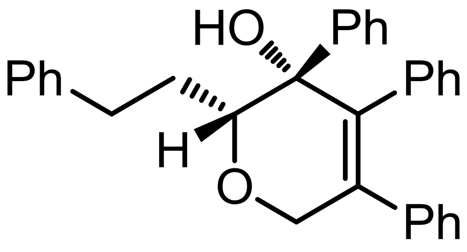

(4a-*ent*)

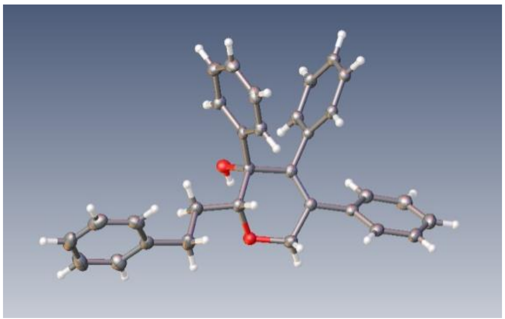

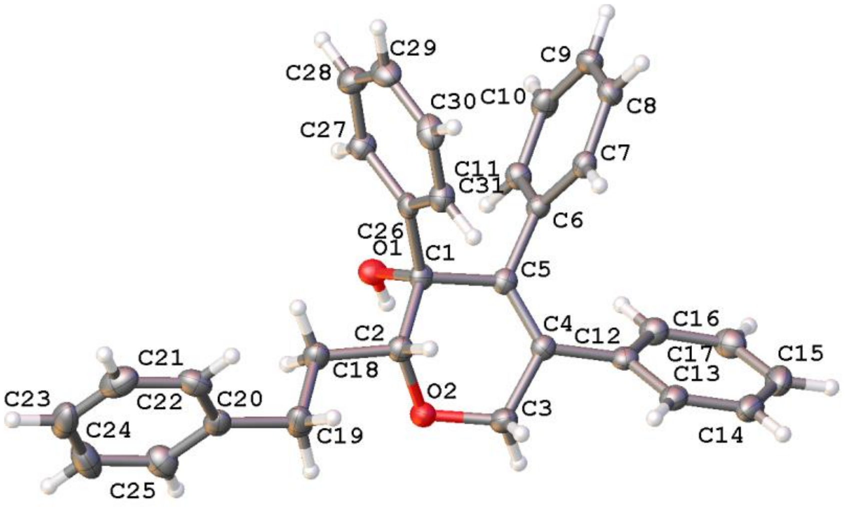

Table 1. Crystal data and structure refinement for cu_dm16550_0m.

Identification code	cu_dm16550_0m	
Empirical formula	C31 H28 O2	
Formula weight	432.53	
Temperature	130 K	
Wavelength	1.54178 Å	
Crystal system	Orthorhombic	
Space group	P 21 21 21	
Unit cell dimensions	a = 5.83870(10) Å	α= 90 °.
	b = 15.6232(3) Å	β= 90 °.
	c = 25.7309(6) Å	γ = 90 °.
Volume	2347.15(8) Å^3	
Z	4	
Density (calculated)	1.224 Mg/m^3	

Absorption coefficient	0.582 mm^{-1}
F(000)	920
Crystal size	0.12 x 0.03 x 0.02 mm^3
Theta range for data collection	3.309 to 69.949 °.
Index ranges	-6<=h<=5, -18<=k<=17, -29<=l<=30
Reflections collected	11626
Independent reflections	4196 [R(int) = 0.0638]
Completeness to theta = 67.679 °	99.8 %
Absorption correction	Semi-empirical from equivalents
Max. and min. transmission	0.7533 and 0.5311
Refinement method	Full-matrix least-squares on F^2
Data / restraints / parameters	4196 / 0 / 299
Goodness-of-fit on F^2	0.990
Final R indices [I>2sigma(I)]	R1 = 0.0395, wR2 = 0.0904
R indices (all data)	R1 = 0.0445, wR2 = 0.0928
Absolute structure parameter	0.0(2)
Extinction coefficient	0.0048(4)
Largest diff. peak and hole	0.144 and -0.183 e.$Å^{-3}$
